# Supplementary material for: Impact of maternal education on response to lifestyle interventions to reduce gestational weight gain: individual participant data meta-analysis
Source: BMJ Open. 2019 Aug 1;9(8):e025620. doi: 10.1136/bmjopen-2018-025620 (PMC6688690; doi:10.1136/bmjopen-2018-025620)
Supplement: Supplementary data [file bmjopen-2018-025620supp001.pdf]

Supplementary Files

Table S1. Study Characteristics of Randomised Control Trials Included in the IPD Meta-Analysis (n = 21)

Author (Year)	Period	Original inclusion/exclusion criteria	Country	Method to measure gestational weight gain (GWG)	Intervention	Intervention Detail	Outcome	Original i-WIP N / N (%) in this analysis
Althuizen *et al*. (2013)	February 2005 to May 2006	Inclusion criteria: primigravida, ability to read and speak Dutch, ≤14 weeks' gestation.	Netherlands	Pre-pregnancy: self-reported. Early pregnancy: measured at 15 weeks' gestation	Mixed	Counselling sessions (The New Life[style] Study) five times during pregnancy (15 minutes each) on weight, physical activity and diet.	Excessive weight gain, BMI, postpartum weight retention, birth weight, macrosomia, preterm birth, gestational diabetes	258<br>187 (72.5%)
Baciuk *et al*. (2008)	Not described	Inclusion criteria: <20 weeks' gestation, singleton pregnancy, receiving prenatal care at the institution and intended to give birth there. Exclusion criteria: engaging in regular physical activity, ≥2 caesarean sections, neurological, cardio-vascular, pulmonary, muscular-skeletal or endocrine disorders, morbid obesity, severe anaemia or vaginal bleeding during pregnancy.	Brazil	Pre-pregnancy: self-reported. Early pregnancy: (estimated from pre-pregnancy as per methods)	Exercise	Water aerobics three times per week (50 minutes each) in an indoor swimming pool.	Maternal cardiovascular capacity, heart rate, blood pressure, experience at delivery and fetal heart rate	71<br>68 (95.8%)
Bogaerts *et al*. (2013)	March 2008 and April 2011	Inclusion: BMI ≥29 kg/m^2. Exclusion criteria: >15 weeks' gestation, pre-existing type 1 diabetes, multiple pregnancy, primary need for nutritional advice and insufficient knowledge of the Dutch language	Belgium	Pre-pregnancy self-reported. Early pregnancy: (estimated from pre-pregnancy as per methods)	Mixed	Two arms. (1) Brochure group: brochure with advice on diet, physical activity and limiting excessive GWG, (2) Prenatal session group: brochure plus four prenatal motivational lifestyle intervention sessions led by a trained midwife.	GWG, Anxiety, feelings of depression	197<br>196 (99.5%)
Haakstad *et al*. (2011)	September 2007 to March 2008	Inclusion criteria: Nulliparous, low pre-pregnancy exercise, sufficient understanding of Norwegian, and ≤24 weeks' gestation. Exclusion criteria: history of miscarriages, severe heart disease, persistent bleeding after 12 weeks' gestation, multiple pregnancy, poorly controlled thyroid disease, pregnancy-induced hypertension or pre-eclampsia, diabetes or gestational diabetes, and other diseases that could interfere with participation.	Norway	Pre-pregnancy self-reported. Early pregnancy: measured at 12-24 weeks' gestation	Exercise	Aerobic dance/strength training class 2-3 times per week (one hour each) for a minimum of 12 weeks. Advised 30 minutes of moderate self-imposed physical activity on the remaining week-days (recorded in a personal training diary).	Infant birth weight, gestational age at delivery, Apgar score.	105<br>83 (79.0%)

Table S1. cont.

Author (Year)	Period	Original inclusion/exclusion criteria	Country	Method to measure GWG	Intervention	Intervention Detail	Outcome	Original i-WIP N / N (%) in this analysis
Harrison *et al*. (2013)	Not described	Inclusion criteria: 12-15 weeks' gestation, BMI ≥25 or ≥23 kg/m^2 if high-risk ethnicity (Polynesian, Asian, and African populations) or ≥30 kg/m^2, increased risk of GDM identified by risk prediction tool, agreement to complete an OGTT at 28 weeks' gestation. Exclusion criteria: multiple pregnancies, type 1 or 2 diabetes, a BMI ≥45 kg/m^2, pre-existing chronic medical conditions, and non-English-speaking	Australia	Pre-pregnancy: self-reported Early pregnancy: measured at 12-15 weeks' gestation.	Mixed	Behaviour change lifestyle intervention with four individual sessions based on the Social Cognitive Theory, at 14-16, 20, 24, and 28 weeks' gestation. Provided with simple healthy eating and physical activity messages. Behavioural change strategies were practised to identify short-term goals, and increase self-efficacy and self-monitoring. Goals were determined individually by the participants. Self-monitoring strategies included pedometers and the use of weight gain charts.	GWG, GDM screening	238<br>187 (78.6%)
Jeffries *et al*. (2009)	July and October 2007	Inclusion criteria: ≤14 weeks' gestation. Exclusion criteria were: age <18 years or >45 years, type 1 or type 2 diabetes mellitus, multiple pregnancy, or non-English speaking.	Australia	Pre-pregnancy self-reported. Early pregnancy: measured at 14 weeks' gestation	Mixed	Advised on optimal GWG based on IOM 1990 guidelines. Provided with a personalised weight measurement card and instructed to record their weight at 16, 20, 24, 28, 30, 32 and 34 weeks' gestation.	GWG, adherence to IOM guidelines (1990)	286<br>236 (82.5%)
Khaledan *et al*. (2010)	Not described	Inclusion criteria: Singleton, healthy pregnancy. Exclusion criteria: chronic disease, multiple pregnancy, complications, risk of preterm delivery, hypertension, severe anaemia, type 1 diabetes with poor control, morbid obesity, low maternal weight, history of completely sedentary lifestyle, heavy smoker.	Iran	Pre-pregnancy: na. Early pregnancy: Not described	Exercise	Aerobic (stepping) and flexibility exercise session three times per week (30-45 minutes each) for eight weeks.	Type of delivery, cause of caesarean section, maternal and fetal weight, birth weight and gestational age	39<br>37 (94.9%)
Khoury *et al*. (2005)	June 1999 to November 2001	Inclusion criteria: 17-20 weeks' gestation, singleton, healthy pregnancy, non-smoker (or stopped ≥5 years previously), 21-38 years, BMI 19-32 kg/m^2. Exclusion criteria: following a vegetarian or Mediterranean diet, immigrant to Norway from non-Western country, high-risk pregnancy, complications in a previous pregnancy, on-going hyperemesis gravidarum or bleeding after gestational week 12	Norway	Pre-pregnancy: na. Early pregnancy: measured at 17-20 week's gestation	Diet	Dietary advice from dietitian at inclusion, 24, 30 and 36 weeks' gestation. Cholesterol-lowering diet; promoted fish, low-fat meats and dairy products, oils, whole grains, fruits, vegetables, and legumes from gestational week 17-20 until delivery.	Maternal, cord, and neonatal plasma lipids and pregnancy-related complications, including hypertensive complications, preterm delivery, and intrauterine growth restriction, birth weight and maternal weight gain.	290<br>198 (68.3%)

Table S1. cont.

Author (Year)	Period	Original inclusion/exclusion criteria	Country	Method to measure GWG	Intervention	Intervention Detail	Outcome	Original i-WIP N / N (%) in this analysis
Nascimento *et al*. (2011)	August 2008 to October 2009	Inclusion criteria: overweight or obese, age >18 years, 14-24 weeks' gestation. Exclusion criteria: multiple gestations, exercising regularly, conditions that contraindicate exercise	Brazil	Pre-pregnancy self-reported. Early pregnancy: measured at 14-24 weeks' gestation	Exercise	One exercise class per week (40 minutes each), under the guidance of a trained physical therapist. Light-intensity to moderate-intensity exercises. Advised to exercise at home five times per week.	GWG, maternal arterial blood pressure and perinatal outcome, perception of quality of life.	82 / 77 (93.9%)
Oostdam *et al*. (2012)	November 2007 and April 2010	Inclusion criteria: obese or overweight and at least one of the following characteristics: history of macrosomia; history of GDM; or first-grade relative with type 2 diabetes. Exclusion criteria: >20 weeks' gestation; <18 years; inadequate knowledge of Dutch language; GDM; hypertension; alcohol abuse; drug abuse; use of any medication that affects insulin secretion or insulin sensitivity; chronic disease that could prevent the woman from implementing the study protocol.	Netherlands	Pre-pregnancy self-reported. Early pregnancy: measured at 15-20 weeks' gestation	Exercise	Aerobic and strength exercise classes (FitFor2 Study). Two days per week (60 minutes each), under supervision of physiotherapist, from 14-20 weeks' gestation to end of pregnancy.	Fasting blood glucose, insulin, HbA1c; body weight, BMI, daily physical activity, birth weight and fetal growth.	124 / 75 (60.5%)
Petrella *et al*. (2014)	April 2011 to October 2011	Inclusion criteria: Pre-pregnancy BMI ≥25 kg/m^2, >18 years and singleton pregnancy. Exclusion criteria: twin pregnancy, chronic diseases (i.e. diabetes mellitus, chronic hypertension, untreated thyroid diseases), previous GDM, smoker, previous bariatric surgery, regular physical activity, dietary supplements or herbal products known to affect body weight, other medical conditions that might affect body weight, and plans to deliver outside the centre	Italy	Pre-pregnancy self-reported. Early pregnancy: measured at 12 weeks' gestation	Mixed	Diet, physical activity and counselling about GWG. Dietary advice: low glycaemic index diet, three main meals and three snacks (1500 kcal/day). Physical activity advice: 3 days per week of moderate intensity activity (30 minutes each), monitored using pedometer. One-hour counselling session about appropriate GWG.	Excessive weight gain, gestational diabetes mellitus, gestational hypertension and preterm delivery.	61 / 58 (95.1%)

Table S1. cont.

Author (Year)	Period	Original inclusion/exclusion criteria	Country	Method to measure GWG	Intervention	Intervention Detail	Outcome	Original i-WIP N / N (%) in this analysis
Phelan *et al*. (2011)	2006 to 2008	Inclusion criteria: 10 - 16 week's gestation, BMI 19.8 - 40 kg/m², non-smoking, >18 years, fluency in English, access to a telephone, and a singleton pregnancy. Exclusion criteria: self-reported major health or psychiatric diseases, weight loss during pregnancy, or a history of >3 miscarriages	USA	Pre-pregnancy self-reported. Early pregnancy: measured at 10-16 weeks' gestation	Mixed	Behavioural intervention with diet and physical activity advice (Fit for Delivery Study). One face-to-face visit to discuss appropriate GWG, physical activity (30 min of walking most days of the week), and calorie goals (20 kcal/kg), with daily self-monitoring of eating, exercise, and weight. Provided with weight scales, food records, pedometers, weekly reminders, personalised graphs of weight gained with feedback, 3 supportive phone calls from the dietitian. If gained over or under weight gain guidelines received 2 additional supportive phone calls per month with meal plans and goals until weight gains returned to appropriate amounts.	GWG and the proportion who returned to pre-gravid weights by 6 months' postpartum.	401 / 395 (98.5%)
Poston *et al*. (2015)	March 2009 to June 2014	Inclusion criteria: >16 years, BMI ≥30 kg/m², singleton pregnancy, 15 - 18(+6) weeks' gestation. Exclusion criteria: unwilling or unable to give informed consent; underlying disorders, including a pre-pregnancy diagnosis of essential hypertension, diabetes, renal disease, systemic lupus erythematosus, antiphospholipid syndrome, sickle- cell disease, thalassaemia, coeliac disease, thyroid disease, and current psychosis; or currently being prescribed metformin.	UK	Pre-pregnancy: na. Early pregnancy: measured at 15-18 week's gestation	Mixed	Behavioural intervention with diet and physical activity advice (UPBEAT Study). One individual interview plus one group/individual session per week (60 minutes each) for 8 weeks. Advised to self-monitor, identify, and problem-solve of barriers to behaviour change and enlist social support. Dietary intervention aimed to promote a healthy pattern of eating and low glycaemic index diet. Exercise advice focused on incremental increases in walking from a pedometer- assessed baseline, tailored to pre-existing activities.	Gestational diabetes and large-for-gestational-age infants (≥90th customised birth weight centile)	1554 / 378 (24.3%)

Table S1. cont.

Author (Year)	Period	Original inclusion/exclusion criteria	Country	Method to measure GWG	Intervention	Intervention Detail	Outcome	Original i-WIP N / N (%) in this analysis
Rauh *et al*. (2013)	February 2010 and August 2011	Inclusion criteria: >18 years; singleton pregnancy; <18 weeks' gestation; BMI ≥18.5 kg/m², and sufficient German. Exclusion criteria: any condition preventing physical activity, GDM and uncontrolled chronic diseases that may affect weight development like thyroid dysfunction or psychiatric diseases	Germany	Pre-pregnancy self-reported. Early pregnancy: measured at 10-16 weeks' gestation	Mixed	Individual counselling sessions on nutrition, physical activity, and GWG monitoring (FeLIPO Study). Twice during pregnancy; 20th (60 minutes) and 30th (30 minutes) week of gestation. Provided with general healthy lifestyle information; prompted to self-monitor behaviour by recording diet, physical activity, and weight gain using weight gain charts; and to set behavioural goals based on the baseline situation (BMI, diet, physical activity) and individual preferences.	Weight gain in excess of IOM recommendations, and weight retention at 4 months' postpartum	250 / 231 (92.4%)
Renault *et al*. (2014)	March 2009 to March 2012	Inclusion criteria: >18 years, singleton pregnancy, normal scan in weeks 11-14, <16 weeks' gestation, sufficient Danish. Exclusion criteria: multiple pregnancy, pre-gestational diabetes, or other serious diseases limiting level of physical activity, previous bariatric surgery, or alcohol or drug abuse.	Denmark	Pre-pregnancy self-reported. Early pregnancy: measured at 11-16 weeks' gestation	Mixed	Physical activity intervention with or without dietary intervention (TOP Study). Physical activity advice: daily step count of 11,000, monitored with pedometer. Diet: hypocaloric (1200-1675 kcal), low-fat, Mediterranean-style diet and contact with an experienced dietitian every 2 weeks, alternating between outpatient visits and phone contacts (measurement of weight, encouragement, and correcting advice on the diet).	GWG, pregnancy complications and delivery complications.	425 / 370 (87.1%)
Sagedal *et al*. (2017)	September 2009 and February 2013	Inclusion criteria: Nulliparous, singleton pregnancy, ≤20 weeks' gestation, pre-pregnancy BMI of ≥19 kg/m^2, sufficient in Norwegian or English, and provided signed, informed consent. Exclusion criteria: pre-existing diabetes, disabilities precluding participation in a physical fitness programme, continued substance abuse, or planned relocation outside of the study area before delivery.	Norway	Pre-pregnancy self-reported. Early pregnancy: measured at ≤20 weeks' gestation	Mixed	Lifestyle intervention with diet and physical activity components (Norwegian Fit for Delivery Study). Dietary advice provided by telephone, with one initial consultation and follow-up 4–6 weeks later (20 minutes). Dietary advice aimed to increase awareness of food choices. GWG based on pre-pregnancy BMI provided. Physical activity: access to exercise classes at a local gym twice/week (60 minutes). Provided with booklets, access to study website, invitation to one cooking class and to an evening meeting.	GWG, birth weight of term infants, maternal fasting glucose levels at 30 weeks of gestation, maternal percentage of fat, incidence of operative deliveries, postpartum weight	606 / 576 (95.0%)

Table S1. cont.

Author (Year)	Period	Original inclusion/exclusion criteria	Country	Method to measure GWG	Intervention	Intervention Detail	Outcome	Original i-WIP N / N (%) in this analysis
Stafne *et al*. (2012)	April 2007 to June 2009 and October 2007 to January 2009	Inclusion criteria: Residing within 30 min drive from the hospital and available to attend weekly appointment, ≥18 years, singleton pregnancy. Exclusion criteria: High-risk pregnancies, diseases that could interfere with participation.	Norway	Pre-pregnancy: na. Early pregnancy: measured at 18-22 week's gestation	Exercise	Exercise sessions, once per week for 12 weeks (60 minutes each), in groups of 8–15 women, instructed by a physiotherapist. Offered between 20 and 36 weeks of pregnancy. Included aerobic activity, strength training and balance exercises. Women encouraged to follow a written home exercise program at least twice per week (45 minutes each).	Self-reported lumbopelvic pain and sick leave due to lumbopelvic pain.	855 / 699 (81.8%)
Vinter *et al*. (2011)	October 2007 to October 2010	Inclusion criteria: 18–40 years, 10–14 weeks' gestation, BMI 30–45 kg/m^2. Exclusion criteria: chronic medical conditions, prior serious obstetric complications, alcohol or drug abuse, non-Danish speaking, multiple pregnancy, positive OGTT.	Denmark	Pre-pregnancy: self-reported. Early pregnancy: measured at 10-14 week's gestation	Mixed	Dietary counselling and physical activity (The LiP Study - Lifestyle in Pregnancy). Four dietary counselling by dietitians at 15, 20, 28, and 35 weeks' gestation. Dietary advice was based on the official Danish recommendations, and aimed to limit GWG to 5 kg. Provided with free full-time membership in a fitness centre for 6 months, with exercise classes once per week (60 minutes each) with physiotherapists. Encouraged to be moderately physically active every day (30–60 minutes per day), provided with a pedometer to motivate and improve daily activity.	GWG, preeclampsia, pregnancy-induced hypertension, GDM, caesarean section, macrosomia, and admission to neonatal intensive care unit.	304 / 292 (96.1%)
Vitolo *et al*. (2011)	January 2007 to May 2008	Inclusion criteria: 10-29 weeks' gestation. Exclusion criteria: positive HIV test previous diagnosis of diabetes, hypertension, anaemia, any conditions preventing women from undertaking, exercise in pregnancy, and >35 years	Brazil	Pre-pregnancy: self-reported. Early pregnancy: collected in first trimester (method not described)	Diet	Dietary counselling provided according to nutritional needs. Underweight women advised to increase energy density of the diet, include two high-energy snacks and fruit daily. Well-nourished women advised to consume six portions fruit and vegetables and restrict energy dense foods. Overweight women were advised on meal timing, portion sizes, to restrict energy dense and processed foods.	GWG, gestational diabetes, pre-eclampsia, infant birth and premature delivery (<37wks), 1st and 5th min Apgar score, head circumference, type of delivery, birth weight <2500g	315 / 268 (85.1%)

Table S1. cont.

Author (Year)	Period	Original inclusion/exclusion criteria	Country	Method to measure GWG	Intervention	Intervention Detail	Outcome	Original i-WIP N / N (%) in this analysis
Walsh *et al*. (2012)	January 2007 to January 2011	Inclusion criteria: Secundigravid women, previous macrosomic infant (≥4 kg). Exclusion criteria: underlying medical disorders, including a previous history of gestational diabetes, use of any drugs, unable to provide full informed consent, <18 years, >18 weeks' gestation, and multiple pregnancy.	Ireland	Pre-pregnancy: na. Early pregnancy: measured at 12-15 week's gestation	Diet	One dietary education session (2 hours), with the research dietitian, at 15 weeks' gestation, groups of 2-6 women. Reinforcement of diet at 28 and 34 weeks' gestation. Advised on a low glycaemic index diet, and general healthy eating guidelines for pregnancy. Received written resources about low glycaemic index diet.	Birth weight, GWG, glucose intolerance.	800 / 517 (64.6%)
Wolff *et al*. (2008)	Not described	Inclusion criteria: non-diabetic, Caucasian, BMI >30 kg/m^2, 15 weeks' gestation. Exclusion criteria: smoker, <18 or >45 years, multiple pregnancy, medical complications known to affect fetal growth adversely or to contraindicate limitation of weight gain.	Denmark	Pre-pregnancy: self-reported. Early pregnancy: measured at 15 week's gestation	Diet	Dietary consultations with a dietitian, 10 times during pregnancy (60 minutes each). Advised to eat a healthy diet according to the official Danish dietary recommendations. Aim to restrict GWG to 6–7kg.	GWG, pregnancy-induced increases in insulin, leptin, and glucose. Weight retentions at 4 weeks' postpartum	59 / 55 (93.2%)

GWG: gestational weight gain; BMI: body mass index; GDM; gestational diabetes; OGTT: oral glucose tolerance test; IOM: Institute of Medicine

Table S2. Global classification of risk of bias on study level for trials included

Study Team	Item 1: Randomisation	Item 2: Allocation concealment	Item 3: Blinding of participants	Item 4: Blinding of outcome Assessment	Item 5: Incomplete outcome data	Item 6: Selective reporting	Global Risk of bias
Althuizen *et al*. (2013)	Low	Low	Unclear	Low	Low	Unclear	Low/Medium
Baciuk *et al*. (2008)	Low	Low	High	Low	Low	Low	Low/Medium
Bogaerts *et al*. (2013)	Low	Unclear	High	High	Low	Low	High
Haakstad *et al*. (2011)	Low	Low	Unclear	Low	Low	High	Low/Medium
Harrison *et al*. (2013)	Low	Low	Unclear	Low	Low	Low	Low/Medium
Jeffries *et al*. (2009)	Low	Low	Unclear	Low	Low	Low	Low/Medium
Khaledan *et al*. (2010)	Low	Unclear	High	High	Low	Low	High
Khoury *et al*. (2005)	Low	Low	Unclear	Low	Low	Low	Low/Medium
Nascimento *et al*. (2011)	Low	Low	High	High	Low	Low	High
Oostdam *et al*. (2012)	Low	Low	High	Low	High	Low	High
Petrella *et al*. (2014)	Low	High	High	High	Low	High	High
Phelan *et al*. (2011)	Low	Low	Unclear	Low	Low	Low	Low/Medium
Poston *et al*. (2015)	Unclear	Unclear	High	High	Unclear	Unclear	High
Rauh *et al*. (2013)	Low	Low	High	High	Low	Unclear	High
Renault *et al*. (2014)	Low	Low	High	High	Low	High	High
Sagedal *et al*. (2016)	Low	Low	Unclear	Low	Unclear	Unclear	Low/Medium
Stafne *et al*. (2012)	Low	Low	High	High	Low	Low	High
Vinter *et al*. (2011)	Low	Low	High	High	Low	High	High
Vitolo *et al*. (2011)	Low	High	High	Low	Low	High	High
Walsh *et al*. (2012)	Low	Low	High	Unclear	Low	High	Low/Medium
Wolff *et al*. (2008)	Low	Low	Unclear	High	High	Low	High

Legend		
⊕ Low	⊖ High	? Unclear

Table S3. Individual Study Results – Gestational Weight Gain per Week

		Intervention			Control			Mean Difference (95% CI)	*P**
**Study name**	**Education**	**N**	**Mean**	**SD**	**N**	**Mean**	**SD**		
Althuizen *et al*. (2013)	Lower	49	0.39	0.34	49	0.47	0.26	-0.08 (-0.02, 0.04)	0.205
	Higher	40	0.47	0.20	49	0.43	0.27	0.04 (-0.06, 0.15)	0.391
Baciuk *et al*. (2008)	Lower	29	0.48	0.23	31	0.51	0.25	-0.04 (-0.16, 0.09)	0.561
	Higher	4	0.41	0.19	4	0.52	0.31	-0.11 (-0.55, 0.33)	0.557
Bogaerts *et al*. (2013)	Lower	73	0.31	0.26	40	0.41	0.25	-0.10 (-0.21, 0.01)	0.064
	Higher	60	0.29	0.24	23	0.42	0.28	-0.14 (-0.27, -0.01)	0.044
Haakstad *et al*. (2011)	Lower	7	0.47	0.15	6	0.39	0.18	0.08 (-0.13, 0.29)	0.411
	Higher	34	0.48	0.17	36	0.51	0.17	-0.03 (-0.11, 0.05)	0.432
Harrison *et al*. (2013)	Lower	56	0.41	0.18	56	0.46	0.25	-0.05 (-0.13, 0.03)	0.197
	Higher	40	0.44	0.19	35	0.47	0.26	-0.02 (-0.13, 0.08)	0.668
Jeffries *et al*. (2009)	Lower	42	0.45	0.18	32	0.47	0.14	-0.03 (-0.12, 0.05)	0.498
	Higher	83	0.43	0.17	79	0.46	0.16	-0.03 (-0.08, 0.03)	0.401
Khaledan *et al*. (2010)	Lower	11	0.61	0.26	14	0.73	0.33	-0.12 (-0.38, 0.13)	0.336
	Higher	6	0.55	0.23	6	0.51	0.17	0.04 (-0.22, 0.30)	0.736
Khoury *et al*. (2005)	Lower	21	0.56	0.20	14	0.54	0.17	0.01 (-0.12, 0.15)	0.822
	Higher	74	0.52	0.19	89	0.57	0.17	-0.05 (-0.10, 0.01)	0.108
Nascimento *et al*. (2011)	Lower	34	0.37	0.22	39	0.40	0.22	-0.03 (-0.13, 0.07)	0.562
	Higher	3	0.40	0.07	1	0.80	na	-0.40 (-0.73, -0.07)	0.035
Oostdam *et al*. (2012)	Lower	28	0.41	0.34	24	0.32	0.20	0.09 (-0.07, 0.25)	0.245
	Higher	10	0.40	0.21	13	0.44	0.21	-0.04 (-0.22, 0.14)	0.680
Petrella *et al*. (2014)	Lower	25	0.22	0.29	27	0.33	0.22	-0.11 (-0.26, 0.04)	0.137
	Higher	5	0.34	0.12	1	0.35	na	-0.01 (-0.39, 0.37)	0.950
Phelan *et al*. (2011)	Lower	27	0.50	0.25	36	0.46	0.19	0.04 (-0.07, 0.15)	0.469
	Higher	170	0.48	0.16	162	0.52	0.19	-0.03 (-0.07, 0.01)	0.094
Poston *et al*. (2015)	Lower	113	0.26	0.20	120	0.27	0.22	0.00 (-0.06, 0.06)	0.853
	Higher	66	0.20	0.21	79	0.26	0.20	-0.06 (-0.14, 0.01)	0.116
Rauh *et al*. (2013)	Lower	64	0.45	0.15	43	0.45	0.18	-0.03 (-0.11, 0.04)	0.904
	Higher	89	0.43	0.13	35	0.47	0.15	-0.07 (-0.13, -0.01)	0.120
Renault *et al*. (2014)	Lower	56	0.38	0.22	28	0.48	0.22	-0.09 (-0.20, 0.01)	0.082
	Higher	186	0.35	0.21	100	0.39	0.19	-0.04 (-0.09, 0.01)	0.169
Sagedal *et al*. (2017)	Lower	89	0.54	0.21	93	0.57	0.21	-0.03 (-0.09, 0.03)	0.339
	Higher	199	0.48	0.20	195	0.52	0.19	-0.04 (-0.08, 0.00)	0.037
Stafne *et al*. (2012)	Lower	31	0.53	0.15	38	0.59	0.23	-0.06 (-0.16, 0.03)	0.178
	Higher	339	0.51	0.17	291	0.52	0.17	-0.01 (-0.03, 0.02)	0.617
Vinter *et al*. (2011)	Lower	59	0.31	0.25	69	0.36	0.22	-0.06 (-0.15, 0.03)	0.212
	Higher	85	0.33	0.20	79	0.38	0.22	-0.05 (-0.12, 0.02)	0.158
Vitolo *et al*. (2011)	Lower	79	0.41	0.18	83	0.45	0.21	-0.04 (-0.10, 0.02)	0.199
	Higher	61	0.42	0.15	45	0.49	0.22	-0.07 (-0.15, 0.00)	0.053
Walsh *et al*. (2012)	Lower	60	0.46	0.16	62	0.52	0.21	-0.06 (-0.13, 0.01)	0.077
	Higher	188	0.49	0.16	207	0.53	0.18	-0.04 (-0.08, -0.01)	0.021
Wolff *et al*. (2008)	Lower	26	0.26	0.16	27	0.40	0.24	-0.14 (-0.25, -0.02)	0.021
	Higher	0†	na		2	0.38	0.24	na	na

Mean difference is calculated as intervention minus control
* P-value is independent-sample t-test between intervention and control
† T-test cannot be computed because at least one of the groups is empty.
Lower education: secondary education or less. Higher education: at least some tertiary education

Table S4. Comparison of maternal characteristics among women included and excluded in the IPD meta-analysis.

	Included		Excluded		*P**
	**N**		**N**		
**Early pregnancy weight** (kg) [a]	5183	78.60 (18.56)	4814	87.78 (51.06)	0.002
**Pre-pregnancy weight** (kg) [a]	5183	76.06 (18.74)	2398	85.82 (123.03)	0.030
**Height** (cm) [a]	5183	166.07 (7.03)	6697	169.30 (63.03)	<0.001
**Pre-pregnancy BMI** (kg/m^2) [a]	5183	27.57 (6.55)	2615	27.31 (12.56)	<0.001
**Gestational age** (weeks)					
**Baseline** [c]	5183	14.5 (4.0 - 32.0)	4478	14.0 (11.0 – 17.0)	<0.001
**Final follow-up** [c]	5183	36.6 (24.0 - 44.0)	6995	38.0 (35.0 – 41.0)	<0.001
**Delivery** [c]	5172	39.7 (26.0 - 44.0)	7007	40.0 (38.85 – 41.0)	0.022
**Infant birth weight** (g) [a]	5164	3531.73 (547.90)	7026	3701.22 (1479.82)	<0.001
**Maternal age** (years) [a]	5183	29.90 (4.97)	7041	30.80 (8.36)	<0.001
**Ethnicity**					
Caucasian [b]	3725	3291 (88.3)	6415	5588 (87.1)	<0.001
Asian [b]		84 (0.02)		231 (0.04)	
Black [b]		123 (0.03)		462 (0.08)	
Central/South American [b]		88 (0.02)		47 (0.01)	
Middle East (including Iran & Turkey) [b]		63 (0.01)		13 (0.00)	
Other [b]		76 (0.02)		74 (0.01)	

Data are presented as [a] mean (SD) [b] n (%) and [c] mean (range of minimum and maximum).

*P-value: T-tests and chi-square

Figure S1. Assessment of small study effects on trials in IPD meta-analysis of low educational attainment and gestational weight gain per week, controlling for BMI and intervention.

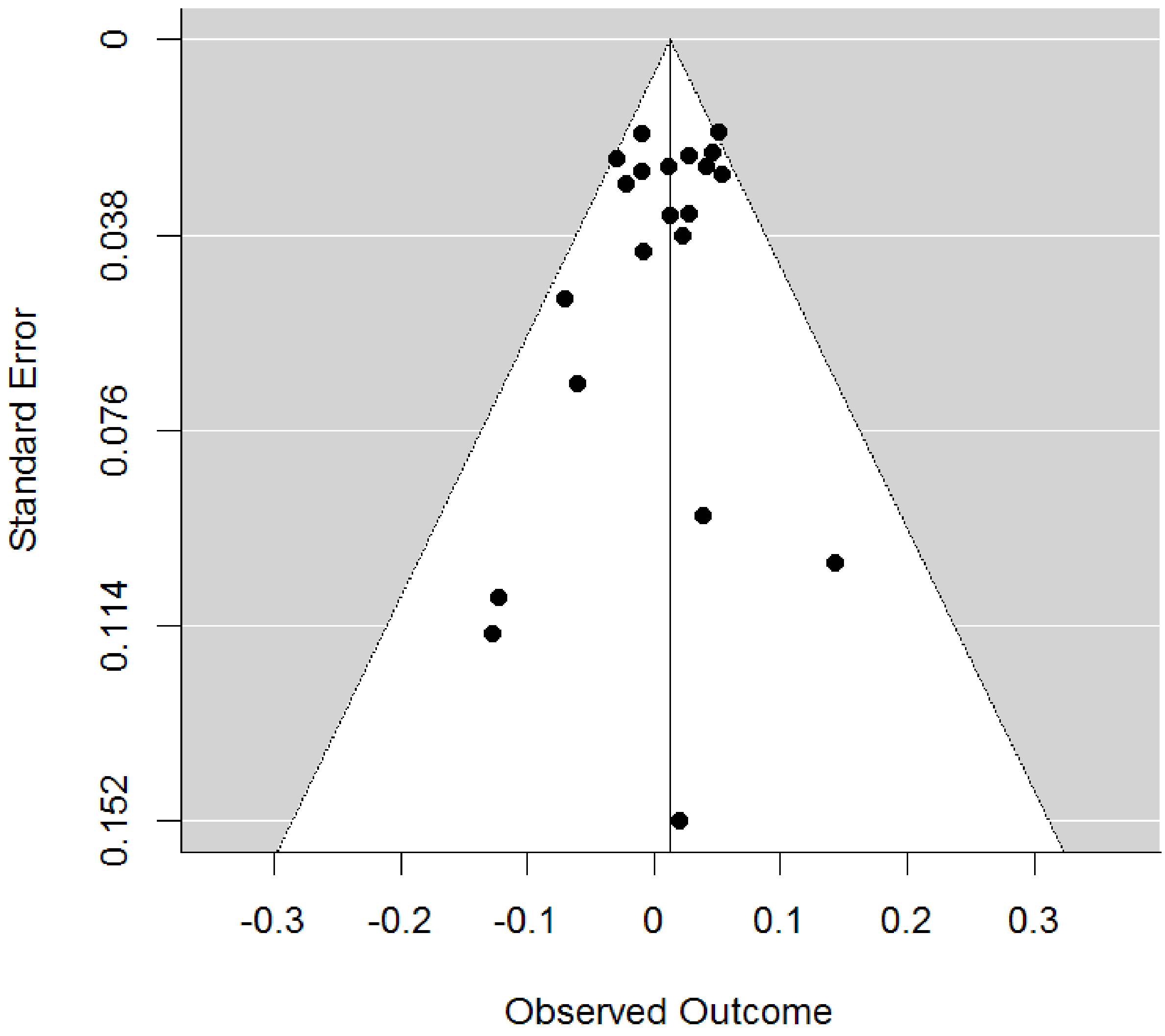

Figure S2. Forest plot of the IPD meta-analysis of low educational attainment (treatment effect [TE]) and gestational weight gain per week, controlling for BMI and intervention.

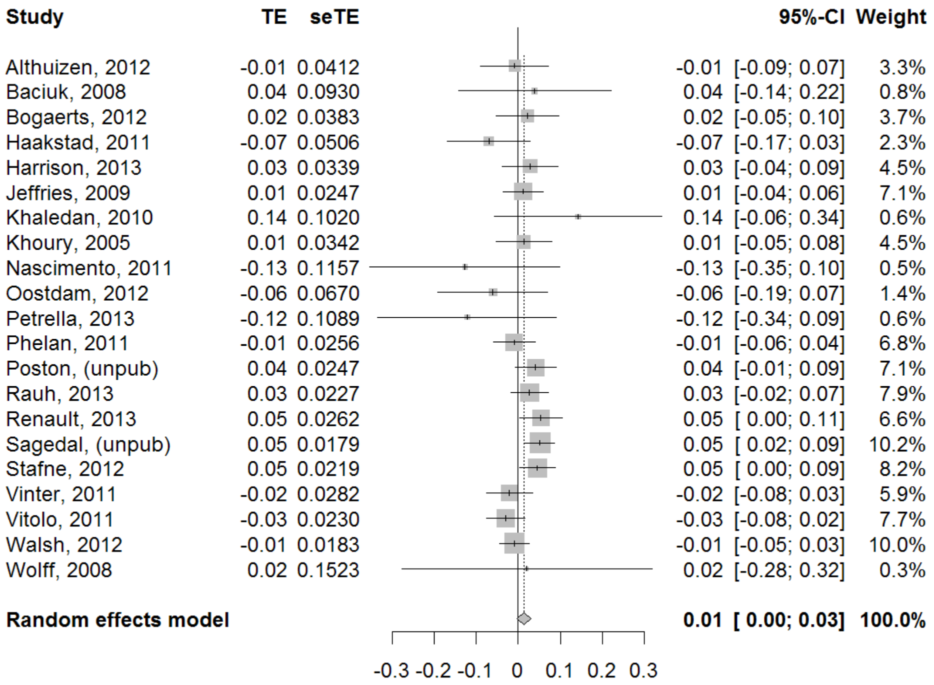

Appendix 1. Identification and selection of studies in individual participant data (IPD) meta-analysis for the i-WIP Database

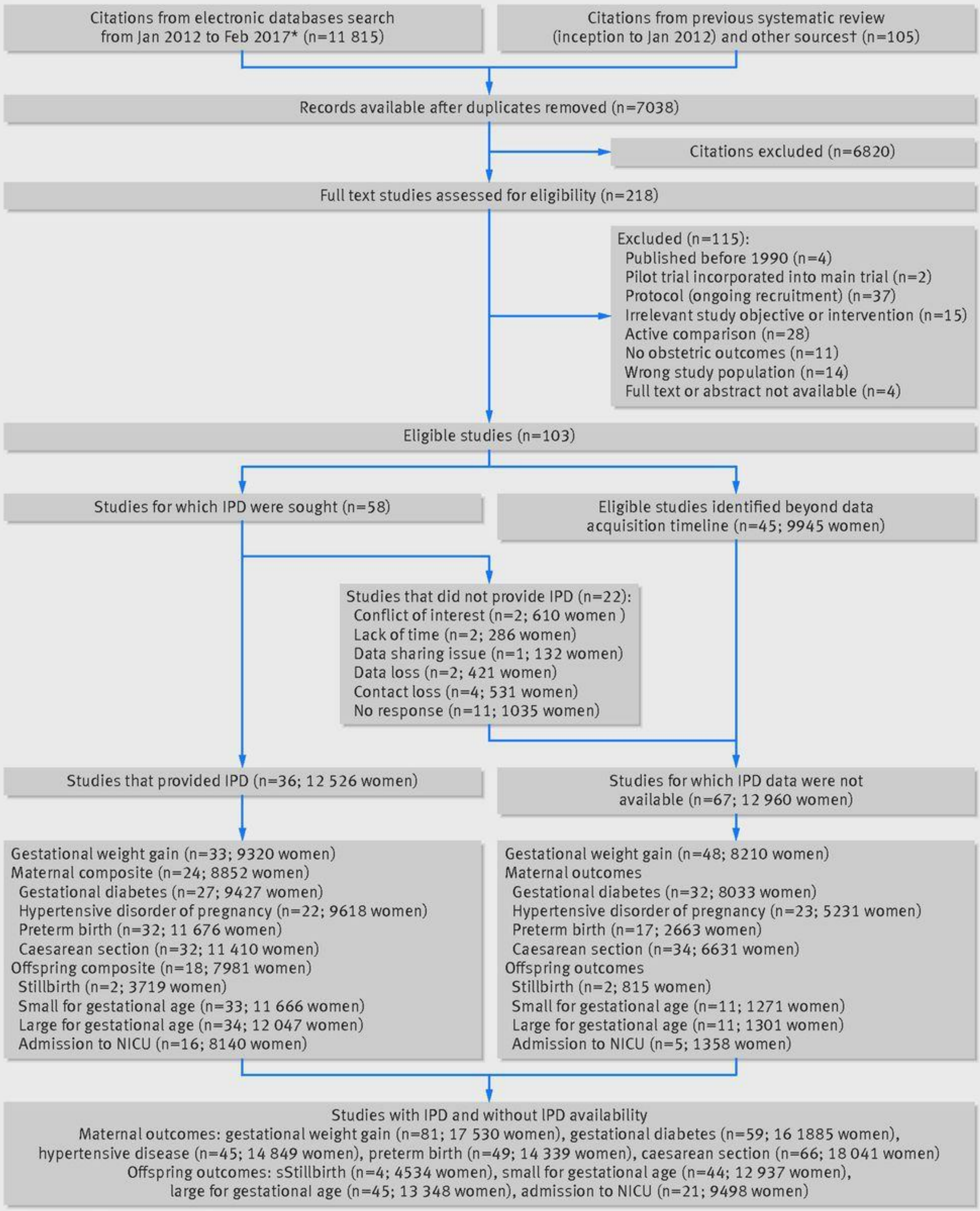

NICU=neonatal intensive care unit
*Database search was updated in October 2013 (9359 records), March 2015 (3551 records), January 2016 (1373 records), and February 2017 (1547 records)
†Other sources: reference search, personal communication, and Google search

Appendix 2. Search strategy for identification of randomised trials on lifestyle interventions in pregnancy and maternal and offspring outcomes (original search strategy from BMJ 2017;358:j3119)

Search strategy for Medline via Ovid

Item	Term
1	Pregnancy/
2	pregnan*.tw.
3	Gravidity/
4	gravid*.tw.
5	gestation*.tw.
6	Pregnant Women/
7	pregnant wom#n.tw.
8	(child adj3 bearing).tw.
9	childbearing.tw.
10	matern*.tw.
11	or/1-10
12	Weight Gain/ph [Physiology]
13	weight gain*.tw.
14	Weight Loss/ph [Physiology]
15	weight loss*.tw.
16	weight change*.tw.
17	Obesity/dh, me, ph, pc, px, th [Diet Therapy, Metabolism, Physiology, Prevention & Control, Psychology, Therapy]
18	obes*.tw.
19	Adiposity/ph [Physiology]
20	adipos*.tw.
21	Overweight/dh, me, ph, pc, px, th [Diet Therapy, Metabolism, Physiology, Prevention & Control, Psychology, Therapy]
22	overweight*.tw.
23	Body Mass Index/
24	bmi.tw.
25	or/12-24
26	exp Randomized Controlled Trial/
27	"randomized controlled trial".pt.
28	"controlled clinical trial".pt.
29	(random$ or placebo$).tw,sh.
30	((singl$ or double$ or triple$ or treble$) and (blind$ or mask$)).tw,sh.
31	single-blind method/
32	double-blind method/
33	or/26-32
34	11 and 25 and 33
35	exp Animals/
36	(rat$ or mouse or mice or hamster$ or animal$ or dog$ or cat$ or bovine or sheep or lamb$).af.
37	35 or 36
38	Humans/
39	human$.tw,ot,kf.
40	37 or 38
41	37 not (37 and 40)
42	34 not 41

Appendix 3. Institute of Medicine Guidelines for gestational weight gain per week in trimester 2 and 3, and extrapolated to trimester 1

Participant level data: A measured, early-pregnancy weight was available for 4,888 participants. Early-pregnancy weight was estimated using self-reported pre-pregnancy weight for the 295 participants missing this variable. Estimated weight gain per week from pre-pregnancy to early-pregnancy was calculated by the following formula, using only participants with both pre- and early-pregnancy weight, n = 3001:

$$\textit{Weight gain from pre- to early-pregnancy} = \frac{\textit{Early-pregnancy weight – pre-pregnancy weight}}{\textit{Baseline GA}}$$

Mean weight gain from pre- to early-pregnancy was 0.175 kg/week. We used the following formula to estimate early-pregnancy weight for the 295 participants missing this variable:

$$\text{Early-pregnancy weight} = (\text{Baseline GA}*0.175\text{ kg}) + \text{pre-pregnancy weight}$$

We used pre-pregnancy weight to categorise participants according to BMI. Self-reported pre-pregnancy weight was available for 3,296 participants, and estimated using early-pregnancy weight for the remaining 1,887 participants. We used the following formula to estimate pre-pregnancy weight:

$$\text{Pre-pregnancy weight} = \text{Early-pregnancy weight} – (\text{Baseline GA}*0.175\text{ kg})$$

Total weight gain was calculated by subtracting early-pregnancy weight from final follow-up weight. Weekly GWG was calculated using the following formula:

$$\textit{Weight Gain per week} = \frac{\textit{Total } GWG}{\textit{Final follow-up GA – Baseline GA}}$$

Trimester 2 and 3 GWG/week was required for comparison with IOM 2009 guidelines. This was calculated using a four-step approach;

1. Trimester 1 GWG/week guidelines were extrapolated from the IOM guidelines(15) .

BMI Category	Total GWG (IOM Guidelines)			Rate of weight gain for Trimester 2 and 3 (IOM Guidelines)		
	**Inadequate**	**Adequate**	**Excess**	**Mean**	**Range**	
**Underweight**	<12.5	12.5 - 18.0	>18.0	0.51	0.44	0.58
**Normal**	<11.5	11.5 - 16.0	>16.0	0.42	0.35	0.50
**Overweight**	<7.0	7.0 - 11.5	>11.5	0.28	0.23	0.33
**Obese**	<5.0	5.0 - 9.0	>9.0	0.22	0.17	0.27

2. The number of weeks in trimester 1 that the participant was enrolled in the intervention was calculated by subtracting the baseline GA from 13. If the participant enrolled in the study at greater than 13 weeks, trimester 1 weeks were set as 0. The number of weeks in trimester 2 and 3 that the participant was enrolled in the intervention were calculated by subtracting the 13 from the final follow-up GA.
3. The rate of weight gain for Trimester 1 was calculated:

(*Rate of weight gain for Trimester 2 and 3*)**27= Total weight gain for Trimester 2 and 3* ***(A)***

Total GWG [ranges for adequate] - Total weight gain for Trimester 2 and 3 [ranges] = Total weight gain for Trimester 1 [ranges] ***(B)***

$$\frac{\textit{Lower range Trimester 1 weight gain} + \textit{Upper range Trimester 1 weight gain}}{2} = \textit{Mean weight gain for Trimester 1 } \mathbf{(C)}$$

$$\frac{\textit{Total weight gain for Trimester 1 [mean and ranges]}}{13} = \textit{Rate of weight gain for Trimester 1 } \mathbf{(D)}$$

BMI Category	(A) Total weight gain for Trimester 2 and 3			Total weight gain for Trimester 1			(D) Rate of weight gain for Trimester 1		
	**Mean**	**Range**		**(C) Mean**	**(B) Range**		**Mean**	**Range**	
**Underweight**	13.77	11.88	15.66	1.48	0.62	2.34	0.11	0.05	0.18
**Normal**	11.34	9.45	13.50	2.28	2.05	2.50	0.18	0.16	0.19
**Overweight**	7.56	6.21	8.91	1.69	0.79	2.59	0.13	0.06	0.20
**Obese**	5.94	4.59	7.29	1.06	0.41	1.71	0.08	0.03	0.13

4. The contribution of trimester 1 weight gain to total GWG was estimated using the mean trimester 1 GWG/week (above) and the following equation:

$$\text{T1 weight gain} = (\text{T1 weight gain per week for BMI}) * (\text{T1 weeks in intervention})$$

5. Trimester 2 and 3 weight gain per week was calculated using the following equation:

$$\textit{T2 and T3 weight gain per week} = \frac{\textit{Total weight gain} - \textit{T1 weight gain}}{\textit{Weeks in T2 and T3}}$$
