# Supplementary material for: Impact of maternal education on response to lifestyle interventions to reduce gestational weight gain: individual participant data meta-analysis
Source: BMJ Open. 2019 Aug 1;9(8):e025620. doi: 10.1136/bmjopen-2018-025620 (PMC6688690; doi:10.1136/bmjopen-2018-025620)
Supplement: Author's manuscript [file bmjopen-2018-025620.draft_revisions.pdf]

BMJ Open

BMJ Open is committed to open peer review. As part of this commitment we make the peer review history of every article we publish publicly available.

When an article is published we post the peer reviewers' comments and the authors' responses online. We also post the versions of the paper that were used during peer review. These are the versions that the peer review comments apply to.

The versions of the paper that follow are the versions that were submitted during the peer review process. They are not the versions of record or the final published versions. They should not be cited or distributed as the published version of this manuscript.

BMJ Open is an open access journal and the full, final, typeset and author-corrected version of record of the manuscript is available on our site with no access controls, subscription charges or pay-per-view fees (http://bmjopen.bmj.com).

If you have any questions on BMJ Open's open peer review process please email editorial.bmjopen@bmj.com

BMJ Open

Impact of maternal education on response to lifestyle interventions to reduce gestational weight gain - Individual participant data meta-analysis

Journal:	*BMJ Open*
Manuscript ID	bmjopen-2018-025620
Article Type:	Research
Date Submitted by the Author:	26-Jul-2018
Complete List of Authors:	O'Brien, Eileen; University College Dublin, UCD Perinatal Research Centre<br>Segurado, Ricardo; University College Dublin, CSTAR, School of Public Health, Physiotherapy and Population Science<br>Geraghty, Aisling; University College Dublin, UCD Perinatal Research Centre<br>Alberdi , Goiuri; University College Dublin, UCD Perinatal Research Centre<br>Rogozinska, Ewelina; Queen Mary's University London, Women's Health Research Unit<br>Astrup, Arne; Univesity of Copenhagen, Department of Nutrition, Exercise and Sports,<br>Barakat Carballo , Rubenomar; Universidad Politecnica de Madrid, Facultad de Ciencias de la Actividad Fısica y del Deporte<br>Bogaerts, Annick; University of Leuven, Department of Development and Regeneration KU Leuven; UC Leuven-Limburg, Faculty of Health and Social Work<br>Cecatti, Jose; University of Campinas, Obstetrics and Gynecology<br>Coomarasamy, Arri; University of Birmingham,<br>de Groot, Christianne; VU University Medical Center, Obstetrics and Gynaecology<br>Devlieger, R.; Universitaire Ziekenhuizen Leuven, Department of Obstetrics and Gynecology; KUL University of Leuven, Department of Development and Regeneration<br>Dodd, Jodie; The Unversity of Adelaide, Obstetrics and Gynaecology; Discipline of Obstetrics and Gynaecology<br>El Beltagy, Nermeen ; Alexandria University, Department of Obstetrics and Gynecology<br>Facchinetti , Fabio ; Azienda Ospedaliera Universitaria Policlinico di Modena, Obstetric Unit, Mother Infant Department<br>Geiker , Nina ; Rigshospitalet, Clinical Nutrition Research<br>Guelfi, Kym; The University of Western Australia, School of Human Sciences<br>Haakstad, Lene; Norges idrettshogskole, Department of Sports Medicine<br>Harrison, Cheryce; Monash University School of Public Health and Preventive Medicine, Monash Centre for Health Research and Implementation<br>Hauner, Hans; Technische Universitat Munchen Zentralinstitut fur Ernahrungs- und Lebensmittelforschung, Else Kroener-Fresenius-Center for Nutritional Medicine<br>Jensen, DM; Syddansk Universitet Det Sundhedsvidenskabelige Fakultet, Department of Endocrinology

Khan, Khalid; Queen Mary, University of London, Multidisciplinary Evidence Synthesis Hub (mEsh); Queen Mary University of London, Women's Health Research Unit
Kinnunen, Tarja; University of Tampere, School of Health Sciences
Luoto, Riitta; Tampereen Yliopisto, Health Sciences
Willem Mol, Ben; University of Adelaide, Obstetrics/Gynaecology
Mørkved, Siv; NTNU Fakultet for ingeniorvitenskap og teknologi Trondheim, Department of Public Health and General Practice; Trondheim University Hospital, Clinical Services
Motahari, Narges ; University of Mazandaran, Department of Sport Physiology
Owens, Julie; The University of Adelaide, Discipline of Obstetrics & Gynaecology
Perales, Maria; Universidad Politecnica de Madrid, Facultad de Ciencias de la Actividad Fısica y del Deporte
Petrella, Elisabetta; Universita degli Studi di Modena e Reggio Emilia Dipartimento di Educazione e Scienze Umane, Mother-Infant Department
Phelan, Suzanne; California Polytechnic State University College of Science and Mathematics, Kinesiology Department
Poston, Lucilla; Kings College, London, Division of Reproduction and Endocrinology
Rauh, Kathrin; Technische Universitat Munchen Zentralinstitut fur Ernahrungs- und Lebensmittelforschung, Else Kroener-Fresenius-Center for Nutritional Medicine; Competence Centre for Nutrition (KErn)
Rayanagoudar, Girish ; Queen Mary University of London, Women's Health Research Unit
Renault , KM ; University of Copenhagen, Departments of Obstetrics and Gynecology
Ruifrok, A; VU medisch centrum ICaR-VU Onderzoeksinstituut, Department of Obstetrics and Gynaecology; Academic Medical Centre, Department of Obstetrics and Gynecology
Sagedal, Linda; Sorlandet sykehus HF, Department of Obstetrics and Gynecology
Salvesen, Kjell; Trondheim University Hospital, Dept of Obstetrics and gynaecology; Norwegian University of Science and Technology, Department of Laboratory Medicine Children's and Women's Health
Scudeller, Tania; Universidade Federal de Sao Paulo, Department of Management and Health Care
Shen, Gary; University of Manitoba College of Medicine, Department of Internal Medicine
Shub, Alexis; University of Melbourne, Obstetrics and Gynaecology
Stafne, Signe; NTNU Fakultet for ingeniorvitenskap og teknologi Trondheim, Department of Public Health and General Practice; Sankt Olavs Hospital Universitetssykehuset i Trondheim, Clinical Services
Surita, Fernanda; University of Campinas, Obstetrics & Gynecology
Thangaratinam, Shakila; Queen Mary University of London, Women's Health Research Unit; Queen Mary University of London, Multidisciplinary Evidence Synthesis Hub (mEsh)
Tonstad, Serena ; Oslo universitetssykehus Ulleval, Department of Obstetrics and Gynecology
van Poppel, Mireille; VU University Medical Center, Department of Public and Occupational Health
Vinter, Christina ; Syddansk Universitet Det Sundhedsvidenskabelige Fakultet, Department of Obstetrics and Gynecology
Vistad, Ingvild ; Sorlandet Hospital Kristiansand , Obstetric and Gynegology
Yeo, SeonAe ; University of North Carolina at Chapel Hill, School of Nursing
McAuliffe, Fionnuala; University College Dublin, UCD Perinatal Research Centre

Keywords: Pregnancy, Lifestyle interventions, Socioeconomic status, Inequalities,

	Nutrition < TROPICAL MEDICINE

SCHOLARONE™
Manuscripts

For peer review only

Title: Impact of maternal education on response to lifestyle interventions to reduce gestational weight gain - Individual participant data meta-analysis

Author List:

E.C. O'Brien [1], R. Segurado [2], A.A. Geraghty [1], G. Alberdi [1], E. Rogozińska [3, 4], A. Astrup [5], R. Barakat Carballo [6], A. Bogaerts [7, 8, 9], J.G. Cecatti [10], A. Coomarasamy [11], C.J. de Groot [12], R. Devlieger [13, 14], J.Dodd [15], N.El Beltagy [16], F. Facchinetti [17], N.R. Geiker [18], K.J Guelfi [19], L. Haakstad [20], C.Harrison [21], H. Hauner [22], D.M. Jensen [23], K.S. Khan [4,26], T. I. Kinnunen [27], R. Luoto [28], B. W. Mol [29], S. Mørkved [30, 31], N. Motahari [32], J. Owens [15], M. Perales, [6], E. Petrella [17], S. Phelan [33], L. Poston [34], K. Rauh [35,36], G. Rayanagoudar [26], K.M. Renault [37, 38], A.E. Ruifrok [12,39], L.R. Sagedal [40], K.Å Salvesen [41,42], T.T. Scudeller [43], G.X. Shen [44], A. Shub [45], S.N. Stafne [30, 31], F. Surita [10], S. Thangaratinam [4,26], S. Tonstad [46], M.N. van Poppel [47], C. Vinter [24], I. Vistad [40], S. Yeo [49], i-WIP (International Weight Management in Pregnancy) Collaborative Group, F.M. McAuliffe [1]*

Affiliations

1 UCD Perinatal Research Centre, Obstetrics & Gynaecology, UCD School of Medicine, University College Dublin, National Maternity Hospital, Dublin, Ireland

2 Centre for Support and Training in Analysis and Research (CSTAR), School of Public Health, Physiotherapy and Sports Science, University College Dublin, Dublin, Ireland

3 Women's Health Research Unit, Barts and The London School of Medicine and Dentistry, Queen Mary University of London, London, UK..

4 Multidisciplinary Evidence Synthesis Hub (mEsh), Barts and The London School of Medicine and Dentistry, Queen Mary University of London, London, UK.

5 Department of Nutrition, Exercise and Sports, Copenhagen University, Copenhagen, Copenhagen, Denmark.

6 Facultad de Ciencias de la Actividad Fısica y del Deporte-INEF, Universidad

For peer review only - http://bmjopen.bmj.com/site/about/guidelines.xhtml

Politecnica de Madrid, Madrid, Spain.

7 Department of Development and Regeneration KU Leuven, University of Leuven, Leuven, Belgium,

8 Faculty of Health and Social Work, research unit Healthy Living, UC Leuven-Limburg, Belgium

9 Faculty of Medicine and Health Sciences, Centre for Research and Innovation in Care (CRIC), University of Antwerp, Belgium.

10 Department of Obstetrics and Gynecology, School of Medical Sciences, University of Campinas (UNICAMP), Campinas, Brazil.

11 School of Clinical and Experimental Medicine, College of Medical and Dental Sciences, University of Birmingham, Birmingham, UK.

12 Department of Obstetrics and Gynaecology, Faculty of Medicine, VU University Medical Center, Amsterdam, The Netherlands.

13 Department of Obstetrics and Gynecology, University Hospitals Leuven, Belgium

14 Department of Development and Regeneration, KUL University of Leuven, Belgium

15 Discipline of Obstetrics and Gynaecology, School of Paediatrics and Reproductive Health, The University of Adelaide, Adelaide, Australia.

16 Department of Obstetrics and Gynecology, Alexandria University, Alexandria, Egypt.

17 Mother-Infant Department, University of Modena and Reggio Emilia, Modena, Italy.

18 Clinical Nutritional Research Unit, Copenhagen University Hospital Herlev-Gentofte, Gentofte, Denmark.

19 School of Human Sciences, The University of Western Australia, Perth, Australia.

20 Norwegian School of Sport Sciences, Department of Sports Medicine, Oslo, Norway.

21 Monash Centre for Health Research and Implementation -MCHRI, School of Public Health Monash University, Melbourne, Australia.

22 Else Kroener-Fresenius-Center for Nutritional Medicine, Klinikum rechts der Isar, Technische Universität München, Munich, Germany.

For peer review only

23 Department of Endocrinology, Odense University Hospital, University of Southern Denmark, Odense, Denmark.

24 Department of Gynecology and Obstetrics, Odense University Hospital, University of Southern Denmark, Odense, Denmark.

25 Department of Clinical Research, Faculty of Health Sciences, University of Southern Denmark

26 Women's Health Research Unit, Barts and The London School of Medicine and Dentistry, Queen Mary University of London, London, UK.

27 Faculty of Social Sciences/ Health Sciences, University of Tampere, Tampere, Finland.

28 School of Health Sciences, University of Tampere, Tampere, Finland.

29 Robinson Institute, School of Paediatrics and Reproductive Health, University of Adelaide, Adelaide, Australia.

30 Department of Public Health and General Practice, Faculty of Medicine, Norwegian University of Science and Technology, Trondheim, Norway.

31 Clinical Services, St. Olavs Hospital, Trondheim University Hospital, Trondheim, Norway.

32 Department of Sport Physiology, Faculty of Physical Education and Sport Sciences, Mazandaran University, Babolsar, Iran.

33 Kinesiology Department, California Polytechnic State University, San Luis Obispo, USA.

34 Division of Women's Health, Women's Health Academic Centre, King's College London, St. Thomas' Hospital, London, UK.

35 Else Kroener-Fresenius-Center for Nutritional Medicine, Chair of Nutritional Medicine, Technische Universität München, Munich, Germany.

36 Competence Centre for Nutrition (KErn), Freising, Germany.

37 Departments of Obstetrics and Gynecology, Hvidovre Hospital, University of

For peer review only

Copenhagen, Copenhagen, Denmark.

38 Obstetric Clinic, Juliane Marie Centret, Rigshospitalet, University of Copenhagen, Copenhagen, Denmark.

39 Department of Obstetrics and Gynecology, Academic Medical Centre, Amsterdam, The Netherlands.

40 Department of Obstetrics and Gynecology, Sorlandet Hospital, Kristiansand, Norway.

41 Department of Obstetrics and Gynaecology, St. Olavs Hospital, Trondheim University Hospital, Trondheim, Norway

42 Department of Laboratory Medicine Children's and Women's Health, Faculty of Medicine, Norwegian University of Science and Technology, Trondheim, Norway.

43 Department of Management and Health Care, Physical Therapy Course, Federal University of São Paulo (UNIFESP), Brazil

44 Department of Internal Medicine, University of Manitoba, Winnipeg, Canada.

45 Department of Obstetrics and Gynaecology, University of Melbourne, Melbourne, Australia.

46 Department of Obstetrics and Gynecology, Oslo University Hospital, Oslo, Norway.

47 Department of Public and Occupational Health, Amsterdam Public Health research institute, VU University Medical Center, Amsterdam, The Netherlands.

Institute of Sport Science, University of Graz, Austria.

48 University of North Carolina at Chapel Hill, School of Nursing, Chapel Hill, NC, USA.

Corresponding author:

Fionnuala M McAuliffe; Email: fionnuala.mcauliffe@ucd.ie

Key Words: Pregnancy; Lifestyle interventions; socioeconomic status; inequalities; nutrition

Word count: 3,737

For peer review only - http://bmjopen.bmj.com/site/about/guidelines.xhtml

Abstract

Objectives: To identify if socioeconomic status is a prognostic factor for gestational weight gain (GWG), and to determine the differential effects of lifestyle interventions (diet-alone, exercise-alone or mixed approach) on gestational weight gain, according to socioeconomic status.

Design: Individual participant data meta-analysis. The PRISMA-IPD Statement guidelines were followed.

Setting: Data from the International Weight Management in Pregnancy (i-WIP) Collaborative Group (randomised controlled trials from 11 countries included).

Participants: 21 randomised controlled trials and 5,183 pregnant women. Maternal educational attainment was used as a proxy for socioeconomic status and women were categorised as having higher education (≥ tertiary) or lower education (≤ secondary).

Interventions: Dietary, physical activity and mixed lifestyle interventions.

Primary Outcome Measure: GWG analysed as: (a) inadequate, adequate, and excess per the Institute of Medicine (2009) guidelines, and (b) as kg/week.

Results: Women of lower educational attainment had increased risk of excessive (OR:1.182; 95% CI:1.008-1.385; P:0.039) and inadequate weight gain (OR:1.284; 95% CI:1.045-1.577; *P*: 0.017). Among women with lower education, diet-alone interventions reduced risk of excessive weight gain (OR:0.515; 95% CI:0.339-0.785; *P*:0.002) and inadequate weight gain (OR:0.504; 95% CI:0.288-0.884; *P*:0.017), and reduced kg/week gain (*B:*-0.055; 95% CI:-0.098–-0.012; *P*:0.012). Mixed interventions reduced risk of excessive weight gain for women with lower education (OR:0.735; 95% CI:0.561-0.963; *P*:0.026). Among women with high education, diet-alone interventions reduced risk of excessive weight gain (OR:0.609; 95% CI:0.437-0.849; *P*:0.003), and mixed interventions reduced kg/week gain (*B:*-0.053; 95% CI:-0.069–-0.037*;* *P*:<0.001). Exercise-alone interventions did not impact GWG in the total cohort, nor stratified by education.

For peer review only - http://bmjopen.bmj.com/site/about/guidelines.xhtml

Conclusions: Pregnant women with lower education are at an increased risk of excessive and inadequate GWG, highlighting the need for targeted intervention in this vulnerable population. Diet-alone interventions seem the most appropriate choice for these women, and additional support through mixed interventions may also be beneficial.

Article Summary

Strengths and limitations of this study

- A major strength is the use of individual patient data to carry out meta-analysis from multiple nutrition and lifestyle interventions.
- The pooling of individual patient data allows for the estimation of the differential effects of education and lifestyle interventions on weight gain across the included cohorts with greater power than individual randomised controlled trials.
- We analysed and reported the findings using gestational weight gain per week, which enables studies that use different gestational ages for measuring gestational weight gain to be compared.
- A limitation is that most of the cohorts included are from wealthy countries; therefore, the results may not be representative of populations living in low and middle-income countries.
- Although the results were statistically significant, the effect sizes are small, thus the relevance of these findings in the clinical setting must be considered.

For peer review only

For peer review only - http://bmjopen.bmj.com/site/about/guidelines.xhtml

Introduction

According to the 2030 Sustainable Development Goals, the global health agenda is calling for improvements in maternal health and nutrition, equal opportunities for education, and reduced social, economic and political inequalities (1). Currently, however, maternal social, economic and educational backgrounds remain to be important determinants of health and lifestyle behaviours among pregnant women. Pregnancy and birth outcomes are negatively influenced by socioeconomic and deprivation factors (2–6). Prevalence of smoking, alcohol consumption and pre-pregnancy body mass index (BMI) are greater among women of lower socioeconomic status, while diet quality and physical activity participation are reduced within this group (7–9).

GWG is also influenced by socioeconomic status, with a recent systematic review finding that those of lower educational attainment are less likely to gain weight within the guidelines' recommended ranges for GWG, as described by the Institute of Medicine (IOM) (10). Excessive GWG increases the risk of adverse maternal and neonatal outcomes, including gestational diabetes, caesarean section, hypertension, large-for-gestational-age, and stillbirth, while inadequate GWG is associated with increased risk of small-for-gestational-age infant (11,12). Moreover, those who gain excessively are at an increased risk of weight retention and elevated BMI up to 15 years post-pregnancy (13), a potential factor exacerbating current global trends of overweight and obesity (14).

Since GWG outside of suggested guidelines is more prevalent among disadvantaged groups, it would seem wise that support and interventions that enable equal opportunities to optimise health are prioritised. However, there remains limited knowledge regarding GWG among women of various socioeconomic position, as recognised by the IOM (15). Pregnancy has been described as a stimulus for positive behaviour change (16) and is a time when women are in regular contact with healthcare professionals (17). Thus, a lifestyle intervention in pregnancy should, in theory, have a greater impact during this time than any other time in a woman's life.

For peer review only - http://bmjopen.bmj.com/site/about/guidelines.xhtml

There are many examples of lifestyle and behaviour interventions in the literature that aim to manage weight gain during pregnancy (18); however few interventions stratify analyses by socioeconomic status. The International Weight Management in Pregnancy (i-WIP) Individual Patient Data Collaborative Network was established to determine the differential effects of weight management interventions in pregnancy on maternal weight gain and pregnancy outcomes (19), and found that diet and physical activity based interventions reduce GWG and lower the odds of caesarean section (20). However, sub-analysis was not conducted among women of various socioeconomic statuses, thus compounding our lack of understanding for the type of pregnancy intervention that may potentially have the greatest effect on those of low socioeconomic status.

This research will conduct IPD meta-analysis using the i-WIP dataset to: (1) identify if socioeconomic status is a prognostic factor for GWG; (2) determine the differential effects of weight management interventions in pregnancy on maternal weight gain according to socioeconomic status.

Methods

The checklist outlined in the Preferred Reporting Items for Systematic reviews and Meta-Analysis of Individual Participant Data (PRISMA-IPD) statement was followed. Full information about the search strategy is available in the i-WIP protocol paper and the i-WIP primary publication (19,20). In brief, major electronic databases from Oct 2013 to Mar 2015 were searched, with an updated search completed in Feb 2017. Eligible studies were randomised controlled trials (RCT) that evaluated any dietary or lifestyle interventions with potential to influence maternal and fetal outcomes related to maternal weight (19,21). The women in the study were required to be ≥18 years of age with a singleton pregnancy and they must have been enrolled to one of the following interventions; diet based, physical activity based, mixed approach (diet, physical activity, GWG monitoring and/or behaviour change components), or standard care (21). In total, 11,920 studies were identified, of

For peer review only - http://bmjopen.bmj.com/site/about/guidelines.xhtml

which 36 studies were included (12,526 participants) (19,20,22). The flow-chart for the identification and selection of studies has been published (20) (Appendix 1).

IPD integrity and risk of bias assessment

The i-WIP Network performed a range checks on the variables used during the analysis and produced summary tables, with a focus on checking the randomisation ratio, baseline characteristics and the method of analysis in the IPD data set with the published information. The i-WIP Network discussed major discrepancies with the trial team. As per the protocol, the i-WIP Network assessed the quality of each trial to evaluate the integrity of the randomisation and follow-up procedure. The Cochrane risk of bias tool was used and studies were classified as high risk of bias if they scored highly in at least one of following domains: randomisation, allocation concealment, blinding of outcome assessment, or incomplete outcome data. To be classified as low risk of bias, all items must have scored as low risk.

Data items and specification of outcomes

The i-WIP protocol pre-specified that socioeconomic status would be evaluated as a potential prognostic variable for GWG, as secondary analysis to the i-WIP project (19). The exposure variable was maternal educational attainment, as this was the most commonly used measure of socioeconomic status among studies in the i-WIP network. It has also been reported as a stable measure of individual socioeconomic status (23). Educational attainment was classified as less than or equal to secondary education (including no education, less than primary, completed primary, some secondary, or completed secondary education), or tertiary education (including vocational training, some university, bachelor degree, post-graduate degree or higher).

Intervention type was classified as diet based, physical activity based, or mixed approach (diet, physical activity, GWG monitoring and/or behaviour change components) (21), and participants were either exposed to the intervention, or to standard care.

For peer review only - http://bmjopen.bmj.com/site/about/guidelines.xhtml

The primary outcome was maternal weight gain in pregnancy. The outcome was examined using two methods: according to the IOM 2009 guidelines (15); and GWG per week as a continuous variable. To calculate GWG, data were required for the following variables; baseline gestational age, final follow-up gestational age, pre-pregnancy or early-pregnancy weight, final follow-up weight and height. The method described in Appendix 2 was used to calculate GWG per week.

Specification of effect measures

The principle measures of effect used were odds ratio (for analysis according to the IOM guidelines), and regression coefficient (for analysis of GWG as a continuous variable).

To assess the magnitude of study variability in the outcomes, we calculated an intra-class correlation coefficient (ICC) from the fitted models, interpretable as an index of heterogeneity such as the I^2. The intra-class correlation coefficient for the study (ICC_{study}) relates to the proportion of variability due to cohort-to-cohort variation, and the intra-class correlation coefficient for the intervention ($ICC_{intervention}$) relates to the proportion of variability in the intervention treatment effect. In the logistic models for categorical GWG the residual variance was replaced by $\pi^2/3$.

Synthesis methods

For comparisons of characteristics across education groups, we used independent sample *t* tests for continuous data and χ^2 tests for categorical data. For the categorical outcomes of inadequate, adequate, and excessive GWG, we used generalised linear mixed models: a multinomial logistic regression with a generalised logit link function. For the continuous outcomes of GWG (kg/week), we used a linear mixed model with an identity link function, assuming the outcome was approximately normally distributed. The models analysing the effect of education included random effects for each cohort. Random effects were specified for both the intercept and the slope of the intervention effect, allowing them to be correlated. The between-study variance in each effect was calculated by the ICC. Separate models

For peer review only

were created for educational attainment strata to analyse the effect of each intervention type, as fixed effects. Linear mixed models were adjusted for factors known to influence GWG (BMI, maternal age, parity, ethnicity), in addition to the main effects and interaction of education and the interventions. In model 1 we adjusted for pre-pregnancy maternal BMI (continuous GWG model only). In model 2 we adjusted for pre-pregnancy maternal BMI (continuous GWG model only), maternal age, parity and ethnicity. As the IOM category definitions are linked to pre-pregnancy BMI category, this outcome intrinsically “adjusts” for maternal BMI; we can consider model 1 as “unadjusted”, and model 2 as an “adjusted” model. All statistical analyses were performed using IBM SPSS Statistics for Windows, Version 20.0 (IBM, Armonk, NY).

Exploration of variation in effects

While controlling for between-study variation in outcomes and intervention effects as random effects, we explored the influence of educational attainment on the intervention effects as pre-specified fixed main effects and an interaction term. Tertiary education and the control (non-intervention) condition were set as the reference levels, and results for IOM categories used the adequate weight gain category as the reference outcome group.

Additional analyses

We proceeded with our model-building in a step-wise fashion, aiming at a pre-specified target of fixed effects as above, and both random intercept and intervention effects. A step-wise approach is often pragmatic in developing a mixed-effects model as small study size and uncertain variance/covariance structure can lead to models failing to converge. We therefore combined the addition of random and fixed effects to a model in the following stages: 1) empty model including no fixed predictors, only a random intercept; 2) education and intervention main effects and interaction added to the model, and controlling for pre-pregnancy maternal BMI (Model 1); 3) Random effect of intervention was explored, to account for potential population or intrinsic variability in the effect of the interventions on

For peer review only

GWG; 4) additional potential confounders were added as individual-level fixed effects to either model 2 or 3: maternal age, parity, ethnicity (Model 2). Our strategy was to remove fixed interaction effects or random effects if not statistically significant, or if model fitting became problematic. Furthermore, within each study, independent sample *t*-tests were carried out to explore the differences in weight gain per week by education level.

Patient involvement

No patients were involved in the design of this study, however we plan to share and disseminate the findings with the ROLO Families Advisory Committee and the ROLO participants via online social media and quarterly newsletters.

Results

Protocol, IPD obtained and Study characteristics

The PRISMA-IPD checklist is included in the additional files. The original i-WIP IPD dataset contained 12,240 participants from 36 studies. Participants were excluded if they were missing data relating to the main outcomes or met the exclusion criteria (Table 1).

In total, 7,057 women were excluded from the analysis, while 5,183 remained. Participants that met the inclusion criteria were enrolled to 21 RCTs (24–44). The study characteristics, methods used to measure GWG, intervention details and outcome measures of the eligible trials are summarised in Table S1.

IPD integrity and Risk of bias assessment

No issues were identified in the integrity of the IPD. The global classification of risk of bias classified 12 studies as high risk and 9 as low/medium risk (Table S2).

Results of individual studies

For the majority of studies, we did not observe a significant difference in GWG/week (kg) between participants in the intervention and control, split by educational attainment (Table

For peer review only - http://bmjopen.bmj.com/site/about/guidelines.xhtml

S3). Within four of the studies (26,32,39,43), women with tertiary education enrolled to the intervention had significantly less weight gain/week. One study found that women with secondary education or less enrolled to the intervention had significantly less weight gain/week (44).

Results of syntheses

Of the 5,183 participants in this analysis, 3,273 (63.1%) had achieved at least some tertiary education, while 1,910 (36.9%) had, at most, completed secondary education. Within the secondary education group, 931 (48.8%) were enrolled to the control arm, and 653 (34.2%), 186 (9.7%) and 140 (7.3%) were enrolled to the intervention arm of mixed, diet, and exercise trials, respectively. Within the tertiary education group, 1,531 (46.8%) were enrolled to the control arm of their respective RCT, while 1,023 (31.2%), 323 (9.9%) and 396 (12.1%) were enrolled to the intervention arm of mixed, diet and exercise trials, respectively.

Compared to women with tertiary education, women with lower education levels had significantly higher early-pregnancy weight and pre-pregnancy BMI, and were significantly shorter in height (Table 2). Fewer women with tertiary education had a BMI >30 kg/m^2, compared to women in the secondary education group (26.8% *vs.* 46.3%, P <0.001). Women within the tertiary education group were older, had lower parity and were more likely to be of Caucasian ethnicity compared to those with lower education.

Mean ± SD GWG per week and percentage of participants within each of the IOM categories, according to educational attainment and BMI category are shown in Table 3. Figure 1 displays GWG (kg/week), according to pre-pregnancy BMI, intervention and educational attainment.

The unadjusted linear mixed-models found that women with lower educational attainment gained significantly more weight per week (kg) compared to those with tertiary education (B = 0.014), however this association was not significant when the model was adjusted for maternal age, parity and ethnicity (Table 4a). The ICC_{study} was 0.1% and $ICC_{intervention}$ was 0.0%. Women of lower educational attainment had an increased risk of both excessive (OR:

For peer review only - http://bmjopen.bmj.com/site/about/guidelines.xhtml

1.182) and inadequate (OR: 1.284) GWG as per the IOM guidelines, controlling for intervention, maternal age, parity, ethnicity, with random effects (Table 5b). The ICC calculations for model 2 of Table 4 (b) were low; excessive weight gain: ICC_{study} 8.1% and $ICC_{intervention}$ 0.4%; inadequate weight gain: ICC_{study} 5.0% and $ICC_{intervention}$ 0.7%.

The intervention-by-education interaction effects were not statistically significant, and for ease of interpretation, these were removed, thus we present results for models stratified by education.

Among those of lower educational attainment, a significant reduction in weight gain per week (kg) was observed for both diet-alone interventions (B = -0.055) and mixed interventions (B = -0.044), compared to those in the control arms, Table 5 (a). No significant effect was observed for exercise-alone interventions. Women with tertiary education enrolled to mixed interventions gained significantly less GWG per week (kg) (B = -0.053), than those in the control arms. Neither diet-alone nor exercise-alone interventions had significant effects on weight gain per week (kg) for women with tertiary education. The adjusted models (Model 2) included BMI, maternal age, parity and ethnicity as confounders, with fixed effects.

Women with lower education were significantly less likely to exceed the IOM guidelines for weight gain per week if they were enrolled to diet-alone interventions (OR: 0.515) or mixed intervention (OR: 0.735), compared to those in the control arms, Table 5 (b). These women were also less likely to gain inadequate weight per week if they were enrolled to a diet-alone intervention (OR: 0.504). Women with tertiary education enrolled to diet-alone interventions were less likely to exceed IOM guidelines (OR: 0.609, however, those enrolled to mixed interventions were more likely to gain inadequate weight gain per week (OR: 1.411), compared to those in the control arms. No effect was observed for exercise-alone interventions. The adjusted models (Model 2) included maternal age, parity and ethnicity as confounders, with fixed effects.

For peer review only - http://bmjopen.bmj.com/site/about/guidelines.xhtml

Discussion

Summary of evidence

This IPD meta-analysis of the i-WIP consortium of lifestyle interventions in pregnancy found that low maternal education is associated with increased risk of both excessive and inadequate GWG per week, although crude differences were not observed in the percentage of women who gained inadequately, adequately and excessively between women of high and low educational attainment. Diet-alone interventions reduced the risk of excessive GWG for all women, and reduced the risk of inadequate GWG for those with lower educational attainment. Mixed interventions were protective against excessive GWG for women with low education, but increased the risk of inadequate GWG among women with higher educational attainment. Exercise-alone interventions were not associated with GWG, regardless of maternal education status.

Compared to the literature

Maternal educational attainment, less than or equal to secondary level, was associated with an 18% increased risk of excessive GWG and a 28% increased risk of inadequate GWG per week, as per the IOM 2009 guidelines (15), compared to those with at least some tertiary education. A recent systematic review of observational studies also found that low maternal educational attainment is associated with women gaining outside the 2009 IOM recommendations for GWG (10). However, the review only provided a narrative of the evidence to date, not a meta-analysis, thus was restricted from drawing firm conclusions. Nonetheless, both the systematic review and our findings highlight the need for additional support for pregnant women of low educational attainment. Although the increased risk of inadequate and excessive GWG among women with lower education was statistically significant, this is not synonymous with clinical significance. Between women of low and high educational attainment, the actual percentage differences in those who gained inadequately (19.0% vs. 14.5%), adequately (21.0% vs. 25.1%) and excessively (60.0% vs. 60.4%) was relatively small.

For peer review only - http://bmjopen.bmj.com/site/about/guidelines.xhtml

The impact of interventions in pregnancy on weight gain has been previously examined (18,20,21,45), however, analyses were not stratified by socioeconomic status within these publications, thus our study provides new insights in this field. A 2015 Cochrane review compared the effect of interventions in pregnancy (diet or exercise, or both) on GWG. Unlike our findings, in which only diet and mixed interventions reduced excessive weight gain, all categories of interventions significantly reduced excessive weight gain compared to control; diet (RR: 0.77), diet and exercise (RR: 0.86), and diet counselling/other (RR: 0.46) (45). However, the review also highlighted an increased risk of low weight gain with all types of interventions; diet (RR: 1.24), diet and exercise (RR: 1.25), and diet counselling/other (RR: 1.21) (45). A systematic review by Thangaratinam *et al.* found that dietary interventions reduced total GWG by 3.84 kg, while mixed and physical activity interventions reduced total GWG to lesser extents (–1.06 kg and –0.72 kg, respectively) (21). Both mixed and physical activity interventions reduced risk of excessive weight gain, but insufficient data were available to assess the impact of dietary interventions on excessive weight gain (21). The i-WIP primary publication in 2017 found that diet and physical activity based interventions during pregnancy reduce GWG (−0.72 kg and −0.73 kg, respectively), but did not find differences in GWG across the subgroups examined (educational attainment was not assessed) (20).

In summary, based on our findings and previous studies, it seems that among women of high and low educational status, dietary interventions are most effective in reducing GWG. Mixed interventions could also be considered for those with lower education. Our study is a novel in that it is the first IPD meta-analysis, using a one-step approach for statistical analyses, in which data from individual pregnant women were used to estimate the effect of varying types of lifestyle interventions on GWG of women from different educational backgrounds.

Clinical relevance of our findings

Dietary interventions have the potential be used as a baseline intervention, offered to all pregnant women, to encourage appropriate weight gain. Women with secondary education or less could be

For peer review only - http://bmjopen.bmj.com/site/about/guidelines.xhtml

offered additional behaviour change counselling or physical activity advice, to complement the dietary intervention.

The success of dietary interventions over other lifestyle interventions in reducing GWG may be attributed to many factors. The importance of a woman's diet in pregnancy has been documented for centuries (46), thus, dietary changes are perhaps more acceptable than changes in physical activity behaviours. Pregnancy itself is an incentive for positive behaviour changes in dietary choices, but less so for physical activity (16). Misconceptions regarding exercise during pregnancy remain, with many perceiving it to pose a safety risk for their baby (47), and believing pregnancy is a time for rest (48). In addition, women may experience feelings of tiredness, nausea and physical discomfort, as well as a time commitments that prevent them from exercising (47–49).

Mixed approach interventions increased the risk of inadequate weight gain among highly educated women. A potential hypothesis is that these women may already be aware of healthy lifestyle strategies, have a higher locus of control (50) and future salience (51), and therefore may be more willingly take actions for reducing weight gain. Consequently, providing multiple approaches to reduce GWG could have a paradoxical effect. However, since up to 80% of highly educated women in this meta-analysis exceeded weight gain guidelines, the focus of healthcare professionals should be on meeting adequate GWG, rather than exerting excess caution in avoiding mixed approach interventions. It is reasonable to suggest that the dietary approach would be sufficient among those with tertiary education in reducing excessive GWG.

Strengths and limitations

The pooling of individual patient data allows for the estimation of the differential effects of education and lifestyle interventions on weight gain across the included cohorts with greater power than individual RCTs (19). IPD meta-analysis does not rely on aggregate data extracted from RCTs, and allows for models to be adjusted for confounders (52). The data are also strengthened by the relatively low variability between studies. In addition, we analysed and reported findings using GWG/week, which enables studies that use different gestational ages for measuring GWG to be compared.

For peer review only - http://bmjopen.bmj.com/site/about/guidelines.xhtml

We acknowledge that a limitation of our work is the lack of pre-pregnancy weight measurements available, a methodological issue that affects much GWG research (53). We classified BMI using self-reported pre-pregnancy weight, which may be subject to underreporting and hence under-classification of BMI (53). However, using the first trimester weight to determine pre-pregnancy BMI would also have been problematic due to weight gain in trimester 1 and potential for over-classification of BMI.(53). Although it has been suggested that pre-pregnancy weight can be somewhat accurately estimated using the first trimester weight, by 12 weeks' gestation weight gain can result in up to 9% of women being misclassified for BMI(54). This was not a viable option since some the first weight measurements were taken as late as 18 weeks. Early-pregnancy weight was estimated using self-reported pre-pregnancy weight for a small number of participants (n=295, 5.7%).

Although limitations of the IOM guidelines have been expressed (55), they are the most widely used recommendations, and are comparable to Intergrowth 21st Project weight gain centiles for women of normal BMI (56). We also used a novel method of extrapolating the IOM expected weight gain per week in trimester 1, and this enabled calculation of trimester 2 and 3 weight gain per week. Lastly, it must be stated that although the results were statistically significant, the effect sizes were small, thus the relevance of our findings in the clinical setting must be considered.

Conclusions

In summary, pregnant women with lower educational attainment are at an increased risk of both excessive and inadequate weight gain, highlighting the need for intervention among this vulnerable population of women. Given that dietary interventions do not widen health inequalities, are successful in promoting appropriate weight gain, and are acceptable; healthcare professionals should consider implementing nutrition based interventions as part of baseline maternity care packages, for all pregnant women.

For peer review only - http://bmjopen.bmj.com/site/about/guidelines.xhtml

Tables

Table 1. Participant Exclusion Criteria for Individual Participant Data

Reason for Exclusion	Excluded N	Remaining N
Full dataset		12240
Data missing: Baseline gestational age	2611	9629
Data missing: Final follow-up gestational age	2309	7320
Data missing: Maternal educational attainment	1878	5442
Multiple pregnancy	17	5425
Less than 18 years of age	75	5350
Data missing: Pre- and early-pregnancy weight	1	5349
Data missing: Final follow-up weight	137	5212
Data missing: Height (for pre-pregnancy BMI)	29	5183

For peer review only - http://bmjopen.bmj.com/site/about/guidelines.xhtml

Table 2. Participant characteristics in the total group, and split by education category

	Total		Lower Education		Higher Education		*P**
	N		N		N		
**Early pregnancy weight (kg)** [a]	5183	78.60 (18.56)	1910	82.51 (20.26)	3273	76.31 (17.08)	<0.001
**Pre-pregnancy weight (kg)** [a]	5183	76.06 (18.74)	1712	79.61 (20.37)	3176	73.17 (16.96)	<0.001
**Height (cm)** [a]	5183	166.07 (7.03)	1910	164.76 (7.17)	3273	166.84 (6.84)	<0.001
**Pre-pregnancy BMI** (kg/m^2) [a]	5183	27.57 (6.55)	1910	29.45 (7.08)	3273	26.47 (5.95)	<0.001
Underweight [b]	5183	77 (1.5)	1910	32 (1.6))	3273	46 (1.4)	<0.001
Normal-weight [b]		2274 (43.9)		603 (31.5)		1672 (51.1)	
Overweight [b]		1073 (20.7)		393 (20.5)		681 (20.8)	
Obese [b]		1763 (34.0)		886 (46.3)		878 (26.8)	
**Gestational age** (weeks)							
**Baseline** [c]	5183	14.5 (4.0 - 32.0)	1910	13.9 (4.0 - 32.0)	3273	14.8 (4.0 - 32.0)	<0.001
**Final follow-up** [c]	5183	36.6 (24.0 - 44.0)	1910	36.6 (27.0 - 44.0)	3273	36.5 (24 - 43.0)	0.196
**Delivery** [c]	5172	39.7 (26.0 - 44.0)	1907	39.50 (27.0 - 44.0)	3265	39.75 (26.0 - 43.0)	<0.001
**Infant birth weight** (g) [a]	5164	3531.73 (547.90)	1908	3476.55 (559.43)	3256	3564.06 (538.49)	<0.001
**Maternal age** (years) [a]	5183	29.90 (4.97)	1910	28.50 (5.51)	3273	30.72 (4.43)	<0.001
**Parity** [a]	5135	0.73 (1.05)	1884	0.93 (1.20)	3251	0.62 (0.93)	<0.001
**Ethnicity**							
Caucasian [b]	3719	3291 (63.5)	1203	954 (49.9)	2516	2338 (71.4)	<0.001
Asian [b]		84 (1.6)		25 (1.3)		60 (1.8)	
Black [b]		123 (2.4)		71 (3.7)		53 (1.6)	
Central/South American [b]		88 (1.7)		76 (3.9)		13 (.4)	
Middle East (including Iran & Turkey) [b]		63 (1.2)		45 (2.3)		19 (.5)	
Other [b]		76 (1.4)		38 (1.9)		39 (1.2)	
Missing [b]		1465 (28.2)		708 (37.0)		758 (23.1)	

Data are presented as [a] mean (SD) [b] n (%) and [c] mean (range of minimum and maximum).
**P*-value: ANOVA and chi-square
BMI: body mass index. Underweight: <18.5 kg/m^2; Normal:18.5 - 24.9 kg/m^2; Overweight: 25.0 - 29.9 kg/m^2; Obese: ≥30 kg/m^2
Lower education: secondary education or less. Higher education: at least some tertiary education

For peer review only - http://bmjopen.bmj.com/site/about/guidelines.xhtml

Table 3. Gestational Weight Gain per Week (kg/week), and per IOM Guidelines, BMI Category and Education

	BMI Category	N	GWG/week (kg) Total GWG/week Mean (SD)	Trimester 2 and 3 GWG/week (kg), and per IOM Guidelines T2 & 3 GWG/week Mean (SD)	Inadequate, n (%)	Adequate, n (%)	Excess, n (%)
All Participants	Total	5183	0.45 (0.22)	0.46 (0.22)	836 (16.1%)	1224 (23.6%)	3123 (60.3%)
	Underweight	76	0.50 (0.16)	0.51 (0.16)	23 (30.3%)	34 (44.7%)	19 (25.0%)
	Normal-weight	2273	0.51 (0.28)	0.51 (0.18)	347 (15.3%)	778 (34.2%)	1148 (50.5%)
	Overweight	1072	0.48 (0.22)	0.49 (0.22)	113 (10.5%)	128 (11.9%)	831 (77.5%)
	Obese	1762	0.35 (0.23)	0.36 (0.24)	353 (20.0%)	284 (16.1%)	1125 (63.8%)
Lower Education	Total	1910	0.42 (0.24)	0.43 (0.25)	363 (19.0%)	401 (21.0%)	1146 (60.0%)
	Underweight	31	0.55 (0.20)	0.56 (0.20)	7 (22.6%)	11 (35.5%)	13 (41.9%)
	Normal-weight	602	0.51 (0.20)	0.52 (0.21)	108 (17.9%)	184 (30.6%)	310 (51.5%)
	Overweight	392	0.46 (0.24)	0.47 (0.24)	55 (14.0%)	59 (15.1%)	278 (70.9%)
	Obese	885	0.34 (0.24)	0.35 (0.25)	193 (21.8%)	147 (16.6%)	545 (61.6%)
Higher Education	Total	3273	0.46 (0.20)	0.47 (0.21)	473 (14.5%)	823 (25.1%)	1977 (60.4%)
	Underweight	45	0.46 (0.10)	0.47 (0.11)	16 (35.6%)	23 (51.1%)	6 (13.3%)
	Normal-weight	1671	0.50 (0.17)	0.51 (0.17)	239 (14.3%)	594 (35.5%)	838 (50.1%)
	Overweight	680	0.49 (0.20)	0.50 (0.20)	58 (8.5%)	69 (10.1%)	553 (81.3%)
	Obese	877	0.35 (0.23)	0.37 (0.24)	160 (18.2%)	137 (15.6%)	580 (66.1%)

GWG: gestational weight gain
BMI: body mass index. Underweight: <18.5 kg/m^2; Normal:18.5 - 24.9 kg/m^2; Overweight: 25.0 - 29.9 kg/m^2; Obese: ≥30 kg/m^2
Lower education: secondary education or less. Higher education: at least some tertiary education

For peer review only

For peer review only - http://bmjopen.bmj.com/site/about/guidelines.xhtml

Table 4. Generalised Linear Mixed-Models to show relationship between maternal educational attainment and (a) gestational weight gain per week (kg), and (b) gestational weight gain per week as per the IOM guidelines

		(a) Gestational Weight Gain per Week (kg/week)			(b) Gestational Weight Gain per Week as per the IOM Guidelines					
					Excessive Weight Gain			Inadequate Weight Gain		
	**Education**	***B***	**95% CI**	***P****	**OR**	**95% CI**	***P****	**OR**	**95% CI**	***P****
**MODEL 1 – (n = 5183) †**	Lower	0.014	(0.001 - 0.027)	0.030	1.224	(1.052 - 1.425)	0.009	1.259	(1.033 - 1.535)	0.022
	Higher	Ref			Ref			Ref		
**MODEL 2 – (n = 5135) ‡**	Lower	0.005	(-0.008 - 0.018)	0.465	1.182	(1.008 - 1.385)	0.039	1.284	(1.045 - 1.577)	0.017
	Higher	Ref			Ref			Ref		

**P*-value: Wald test.

† Adjusted for intervention with random intercept and intervention slope. Weight gain per week (kg/week) additionally adjusted for BMI (kg/m^2)

‡ Adjusted for intervention, maternal age, parity, ethnicity, with random intercept and intervention slope. Weight gain per week (kg/week) additionally adjusted for BMI (kg/m^2)

Lower education: secondary education or less. Higher education: at least some tertiary education

For peer review only - http://bmjopen.bmj.com/site/about/guidelines.xhtml

Table 5. Generalised Linear Mixed-Models to show relationship between maternal educational attainment, type of intervention and (a) gestational weight gain per week (kg), and (b) gestational weight gain per week as per the IOM guidelines

			(a) Gestational Weight Gain per Week (kg/week)			(b) Gestational Weight Gain per Week as per the IOM Guidelines					
						Excessive Weight Gain			Inadequate Weight Gain		
	**Education**	**Intervention Type**	***B***	**95% CI**	***P****	**OR**	**95% CI**	***P****	**OR**	**95% CI**	***P****
**MODEL 1 †**	**Lower (n = 1910)**	Mixed	-0.044	(-0.069, -0.019)	<0.001	0.745	(0.572, 0.971)	0.029	1.039	(0.745, 1.449)	0.821
		Diet	-0.053	(-0.097, -0.009)	0.018	0.524	(0.351, 0.782)	0.002	0.496	(0.285, 0.864)	0.013
		Exercise	-0.020	(-0.069, 0.029)	0.422	0.704	(0.446, 1.110)	0.131	0.634	(0.333, 1.208)	0.166
		Control	Ref			Ref			Ref		
	**Higher (n = 3273)**	Mixed	-0.056	(-0.071, -0.040)	<0.001	0.930	(0.753, 1.150)	0.504	1.407	(1.057, 1.874)	0.019
		Diet	-0.015	(-0.038, 0.009)	0.213	0.619	(0.446, 0.858)	0.004	0.943	(0.565, 1.574)	0.822
		Exercise	-0.014	(-0.036, 0.008)	0.206	1.027	(0.753, 1.402)	0.865	1.209	(0.770, 1.899)	0.410
		Control	Ref			Ref			Ref		
**MODEL 2 ‡**	**Lower (n = 1884)**	Mixed	-0.044	(-0.069, -0.020)	<0.001	0.735	(0.561, 0.963)	0.026	1.041	(0.744, 1.458)	0.813
		Diet	-0.055	(-0.098, -0.012)	0.012	0.515	(0.339, 0.785)	0.002	0.504	(0.288, 0.884)	0.017
		Exercise	-0.018	(-0.068, 0.032)	0.472	0.660	(0.405, 1.075)	0.095	0.612	(0.314, 1.193)	0.149
		Control	Ref			Ref			Ref		
	**Higher (n = 3251)**	Mixed	-0.053	(-0.069, -0.037)	<0.001	0.947	(0.765, 1.174)	0.620	1.411	(1.056, 1.884)	0.020
		Diet	-0.013	(-0.037, 0.010)	0.271	0.609	(0.437, 0.849)	0.003	0.962	(0.572, 1.616)	0.882
		Exercise	-0.018	(-0.040, 0.004)	0.111	1.030	(0.751, 1.413)	0.855	1.205	(0.761, 1.909)	0.427
		Control	Ref			Ref			Ref		

**P*-value: Wald test
† With fixed effects. Weight gain per week (kg/week) adjusted for BMI (kg/m^2)
‡ With fixed effects. Adjusted for maternal age, parity and ethnicity. Weight gain per week (kg/week) adjusted for BMI (kg/m^2)
Lower education: secondary education or less. Higher education: at least some tertiary education

For peer review only - http://bmjopen.bmj.com/site/about/guidelines.xhtml

Author contributions

E.C. O'Brien	Wrote a proposal to the i-WIP data access committee. Set the objectives, study design and data analysis plan. Analysis and interpretation of data; drafting of manuscript; critical revision; final approval of version to be published; agreeable to be accountable for all aspects of the work.
R. Segurado	Analysis and interpretation of data; drafting of manuscript; critical revision; final approval of version to be published; agreeable to be accountable for all aspects of the work.
A.A. Geraghty	Analysis and interpretation of data; drafting of manuscript; critical revision; final approval of version to be published; agreeable to be accountable for all aspects of the work.
G. Alberdi	Analysis and interpretation of data; drafting of manuscript; critical revision; final approval of version to be published; agreeable to be accountable for all aspects of the work.
E. Rogozińska	i-WIP IPD database manager; study conception and design; critical revision; final approval of version to be published; agreeable to be accountable for all aspects of the work.
A. Astrup	Study conception and design; critical revision; final approval of version to be published; agreeable to be accountable for all aspects of the work.
R. Barakat Carballo	Study conception and design; critical revision; final approval of version to be published; agreeable to be accountable for all aspects of the work.
A. Bogaerts	Study conception and design; critical revision; final approval of version to be published; agreeable to be accountable for all aspects of the work.
J.G. Cecatti	Study conception and design; critical revision; final approval of version to be published; agreeable to be accountable for all aspects of the work.
A. Coomarasamy	Study conception and design; critical revision; final approval of version to be published; agreeable to be accountable for all aspects of the work.
C.J. de Groot	Study conception and design; critical revision; final approval of version to be published; agreeable to be accountable for all aspects of the work.
R. Devlieger	Study conception and design; critical revision; final approval of version to be published; agreeable to be accountable for all aspects of the work.
J.Dodd	Study conception and design; critical revision; final approval of version to be published; agreeable to be accountable for all aspects of the work.
N.El Beltagy	Study conception and design; critical revision; final approval of version to be published; agreeable to be accountable for all aspects of the work.
F. Facchinetti	Study conception and design; critical revision; final approval of version to be published; agreeable to be accountable for all aspects of the work.
N.R. Geiker	Study conception and design; critical revision; final approval of

For peer review only

	version to be published; agreeable to be accountable for all aspects of the work.
K.J Guelfi	Study conception and design; critical revision; final approval of version to be published; agreeable to be accountable for all aspects of the work.
L. Haakstad	Study conception and design; critical revision; final approval of version to be published; agreeable to be accountable for all aspects of the work.
C.Harrison	Study conception and design; critical revision; final approval of version to be published; agreeable to be accountable for all aspects of the work.
H. Hauner	Study conception and design; critical revision; final approval of version to be published; agreeable to be accountable for all aspects of the work.
D.M. Jensen	Study conception and design; critical revision; final approval of version to be published; agreeable to be accountable for all aspects of the work.
K.S. Khan	Study conception and design; critical revision; final approval of version to be published; agreeable to be accountable for all aspects of the work.
T. I. Kinnunen	Study conception and design; critical revision; final approval of version to be published; agreeable to be accountable for all aspects of the work.
R. Luoto	Study conception and design; critical revision; final approval of version to be published; agreeable to be accountable for all aspects of the work.
B. W. Mol	Study conception and design; critical revision; final approval of version to be published; agreeable to be accountable for all aspects of the work.
S. Mørkved	Study conception and design; critical revision; final approval of version to be published; agreeable to be accountable for all aspects of the work.
N. Motahari	Study conception and design; critical revision; final approval of version to be published; agreeable to be accountable for all aspects of the work.
J. Owens	Study conception and design; critical revision; final approval of version to be published; agreeable to be accountable for all aspects of the work.
M. Perales	Study conception and design; critical revision; final approval of version to be published; agreeable to be accountable for all aspects of the work.
E. Petrella	Study conception and design; critical revision; final approval of version to be published; agreeable to be accountable for all aspects of the work.
S. Phelan	Study conception and design; critical revision; final approval of version to be published; agreeable to be accountable for all aspects of the work.
L. Poston	Study conception and design; critical revision; final approval of version to be published; agreeable to be accountable for all aspects of the work.
K. Rauh	Study conception and design; critical revision; final approval of

For peer review only

For peer review only - http://bmjopen.bmj.com/site/about/guidelines.xhtml

	version to be published; agreeable to be accountable for all aspects of the work.
G. Rayanagoudar	Study conception and design; critical revision; final approval of version to be published; agreeable to be accountable for all aspects of the work.
K.M. Renault	Study conception and design; critical revision; final approval of version to be published; agreeable to be accountable for all aspects of the work.
A.E. Ruifrok	Study conception and design; critical revision; final approval of version to be published; agreeable to be accountable for all aspects of the work.
L.R. Sagedal	Study conception and design; critical revision; final approval of version to be published; agreeable to be accountable for all aspects of the work.
K.Å Salvesen	Study conception and design; critical revision; final approval of version to be published; agreeable to be accountable for all aspects of the work.
T.T. Scudeller	Study conception and design; critical revision; final approval of version to be published; agreeable to be accountable for all aspects of the work.
G.X. Shen	Study conception and design; critical revision; final approval of version to be published; agreeable to be accountable for all aspects of the work.
A. Shub	Study conception and design; critical revision; final approval of version to be published; agreeable to be accountable for all aspects of the work.
S.N. Stafne	Study conception and design; critical revision; final approval of version to be published; agreeable to be accountable for all aspects of the work.
F. Surita	Study conception and design; critical revision; final approval of version to be published; agreeable to be accountable for all aspects of the work.
S. Thangaratinam	Study conception and design; critical revision; final approval of version to be published; agreeable to be accountable for all aspects of the work.
S. Tonstad	Study conception and design; critical revision; final approval of version to be published; agreeable to be accountable for all aspects of the work.
M.N. van Poppel	Study conception and design; critical revision; final approval of version to be published; agreeable to be accountable for all aspects of the work.
C. Vinter	Study conception and design; critical revision; final approval of version to be published; agreeable to be accountable for all aspects of the work.
I. Vistad	Study conception and design; critical revision; final approval of version to be published; agreeable to be accountable for all aspects of the work.
S. Yeo	Study conception and design; critical revision; final approval of version to be published; agreeable to be accountable for all aspects of the work.
i-WIP (International	NA – group name

For peer review only

Weight Management in Pregnancy) Collaborative Group	
F.M. McAuliffe	Wrote a proposal to the i-WIP data access committee. Set the objectives, study design and data analysis plan. Analysis and interpretation of data; drafting of manuscript; critical revision; final approval of version to be published; agreeable to be accountable for all aspects of the work.

Competing interests statement

The authors did not report any potential conflicts of interest

Funding Statement

The data come from a National Institute for Health Research (NIHR) funded project (HTA - 12/01/50) and the Queen Mary University of London is its legal Sponsor. The database was initially funded by the NIHR and is currently funded by World Health Organization (WHO). This work was also supported by the Health Research Board, Health Research Centre for Health and Diet Research, Ireland.

Data sharing statement

The Queen Mary University of London is the legal custodian of the i-WIP dataset. The access to the dataset is regulated by prespecified terms and conditions (available on request) and overseen by Data Access Committee. For further information visit: https://iwipgroup.wixsite.com/collaboration/related-projects

Acknowledgements

We are also grateful to the i-WIP Network for the individual participant data shared and to Helena Teede, Janette Khoury and Márcia Vitolo for the contribution of data.

For peer review only

References

1. United Nations. Transforming Our World: The 2030 Agenda for Sustainable Development. New York; 2015.
2. Agyemang C, Vrijkotte TGM, Droomers M, van der Wal MF, Bonsel GJ, Stronks K. The effect of neighbourhood income and deprivation on pregnancy outcomes in Amsterdam, The Netherlands. J Epidemiol Community Health. 2009;63(9):755-760.
3. Metcalfe A, Lail P, Ghali WA, Sauve RS. The association between neighbourhoods and adverse birth outcomes: a systematic review and meta-analysis of multi-level studies. Paediatr Perinat Epidemiol. 2011;25(3):236-245.
4. Morgen CS, Bjørk C, Andersen PK, Mortensen LH, Nybo Andersen A-M, Bjork C, et al. Socioeconomic position and the risk of preterm birth-a study within the Danish National Birth Cohort. Int J Epidemiol. 2008;37:1109-1120.
5. Silva LM, Jansen PW, Steegers E a P, Jaddoe VW V, Arends LR, Tiemeier H, et al. Mother's educational level and fetal growth: The genesis of health inequalities. Int J Epidemiol. 2010;39:1250-1261.
6. Blumenshine P, Egerter S, Barclay CJ, Cubbin C, Braveman PA. Socioeconomic Disparities in Adverse Birth Outcomes. Am J Prev Med. 2010;39(3):263-272.
7. Tofani AA, Lamarca G de A, Sheiham A, Vettore MV. The different effects of neighbourhood and individual social capital on health-compromising behaviours in women during pregnancy: a multi-level analysis. BMC Public Health. 2015;15:890.
8. Freisling H, Elmadfa I, Gall I. The effect of socioeconomic status on dietary intake, physical activity and Body Mass Index in Austrian pregnant women. J Hum Nutr Diet. 2006;19:437-445.
9. O'Brien EC, Alberdi G, Geraghty AA, McAuliffe FM. Lower education predicts poor response to dietary intervention in pregnancy, regardless of neighbourhood

For peer review only - http://bmjopen.bmj.com/site/about/guidelines.xhtml

affluence: secondary analysis from the ROLO randomised control trial. Public Health Nutr. 2017;20(16):2959–69.

10. O'Brien EC, Alberdi G, McAuliffe FM. The influence of socioeconomic status on gestational weight gain: a systematic review. J Public Heal. 2018;40(1):41-55

11. Truong YN, Yee LM, Caughey AB, Cheng YW. Weight gain in pregnancy: does the Institute of Medicine have it right? Am J Obstet Gynecol. 2015;212(3):362.e1-e8.

12. Goldstein RF, Abell SK, Ranasinha S, Misso M, Boyle J a., Black MH, et al. Association of gestational weight gain with maternal and infant outcomes: A systematic review and meta-analysis. JAMA. 2017;317(21):2207-2225.

13. Amorim AR, Rössner S, Neovius M, Lourenço PM, Linné Y. Does excess pregnancy weight gain constitute a major risk for increasing long-term BMI? Obesity (Silver Spring). 2007;15(5):1278-1286.

14. NCD Risk Factor Collaboration (NCD-RisC). Trends in adult body-mass index in 200 countries from 1975 to 2014: a pooled analysis of 1698 population-based measurement studies with 19.2 million participants. Lancet. 2016;387(10026):1377-1396.

15. Institute of Medicine. National Research Council (US) Committee to Reexamine IOM Pregnancy Weight Guidelines. Weight Gain During Pregnancy. Washington (DC): National Academies Press (US); 2009.

16. O'Brien OA, Lindsay KL, McCarthy M, McGloin AF, Kennelly M, Scully HA, et al. Influences on the food choices and physical activity behaviours of overweight and obese pregnant women: A qualitative study. Midwifery. 2017;(47):28-35.

17. Jackson RA, Stotland NE, Caughey AB, Gerbert B. Improving diet and exercise in pregnancy with Video Doctor counseling: A randomized trial. Patient Educ Couns. 2011;83(2):203-209.

For peer review only

18. Thangaratinam S, Rogozinska E, Jolly K, Glinkowski S, Duda W, Borowiack E, et al. Interventions to reduce or prevent obesity in pregnant women: a systematic review. Health Technol Assess. 2012;16(31).

19. Ruifrok AE, Rogozinska E, van Poppel MNM, Rayanagoudar G, Kerry S, de Groot CJM, et al. Study protocol: differential effects of diet and physical activity based interventions in pregnancy on maternal and fetal outcomes--individual patient data (IPD) meta-analysis and health economic evaluation. Syst Rev. 2014;3:131.

20. Stewart LA, Clarke M, Rovers M, Riley RD, Simmonds M, Stewart G, et al. Preferred Reporting Items for a Systematic Review and Meta-analysis of Individual Participant Data. JAMA. 2015;313(16):1657.

21. Thangaratinam S, Rogozinska E, Jolly K, Glinkowski S, Roseboom T, Tomlinson JW, et al. Effects of interventions in pregnancy on maternal weight and obstetric outcomes: meta-analysis of randomised evidence. BMJ. 2012;344:e2088.

22. Ruifrok AE, Rogozinska E, van Poppel MNM, Rayanagoudar G, Kerry S, de Groot CJM, et al. Erratum to: Study protocol: differential effects of diet and physical activity based interventions in pregnancy on maternal and fetal outcomes: individual patient data (IPD) meta-analysis and health economic evaluation. Syst Rev. 2015;4:101.

23. Shavers VL. Measurement of socioeconomic status in health disparities research. J Natl Med Assoc. 2007;99(9):1013-1023.

24. Althuizen E, van der Wijden C, van Mechelen W, Seidell J, van Poppel M. The effect of a counselling intervention on weight changes during and after pregnancy: a randomised trial. BJOG. 2013;120:92-99.

25. Baciuk EP, Pereira RI, Cecatti JG, Braga AF, Cavalcante SR. Water aerobics in pregnancy: Cardiovascular response, labor and neonatal outcomes. Reprod Health. 2008;5:10.

For peer review only - http://bmjopen.bmj.com/site/about/guidelines.xhtml

26. Bogaerts AFL, Devlieger R, Nuyts E, Witters I, Gyselaers W, van den Bergh BRH. Effects of lifestyle intervention in obese pregnant women on gestational weight gain and mental health: a randomized controlled trial. Int J Obes. 2013;37(6):814-821.

27. Haakstad LAH, Bø K. Exercise in pregnant women and birth weight: a randomized controlled trial. BMC Pregnancy Childbirth. 2011;11:66.

28. Harrison CL, Lombard CB, Strauss BJ, Teede HJ. Optimizing healthy gestational weight gain in women at high risk of gestational diabetes: A randomized controlled trial. Obesity. 2013;21(5):904-909.

29. Jeffries K, Shub A, Walker SP, Hiscock R, Permezel M. Reducing excessive weight gain in pregnancy: a randomised controlled trial. Med J Aust. 2009;191(8):429-433.

30. Khaledan A, Mirdar S, Motahari Tabari N, Ahmad Shirvani M. Effect of an Aerobic Exercise Program on Fetal Growth in Pregnant Women. J Hayat. 2010;16(1):55-64.

31. Khoury J, Henriksen T, Christophersen B, Tonstad S. Effect of a cholesterol-lowering diet on maternal, cord, and neonatal lipids, and pregnancy outcome: A randomized clinical trial. Am J Obstet Gynecol. 2005;193(4):1292-1301.

32. Nascimento S, Surita F, Parpinelli M, Siani S, Pinto e Silva J. The effect of an antenatal physical exercise programme on maternal/perinatal outcomes and quality of life in overweight and obese pregnant women: a randomised clinical trial. BJOG. 2011;118:1455-1463.

33. Oostdam N, van Poppel M, Wouters M, Eekhoff E, Bekedam D, Kuchenbecker W, et al. No effect of the FitFor2 exercise programme on blood glucose, insulin sensitivity, and birthweight in pregnant women who were overweight and at risk for gestational diabetes: results of a randomised controlled trial. BJOG. 2012;119:1098-1107.

34. Petrella E, Malavolti M, Bertarini V, Pignatti L, Neri I, Battistini NC, et al. Gestational weight gain in overweight and obese women enrolled in a healthy lifestyle and eating habits program. J Matern Neonatal Med. 2014;27(13):1348-1352.

For peer review only

35. Phelan S, Phipps MG, Abrams B, Darroch F, Schaffner A, Wing RR. Randomized trial of a behavioral intervention to prevent excessive gestational weight gain: the Fit for Delivery Study. Am J Clin Nutr. 2011;93(4):772-779.

36. Poston L, Bell R, Croker H, Flynn AC, Godfrey KM, Goff L, et al. Effect of a behavioural intervention in obese pregnant women (the UPBEAT study): a multicentre, randomised controlled trial. Lancet Diabetes Endocrinol. 2015;3(10):767-777.

37. Rauh K, Gabriel E, Kerschbaum E, Schuster T, von Kries R, Amann-Gassner U, et al. Safety and efficacy of a lifestyle intervention for pregnant women to prevent excessive maternal weight gain: a cluster-randomized controlled trial. BMC Pregnancy Childbirth. 2013;13(1):151.

38. Renault KM, N Nørgaard K, Nilas L, Carlsen EM, Cortes D, Pryds O, et al. The Treatment of Obese Pregnant Women (TOP) study: A randomized controlled trial of the effect of physical activity intervention assessed by pedometer with or without dietary intervention in obese pregnant women. Am J Obstet Gynecol. 2014;210(2):134.e1-e134.e9.

39. Sagedal L, Øverby N, Bere E, Torstveit M, Lohne-Seiler H, Småstuen M, et al. Lifestyle intervention to limit gestational weight gain: the Norwegian Fit for Delivery randomised controlled trial. BJOG. 2016. doi: 10.1111/1471-0528.13862

40. Stafne SN, Salvesen KÅ, Romundstad PR, Stuge B, Mørkved S. Does regular exercise during pregnancy influence lumbopelvic pain? A randomized controlled trial. Acta Obstet Gynecol Scand. 2012;91:552-559.

41. Vinter CA, Jensen DM, Ovesen P, Beck-Nielsen H, Jørgensen JS. The LiP (Lifestyle in Pregnancy) study: a randomized controlled trial of lifestyle intervention in 360 obese pregnant women. Diabetes Care. 2011;34(12):2502-2507.

For peer review only

For peer review only - http://bmjopen.bmj.com/site/about/guidelines.xhtml

42. Vítolo MR, Bueno MSF, Gama CM. [Impact of a dietary counseling program on the gain weight speed of pregnant women attended in a primary care service]. Rev Bras Ginecol Obs. 2011;33(1):13-9.

43. Walsh JM, McGowan CA, Mahony R, Foley ME, McAuliffe FM. Low glycaemic index diet in pregnancy to prevent macrosomia (ROLO study): randomised control trial. BMJ. 2012;345:e5605.

44. Wolff S, Legarth J, Vangsgaard K, Toubro S, Astrup A. A randomized trial of the effects of dietary counseling on gestational weight gain and glucose metabolism in obese pregnant women. Int J Obes. 2008;32:495-501.

45. Muktabhant B, Lawrie TA, Lumbiganon P, Laopaiboon M. Diet or exercise, or both, for preventing excessive weight gain in pregnancy. Cochrane Database Syst Rev. 2015;6:CD007145.

46. Institute of Medicine (US) and Food and Nutritional Board. Historical Trends in Clinical Practice, Maternal Nutritional Status, and the Course and Outcome of Pregnancy. In: Nutrition During Pregnancy. Washington (DC); 1990:37-38.

47. Duncombe D, Wertheim EH, Skouteris H, Paxton SJ, Kelly L. Factors related to exercise over the course of pregnancy including women's beliefs about the safety of exercise during pregnancy. Midwifery. 2009;25(4):430-438.

48. Heery E, McConnon A, Kelleher CC, Wall PG, McAuliffe FM. Perspectives on weight gain and lifestyle practices during pregnancy among women with a history of macrosomia: a qualitative study in the Republic of Ireland. BMC Pregnancy Childbirth. 2013;13:202.

49. Cioffi J, Schmied V, Dahlen H, Mills A, Thornton C, Duff M, et al. Physical Activity in Pregnancy: Women's Perceptions, Practices, and Influencing Factors. J Midwifery Womens Health. 2010;55(5):455-461

For peer review only - http://bmjopen.bmj.com/site/about/guidelines.xhtml

50. Weisman CS, Hillemeier MM, Chase GA, Misra DP, Chuang CH, Parrott R, et al. Women's perceived control of their birth outcomes in the Central Pennsylvania Women's Health Study: implications for the use of preconception care. Womens Health Issues. 2008;18(1):17-25.

51. Wardle J, Steptoe A. Socioeconomic differences in attitudes and beliefs about healthy lifestyles. J Epidemiol Community Health. 2003;57(6):440-443.

52. Stewart LA, Clarke M, Rovers M, Riley RD, Simmonds M, Stewart G, et al. Preferred Reporting Items for a Systematic Review and Meta-analysis of Individual Participant Data. JAMA. 2015;313(16):1657.

53. Gilmore LA, Redman LM. Weight gain in pregnancy and application of the 2009 IOM guidelines: toward a uniform approach. Obesity (Silver Spring). 2015;23(3):507-511.

54. Krukowski RA, West DS, DiCarlo M, Shankar K, Cleves MA, Saylors ME, et al. Are early first trimester weights valid proxies for preconception weight? BMC Pregnancy Childbirth. 2016;16(1):357.

55. Kapadia MZ, Park CK, Beyene J, Giglia L, Maxwell C, McDonald SD. Can we safely recommend gestational weight gain below the 2009 guidelines in obese women? A systematic review and meta-analysis. Obes Rev. 2015;16(3):189-206.

56. Cheikh Ismail L, Bishop DC, Pang R, Ohuma EO, Kac G, Abrams B, et al. Gestational weight gain standards based on women enrolled in the Fetal Growth Longitudinal Study of the INTERGROWTH-21st Project: a prospective longitudinal cohort study. BMJ. 2016;352:i555.

Figure Captions

Figure 1. Gestational weight gain (kg/week), according to pre-pregnancy BMI, intervention and educational attainment

For peer review only - http://bmjopen.bmj.com/site/about/guidelines.xhtml

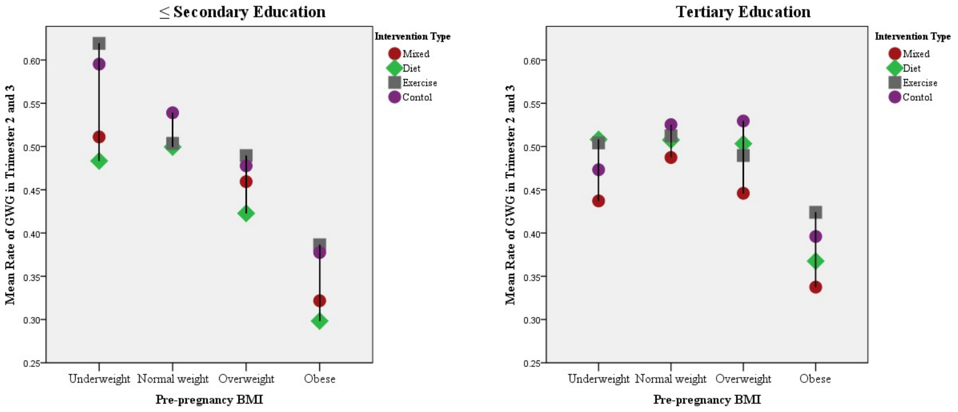

Figure 1. Gestational weight gain (kg/week), according to pre-pregnancy BMI, intervention and educational attainment

440x190mm (72 x 72 DPI)

Supplementary Files

For peer review only

Table S1. Study Characteristics of Randomised Control Trials Included in the IPD Meta-Analysis (n = 21)

Author (Year)	Period	Original inclusion/exclusion criteria	Country	Method to measure gestational weight gain (GWG)	Intervention	Intervention Detail	Outcome	Original i-WIP N	N (%) in this analysis
Althuizen *et al*. (2013)	February 2005 to May 2006	Inclusion criteria: primigravida, ability to read and speak Dutch, ≤14 weeks' gestation.	Netherlands	Pre-pregnancy: self-reported. Early pregnancy: measured at 15 weeks' gestation	Mixed	Counselling sessions (The New Life[style] Study) five times during pregnancy (15 minutes each) on weight, physical activity and diet.	Excessive weight gain, BMI, postpartum weight retention, birth weight, macrosomia, preterm birth, gestational diabetes	258	187 (72.5%)
Baciuk *et al*. (2008)	Not described	Inclusion criteria: <20 weeks' gestation, singleton pregnancy, receiving prenatal care at the institution and intended to give birth there. Exclusion criteria: engaging in regular physical activity, ≥2 caesarean sections, neurological, cardio-vascular, pulmonary, muscular-skeletal or endocrine disorders, morbid obesity, severe anaemia or vaginal bleeding during pregnancy.	Brazil	Pre-pregnancy: self-reported. Early pregnancy: (estimated from pre-pregnancy as per methods)	Exercise	Water aerobics three times per week (50 minutes each) in an indoor swimming pool.	Maternal cardiovascular capacity, heart rate, blood pressure, experience at delivery and fetal heart rate	71	68 (95.8%)
Bogaerts *et al*. (2013)	March 2008 and April 2011	Inclusion: BMI ≥29 kg/m^2. Exclusion criteria: >15 weeks' gestation, pre-existing type 1 diabetes, multiple pregnancy, primary need for nutritional advice and insufficient knowledge of the Dutch language	Belgium	Pre-pregnancy self-reported. Early pregnancy: (estimated from pre-pregnancy as per methods)	Mixed	Two arms. (1) Brochure group: brochure with advice on diet, physical activity and limiting excessive GWG, (2) Prenatal session group: brochure plus four prenatal motivational lifestyle intervention sessions led by a trained midwife.	GWG, Anxiety, feelings of depression	197	196 (99.5%)
Haakstad *et al*. (2011)	September 2007 to March 2008	Inclusion criteria: Nulliparous, low pre-pregnancy exercise, sufficient understanding of Norwegian, and ≤24 weeks' gestation. Exclusion criteria: history of miscarriages, severe heart disease, persistent bleeding after 12 weeks' gestation, multiple pregnancy, poorly controlled thyroid disease, pregnancy-induced hypertension or pre-eclampsia, diabetes or gestational diabetes, and other diseases that could interfere with participation.	Norway	Pre-pregnancy self-reported. Early pregnancy: measured at 12-24 weeks' gestation	Exercise	Aerobic dance/strength training class 2-3 times per week (one hour each) for a minimum of 12 weeks. Advised 30 minutes of moderate self-imposed physical activity on the remaining week-days (recorded in a personal training diary).	Infant birth weight, gestational age at delivery, Apgar score.	105	83 (79.0%)

For peer review only

Table S1. cont.

Author (Year)	Period	Original inclusion/exclusion criteria	Country	Method to measure GWG	Intervention	Intervention Detail	Outcome	Original i-WIP N / N (%) in this analysis
Harrison *et al*. (2013)	Not described	Inclusion criteria: 12-15 weeks' gestation, BMI ≥25 or ≥23 kg/m^2 if high-risk ethnicity (Polynesian, Asian, and African populations) or ≥30 kg/m^2, increased risk of GDM identified by risk prediction tool, agreement to complete an OGTT at 28 weeks' gestation. Exclusion criteria: multiple pregnancies, type 1 or 2 diabetes, a BMI ≥45 kg/m^2, pre-existing chronic medical conditions, and non-English-speaking	Australia	Pre-pregnancy: self-reported Early pregnancy: measured at 12-15 weeks' gestation.	Mixed	Behaviour change lifestyle intervention with four individual sessions based on the Social Cognitive Theory, at 14-16, 20, 24, and 28 weeks' gestation. Provided with simple healthy eating and physical activity messages. Behavioural change strategies were practised to identify short-term goals, and increase self-efficacy and self-monitoring. Goals were determined individually by the participants. Self-monitoring strategies included pedometers and the use of weight gain charts.	GWG, GDM screening	238 / 187 (78.6%)
Jeffries *et al*. (2009)	July and October 2007	Inclusion criteria: ≤14 weeks' gestation. Exclusion criteria were: age <18 years or >45 years, type 1 or type 2 diabetes mellitus, multiple pregnancy, or non-English speaking.	Australia	Pre-pregnancy self-reported. Early pregnancy: measured at 14 weeks' gestation	Mixed	Advised on optimal GWG based on IOM 1990 guidelines. Provided with a personalised weight measurement card and instructed to record their weight at 16, 20, 24, 28, 30, 32 and 34 weeks' gestation.	GWG, adherence to IOM guidelines (1990)	286 / 236 (82.5%)
Khaledan *et al.* (2010)	Not described	Inclusion criteria: Singleton, healthy pregnancy. Exclusion criteria: chronic disease, multiple pregnancy, complications, risk of preterm delivery, hypertension, severe anaemia, type 1 diabetes with poor control, morbid obesity, low maternal weight, history of completely sedentary lifestyle, heavy smoker.	Iran	Pre-pregnancy: na. Early pregnancy: Not described	Exercise	Aerobic (stepping) and flexibility exercise session three times per week (30-45 minutes each) for eight weeks.	Type of delivery, cause of caesarean section, maternal and fetal weight, birth weight and gestational age	39 / 37 (94.9%)
Khoury *et al*. (2005)	June 1999 to November 2001	Inclusion criteria: 17-20 weeks' gestation, singleton, healthy pregnancy, non-smoker (or stopped ≥5 years previously), 21-38 years, BMI 19-32 kg/m^2. Exclusion criteria: following a vegetarian or Mediterranean diet, immigrant to Norway from non-Western country, high-risk pregnancy, complications in a previous pregnancy, on-going hyperemesis gravidarum or bleeding after gestational week 12	Norway	Pre-pregnancy: na. Early pregnancy: measured at 17-20 week's gestation	Diet	Dietary advice from dietitian at inclusion, 24, 30 and 36 weeks' gestation. Cholesterol-lowering diet; promoted fish, low-fat meats and dairy products, oils, whole grains, fruits, vegetables, and legumes from gestational week 17-20 until delivery.	Maternal, cord, and neonatal plasma lipids and pregnancy-related complications, including hypertensive complications, preterm delivery, and intrauterine growth restriction, birth weight and maternal weight gain.	290 / 198 (68.3%)

For peer review only

Table S1. cont.

Author (Year)	Period	Original inclusion/exclusion criteria	Country	Method to measure GWG	Intervention	Intervention Detail	Outcome	Original i-WIP N	N (%) in this analysis
Nascimento *et al.* (2011)	August 2008 to October 2009	Inclusion criteria: overweight or obese, age >18 years, 14-24 weeks' gestation. Exclusion criteria: multiple gestations, exercising regularly, conditions that contraindicate exercise	Brazil	Pre-pregnancy self-reported. Early pregnancy: measured at 14-24 weeks' gestation	Exercise	One exercise class per week (40 minutes each), under the guidance of a trained physical therapist. Light-intensity to moderate-intensity exercises. Advised to exercise at home five times per week.	GWG, maternal arterial blood pressure and perinatal outcome, perception of quality of life.	82	77 (93.9%)
Oostdam *et al.* (2012)	November 2007 and April 2010	Inclusion criteria: obese or overweight and at least one of the following characteristics: history of macrosomia; history of GDM; or first-grade relative with type 2 diabetes. Exclusion criteria: >20 weeks' gestation; <18 years; inadequate knowledge of Dutch language; GDM; hypertension; alcohol abuse; drug abuse; use of any medication that affects insulin secretion or insulin sensitivity; chronic disease that could prevent the woman from implementing the study protocol.	Netherlands	Pre-pregnancy self-reported. Early pregnancy: measured at 15-20 weeks' gestation	Exercise	Aerobic and strength exercise classes (FitFor2 Study). Two days per week (60 minutes each), under supervision of physiotherapist, from 14-20 weeks' gestation to end of pregnancy.	Fasting blood glucose, insulin, HbA1c; body weight, BMI, daily physical activity, birth weight and fetal growth.	124	75 (60.5%)
Petrella *et al.* (2014)	April 2011 to October 2011	Inclusion criteria: Pre-pregnancy BMI ≥25 kg/m^2, >18 years and singleton pregnancy. Exclusion criteria: twin pregnancy, chronic diseases (i.e. diabetes mellitus, chronic hypertension, untreated thyroid diseases), previous GDM, smoker, previous bariatric surgery, regular physical activity, dietary supplements or herbal products known to affect body weight, other medical conditions that might affect body weight, and plans to deliver outside the centre	Italy	Pre-pregnancy self-reported. Early pregnancy: measured at 12 weeks' gestation	Mixed	Diet, physical activity and counselling about GWG. Dietary advice: low glycaemic index diet, three main meals and three snacks (1500 kcal/day). Physical activity advice: 3 days per week of moderate intensity activity (30 minutes each), monitored using pedometer. One-hour counselling session about appropriate GWG.	Excessive weight gain, gestational diabetes mellitus, gestational hypertension and preterm delivery.	61	58 (95.1%)

For peer review only

Table S1. cont.

Author (Year)	Period	Original inclusion/exclusion criteria	Country	Method to measure GWG	Intervention	Intervention Detail	Outcome	Original i-WIP N<br>N (%) in this analysis
Phelan *et al*. (2011)	2006 to 2008	Inclusion criteria: 10 - 16 week's gestation, BMI 19.8 - 40 kg/m², non-smoking, >18 years, fluency in English, access to a telephone, and a singleton pregnancy. Exclusion criteria: self-reported major health or psychiatric diseases, weight loss during pregnancy, or a history of >3 miscarriages	USA	Pre-pregnancy self-reported. Early pregnancy: measured at 10-16 weeks' gestation	Mixed	Behavioural intervention with diet and physical activity advice (Fit for Delivery Study). One face-to-face visit to discuss appropriate GWG, physical activity (30 min of walking most days of the week), and calorie goals (20 kcal/kg), with daily self-monitoring of eating, exercise, and weight. Provided with weight scales, food records, pedometers, weekly reminders, personalised graphs of weight gained with feedback, 3 supportive phone calls from the dietitian. If gained over or under weight gain guidelines received 2 additional supportive phone calls per month with meal plans and goals until weight gains returned to appropriate amounts.	GWG and the proportion who returned to pre-gravid weights by 6 months' postpartum.	401<br>395 (98.5%)
Poston *et al*. (2015)	March 2009 to June 2014	Inclusion criteria: >16 years, BMI ≥30 kg/m², singleton pregnancy, 15 - 18(+6) weeks' gestation. Exclusion criteria: unwilling or unable to give informed consent; underlying disorders, including a pre-pregnancy diagnosis of essential hypertension, diabetes, renal disease, systemic lupus erythematosus, antiphospholipid syndrome, sickle- cell disease, thalassaemia, coeliac disease, thyroid disease, and current psychosis; or currently being prescribed metformin.	UK	Pre-pregnancy: na. Early pregnancy: measured at 15-18 week's gestation	Mixed	Behavioural intervention with diet and physical activity advice (UPBEAT Study). One individual interview plus one group/individual session per week (60 minutes each) for 8 weeks. Advised to self-monitor, identify, and problem-solve of barriers to behaviour change and enlist social support. Dietary intervention aimed to promote a healthy pattern of eating and low glycaemic index diet. Exercise advice focused on incremental increases in walking from a pedometer- assessed baseline, tailored to pre-existing activities.	Gestational diabetes and large-for-gestational-age infants (≥90th customised birth weight centile)	1554<br>378 (24.3%)

For peer review only

Table S1. cont.

Author (Year)	Period	Original inclusion/exclusion criteria	Country	Method to measure GWG	Intervention	Intervention Detail	Outcome	Original i-WIP N	N (%) in this analysis
Rauh *et al*. (2013)	February 2010 and August 2011	Inclusion criteria: >18 years; singleton pregnancy; <18 weeks' gestation; BMI ≥18.5 kg/m^2, and sufficient German. Exclusion criteria: any condition preventing physical activity, GDM and uncontrolled chronic diseases that may affect weight development like thyroid dysfunction or psychiatric diseases	Germany	Pre-pregnancy self-reported. Early pregnancy: measured at 10-16 weeks' gestation	Mixed	Individual counselling sessions on nutrition, physical activity, and GWG monitoring (FeLIPO Study). Twice during pregnancy; 20th (60 minutes) and 30th (30 minutes) week of gestation. Provided with general healthy lifestyle information; prompted to self-monitor behaviour by recording diet, physical activity, and weight gain using weight gain charts; and to set behavioural goals based on the baseline situation (BMI, diet, physical activity) and individual preferences.	Weight gain in excess of IOM recommendations, and weight retention at 4 months' postpartum	250	231 (92.4%)
Renault *et al*. (2014)	March 2009 to March 2012	Inclusion criteria: >18 years, singleton pregnancy, normal scan in weeks 11-14, <16 weeks' gestation, sufficient Danish. Exclusion criteria: multiple pregnancy, pre-gestational diabetes, or other serious diseases limiting level of physical activity, previous bariatric surgery, or alcohol or drug abuse.	Denmark	Pre-pregnancy self-reported. Early pregnancy: measured at 11-16 weeks' gestation	Mixed	Physical activity intervention with or without dietary intervention (TOP Study). Physical activity advice: daily step count of 11,000, monitored with pedometer. Diet: hypocaloric (1200-1675 kcal), low-fat, Mediterranean-style diet and contact with an experienced dietitian every 2 weeks, alternating between outpatient visits and phone contacts (measurement of weight, encouragement, and correcting advice on the diet).	GWG, pregnancy complications and delivery complications.	425	370 (87.1%)
Sagedal *et al*. (2017)	September 2009 and February 2013	Inclusion criteria: Nulliparous, singleton pregnancy, ≤20 weeks' gestation, pre-pregnancy BMI of ≥19 kg/m^2, sufficient in Norwegian or English, and provided signed, informed consent. Exclusion criteria: pre-existing diabetes, disabilities precluding participation in a physical fitness programme, continued substance abuse, or planned relocation outside of the study area before delivery.	Norway	Pre-pregnancy self-reported. Early pregnancy: measured at ≤20 weeks' gestation	Mixed	Lifestyle intervention with diet and physical activity components (Norwegian Fit for Delivery Study). Dietary advice provided by telephone, with one initial consultation and follow-up 4–6 weeks later (20 minutes). Dietary advice aimed to increase awareness of food choices. GWG based on pre-pregnancy BMI provided. Physical activity: access to exercise classes at a local gym twice/week (60 minutes). Provided with booklets, access to study website, invitation to one cooking class and to an evening meeting.	GWG, birth weight of term infants, maternal fasting glucose levels at 30 weeks of gestation, maternal percentage of fat, incidence of operative deliveries, postpartum weight	606	576 (95.0%)

For peer review only

Table S1. cont.

Author (Year)	Period	Original inclusion/exclusion criteria	Country	Method to measure GWG	Intervention	Intervention Detail	Outcome	Original i-WIP N	N (%) in this analysis
Stafne *et al*. (2012)	April 2007 to June 2009 and October 2007 to January 2009	Inclusion criteria: Residing within 30 min drive from the hospital and available to attend weekly appointment, ≥18 years, singleton pregnancy. Exclusion criteria: High-risk pregnancies, diseases that could interfere with participation.	Norway	Pre-pregnancy: na. Early pregnancy: measured at 18-22 week's gestation	Exercise	Exercise sessions, once per week for 12 weeks (60 minutes each), in groups of 8–15 women, instructed by a physiotherapist. Offered between 20 and 36 weeks of pregnancy. Included aerobic activity, strength training and balance exercises. Women encouraged to follow a written home exercise program at least twice per week (45 minutes each).	Self-reported lumbopelvic pain and sick leave due to lumbopelvic pain.	855	699 (81.8%)
Vinter *et al*. (2011)	October 2007 to October 2010	Inclusion criteria: 18–40 years, 10–14 weeks' gestation, BMI 30–45 kg/m^2. Exclusion criteria: chronic medical conditions, prior serious obstetric complications, alcohol or drug abuse, non-Danish speaking, multiple pregnancy, positive OGTT.	Denmark	Pre-pregnancy: self-reported. Early pregnancy: measured at 10-14 week's gestation	Mixed	Dietary counselling and physical activity (The LiP Study - Lifestyle in Pregnancy). Four dietary counselling by dietitians at 15, 20, 28, and 35 weeks' gestation. Dietary advice was based on the official Danish recommendations, and aimed to limit GWG to 5 kg. Provided with free full-time membership in a fitness centre for 6 months, with exercise classes once per week (60 minutes each) with physiotherapists. Encouraged to be moderately physically active every day (30–60 minutes per day), provided with a pedometer to motivate and improve daily activity.	GWG, preeclampsia, pregnancy-induced hypertension, GDM, caesarean section, macrosomia, and admission to neonatal intensive care unit.	304	292 (96.1%)
Vitolo *et al*. (2011)	January 2007 to May 2008	Inclusion criteria: 10-29 weeks' gestation. Exclusion criteria: positive HIV test previous diagnosis of diabetes, hypertension, anaemia, any conditions preventing women from undertaking, exercise in pregnancy, and >35 years	Brazil	Pre-pregnancy: self-reported. Early pregnancy: collected in first trimester (method not described)	Diet	Dietary counselling provided according to nutritional needs. Underweight women advised to increase energy density of the diet, include two high-energy snacks and fruit daily. Well-nourished women advised to consume six portions fruit and vegetables and restrict energy dense foods. Overweight women were advised on meal timing, portion sizes, to restrict energy dense and processed foods.	GWG, gestational diabetes, pre-eclampsia, infant birth and premature delivery (<37wks), 1st and 5th min Apgar score, head circumference, type of delivery, birth weight <2500g	315	268 (85.1%)

For peer review only

Table S1. cont.

Author (Year)	Period	Original inclusion/exclusion criteria	Country	Method to measure GWG	Intervention	Intervention Detail	Outcome	Original i-WIP N / N (%) in this analysis
Walsh *et al.* (2012)	January 2007 to January 2011	Inclusion criteria: Secundigravid women, previous macrosomic infant (≥4 kg). Exclusion criteria: underlying medical disorders, including a previous history of gestational diabetes, use of any drugs, unable to provide full informed consent, <18 years, >18 weeks' gestation, and multiple pregnancy.	Ireland	Pre-pregnancy: na. Early pregnancy: measured at 12-15 week's gestation	Diet	One dietary education session (2 hours), with the research dietitian, at 15 weeks' gestation, groups of 2-6 women. Reinforcement of diet at 28 and 34 weeks' gestation. Advised on a low glycaemic index diet, and general healthy eating guidelines for pregnancy. Received written resources about low glycaemic index diet.	Birth weight, GWG, glucose intolerance.	800 / 517 (64.6%)
Wolff *et al.* (2008)	Not described	Inclusion criteria: non-diabetic, Caucasian, BMI >30 kg/m^2, 15 weeks' gestation. Exclusion criteria: smoker, <18 or >45 years, multiple pregnancy, medical complications known to affect fetal growth adversely or to contraindicate limitation of weight gain.	Denmark	Pre-pregnancy: self-reported. Early pregnancy: measured at 15 week's gestation	Diet	Dietary consultations with a dietitian, 10 times during pregnancy (60 minutes each). Advised to eat a healthy diet according to the official Danish dietary recommendations. Aim to restrict GWG to 6–7kg.	GWG, pregnancy-induced increases in insulin, leptin, and glucose. Weight retentions at 4 weeks' postpartum	59 / 55 (93.2%)

GWG: gestational weight gain; BMI: body mass index; GDM; gestational diabetes; OGTT: oral glucose tolerance test; IOM: Institute of Medicine

For peer review only

Table S2. Global classification of risk of bias on study level for trials included

Study Team	Item 1: Randomisation	Item 2: Allocation concealment	Item 3: Blinding of participants	Item 4: Blinding of outcome Assessment	Item 5: Incomplete outcome data	Item 6: Selective reporting	Global Risk of bias
Althuizen *et al*. (2013)	+	+	?	+	+	?	Low/Medium
Baciuk *et al*. (2008)	+	+	−	+	+	+	Low/Medium
Bogaerts *et al*. (2013)	+	?	−	−	+	+	High
Haakstad *et al*. (2011)	+	+	?	+	+	−	Low/Medium
Harrison *et al*. (2013)	+	+	?	+	+	+	Low/Medium
Jeffries *et al*. (2009)	+	+	?	+	+	+	Low/Medium
Khaledan *et al*. (2010)	+	?	−	−	+	+	High
Khoury *et al*. (2005)	+	+	?	+	+	+	Low/Medium
Nascimento *et al*. (2011)	+	+	−	−	+	+	High
Oostdam *et al*. (2012)	+	+	−	+	−	+	High
Petrella *et al*. (2014)	+	−	−	−	+	−	High
Phelan *et al*. (2011)	+	+	?	+	+	+	Low/Medium
Poston *et al*. (2015)	?	?	−	−	?	?	High
Rauh *et al*. (2013)	+	+	−	−	+	?	High
Renault *et al*. (2014)	+	+	−	−	+	−	High
Sagedal *et al*. (2016)	+	+	?	+	?	?	Low/Medium
Stafne *et al*. (2012)	+	+	−	−	+	+	High
Vinter *et al*. (2011)	+	+	−	−	+	−	High
Vitolo *et al*. (2011)	+	−	−	+	+	−	High
Walsh *et al*. (2012)	+	+	−	?	+	−	Low/Medium
Wolff *et al*. (2008)	+	+	?	−	−	+	High

Legend		
+	−	?

Low	High	Unclear

For peer review only

Table S3. Individual Study Results – Gestational Weight Gain per Week

		Control			Intervention			
**Study name**	**Education**	**N**	**Mean**	**SD**	**N**	**Mean**	**SD**	***P****
Althuizen *et al.* (2013)	Lower	49	0.47	0.26	49	0.39	0.34	0.205
	Higher	49	0.43	0.27	40	0.47	0.20	0.391
Baciuk *et al.* (2008)	Lower	31	0.51	0.25	29	0.48	0.23	0.561
	Higher	4	0.52	0.31	4	0.41	0.19	0.557
Bogaerts *et al.* (2013)	Lower	40	0.41	0.25	73	0.31	0.26	0.064
	Higher	23	0.42	0.28	60	0.29	0.24	0.044
Haakstad *et al.* (2011)	Lower	6	0.39	0.18	7	0.47	0.15	0.411
	Higher	36	0.51	0.17	34	0.48	0.17	0.432
Harrison *et al.* (2013)	Lower	56	0.46	0.25	56	0.41	0.18	0.197
	Higher	35	0.47	0.26	40	0.44	0.19	0.668
Jeffries *et al.* (2009)	Lower	32	0.47	0.14	42	0.45	0.18	0.498
	Higher	79	0.46	0.16	83	0.43	0.17	0.401
Khaledan *et al.* (2010)	Lower	14	0.73	0.33	11	0.61	0.26	0.336
	Higher	6	0.51	0.17	6	0.55	0.23	0.736
Khoury *et al.* (2005)	Lower	14	0.54	0.17	21	0.56	0.20	0.822
	Higher	89	0.57	0.17	74	0.52	0.19	0.108
Nascimento *et al.* (2011)	Lower	39	0.40	0.22	34	0.37	0.22	0.562
	Higher	1	0.80	na	3	0.40	0.07	0.035
Oostdam *et al.* (2012)	Lower	24	0.32	0.20	28	0.41	0.34	0.245
	Higher	13	0.44	0.21	10	0.40	0.21	0.680
Petrella *et al.* (2014)	Lower	27	0.33	0.22	25	0.22	0.29	0.137
	Higher	1	0.35	na	5	0.34	0.12	0.950
Phelan *et al.* (2011)	Lower	36	0.46	0.19	27	0.50	0.25	0.469
	Higher	162	0.52	0.19	170	0.48	0.16	0.094
Poston *et al.* (2015)	Lower	120	0.27	0.22	113	0.26	0.20	0.853
	Higher	79	0.26	0.20	66	0.20	0.21	0.116
Rauh *et al.* (2013)	Lower	43	0.45	0.18	64	0.45	0.15	0.904
	Higher	35	0.47	0.15	89	0.43	0.13	0.120
Renault *et al.* (2014)	Lower	28	0.48	0.22	56	0.38	0.22	0.082
	Higher	100	0.39	0.19	186	0.35	0.21	0.169
Sagedal *et al.* (2017)	Lower	93	0.57	0.21	89	0.54	0.21	0.339
	Higher	195	0.52	0.19	199	0.48	0.20	0.037
Stafne *et al.* (2012)	Lower	38	0.59	0.23	31	0.53	0.15	0.178
	Higher	291	0.52	0.17	339	0.51	0.17	0.617
Vinter *et al.* (2011)	Lower	69	0.36	0.22	59	0.31	0.25	0.212
	Higher	79	0.38	0.22	85	0.33	0.20	0.158
Vitolo *et al.* (2011)	Lower	83	0.45	0.21	79	0.41	0.18	0.199
	Higher	45	0.49	0.22	61	0.42	0.15	0.053
Walsh *et al.* (2012)	Lower	62	0.52	0.21	60	0.46	0.16	0.077
	Higher	207	0.53	0.18	188	0.49	0.16	0.021
Wolff *et al.* (2008)	Lower	27	0.40	0.24	26	0.26	0.16	0.021
	Higher	2	0.38	0.24	0†	na		na

* P-value is independent-sample t-test between intervention and control
† T-test cannot be computed because at least one of the groups is empty.
Lower education: secondary education or less. Higher education: at least some tertiary education

For peer review only

Appendix 1. Identification and selection of studies in individual participant data (IPD) meta-analysis for the i-WIP Database

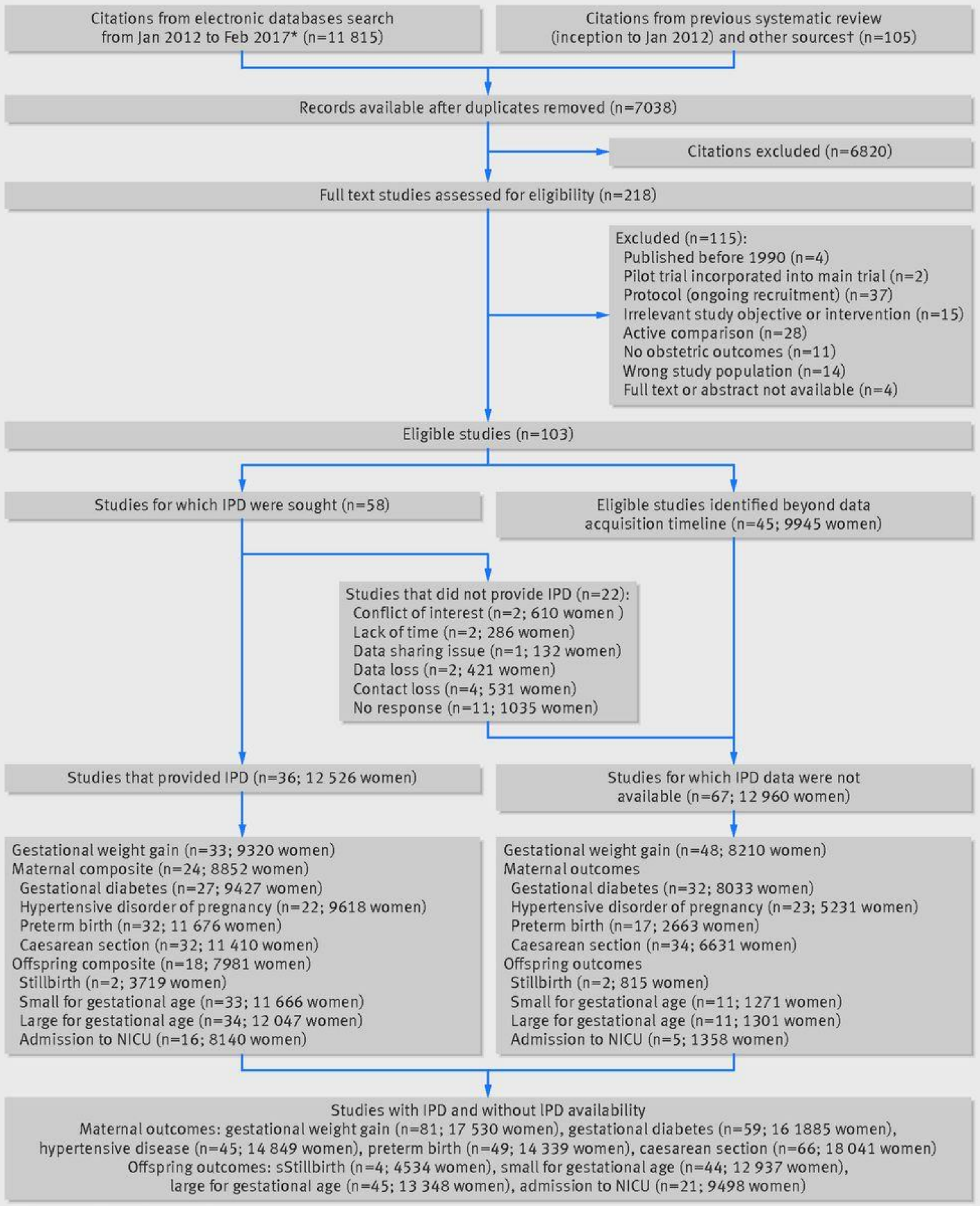

NICU=neonatal intensive care unit
*Database search was updated in October 2013 (9359 records), March 2015 (3551 records), January 2016 (1373 records), and February 2017 (1547 records)
†Other sources: reference search, personal communication, and Google search

Appendix 2. Institute of Medicine Guidelines for gestational weight gain per week in trimester 2 and 3, and extrapolated to trimester 1

Participant level data: A measured, early-pregnancy weight was available for 4,888 participants. Early-pregnancy weight was estimated using self-reported pre-pregnancy weight for the 295 participants missing this variable. Estimated weight gain per week from pre-pregnancy to early-pregnancy was calculated by the following formula, using only participants with both pre- and early-pregnancy weight, n = 3001:

$$\textit{Weight gain from pre- to early-pregnancy} = \frac{\textit{Early-pregnancy weight – pre-pregnancy weight}}{\textit{Baseline GA}}$$

Mean weight gain from pre- to early-pregnancy was 0.175 kg/week. We used the following formula to estimate early-pregnancy weight for the 295 participants missing this variable:

$$\text{Early-pregnancy weight} = (\text{Baseline GA}*0.175\text{ kg}) + \text{pre-pregnancy weight}$$

We used pre-pregnancy weight to categorise participants according to BMI. Self-reported pre-pregnancy weight was available for 3,296 participants, and estimated using early-pregnancy weight for the remaining 1,887 participants. We used the following formula to estimate pre-pregnancy weight:

$$\text{Pre-pregnancy weight} = \text{Early-pregnancy weight} - (\text{Baseline GA}*0.175\text{ kg})$$

Total weight gain was calculated by subtracting early-pregnancy weight from final follow-up weight. Weekly GWG was calculated using the following formula:

$$\textit{Weight Gain per week} = \frac{\textit{Total GWG}}{\textit{Final follow-up GA – Baseline GA}}$$

Trimester 2 and 3 GWG/week was required for comparison with IOM 2009 guidelines. This was calculated using a four-step approach;

1. Trimester 1 GWG/week guidelines were extrapolated from the IOM guidelines(15) .

For peer review only

BMI Category	Total GWG (IOM Guidelines)			Rate of weight gain for Trimester 2 and 3 (IOM Guidelines)		
	Inadequate	Adequate	Excess	Mean	Range	
**Underweight**	<12.5	12.5 - 18.0	>18.0	0.51	0.44	0.58
**Normal**	<11.5	11.5 - 16.0	>16.0	0.42	0.35	0.50
**Overweight**	<7.0	7.0 - 11.5	>11.5	0.28	0.23	0.33
**Obese**	<5.0	5.0 - 9.0	>9.0	0.22	0.17	0.27

For peer review only

2. The number of weeks in trimester 1 that the participant was enrolled in the intervention was calculated by subtracting the baseline GA from 13. If the participant enrolled in the study at greater than 13 weeks, trimester 1 weeks were set as 0. The number of weeks in trimester 2 and 3 that the participant was enrolled in the intervention were calculated by subtracting the 13 from the final follow-up GA.

3. The rate of weight gain for Trimester 1 was calculated:

   (*Rate of weight gain for Trimester 2 and 3*)**27= Total weight gain for Trimester 2 and 3* ***(A)***

   Total GWG [ranges for adequate] - Total weight gain for Trimester 2 and 3 [ranges] = Total weight gain for Trimester 1 [ranges] ***(B)***

$$\frac{\textit{Lower range Trimester 1 weight gain} + \textit{Upper range Trimester 1 weight gain}}{2} = \textit{Mean weight gain for Trimester 1}\ \textbf{(C)}$$

$$\frac{\textit{Total weight gain for Trimester 1 [mean and ranges]}}{13} = \textit{Rate of weight gain for Trimester 1}\ \textbf{(D)}$$

BMI Category	(A) Total weight gain for Trimester 2 and 3			Total weight gain for Trimester 1			(D) Rate of weight gain for Trimester 1		
	Mean	Range		(C) Mean	(B) Range		Mean	Range	
**Underweight**	13.77	11.88	15.66	1.48	0.62	2.34	0.11	0.05	0.18
**Normal**	11.34	9.45	13.50	2.28	2.05	2.50	0.18	0.16	0.19
**Overweight**	7.56	6.21	8.91	1.69	0.79	2.59	0.13	0.06	0.20
**Obese**	5.94	4.59	7.29	1.06	0.41	1.71	0.08	0.03	0.13

4. The contribution of trimester 1 weight gain to total GWG was estimated using the mean trimester 1 GWG/week (above) and the following equation:

$$\text{T1 weight gain} = (\text{T1 weight gain per week for BMI}) * (\text{T1 weeks in intervention})$$

5. Trimester 2 and 3 weight gain per week was calculated using the following equation:

$$\textit{T2 and T3 weight gain per week} = \frac{\textit{Total weight gain} - \textit{T1 weight gain}}{\textit{Weeks in T2 and T3}}$$

For peer review only

S2 Appendix. PRISMA-IPD Checklist of items to include when reporting a systematic review and meta-analysis of individual participant data (IPD)

PRISMA-IPD Section/topic	Item No	Checklist item	Reported on page
Title			
Title	1	Identify the report as a systematic review and meta-analysis of individual participant data.	1
Abstract			
Structured summary	2	Provide a structured summary including as applicable:	5-6
		**Background**: state research question and main objectives, with information on participants, interventions, comparators and outcomes.	
		**Methods**: report eligibility criteria; data sources including dates of last bibliographic search or elicitation, noting that IPD were sought; methods of assessing risk of bias.	
		**Results**: provide number and type of studies and participants identified and number (%) obtained; summary effect estimates for main outcomes (benefits and harms) with confidence intervals and measures of statistical heterogeneity. Describe the direction and size of summary effects in terms meaningful to those who would put findings into practice.	
		**Discussion:** state main strengths and limitations of the evidence, general interpretation of the results and any important implications.	
		**Other:** report primary funding source, registration number and registry name for the systematic review and IPD meta-analysis.	

For peer review only

Introduction			
Rationale	3	Describe the rationale for the review in the context of what is already known.	8-9
Objectives	4	Provide an explicit statement of the questions being addressed with reference, as applicable, to participants, interventions, comparisons, outcomes and study design (PICOS). Include any hypotheses that relate to particular types of participant-level subgroups.	9
Methods			
Protocol and registration	5	Indicate if a protocol exists and where it can be accessed. If available, provide registration information including registration number and registry name. Provide publication details, if applicable.	9
Eligibility criteria	6	Specify inclusion and exclusion criteria including those relating to participants, interventions, comparisons, outcomes, study design and characteristics (e.g. years when conducted, required minimum follow-up). Note whether these were applied at the study or individual level i.e. whether eligible participants were included (and ineligible participants excluded) from a study that included a wider population than specified by the review inclusion criteria. The rationale for criteria should be stated.	9
Identifying studies - information sources	7	Describe all methods of identifying published and unpublished studies including, as applicable: which bibliographic databases were searched with dates of coverage; details of any hand searching including of conference proceedings; use of study registers and agency or company databases; contact with the original research team and experts in the field; open adverts and surveys. Give the date of last search or elicitation.	9 and Protocol paper attached
Identifying studies - search	8	Present the full electronic search strategy for at least one database, including any limits used, such that it could be repeated.	9 and Protocol paper attached

For peer review only

Study selection processes	9	State the process for determining which studies were eligible for inclusion.	9 and Protocol paper attached
Data collection processes	10	Describe how IPD were requested, collected and managed, including any processes for querying and confirming data with investigators. If IPD were not sought from any eligible study, the reason for this should be stated (for each such study).<br><br>If applicable, describe how any studies for which IPD were not available were dealt with. This should include whether, how and what aggregate data were sought or extracted from study reports and publications (such as extracting data independently in duplicate) and any processes for obtaining and confirming these data with investigators.	9 and Protocol paper attached
Data items	11	Describe how the information and variables to be collected were chosen. List and define all study level and participant level data that were sought, including baseline and follow-up information. If applicable, describe methods of standardising or translating variables within the IPD datasets to ensure common scales or measurements across studies.	10
IPD integrity	A1	Describe what aspects of IPD were subject to data checking (such as sequence generation, data consistency and completeness, baseline imbalance) and how this was done.	10
Risk of bias assessment in individual studies.	12	Describe methods used to assess risk of bias in the individual studies and whether this was applied separately for each outcome. If applicable, describe how findings of IPD checking were used to inform the assessment. Report if and how risk of bias assessment was used in any data synthesis.	10
Specification of outcomes	13	State all treatment comparisons of interests. State all outcomes addressed and define them in detail. State whether they were pre-specified for the review and, if applicable, whether they were primary/main or secondary/additional	10-11

For peer review only

and effect measures		outcomes. Give the principal measures of effect (such as risk ratio, hazard ratio, difference in means) used for each outcome.	
Synthesis methods	14	Describe the meta-analysis methods used to synthesise IPD. Specify any statistical methods and models used. Issues should include (but are not restricted to):<br>• Use of a one-stage or two-stage approach.<br>• How effect estimates were generated separately within each study and combined across studies (where applicable).<br>• Specification of one-stage models (where applicable) including how clustering of patients within studies was accounted for.<br>• Use of fixed or random effects models and any other model assumptions, such as proportional hazards.<br>• How (summary) survival curves were generated (where applicable).<br>• Methods for quantifying statistical heterogeneity (such as I^2 and τ^2).<br>• How studies providing IPD and not providing IPD were analysed together (where applicable).<br>• How missing data within the IPD were dealt with (where applicable).	11-12
Exploration of variation in effects	A2	If applicable, describe any methods used to explore variation in effects by study or participant level characteristics (such as estimation of interactions between effect and covariates). State all participant-level characteristics that were analysed as potential effect modifiers, and whether these were pre-specified.	12
Risk of bias across studies	15	Specify any assessment of risk of bias relating to the accumulated body of evidence, including any pertaining to not obtaining IPD for particular studies, outcomes or other variables.	10
Additional analyses	16	Describe methods of any additional analyses, including sensitivity analyses. State which of these were pre-specified.	12
Results			
Study	17	Give numbers of studies screened, assessed for eligibility, and included in the systematic review with reasons for	13 and Table 1

For peer review only

selection and IPD obtained		exclusions at each stage. Indicate the number of studies and participants for which IPD were sought and for which IPD were obtained. For those studies where IPD were not available, give the numbers of studies and participants for which aggregate data were available. Report reasons for non-availability of IPD. Include a flow diagram.	
Study characteristics	18	For each study, present information on key study and participant characteristics (such as description of interventions, numbers of participants, demographic data, unavailability of outcomes, funding source, and if applicable duration of follow-up). Provide (main) citations for each study. Where applicable, also report similar study characteristics for any studies not providing IPD.	13 and Table S1
IPD integrity	A3	Report any important issues identified in checking IPD or state that there were none.	13 and Table S2
Risk of bias within studies	19	Present data on risk of bias assessments. If applicable, describe whether data checking led to the up-weighting or down-weighting of these assessments. Consider how any potential bias impacts on the robustness of meta-analysis conclusions.	13 and Table S2
Results of individual studies	20	For each comparison and for each main outcome (benefit or harm), for each individual study report the number of eligible participants for which data were obtained and show simple summary data for each intervention group (including, where applicable, the number of events), effect estimates and confidence intervals. These may be tabulated or included on a forest plot.	14 and Table S3
Results of syntheses	21	Present summary effects for each meta-analysis undertaken, including confidence intervals and measures of statistical heterogeneity. State whether the analysis was pre-specified, and report the numbers of studies and participants and, where applicable, the number of events on which it is based.	14-15. Tables 2-5
		When exploring variation in effects due to patient or study characteristics, present summary interaction estimates for each characteristic examined, including confidence intervals and measures of statistical heterogeneity. State whether	

For peer review only

		the analysis was pre-specified. State whether any interaction is consistent across trials.	
		Provide a description of the direction and size of effect in terms meaningful to those who would put findings into practice.	
Risk of bias across studies	22	Present results of any assessment of risk of bias relating to the accumulated body of evidence, including any pertaining to the availability and representativeness of available studies, outcomes or other variables.	13
Additional analyses	23	Give results of any additional analyses (e.g. sensitivity analyses). If applicable, this should also include any analyses that incorporate aggregate data for studies that do not have IPD. If applicable, summarise the main meta-analysis results following the inclusion or exclusion of studies for which IPD were not available.	15
Discussion			
Summary of evidence	24	Summarise the main findings, including the strength of evidence for each main outcome.	16
Strengths and limitations	25	Discuss any important strengths and limitations of the evidence including the benefits of access to IPD and any limitations arising from IPD that were not available.	18-19
Conclusions	26	Provide a general interpretation of the findings in the context of other evidence.	19
Implications	A4	Consider relevance to key groups (such as policy makers, service providers and service users). Consider implications for future research.	17-18
Funding			

For peer review only

Funding	27	Describe sources of funding and other support (such as supply of IPD), and the role in the systematic review of those providing such support.	6

For peer review only

For peer review only

BMJ Open

Impact of maternal education on response to lifestyle interventions to reduce gestational weight gain - Individual participant data meta-analysis

Journal:	*BMJ Open*
Manuscript ID	bmjopen-2018-025620.R1
Article Type:	Research
Date Submitted by the Author:	11-Mar-2019

Complete List of Authors:	O'Brien, Eileen; University College Dublin, UCD Perinatal Research Centre<br>Segurado, Ricardo; University College Dublin, CSTAR, School of Public Health, Physiotherapy and Population Science<br>Geraghty, Aisling; University College Dublin, UCD Perinatal Research Centre<br>Alberdi , Goiuri; University College Dublin, UCD Perinatal Research Centre<br>Rogozinska, Ewelina; Queen Mary's University London, Women's Health Research Unit<br>Astrup, Arne; Univesity of Copenhagen, Department of Nutrition, Exercise and Sports,<br>Barakat Carballo , Rubenomar; Universidad Politecnica de Madrid, Facultad de Ciencias de la Actividad Fısica y del Deporte<br>Bogaerts, Annick; University of Leuven, Department of Development and Regeneration KU Leuven; UC Leuven-Limburg, Faculty of Health and Social Work<br>Cecatti, Jose; University of Campinas, Obstetrics and Gynecology<br>Coomarasamy, Arri; University of Birmingham,<br>de Groot, Christianne; VU University Medical Center, Obstetrics and Gynaecology<br>Devlieger, R.; Universitaire Ziekenhuizen Leuven, Department of Obstetrics and Gynecology; KUL University of Leuven, Department of Development and Regeneration<br>Dodd, Jodie; The Unversity of Adelaide, Obstetrics and Gynaecology; Discipline of Obstetrics and Gynaecology<br>El Beltagy, Nermeen ; Alexandria University, Department of Obstetrics and Gynecology<br>Facchinetti , Fabio ; Azienda Ospedaliera Universitaria Policlinico di Modena, Obstetric Unit, Mother Infant Department<br>Geiker , Nina ; Rigshospitalet, Clinical Nutrition Research<br>Guelfi, Kym; The University of Western Australia, School of Human Sciences<br>Haakstad, Lene; Norges idrettshogskole, Department of Sports Medicine<br>Harrison, Cheryce; Monash University School of Public Health and Preventive Medicine, Monash Centre for Health Research and Implementation<br>Hauner, Hans; Technische Universitat Munchen Zentralinstitut fur Ernahrungs- und Lebensmittelforschung, Else Kroener-Fresenius-Center

	for Nutritional Medicine<br>Jensen, DM; Syddansk Universitet Det Sundhedsvidenskabelige Fakultet, Department of Endocrinology<br>Khan, Khalid; Queen Mary, University of London, Multidisciplinary Evidence Synthesis Hub (mEsh); Queen Mary University of London, Women's Health Research Unit<br>Kinnunen, Tarja; University of Tampere, School of Health Sciences<br>Luoto, Riitta; Tampereen Yliopisto, Health Sciences<br>Willem Mol, Ben; University of Adelaide, Obstetrics/Gynaecology<br>Mørkved, Siv; NTNU Fakultet for ingeniorvitenskap og teknologi Trondheim, Department of Public Health and General Practice; Trondheim University Hospital, Clinical Services<br>Motahari-Tabari, Narges ; University of Mazandaran, Department of Sport Physiology<br>Owens, Julie; The University of Adelaide, Discipline of Obstetrics & Gynaecology<br>Perales, Maria; Universidad Politecnica de Madrid, Facultad de Ciencias de la Actividad Fısica y del Deporte<br>Petrella, Elisabetta; Universita degli Studi di Modena e Reggio Emilia Dipartimento di Educazione e Scienze Umane, Mother-Infant Department<br>Phelan, Suzanne; California Polytechnic State University College of Science and Mathematics, Kinesiology Department<br>Poston, Lucilla; Kings College, London, Division of Reproduction and Endocrinology<br>Rauh, Kathrin; Technische Universitat Munchen Zentralinstitut fur Ernahrungs- und Lebensmittelforschung, Else Kroener-Fresenius-Center for Nutritional Medicine; Competence Centre for Nutrition (KErn)<br>Rayanagoudar, Girish ; Queen Mary University of London, Women's Health Research Unit<br>Renault , KM ; University of Copenhagen, Departments of Obstetrics and Gynecology<br>Ruifrok, A; VU medisch centrum ICaR-VU Onderzoeksinstituut, Department of Obstetrics and Gynaecology; Academic Medical Centre, Department of Obstetrics and Gynecology<br>Sagedal, Linda; Sorlandet sykehus HF, Department of Obstetrics and Gynecology<br>Salvesen, Kjell; Trondheim University Hospital, Dept of Obstetrics and gynaecology; Norwegian University of Science and Technology, Department of Laboratory Medicine Children's and Women's Health<br>Scudeller, Tania; Universidade Federal de Sao Paulo, Department of Management and Health Care<br>Shen, Gary; University of Manitoba College of Medicine, Department of Internal Medicine<br>Shub, Alexis; University of Melbourne, Obstetrics and Gynaecology<br>Stafne, Signe; NTNU Fakultet for ingeniorvitenskap og teknologi Trondheim, Department of Public Health and General Practice; Sankt Olavs Hospital Universitetssykehuset i Trondheim, Clinical Services<br>Surita, Fernanda; University of Campinas, Obstetrics & Gynecology<br>Thangaratinam, Shakila; Queen Mary University of London, Women's Health Research Unit; Queen Mary University of London, Multidisciplinary Evidence Synthesis Hub (mEsh)<br>Tonstad, Serena ; Oslo universitetssykehus Ulleval, Department of Obstetrics and Gynecology<br>van Poppel, Mireille; VU University Medical Center, Department of Public and Occupational Health<br>Vinter, Christina ; Syddansk Universitet Det Sundhedsvidenskabelige Fakultet, Department of Obstetrics and Gynecology<br>Vistad, Ingvild ; Sorlandet Hospital Kristiansand , Obstetric and Gynegology<br>Yeo, SeonAe ; University of North Carolina at Chapel Hill, School of Nursing

	McAuliffe, Fionnuala; University College Dublin, UCD Perinatal Research Centre
**Primary Subject Heading**:	Nutrition and metabolism
Secondary Subject Heading:	Epidemiology, Nutrition and metabolism, Public health
Keywords:	Pregnancy, Lifestyle interventions, Socioeconomic status, Inequalities, Nutrition < TROPICAL MEDICINE

SCHOLARONE™
Manuscripts

Title: Impact of maternal education on response to lifestyle interventions to reduce gestational weight gain - Individual participant data meta-analysis

Author List:

E.C. O'Brien [1], R. Segurado [2], A.A. Geraghty [1], G. Alberdi [1], E. Rogozińska [3, 4], A. Astrup [5], R. Barakat Carballo [6], A. Bogaerts [7, 8, 9], J.G. Cecatti [10], A. Coomarasamy [11], C.J. de Groot [12], R. Devlieger [13, 14], J.Dodd [15], N.El Beltagy [16], F. Facchinetti [17], N.R. Geiker [18], K.J Guelfi [19], L. Haakstad [20], C.Harrison [21], H. Hauner [22], D.M. Jensen [23], K.S. Khan [4,26], T. I. Kinnunen [27], R. Luoto [28], B. W. Mol [29], S. Mørkved [30, 31], N. Motahari-Tabari [32], J. Owens [15], M. Perales, [6], E. Petrella [17], S. Phelan [33], L. Poston [34], K. Rauh [35,36], G. Rayanagoudar [26], K.M. Renault [37, 38], A.E. Ruifrok [12,39], L.R. Sagedal [40], K.Å Salvesen [41,42], T.T. Scudeller [43], G.X. Shen [44], A. Shub [45], S.N. Stafne [30, 31], F. Surita [10], S. Thangaratinam [4,26], S. Tonstad [46], M.N. van Poppel [47], C. Vinter [24], I. Vistad [40], S. Yeo [49], i-WIP (International Weight Management in Pregnancy) Collaborative Group, F.M. McAuliffe [1]*

Affiliations

1 UCD Perinatal Research Centre, Obstetrics & Gynaecology, UCD School of Medicine, University College Dublin, National Maternity Hospital, Dublin, Ireland

2 Centre for Support and Training in Analysis and Research (CSTAR), School of Public Health, Physiotherapy and Sports Science, University College Dublin, Dublin, Ireland

3 Women's Health Research Unit, Barts and The London School of Medicine and Dentistry, Queen Mary University of London, London, UK..

4 Multidisciplinary Evidence Synthesis Hub (mEsh), Barts and The London School of Medicine and Dentistry, Queen Mary University of London, London, UK.

5 Department of Nutrition, Exercise and Sports, Copenhagen University, Copenhagen, Copenhagen, Denmark.

6 Facultad de Ciencias de la Actividad Fısica y del Deporte-INEF, Universidad

For peer review only - http://bmjopen.bmj.com/site/about/guidelines.xhtml

Politecnica de Madrid, Madrid, Spain.

7 Department of Development and Regeneration KU Leuven, University of Leuven, Leuven, Belgium,

8 Faculty of Health and Social Work, research unit Healthy Living, UC Leuven-Limburg, Belgium

9 Faculty of Medicine and Health Sciences, Centre for Research and Innovation in Care (CRIC), University of Antwerp, Belgium.

10 Department of Obstetrics and Gynecology, School of Medical Sciences, University of Campinas (UNICAMP), Campinas, Brazil.

11 School of Clinical and Experimental Medicine, College of Medical and Dental Sciences, University of Birmingham, Birmingham, UK.

12 Department of Obstetrics and Gynaecology, Faculty of Medicine, VU University Medical Center, Amsterdam, The Netherlands.

13 Department of Obstetrics and Gynecology, University Hospitals Leuven, Belgium

14 Department of Development and Regeneration, KUL University of Leuven, Belgium

15 Discipline of Obstetrics and Gynaecology, School of Paediatrics and Reproductive Health, The University of Adelaide, Adelaide, Australia.

16 Department of Obstetrics and Gynecology, Alexandria University, Alexandria, Egypt.

17 Mother-Infant Department, University of Modena and Reggio Emilia, Modena, Italy.

18 Clinical Nutritional Research Unit, Copenhagen University Hospital Herlev-Gentofte, Gentofte, Denmark.

19 School of Human Sciences, The University of Western Australia, Perth, Australia.

20 Norwegian School of Sport Sciences, Department of Sports Medicine, Oslo, Norway.

21 Monash Centre for Health Research and Implementation -MCHRI, School of Public Health Monash University, Melbourne, Australia.

22 Else Kroener-Fresenius-Center for Nutritional Medicine, Klinikum rechts der Isar, Technische Universität München, Munich, Germany.

For peer review only

For peer review only - http://bmjopen.bmj.com/site/about/guidelines.xhtml

23 Department of Endocrinology, Odense University Hospital, University of Southern Denmark, Odense, Denmark.

24 Department of Gynecology and Obstetrics, Odense University Hospital, University of Southern Denmark, Odense, Denmark.

25 Department of Clinical Research, Faculty of Health Sciences, University of Southern Denmark

26 Women's Health Research Unit, Barts and The London School of Medicine and Dentistry, Queen Mary University of London, London, UK.

27 Faculty of Social Sciences/ Health Sciences, University of Tampere, Tampere, Finland.

28 School of Health Sciences, University of Tampere, Tampere, Finland.

29 Robinson Institute, School of Paediatrics and Reproductive Health, University of Adelaide, Adelaide, Australia.

30 Department of Public Health and General Practice, Faculty of Medicine, Norwegian University of Science and Technology, Trondheim, Norway.

31 Clinical Services, St. Olavs Hospital, Trondheim University Hospital, Trondheim, Norway.

32 Department of Sport Physiology, Faculty of Physical Education and Sport Sciences, Mazandaran University, Babolsar, Iran.

33 Kinesiology Department, California Polytechnic State University, San Luis Obispo, USA.

34 Division of Women's Health, Women's Health Academic Centre, King's College London, St. Thomas' Hospital, London, UK.

35 Else Kroener-Fresenius-Center for Nutritional Medicine, Chair of Nutritional Medicine, Technische Universität München, Munich, Germany.

36 Competence Centre for Nutrition (KErn), Freising, Germany.

37 Departments of Obstetrics and Gynecology, Hvidovre Hospital, University of

For peer review only

Copenhagen, Copenhagen, Denmark.

38 Obstetric Clinic, Juliane Marie Centret, Rigshospitalet, University of Copenhagen, Copenhagen, Denmark.

39 Department of Obstetrics and Gynecology, Academic Medical Centre, Amsterdam, The Netherlands.

40 Department of Obstetrics and Gynecology, Sorlandet Hospital, Kristiansand, Norway.

41 Department of Obstetrics and Gynaecology, St. Olavs Hospital, Trondheim University Hospital, Trondheim, Norway

42 Department of Laboratory Medicine Children's and Women's Health, Faculty of Medicine, Norwegian University of Science and Technology, Trondheim, Norway.

43 Department of Management and Health Care, Physical Therapy Course, Federal University of São Paulo (UNIFESP), Brazil

44 Department of Internal Medicine, University of Manitoba, Winnipeg, Canada.

45 Department of Obstetrics and Gynaecology, University of Melbourne, Melbourne, Australia.

46 Department of Obstetrics and Gynecology, Oslo University Hospital, Oslo, Norway.

47 Department of Public and Occupational Health, Amsterdam Public Health research institute, VU University Medical Center, Amsterdam, The Netherlands.

Institute of Sport Science, University of Graz, Austria.

48 University of North Carolina at Chapel Hill, School of Nursing, Chapel Hill, NC, USA.

Corresponding author:

Fionnuala M McAuliffe; Email: fionnuala.mcauliffe@ucd.ie

Key Words: Pregnancy; Lifestyle interventions; socioeconomic status; inequalities; nutrition

Word count: 3,737

For peer review only - http://bmjopen.bmj.com/site/about/guidelines.xhtml

Abstract

Objectives: To identify if maternal educational attainment is a prognostic factor for gestational weight gain (GWG), and to determine the differential effects of lifestyle interventions (diet-alone, exercise-alone or mixed approach) on gestational weight gain, stratified by educational attainment.

Design: Individual participant data meta-analysis using the previously established International Weight Management in Pregnancy (i-WIP) Collaborative Group database (https://iwipgroup.wixsite.com/collaboration). PRISMA-IPD Statement guidelines were followed.

Data sources: Major electronic databases, from inception to February 2017.

Eligibility criteria: Randomised controlled trials on diet and physical activity based interventions in pregnancy. Maternal educational attainment was required for inclusion and was categorised as higher education (≥ tertiary) or lower education (≤ secondary).

Risk of bias: Cochrane risk of bias tool was used.

Data synthesis: Principle measures of effect were odds ratio and regression coefficient.

Results: From the 36 randomised controlled trials in the i-WIP database, 21 trials and 5,183 pregnant women were included. Women of lower educational attainment had an increased risk of excessive (OR:1.182; 95% CI:1.008-1.385; P:0.039) and inadequate weight gain (OR:1.284; 95% CI:1.045-1.577; *P*: 0.017). Among women with lower education, diet-alone interventions reduced risk of excessive weight gain (OR:0.515; 95% CI:0.339-0.785; *P*:0.002) and inadequate weight gain (OR:0.504; 95% CI:0.288-0.884; *P*:0.017), and reduced kg/week gain (*B*:-0.055; 95% CI:-0.098–-0.012; *P*:0.012). Mixed interventions reduced risk of excessive weight gain for women with lower education (OR:0.735; 95% CI:0.561-0.963; *P*:0.026). Among women with high education, diet-alone interventions reduced risk of excessive weight gain (OR:0.609; 95% CI:0.437-0.849; *P*:0.003), and mixed interventions reduced kg/week gain (*B*:-0.053; 95% CI:-0.069–-0.037; *P*:<0.001). Exercise-alone interventions did not impact GWG in the total cohort, nor stratified by education.

For peer review only - http://bmjopen.bmj.com/site/about/guidelines.xhtml

Conclusions: Pregnant women with lower education are at an increased risk of excessive and inadequate GWG. Diet-alone interventions seem the most appropriate choice for these women, and additional support through mixed interventions may also be beneficial.

Article Summary

Strengths and limitations of this study

- A major strength is the use of individual patient data to carry out meta-analysis from multiple nutrition and lifestyle interventions.
- The pooling of individual patient data allows for the estimation of the differential effects of education and lifestyle interventions on weight gain across the included cohorts with greater power than individual randomised controlled trials.
- We analysed and reported the findings using gestational weight gain per week, which enables comparison of studies that use different gestational ages for measuring gestational weight gain.
- A limitation is that most of the cohorts included are from wealthy countries; therefore, the results may not be representative of populations living in low and middle-income countries.
- Although the results were statistically significant, the effect sizes are small, thus the relevance of these findings in the clinical setting must be considered.

For peer review only

For peer review only - http://bmjopen.bmj.com/site/about/guidelines.xhtml

Introduction

According to the 2030 Sustainable Development Goals, the global health agenda is calling for improvements in maternal health and nutrition, equal opportunities for education, and reduced social, economic and political inequalities (1). Currently, however, maternal social, economic and educational backgrounds remain to be important determinants of health and lifestyle behaviours among pregnant women. Pregnancy and birth outcomes are negatively influenced by socioeconomic and deprivation factors (2–6). Prevalence of smoking, alcohol consumption and pre-pregnancy body mass index (BMI) are greater among women of lower socioeconomic status, while diet quality and physical activity participation are reduced within this group (7–9).

GWG is also influenced by socioeconomic status, with a recent systematic review finding that those of lower educational attainment are less likely to gain weight within the guidelines' recommended ranges for GWG, as described by the Institute of Medicine (IOM) (10). Excessive GWG increases the risk of adverse maternal and neonatal outcomes, including gestational diabetes, caesarean section, hypertension, large-for-gestational-age and stillbirth, while inadequate GWG is associated with increased risk of small-for-gestational-age infant (11,12). Moreover, greater GWG is associated with weight retention postpartum (13) and elevated BMI up to 15 years post-pregnancy (14), a potential factor exacerbating current global trends of overweight and obesity (15).

Since GWG outside of suggested guidelines is more prevalent among disadvantaged groups, it would seem wise that support and interventions which enable equal opportunities to optimise health are prioritised. However, there remains limited knowledge regarding GWG among women of various socioeconomic position, as recognised by the IOM (16). Pregnancy has been described as a stimulus for positive behaviour change (17) and is a time when women are in regular contact with healthcare professionals (18). Thus, a lifestyle intervention in pregnancy should, in theory, have a greater impact during this time than any other time in a woman's life.

For peer review only - http://bmjopen.bmj.com/site/about/guidelines.xhtml

There are many examples of lifestyle and behaviour interventions in the literature that aim to manage weight gain during pregnancy (19); however, few interventions stratify analyses by socioeconomic status. The International Weight Management in Pregnancy (i-WIP) Individual Patient Data Collaborative Network was established to determine the differential effects of weight management interventions in pregnancy on maternal weight gain and pregnancy outcomes (20), and found that diet and physical activity based interventions reduce GWG and lower the odds of caesarean section (21). However, sub-analysis was not conducted among women of various educational attainment, thus compounding our lack of understanding for the type of pregnancy intervention that has the greatest effect on those of low educational attainment.

This research will conduct IPD meta-analysis using the previously established i-WIP dataset to: (1) identify if educational attainment is a prognostic factor for GWG; (2) determine the differential effects of weight management interventions in pregnancy on maternal weight gain stratified by educational attainment.

Methods

The checklist outlined in the Preferred Reporting Items for Systematic reviews and Meta-Analysis of Individual Participant Data (PRISMA-IPD) statement was followed (22). Full information about the search strategy is available in the i-WIP protocol paper and the i-WIP primary publication (20,21). In brief, major electronic databases from October 2013 to March 2015 were searched, with updated literature searches completed by the i-WIP team in February 2017 and more recently in June 2018. Given the logistics and resources involved in collating IPD, trials identified in the most recent literature search have not yet been added to the i-WIP dataset. Eligible studies were randomised controlled trials (RCT) that evaluated any dietary or lifestyle interventions with potential to influence maternal and fetal outcomes related to maternal weight (20,23). The women in the study were required to be ≥18 years of age with a singleton pregnancy and they must have been enrolled to one of the following

For peer review only

For peer review only - http://bmjopen.bmj.com/site/about/guidelines.xhtml

interventions; diet based, physical activity based, mixed approach (diet, physical activity, GWG monitoring and/or behaviour change components), or standard care (23). In total, 11,920 studies were identified, of which 36 studies were included (12,526 participants) (20,21,24). The flow-chart for the identification and selection of studies has been published (21) (Appendix 1). A full search strategy from the original i-WIP IPD for one database is presented in Appendix 2. All individual trials included in this IPD meta-analysis had ethical approval from relevant ethical committees.

IPD integrity and risk of bias assessment

The i-WIP Network performed a range of checks on the variables used during the analysis and produced summary tables with a focus on checking the randomisation ratio, baseline characteristics and the method of analysis in the IPD data set with the published information. The i-WIP Network discussed major discrepancies with the trial team. As per the protocol, the i-WIP Network assessed the quality of each trial to evaluate the integrity of the randomisation and follow-up procedure. The Cochrane risk of bias tool was used and studies were classified as high risk of bias if they scored highly in at least one of following domains: randomisation, allocation concealment, blinding of outcome assessment, or incomplete outcome data. To be classified as low risk of bias, all items must have scored as low risk. In terms of publication bias, a funnel plot was created to assess the small study effects on trials in the IPD meta-analysis of educational attainment and gestational weight gain per week. (Figure S1).

Data items and specification of outcomes

The i-WIP protocol pre-specified that socioeconomic status would be evaluated as a potential prognostic variable for GWG, as secondary analysis to the i-WIP project (20). The exposure variable was maternal educational attainment, as this was the most commonly used measure of socioeconomic status among studies in the i-WIP network. It has also been reported as a stable measure of individual socioeconomic status (25). Educational

For peer review only

attainment was classified as less than or equal to secondary education (including no education, less than primary, completed primary, some secondary, or completed secondary education), or tertiary education (including vocational training, some university, bachelor degree, post-graduate degree or higher).

Intervention type was classified as diet based, physical activity based, or mixed approach (diet, physical activity, GWG monitoring and/or behaviour change components) (23), and participants were either exposed to the intervention or to standard care.

The primary outcome was maternal weight gain in pregnancy. The outcome was examined using two methods: according to the IOM 2009 guidelines (16); and GWG per week as a continuous variable. To calculate GWG, data were required for the following variables; baseline gestational age, final follow-up gestational age, pre-pregnancy or early-pregnancy weight, final follow-up weight and height. The method described in Appendix 3 was used to calculate GWG per week.

Specification of effect measures

The principle measures of effect used were odds ratio (for analysis according to the IOM guidelines) and regression coefficient (for analysis of GWG as a continuous variable).

To assess the magnitude of study variability in the outcomes, we calculated an intra-class correlation coefficient (ICC) from the fitted models, interpretable as an index of heterogeneity such as the I^2. The intra-class correlation coefficient for the study (ICC_{study}) relates to the proportion of variability due to cohort-to-cohort variation, and the intra-class correlation coefficient for the intervention ($ICC_{intervention}$) relates to the proportion of variability in the intervention treatment effect. In the logistic models for categorical GWG the residual variance was replaced by $\pi^2/3$.

Synthesis methods

For comparisons of characteristics across education groups, we used independent sample *t* tests for continuous data and χ^2 tests for categorical data. For the categorical outcomes of

For peer review only - http://bmjopen.bmj.com/site/about/guidelines.xhtml

inadequate, adequate, and excessive GWG, we used generalised linear mixed models: a multinomial logistic regression with a generalised logit link function. For the continuous outcomes of GWG (kg/week), we used a linear mixed model with an identity link function, assuming the outcome was approximately normally distributed. The models analysing the effect of education included random effects for each cohort. Random effects were specified for both the intercept and the slope of the intervention effect, allowing them to be correlated. The between-study variance in each effect was calculated by the ICC. Separate models were created for educational attainment strata to analyse the effect of each intervention type, as fixed effects. Linear mixed models were adjusted for factors known to influence GWG (BMI, maternal age, parity, ethnicity), in addition to the main effects and interaction of education and the interventions. In model 1 we adjusted for pre-pregnancy maternal BMI (continuous GWG model only). In model 2 we adjusted for pre-pregnancy maternal BMI (continuous GWG model only), maternal age, parity and ethnicity. As the IOM category definitions are linked to pre-pregnancy BMI category, this outcome intrinsically "adjusts" for maternal BMI; we can consider model 1 as "unadjusted", and model 2 as an "adjusted" model. All statistical analyses were performed using IBM SPSS Statistics for Windows, Version 20.0 (IBM, Armonk, NY).

For peer review only

Exploration of variation in effects

While controlling for between-study variation in outcomes and intervention effects as random effects, we explored the influence of educational attainment on the intervention effects as pre-specified fixed main effects and an interaction term. Tertiary education and the control (non-intervention) condition were set as the reference levels, and results for IOM categories used the adequate weight gain category as the reference outcome group.

Additional analyses

We proceeded with our model-building in a step-wise fashion, aiming at a pre-specified target of fixed effects as above, and both random intercept and intervention effects. A step-

For peer review only - http://bmjopen.bmj.com/site/about/guidelines.xhtml

wise approach is often pragmatic in developing a mixed-effects model as small study size and uncertain variance/covariance structure can lead to models failing to converge. We therefore combined the addition of random and fixed effects to a model in the following stages: 1) empty model including no fixed predictors, only a random intercept; 2) education and intervention main effects and interaction added to the model, and controlling for pre-pregnancy maternal BMI (Model 1); 3) Random effect of intervention was explored to account for potential population or intrinsic variability in the effect of the interventions on GWG; 4) additional potential confounders were added as individual-level fixed effects to either model 2 or 3: maternal age, parity, ethnicity (Model 2). Our strategy was to remove fixed interaction effects or random effects if not statistically significant, or if model fitting became problematic. Furthermore, within each study, independent sample *t*-tests were carried out to explore the differences in weight gain per week by education level.

Patient involvement

No patients were involved in the design of this study. However we plan to share and disseminate the findings with the ROLO Families Advisory Committee and the ROLO participants via online social media and quarterly newsletters.

Results

Protocol, IPD obtained and Study characteristics

The PRISMA-IPD checklist is included in the additional files. The original i-WIP IPD dataset contained 12,240 participants from 36 studies. Participants were excluded if they were missing data relating to the main outcomes or met the exclusion criteria (Table 1).

In total, 7,057 women were excluded from the analysis, while 5,183 remained. Participants that met the inclusion criteria were enrolled to 21 RCTs (26–46). The study characteristics, methods used to measure GWG, intervention details and outcome measures of the eligible trials are summarised in Table S1.

For peer review only

For peer review only - http://bmjopen.bmj.com/site/about/guidelines.xhtml

IPD integrity and Risk of bias assessment

No issues were identified in the integrity of the IPD. The global classification of risk of bias classified 12 studies as high risk and 9 as low/medium risk (Table S2). In terms of publication bias, the funnel plot (Figure S1) demonstrated an even distribution of effect estimates and standard error, indicating low bias in publications.

Results of individual studies

For the majority of studies, we did not observe a significant difference in GWG/week (kg) between participants in the intervention and control, split by educational attainment (Table S3). Within four of the studies (28,34,41,45), women with tertiary education enrolled to the intervention had significantly less weight gain/week. One study found that women with secondary education or less enrolled to the intervention had significantly less weight gain/week (46). The forest plot (Figure S2) demonstrates the results of individual studies for the IPD meta-analysis of low educational attainment and gestational weight gain per week, controlling for BMI and intervention. A comparison of maternal characteristics among women included and excluded in the IPD meta-analysis (Table S4) demonstrated some differences across the populations.

Results of syntheses

Of the 5,183 participants in this analysis, 3,273 (63.1%) had achieved at least some tertiary education, while 1,910 (36.9%) had, at most, completed secondary education. Within the secondary education group, 931 (48.8%) were enrolled to the control arm, and 653 (34.2%), 186 (9.7%) and 140 (7.3%) were enrolled to the intervention arm of mixed, diet, and exercise trials, respectively. Within the tertiary education group, 1,531 (46.8%) were enrolled to the control arm of their respective RCT, while 1,023 (31.2%), 323 (9.9%) and 396 (12.1%) were enrolled to the intervention arm of mixed, diet and exercise trials, respectively.

Compared to women with tertiary education, women with lower education levels had significantly higher early-pregnancy weight and pre-pregnancy BMI, and were significantly

For peer review only - http://bmjopen.bmj.com/site/about/guidelines.xhtml

shorter in height (Table 2). Fewer women with tertiary education had a BMI >30 kg/m^2, compared to women in the secondary education group (26.8% *vs.* 46.3%, *P* <0.001). Women within the tertiary education group were older, had lower parity and were more likely to be of Caucasian ethnicity compared to those with lower education.

Mean ± SD GWG per week and percentage of participants within each of the IOM categories, stratified by educational attainment and BMI category are shown in Table 3. Figure 1 displays GWG (kg/week), according to pre-pregnancy BMI, intervention and educational attainment.

The unadjusted linear mixed-models found that women with lower educational attainment gained significantly more weight per week (kg) compared to those with tertiary education (B = 0.014), however this association was not significant when the model was adjusted for maternal age, parity and ethnicity (Table 4a). The ICC_{study} was 0.1% and $ICC_{intervention}$ was 0.0%. Women of lower educational attainment had an increased risk of both excessive (OR: 1.182) and inadequate (OR: 1.284) GWG as per the IOM guidelines, controlling for intervention, maternal age, parity, ethnicity, with random effects (Table 4b). The ICC calculations for model 2 of Table 4 (b) were low; excessive weight gain: ICC_{study} 8.1% and $ICC_{intervention}$ 0.4%; inadequate weight gain: ICC_{study} 5.0% and $ICC_{intervention}$ 0.7%.

The intervention-by-education interaction effects were not statistically significant, and for ease of interpretation, these were removed, thus we present results for models stratified by education.

Among those of lower educational attainment, a significant reduction in weight gain per week (kg) was observed for both diet-alone interventions (B = -0.055) and mixed interventions (B = -0.044), compared to those in the control arms, Table 5 (a). No significant effect was observed for exercise-alone interventions. Women with tertiary education enrolled to mixed interventions gained significantly less GWG per week (kg) (B = -0.053), than those in the control arms. Neither diet-alone nor exercise-alone interventions had significant effects on

For peer review only - http://bmjopen.bmj.com/site/about/guidelines.xhtml

weight gain per week (kg) for women with tertiary education. The adjusted models (Model 2) included BMI, maternal age, parity and ethnicity as confounders, with fixed effects.

Women with lower education were significantly less likely to exceed the IOM guidelines for weight gain per week if they were enrolled to diet-alone interventions (OR: 0.515) or mixed intervention (OR: 0.735), compared to those in the control arms, Table 5 (b). These women were also less likely to gain inadequate weight per week if they were enrolled to a diet-alone intervention (OR: 0.504). Women with tertiary education enrolled to diet-alone interventions were less likely to exceed IOM guidelines (OR: 0.609), however, those enrolled to mixed interventions were more likely to gain inadequate weight gain per week (OR: 1.411), compared to those in the control arms. No effect was observed for exercise-alone interventions. The adjusted models (Model 2) included maternal age, parity and ethnicity as confounders, with fixed effects.

For peer review only

For peer review only - http://bmjopen.bmj.com/site/about/guidelines.xhtml

Discussion

Summary of evidence

This IPD meta-analysis of the i-WIP consortium of lifestyle interventions in pregnancy found that low maternal education is associated with increased risk of both excessive and inadequate GWG per week, although crude differences were not observed in the percentage of women who gained inadequately, adequately and excessively between women of high and low educational attainment. Diet-alone interventions reduced the risk of excessive GWG for all women, and reduced the risk of inadequate GWG for those with lower educational attainment. Mixed interventions were protective against excessive GWG for women with low education, but increased the risk of inadequate GWG among women with higher educational attainment. Exercise-alone interventions were not associated with GWG, regardless of maternal education status.

Compared to the literature

Maternal educational attainment less than or equal to secondary level was associated with an 18% increased risk of excessive GWG and a 28% increased risk of inadequate GWG per week, as per the IOM 2009 guidelines (16), compared to those with at least some tertiary education. A systematic review of observational studies also found that low maternal educational attainment is associated with women gaining outside the 2009 IOM recommendations for GWG (10). However, the review only provided a narrative of the evidence to date, not a meta-analysis, thus was restricted from drawing firm conclusions. Nonetheless, both the systematic review and our findings highlight the need for additional support for pregnant women of low educational attainment. Although the increased risk of inadequate and excessive GWG among women with lower education was statistically significant, this is not synonymous with clinical significance. Between women of low and high educational attainment, the actual percentage differences in those who gained inadequately (19.0% vs. 14.5%), adequately (21.0% vs. 25.1%) and excessively (60.0% vs. 60.4%) was relatively small.

For peer review only - http://bmjopen.bmj.com/site/about/guidelines.xhtml

The impact of interventions in pregnancy on weight gain has been previously examined (19,21,23,47), however, analyses were not stratified by socioeconomic status within these publications, thus our study provides new insights in this field. A 2015 Cochrane review compared the effect of interventions in pregnancy (diet or exercise, or both) on GWG. Unlike our findings, in which only diet and mixed interventions reduced excessive weight gain, all categories of interventions significantly reduced excessive weight gain compared to control; diet (RR: 0.77), diet and exercise (RR: 0.86), and diet counselling/other (RR: 0.46) (47). However, the review also highlighted an increased risk of low weight gain with all types of interventions; diet (RR: 1.24), diet and exercise (RR: 1.25), and diet counselling/other (RR: 1.21) (47). A systematic review by Thangaratinam *et al.* found that dietary interventions reduced total GWG by 3.84 kg, while mixed and physical activity interventions reduced total GWG to lesser extents (–1.06 kg and –0.72 kg, respectively) (23). Both mixed and physical activity interventions reduced risk of excessive weight gain, but insufficient data were available to assess the impact of dietary interventions on excessive weight gain (23). The i-WIP primary publication in 2017 found that diet and physical activity based interventions during pregnancy reduce GWG (−0.72 kg and −0.73 kg, respectively), but did not find differences in GWG across the subgroups examined (educational attainment was not assessed) (21).

In summary, based on our findings and previous studies, it seems that among women of high and low educational status, dietary interventions are most effective in reducing GWG. Mixed interventions could also be considered for those with lower education. Our study is novel in that it is the first IPD meta-analysis, using a one-step approach for statistical analyses, in which data from individual pregnant women were used to estimate the effect of varying types of lifestyle interventions on GWG of women from different educational backgrounds.

Clinical relevance of our findings

In practice, dietary interventions have the potential be used as a baseline intervention, offered to all pregnant women, to encourage appropriate weight gain. Women with secondary education or less could be offered additional behaviour change counselling or physical activity advice, to

For peer review only - http://bmjopen.bmj.com/site/about/guidelines.xhtml

complement the dietary intervention. The reduction in gestational weight gain associated with dietary and mixed interventions among women of low educational attainment can be considered clinically significant, given that if a woman was to follow the prescribed intervention for the duration of trimester two and three (27 weeks) she would gain 1.49 and 1.19 kg less, respectively, than a woman following usual care.

The success of dietary interventions over other lifestyle interventions in reducing GWG may be attributed to many factors. The importance of a woman's diet in pregnancy has been documented for centuries (48), thus, dietary changes are perhaps more acceptable than changes in physical activity behaviours. Pregnancy itself is an incentive for positive behaviour changes in dietary choices, but less so for physical activity (17). Misconceptions regarding exercise during pregnancy remain, with many perceiving it to pose a safety risk for their baby (49), and believing pregnancy is a time for rest (50). In addition, women may experience feelings of tiredness, nausea and physical discomfort, as well as time commitments that prevent them from exercising (49–51).

Mixed approach interventions increased the risk of inadequate weight gain among highly educated women. A potential hypothesis is that these women may already be aware of healthy lifestyle strategies, have a higher locus of control (52) and future salience (53), and therefore may be more willingly to take actions for reducing weight gain. Consequently, providing multiple approaches to reduce GWG could have a paradoxical effect. However, since up to 80% of highly educated women in this meta-analysis exceeded weight gain guidelines, the focus of healthcare professionals should be on meeting adequate GWG, rather than exerting excess caution in avoiding mixed approach interventions. It is reasonable to suggest that the dietary approach would be sufficient among those with tertiary education in reducing excessive GWG.

Strengths and limitations

The pooling of individual patient data allows for the estimation of the differential effects of education and lifestyle interventions on weight gain across the included cohorts with greater power than individual RCTs (20). IPD meta-analysis does not rely on aggregate data extracted from RCTs, and allows for models to be adjusted for confounders (22). Despite the congruence between

For peer review only - http://bmjopen.bmj.com/site/about/guidelines.xhtml

our results and those in the wider literature, it must be highlighted that reporting of negative findings in lifestyle interventions is poor, thus the evidence base to which we compare our findings may not reflect the true nature of lifestyle interventions in pregnancy. The data were however strengthened by the relatively low variability between studies. In addition, we analysed and reported findings using GWG/week, which enables studies that use different gestational ages for measuring GWG to be compared.

We acknowledge that a limitation of our work is the lack of pre-pregnancy weight measurements available, a methodological issue that affects much GWG research (54). We classified BMI using self-reported pre-pregnancy weight, which may be subject to underreporting and hence under-classification of BMI (54). However, using the first trimester weight to determine pre-pregnancy BMI would also have been problematic due to weight gain in trimester 1 and potential for over-classification of BMI (54). Although it has been suggested that pre-pregnancy weight can be somewhat accurately estimated using the first trimester weight, by 12 weeks' gestation weight gain can result in up to 9% of women being misclassified for BMI (55). This was not a viable option since some the first weight measurements were taken as late as 18 weeks. Early-pregnancy weight was estimated using self-reported pre-pregnancy weight for a small number of participants (n=295, 5.7%).

While limitations of the IOM guidelines have been expressed (56), they are the most widely used recommendations, and are comparable to Intergrowth 21st Project weight gain centiles for women of normal BMI (57). We also used a novel method of extrapolating the IOM expected weight gain per week in trimester 1, and this enabled calculation of trimester 2 and 3 weight gain per week.

The maternal characteristics of women who were included in the IPD meta-analysis were different to those excluded; a finding that is likely related to the eligibility criteria of this meta-analysis which excluded all data from some studies that did not collect data pertaining to key characteristics required.

Lastly, it must be stated that although the results were statistically significant, the effect sizes were small, thus the relevance of our findings in the clinical setting must be considered.

For peer review only - http://bmjopen.bmj.com/site/about/guidelines.xhtml

Conclusions

In summary, pregnant women with lower educational attainment are at an increased risk of both excessive and inadequate weight gain highlighting the need for intervention among this vulnerable population of women. Given that dietary interventions do not widen health inequalities, are successful in promoting appropriate weight gain, and are acceptable; healthcare professionals should consider implementing nutrition based interventions as part of baseline maternity care packages for all pregnant women.

For peer review only

For peer review only - http://bmjopen.bmj.com/site/about/guidelines.xhtml

Tables

Table 1. Participant Exclusion Criteria for Individual Participant Data

Reason for Exclusion	Excluded N	Remaining N
Full dataset		12240
Data missing: Baseline gestational age	2611	9629
Data missing: Final follow-up gestational age	2309	7320
Data missing: Maternal educational attainment	1878	5442
Multiple pregnancy	17	5425
Less than 18 years of age	75	5350
Data missing: Pre- and early-pregnancy weight	1	5349
Data missing: Final follow-up weight	137	5212
Data missing: Height (for pre-pregnancy BMI)	29	5183

For peer review only

Table 2. Participant characteristics in the total group, and split by education category

	Total N		Lower Education N		Higher Education N		*P**
**Early pregnancy weight (kg)** [a]	5183	78.60 (18.56)	1910	82.51 (20.26)	3273	76.31 (17.08)	<0.001
**Pre-pregnancy weight (kg)** [a]	5183	76.06 (18.74)	1712	79.61 (20.37)	3176	73.17 (16.96)	<0.001
**Height (cm)** [a]	5183	166.07 (7.03)	1910	164.76 (7.17)	3273	166.84 (6.84)	<0.001
**Pre-pregnancy BMI** (kg/m^2) [a]	5183	27.57 (6.55)	1910	29.45 (7.08)	3273	26.47 (5.95)	<0.001
Underweight [b]	5183	77 (1.5)	1910	32 (1.6))	3273	46 (1.4)	<0.001
Normal-weight [b]		2274 (43.9)		603 (31.5)		1672 (51.1)	
Overweight [b]		1073 (20.7)		393 (20.5)		681 (20.8)	
Obese [b]		1763 (34.0)		886 (46.3)		878 (26.8)	
**Gestational age** (weeks)							
**Baseline** [c]	5183	14.5 (4.0 - 32.0)	1910	13.9 (4.0 - 32.0)	3273	14.8 (4.0 - 32.0)	<0.001
**Final follow-up** [c]	5183	36.6 (24.0 - 44.0)	1910	36.6 (27.0 - 44.0)	3273	36.5 (24 - 43.0)	0.196
**Delivery** [c]	5172	39.7 (26.0 - 44.0)	1907	39.50 (27.0 - 44.0)	3265	39.75 (26.0 - 43.0)	<0.001
**Infant birth weight** (g) [a]	5164	3531.73 (547.90)	1908	3476.55 (559.43)	3256	3564.06 (538.49)	<0.001
**Maternal age** (years) [a]	5183	29.90 (4.97)	1910	28.50 (5.51)	3273	30.72 (4.43)	<0.001
**Parity** [a]	5135	0.73 (1.05)	1884	0.93 (1.20)	3251	0.62 (0.93)	<0.001
**Ethnicity**							
Caucasian [b]	3719	3291 (63.5)	1203	954 (49.9)	2516	2338 (71.4)	<0.001
Asian [b]		84 (1.6)		25 (1.3)		60 (1.8)	
Black [b]		123 (2.4)		71 (3.7)		53 (1.6)	
Central/South American [b]		88 (1.7)		76 (3.9)		13 (.4)	
Middle East (including Iran & Turkey) [b]		63 (1.2)		45 (2.3)		19 (.5)	
Other [b]		76 (1.4)		38 (1.9)		39 (1.2)	
Missing [b]		1465 (28.2)		708 (37.0)		758 (23.1)	

Data are presented as [a] mean (SD) [b] n (%) and [c] mean (range of minimum and maximum).
**P*-value: ANOVA and chi-square
BMI: body mass index. Underweight: <18.5 kg/m^2; Normal:18.5 - 24.9 kg/m^2; Overweight: 25.0 - 29.9 kg/m^2; Obese: ≥30 kg/m^2
Lower education: secondary education or less. Higher education: at least some tertiary education

For peer review only - http://bmjopen.bmj.com/site/about/guidelines.xhtml

Table 3. Gestational Weight Gain per Week (kg/week), and per IOM Guidelines, BMI Category and Education

			GWG/week (kg)	Trimester 2 and 3 GWG/week (kg), and per IOM Guidelines			
	BMI Category	N	Total GWG/week Mean (SD)	T2 & 3 GWG/week Mean (SD)	Inadequate, n (%)	Adequate, n (%)	Excess, n (%)
All Participants	Total	5183	0.45 (0.22)	0.46 (0.22)	836 (16.1%)	1224 (23.6%)	3123 (60.3%)
	Underweight	76	0.50 (0.16)	0.51 (0.16)	23 (30.3%)	34 (44.7%)	19 (25.0%)
	Normal-weight	2273	0.51 (0.28)	0.51 (0.18)	347 (15.3%)	778 (34.2%)	1148 (50.5%)
	Overweight	1072	0.48 (0.22)	0.49 (0.22)	113 (10.5%)	128 (11.9%)	831 (77.5%)
	Obese	1762	0.35 (0.23)	0.36 (0.24)	353 (20.0%)	284 (16.1%)	1125 (63.8%)
Lower Education	Total	1910	0.42 (0.24)	0.43 (0.25)	363 (19.0%)	401 (21.0%)	1146 (60.0%)
	Underweight	31	0.55 (0.20)	0.56 (0.20)	7 (22.6%)	11 (35.5%)	13 (41.9%)
	Normal-weight	602	0.51 (0.20)	0.52 (0.21)	108 (17.9%)	184 (30.6%)	310 (51.5%)
	Overweight	392	0.46 (0.24)	0.47 (0.24)	55 (14.0%)	59 (15.1%)	278 (70.9%)
	Obese	885	0.34 (0.24)	0.35 (0.25)	193 (21.8%)	147 (16.6%)	545 (61.6%)
Higher Education	Total	3273	0.46 (0.20)	0.47 (0.21)	473 (14.5%)	823 (25.1%)	1977 (60.4%)
	Underweight	45	0.46 (0.10)	0.47 (0.11)	16 (35.6%)	23 (51.1%)	6 (13.3%)
	Normal-weight	1671	0.50 (0.17)	0.51 (0.17)	239 (14.3%)	594 (35.5%)	838 (50.1%)
	Overweight	680	0.49 (0.20)	0.50 (0.20)	58 (8.5%)	69 (10.1%)	553 (81.3%)
	Obese	877	0.35 (0.23)	0.37 (0.24)	160 (18.2%)	137 (15.6%)	580 (66.1%)

GWG: gestational weight gain
BMI: body mass index. Underweight: <18.5 kg/m^2; Normal:18.5 - 24.9 kg/m^2; Overweight: 25.0 - 29.9 kg/m^2; Obese: ≥30 kg/m^2
Lower education: secondary education or less. Higher education: at least some tertiary education

For peer review only

Table 4. Generalised Linear Mixed-Models to show relationship between maternal educational attainment and (a) gestational weight gain per week (kg), and (b) gestational weight gain per week as per the IOM guidelines

		(a) Gestational Weight Gain per Week (kg/week)			(b) Gestational Weight Gain per Week as per the IOM Guidelines					
					Excessive Weight Gain			Inadequate Weight Gain		
	**Education**	***B***	**95% CI**	***P****	**OR**	**95% CI**	***P****	**OR**	**95% CI**	***P****
**MODEL 1 – (n = 5183) †**	Lower	0.014	(0.001 - 0.027)	0.032	1.224	(1.052 - 1.425)	0.009	1.259	(1.033 - 1.535)	0.022
	Higher	Ref			Ref			Ref		
**MODEL 2 – (n = 5135) ‡**	Lower	0.005	(-0.008 - 0.018)	0.465	1.182	(1.008 - 1.385)	0.039	1.284	(1.045 - 1.577)	0.017
	Higher	Ref			Ref			Ref		

**P*-value: Wald test.
† Adjusted for intervention with random intercept and intervention slope. Weight gain per week (kg/week) additionally adjusted for BMI (kg/m^2)
‡ Adjusted for intervention, maternal age, parity, ethnicity, with random intercept and intervention slope. Weight gain per week (kg/week) additionally adjusted for BMI (kg/m^2)
Lower education: secondary education or less. Higher education: at least some tertiary education

For peer review only

For peer review only - http://bmjopen.bmj.com/site/about/guidelines.xhtml

Table 5. Generalised Linear Mixed-Models to show relationship between maternal educational attainment, type of intervention and (a) gestational weight gain per week (kg), and (b) gestational weight gain per week as per the IOM guidelines

			(a) Gestational Weight Gain per Week (kg/week)			(b) Gestational Weight Gain per Week as per the IOM Guidelines					
						Excessive Weight Gain			Inadequate Weight Gain		
	Education	Intervention Type	*B*	95% CI	*P**	OR	95% CI	*P**	OR	95% CI	*P**
MODEL 1 †	Lower (n = 1910)	Mixed	-0.044	(-0.069, -0.019)	<0.001	0.745	(0.572, 0.971)	0.029	1.039	(0.745, 1.449)	0.821
		Diet	-0.053	(-0.097, -0.009)	0.018	0.524	(0.351, 0.782)	0.002	0.496	(0.285, 0.864)	0.013
		Exercise	-0.020	(-0.069, 0.029)	0.422	0.704	(0.446, 1.110)	0.131	0.634	(0.333, 1.208)	0.166
		Control	Ref			Ref			Ref		
	Higher (n = 3273)	Mixed	-0.056	(-0.071, -0.040)	<0.001	0.930	(0.753, 1.150)	0.504	1.407	(1.057, 1.874)	0.019
		Diet	-0.015	(-0.038, 0.009)	0.213	0.619	(0.446, 0.858)	0.004	0.943	(0.565, 1.574)	0.822
		Exercise	-0.014	(-0.036, 0.008)	0.206	1.027	(0.753, 1.402)	0.865	1.209	(0.770, 1.899)	0.410
		Control	Ref			Ref			Ref		
MODEL 2 ‡	Lower (n = 1884)	Mixed	-0.044	(-0.069, -0.020)	<0.001	0.735	(0.561, 0.963)	0.026	1.041	(0.744, 1.458)	0.813
		Diet	-0.055	(-0.098, -0.012)	0.012	0.515	(0.339, 0.785)	0.002	0.504	(0.288, 0.884)	0.017
		Exercise	-0.018	(-0.068, 0.032)	0.472	0.660	(0.405, 1.075)	0.095	0.612	(0.314, 1.193)	0.149
		Control	Ref			Ref			Ref		
	Higher (n = 3251)	Mixed	-0.053	(-0.069, -0.037)	<0.001	0.947	(0.765, 1.174)	0.620	1.411	(1.056, 1.884)	0.020
		Diet	-0.013	(-0.037, 0.010)	0.271	0.609	(0.437, 0.849)	0.003	0.962	(0.572, 1.616)	0.882
		Exercise	-0.018	(-0.040, 0.004)	0.111	1.030	(0.751, 1.413)	0.855	1.205	(0.761, 1.909)	0.427
		Control	Ref			Ref			Ref		

**P*-value: Wald test
† With fixed effects. Weight gain per week (kg/week) adjusted for BMI (kg/m^2)
‡ With fixed effects. Adjusted for maternal age, parity and ethnicity. Weight gain per week (kg/week) adjusted for BMI (kg/m^2)
Lower education: secondary education or less. Higher education: at least some tertiary education

For peer review only

For peer review only - http://bmjopen.bmj.com/site/about/guidelines.xhtml

Author contributions

E.C. O'Brien	Wrote a proposal to the i-WIP data access committee. Set the objectives, study design and data analysis plan. Analysis and interpretation of data; drafting of manuscript; critical revision; final approval of version to be published; agreeable to be accountable for all aspects of the work.
R. Segurado	Analysis and interpretation of data; drafting of manuscript; critical revision; final approval of version to be published; agreeable to be accountable for all aspects of the work.
A.A. Geraghty	Analysis and interpretation of data; drafting of manuscript; critical revision; final approval of version to be published; agreeable to be accountable for all aspects of the work.
G. Alberdi	Analysis and interpretation of data; drafting of manuscript; critical revision; final approval of version to be published; agreeable to be accountable for all aspects of the work.
E. Rogozińska	i-WIP IPD database manager; study conception and design; critical revision; final approval of version to be published; agreeable to be accountable for all aspects of the work.
A. Astrup	Study conception and design; critical revision; final approval of version to be published; agreeable to be accountable for all aspects of the work.
R. Barakat Carballo	Study conception and design; critical revision; final approval of version to be published; agreeable to be accountable for all aspects of the work.
A. Bogaerts	Study conception and design; critical revision; final approval of version to be published; agreeable to be accountable for all aspects of the work.
J.G. Cecatti	Study conception and design; critical revision; final approval of version to be published; agreeable to be accountable for all aspects of the work.
A. Coomarasamy	Study conception and design; critical revision; final approval of version to be published; agreeable to be accountable for all aspects of the work.
C.J. de Groot	Study conception and design; critical revision; final approval of version to be published; agreeable to be accountable for all aspects of the work.
R. Devlieger	Study conception and design; critical revision; final approval of version to be published; agreeable to be accountable for all aspects of the work.
J.Dodd	Study conception and design; critical revision; final approval of version to be published; agreeable to be accountable for all aspects of the work.
N.El Beltagy	Study conception and design; critical revision; final approval of version to be published; agreeable to be accountable for all aspects of the work.
F. Facchinetti	Study conception and design; critical revision; final approval of version to be published; agreeable to be accountable for all aspects of the work.
N.R. Geiker	Study conception and design; critical revision; final approval of

For peer review only

	version to be published; agreeable to be accountable for all aspects of the work.
K.J Guelfi	Study conception and design; critical revision; final approval of version to be published; agreeable to be accountable for all aspects of the work.
L. Haakstad	Study conception and design; critical revision; final approval of version to be published; agreeable to be accountable for all aspects of the work.
C.Harrison	Study conception and design; critical revision; final approval of version to be published; agreeable to be accountable for all aspects of the work.
H. Hauner	Study conception and design; critical revision; final approval of version to be published; agreeable to be accountable for all aspects of the work.
D.M. Jensen	Study conception and design; critical revision; final approval of version to be published; agreeable to be accountable for all aspects of the work.
K.S. Khan	Study conception and design; critical revision; final approval of version to be published; agreeable to be accountable for all aspects of the work.
T. I. Kinnunen	Study conception and design; critical revision; final approval of version to be published; agreeable to be accountable for all aspects of the work.
R. Luoto	Study conception and design; critical revision; final approval of version to be published; agreeable to be accountable for all aspects of the work.
B. W. Mol	Study conception and design; critical revision; final approval of version to be published; agreeable to be accountable for all aspects of the work.
S. Mørkved	Study conception and design; critical revision; final approval of version to be published; agreeable to be accountable for all aspects of the work.
N. Motahari	Study conception and design; critical revision; final approval of version to be published; agreeable to be accountable for all aspects of the work.
J. Owens	Study conception and design; critical revision; final approval of version to be published; agreeable to be accountable for all aspects of the work.
M. Perales	Study conception and design; critical revision; final approval of version to be published; agreeable to be accountable for all aspects of the work.
E. Petrella	Study conception and design; critical revision; final approval of version to be published; agreeable to be accountable for all aspects of the work.
S. Phelan	Study conception and design; critical revision; final approval of version to be published; agreeable to be accountable for all aspects of the work.
L. Poston	Study conception and design; critical revision; final approval of version to be published; agreeable to be accountable for all aspects of the work.
K. Rauh	Study conception and design; critical revision; final approval of

For peer review only

	version to be published; agreeable to be accountable for all aspects of the work.
G. Rayanagoudar	Study conception and design; critical revision; final approval of version to be published; agreeable to be accountable for all aspects of the work.
K.M. Renault	Study conception and design; critical revision; final approval of version to be published; agreeable to be accountable for all aspects of the work.
A.E. Ruifrok	Study conception and design; critical revision; final approval of version to be published; agreeable to be accountable for all aspects of the work.
L.R. Sagedal	Study conception and design; critical revision; final approval of version to be published; agreeable to be accountable for all aspects of the work.
K.Å Salvesen	Study conception and design; critical revision; final approval of version to be published; agreeable to be accountable for all aspects of the work.
T.T. Scudeller	Study conception and design; critical revision; final approval of version to be published; agreeable to be accountable for all aspects of the work.
G.X. Shen	Study conception and design; critical revision; final approval of version to be published; agreeable to be accountable for all aspects of the work.
A. Shub	Study conception and design; critical revision; final approval of version to be published; agreeable to be accountable for all aspects of the work.
S.N. Stafne	Study conception and design; critical revision; final approval of version to be published; agreeable to be accountable for all aspects of the work.
F. Surita	Study conception and design; critical revision; final approval of version to be published; agreeable to be accountable for all aspects of the work.
S. Thangaratinam	Study conception and design; critical revision; final approval of version to be published; agreeable to be accountable for all aspects of the work.
S. Tonstad	Study conception and design; critical revision; final approval of version to be published; agreeable to be accountable for all aspects of the work.
M.N. van Poppel	Study conception and design; critical revision; final approval of version to be published; agreeable to be accountable for all aspects of the work.
C. Vinter	Study conception and design; critical revision; final approval of version to be published; agreeable to be accountable for all aspects of the work.
I. Vistad	Study conception and design; critical revision; final approval of version to be published; agreeable to be accountable for all aspects of the work.
S. Yeo	Study conception and design; critical revision; final approval of version to be published; agreeable to be accountable for all aspects of the work.
i-WIP (International	NA – group name

For peer review only

Weight Management in Pregnancy) Collaborative Group	
F.M. McAuliffe	Wrote a proposal to the i-WIP data access committee. Set the objectives, study design and data analysis plan. Analysis and interpretation of data; drafting of manuscript; critical revision; final approval of version to be published; agreeable to be accountable for all aspects of the work.

Competing interests statement

The authors did not report any potential conflicts of interest

Funding Statement

The data come from a National Institute for Health Research (NIHR) funded project (HTA - 12/01/50) and the Queen Mary University of London is its legal Sponsor. The database was initially funded by the NIHR and is currently funded by World Health Organization (WHO). This work was also supported by the Health Research Board, Health Research Centre for Health and Diet Research, Ireland.

Data sharing statement

The Queen Mary University of London is the legal custodian of the i-WIP dataset. The access to the dataset is regulated by prespecified terms and conditions (available on request) and overseen by Data Access Committee. For further information visit: https://iwipgroup.wixsite.com/collaboration/related-projects

Acknowledgements

We are also grateful to the i-WIP Network for the individual participant data shared and to Helena Teede, Janette Khoury and Márcia Vitolo for the contribution of data.

For peer review only - http://bmjopen.bmj.com/site/about/guidelines.xhtml

References

1. United Nations. Transforming Our World: The 2030 Agenda for Sustainable Development. New York; 2015.

2. Agyemang C, Vrijkotte TGM, Droomers M, van der Wal MF, Bonsel GJ, Stronks K. The effect of neighbourhood income and deprivation on pregnancy outcomes in Amsterdam, The Netherlands. J Epidemiol Community Health. 2009;63(9):755-760.

3. Metcalfe A, Lail P, Ghali WA, Sauve RS. The association between neighbourhoods and adverse birth outcomes: a systematic review and meta-analysis of multi-level studies. Paediatr Perinat Epidemiol. 2011;25(3):236-245.

4. Morgen CS, Bjørk C, Andersen PK, Mortensen LH, Nybo Andersen A-M, Bjork C, et al. Socioeconomic position and the risk of preterm birth-a study within the Danish National Birth Cohort. Int J Epidemiol. 2008;37:1109-1120.

5. Silva LM, Jansen PW, Steegers E a P, Jaddoe VW V, Arends LR, Tiemeier H, et al. Mother's educational level and fetal growth: The genesis of health inequalities. Int J Epidemiol. 2010;39:1250-1261.

6. Blumenshine P, Egerter S, Barclay CJ, Cubbin C, Braveman PA. Socioeconomic Disparities in Adverse Birth Outcomes. Am J Prev Med. 2010;39(3):263-272.

7. Tofani AA, Lamarca G de A, Sheiham A, Vettore MV. The different effects of neighbourhood and individual social capital on health-compromising behaviours in women during pregnancy: a multi-level analysis. BMC Public Health. 2015;15:890.

8. Freisling H, Elmadfa I, Gall I. The effect of socioeconomic status on dietary intake, physical activity and Body Mass Index in Austrian pregnant women. J Hum Nutr Diet. 2006;19:437-445.

9. O'Brien EC, Alberdi G, Geraghty AA, McAuliffe FM. Lower education predicts poor response to dietary intervention in pregnancy, regardless of neighbourhood

For peer review only - http://bmjopen.bmj.com/site/about/guidelines.xhtml

affluence: secondary analysis from the ROLO randomised control trial. Public Health Nutr. 2017;20(16):2959–69.

10. O'Brien EC, Alberdi G, McAuliffe FM. The influence of socioeconomic status on gestational weight gain: a systematic review. J Public Heal. 2018;40(1):41-55

11. Truong YN, Yee LM, Caughey AB, Cheng YW. Weight gain in pregnancy: does the Institute of Medicine have it right? Am J Obstet Gynecol. 2015;212(3):362.e1-e8.

12. Goldstein RF, Abell SK, Ranasinha S, Misso M, Boyle J a., Black MH, et al. Association of gestational weight gain with maternal and infant outcomes: A systematic review and meta-analysis. JAMA. 2017;317(21):2207-2225.

13. O'Brien EC, Geraghty AA, O'Sullivan EJ, Riordan J, Horan MK, Larkin E, et al. Five-year follow up of a low glycaemic index dietary randomised controlled trial in pregnancy—no long-term maternal effects of a dietary intervention. BJOG.2019;126(4):514–24.

14. Amorim AR, Rössner S, Neovius M, Lourenço PM, Linné Y. Does excess pregnancy weight gain constitute a major risk for increasing long-term BMI? Obesity (Silver Spring). 2007;15(5):1278-1286.

15. NCD Risk Factor Collaboration (NCD-RisC). Trends in adult body-mass index in 200 countries from 1975 to 2014: a pooled analysis of 1698 population-based measurement studies with 19.2 million participants. Lancet. 2016;387(10026):1377-1396.

16. Institute of Medicine. National Research Council (US) Committee to Reexamine IOM Pregnancy Weight Guidelines. Weight Gain During Pregnancy. Washington (DC): National Academies Press (US); 2009.

17. O'Brien OA, Lindsay KL, McCarthy M, McGloin AF, Kennelly M, Scully HA, et al. Influences on the food choices and physical activity behaviours of overweight and obese pregnant women: A qualitative study. Midwifery. 2017;(47):28-35.

For peer review only

18. Jackson RA, Stotland NE, Caughey AB, Gerbert B. Improving diet and exercise in pregnancy with Video Doctor counseling: A randomized trial. Patient Educ Couns. 2011;83(2):203-209.

19. Thangaratinam S, Rogozinska E, Jolly K, Glinkowski S, Duda W, Borowiack E, et al. Interventions to reduce or prevent obesity in pregnant women: a systematic review. Health Technol Assess. 2012;16(31).

20. Ruifrok AE, Rogozinska E, van Poppel MNM, Rayanagoudar G, Kerry S, de Groot CJM, et al. Study protocol: differential effects of diet and physical activity based interventions in pregnancy on maternal and fetal outcomes--individual patient data (IPD) meta-analysis and health economic evaluation. Syst Rev. 2014;3:131.

21. The International Weight Management in Pregnancy (i-WIP) Collaborative Group. Effect of diet and physical activity based interventions in pregnancy on gestational weight gain and pregnancy outcomes: meta-analysis of individual participant data from randomised trials. BMJ. 2017;358:j3119

22. Stewart LA, Clarke M, Rovers M, Riley RD, Simmonds M, Stewart G, et al. Preferred Reporting Items for a Systematic Review and Meta-analysis of Individual Participant Data. JAMA. 2015;313(16):1657.

23. Thangaratinam S, Rogozinska E, Jolly K, Glinkowski S, Roseboom T, Tomlinson JW, et al. Effects of interventions in pregnancy on maternal weight and obstetric outcomes: meta-analysis of randomised evidence. BMJ. 2012;344:e2088.

24. Ruifrok AE, Rogozinska E, van Poppel MNM, Rayanagoudar G, Kerry S, de Groot CJM, et al. Erratum to: Study protocol: differential effects of diet and physical activity based interventions in pregnancy on maternal and fetal outcomes: individual patient data (IPD) meta-analysis and health economic evaluation. Syst Rev. 2015;4:101.

25. Shavers VL. Measurement of socioeconomic status in health disparities research. J Natl Med Assoc. 2007;99(9):1013-1023.

For peer review only - http://bmjopen.bmj.com/site/about/guidelines.xhtml

26. Althuizen E, van der Wijden C, van Mechelen W, Seidell J, van Poppel M. The effect of a counselling intervention on weight changes during and after pregnancy: a randomised trial. BJOG. 2013;120:92-99.

27. Baciuk EP, Pereira RI, Cecatti JG, Braga AF, Cavalcante SR. Water aerobics in pregnancy: Cardiovascular response, labor and neonatal outcomes. Reprod Health. 2008;5:10.

28. Bogaerts AFL, Devlieger R, Nuyts E, Witters I, Gyselaers W, van den Bergh BRH. Effects of lifestyle intervention in obese pregnant women on gestational weight gain and mental health: a randomized controlled trial. Int J Obes. 2013;37(6):814-821.

29. Haakstad LAH, Bø K. Exercise in pregnant women and birth weight: a randomized controlled trial. BMC Pregnancy Childbirth. 2011;11:66.

30. Harrison CL, Lombard CB, Strauss BJ, Teede HJ. Optimizing healthy gestational weight gain in women at high risk of gestational diabetes: A randomized controlled trial. Obesity. 2013;21(5):904-909.

31. Jeffries K, Shub A, Walker SP, Hiscock R, Permezel M. Reducing excessive weight gain in pregnancy: a randomised controlled trial. Med J Aust. 2009;191(8):429-433.

32. Khaledan A, Mirdar S, Motahari Tabari N, Ahmad Shirvani M. Effect of an Aerobic Exercise Program on Fetal Growth in Pregnant Women. J Hayat. 2010;16(1):55-64.

33. Khoury J, Henriksen T, Christophersen B, Tonstad S. Effect of a cholesterol-lowering diet on maternal, cord, and neonatal lipids, and pregnancy outcome: A randomized clinical trial. Am J Obstet Gynecol. 2005;193(4):1292-1301.

34. Nascimento S, Surita F, Parpinelli M, Siani S, Pinto e Silva J. The effect of an antenatal physical exercise programme on maternal/perinatal outcomes and quality of life in overweight and obese pregnant women: a randomised clinical trial. BJOG. 2011;118:1455-1463.

For peer review only

35. Oostdam N, van Poppel M, Wouters M, Eekhoff E, Bekedam D, Kuchenbecker W, et al. No effect of the FitFor2 exercise programme on blood glucose, insulin sensitivity, and birthweight in pregnant women who were overweight and at risk for gestational diabetes: results of a randomised controlled trial. BJOG. 2012;119:1098-1107.

36. Petrella E, Malavolti M, Bertarini V, Pignatti L, Neri I, Battistini NC, et al. Gestational weight gain in overweight and obese women enrolled in a healthy lifestyle and eating habits program. J Matern Neonatal Med. 2014;27(13):1348-1352.

37. Phelan S, Phipps MG, Abrams B, Darroch F, Schaffner A, Wing RR. Randomized trial of a behavioral intervention to prevent excessive gestational weight gain: the Fit for Delivery Study. Am J Clin Nutr. 2011;93(4):772-779.

38. Poston L, Bell R, Croker H, Flynn AC, Godfrey KM, Goff L, et al. Effect of a behavioural intervention in obese pregnant women (the UPBEAT study): a multicentre, randomised controlled trial. Lancet Diabetes Endocrinol. 2015;3(10):767-777.

39. Rauh K, Gabriel E, Kerschbaum E, Schuster T, von Kries R, Amann-Gassner U, et al. Safety and efficacy of a lifestyle intervention for pregnant women to prevent excessive maternal weight gain: a cluster-randomized controlled trial. BMC Pregnancy Childbirth. 2013;13(1):151.

40. Renault KM, N Nørgaard K, Nilas L, Carlsen EM, Cortes D, Pryds O, et al. The Treatment of Obese Pregnant Women (TOP) study: A randomized controlled trial of the effect of physical activity intervention assessed by pedometer with or without dietary intervention in obese pregnant women. Am J Obstet Gynecol. 2014;210(2):134.e1-e134.e9.

41. Sagedal L, Øverby N, Bere E, Torstveit M, Lohne-Seiler H, Småstuen M, et al. Lifestyle intervention to limit gestational weight gain: the Norwegian Fit for Delivery randomised controlled trial. BJOG. 2016. doi: 10.1111/1471-0528.13862

For peer review only

For peer review only - http://bmjopen.bmj.com/site/about/guidelines.xhtml

42. Stafne SN, Salvesen KÅ, Romundstad PR, Stuge B, Mørkved S. Does regular exercise during pregnancy influence lumbopelvic pain? A randomized controlled trial. Acta Obstet Gynecol Scand. 2012;91:552-559.

43. Vinter CA, Jensen DM, Ovesen P, Beck-Nielsen H, Jørgensen JS. The LiP (Lifestyle in Pregnancy) study: a randomized controlled trial of lifestyle intervention in 360 obese pregnant women. Diabetes Care. 2011;34(12):2502-2507.

44. Vítolo MR, Bueno MSF, Gama CM. [Impact of a dietary counseling program on the gain weight speed of pregnant women attended in a primary care service]. Rev Bras Ginecol Obs. 2011;33(1):13-9.

45. Walsh JM, McGowan CA, Mahony R, Foley ME, McAuliffe FM. Low glycaemic index diet in pregnancy to prevent macrosomia (ROLO study): randomised control trial. BMJ. 2012;345:e5605.

46. Wolff S, Legarth J, Vangsgaard K, Toubro S, Astrup A. A randomized trial of the effects of dietary counseling on gestational weight gain and glucose metabolism in obese pregnant women. Int J Obes. 2008;32:495-501.

47. Muktabhant B, Lawrie TA, Lumbiganon P, Laopaiboon M. Diet or exercise, or both, for preventing excessive weight gain in pregnancy. Cochrane Database Syst Rev. 2015;6:CD007145.

48. Institute of Medicine (US) and Food and Nutritional Board. Historical Trends in Clinical Practice, Maternal Nutritional Status, and the Course and Outcome of Pregnancy. In: Nutrition During Pregnancy. Washington (DC); 1990:37-38.

49. Duncombe D, Wertheim EH, Skouteris H, Paxton SJ, Kelly L. Factors related to exercise over the course of pregnancy including women's beliefs about the safety of exercise during pregnancy. Midwifery. 2009;25(4):430-438.

50. Heery E, McConnon A, Kelleher CC, Wall PG, McAuliffe FM. Perspectives on weight gain and lifestyle practices during pregnancy among women with a history of

For peer review only

For peer review only - http://bmjopen.bmj.com/site/about/guidelines.xhtml

macrosomia: a qualitative study in the Republic of Ireland. BMC Pregnancy Childbirth. 2013;13:202.

51. Cioffi J, Schmied V, Dahlen H, Mills A, Thornton C, Duff M, et al. Physical Activity in Pregnancy: Women's Perceptions, Practices, and Influencing Factors. J Midwifery Womens Health. 2010;55(5):455-461

52. Weisman CS, Hillemeier MM, Chase GA, Misra DP, Chuang CH, Parrott R, et al. Women's perceived control of their birth outcomes in the Central Pennsylvania Women's Health Study: implications for the use of preconception care. Womens Health Issues. 2008;18(1):17-25.

53. Wardle J, Steptoe A. Socioeconomic differences in attitudes and beliefs about healthy lifestyles. J Epidemiol Community Health. 2003;57(6):440-443.

54. Gilmore LA, Redman LM. Weight gain in pregnancy and application of the 2009 IOM guidelines: toward a uniform approach. Obesity (Silver Spring). 2015;23(3):507-511.

55. Krukowski RA, West DS, DiCarlo M, Shankar K, Cleves MA, Saylors ME, et al. Are early first trimester weights valid proxies for preconception weight? BMC Pregnancy Childbirth. 2016;16(1):357.

56. Kapadia MZ, Park CK, Beyene J, Giglia L, Maxwell C, McDonald SD. Can we safely recommend gestational weight gain below the 2009 guidelines in obese women? A systematic review and meta-analysis. Obes Rev. 2015;16(3):189-206.

57. Cheikh Ismail L, Bishop DC, Pang R, Ohuma EO, Kac G, Abrams B, et al. Gestational weight gain standards based on women enrolled in the Fetal Growth Longitudinal Study of the INTERGROWTH-21st Project: a prospective longitudinal cohort study. BMJ. 2016;352:i555.

For peer review only

Figure Captions

Figure 1. Gestational weight gain (kg/week), stratified by to pre-pregnancy BMI, intervention and educational attainment

For peer review only

For peer review only - http://bmjopen.bmj.com/site/about/guidelines.xhtml

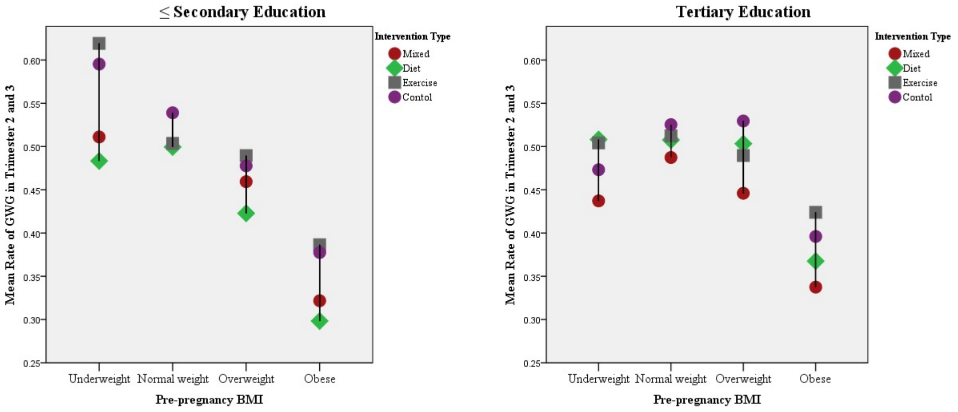

Figure 1. Gestational weight gain (kg/week), according to pre-pregnancy BMI, intervention and educational attainment

440x190mm (72 x 72 DPI)

Supplementary Files

For peer review only

Table S1. Study Characteristics of Randomised Control Trials Included in the IPD Meta-Analysis (n = 21)

Author (Year)	Period	Original inclusion/exclusion criteria	Country	Method to measure gestational weight gain (GWG)	Intervention	Intervention Detail	Outcome	Original i-WIP N<br>N (%) in this analysis
Althuizen *et al*. (2013)	February 2005 to May 2006	Inclusion criteria: primigravida, ability to read and speak Dutch, ≤14 weeks' gestation.	Netherlands	Pre-pregnancy: self-reported. Early pregnancy: measured at 15 weeks' gestation	Mixed	Counselling sessions (The New Life[style] Study) five times during pregnancy (15 minutes each) on weight, physical activity and diet.	Excessive weight gain, BMI, postpartum weight retention, birth weight, macrosomia, preterm birth, gestational diabetes	258<br>187 (72.5%)
Baciuk *et al*. (2008)	Not described	Inclusion criteria: <20 weeks' gestation, singleton pregnancy, receiving prenatal care at the institution and intended to give birth there. Exclusion criteria: engaging in regular physical activity, ≥2 caesarean sections, neurological, cardio-vascular, pulmonary, muscular-skeletal or endocrine disorders, morbid obesity, severe anaemia or vaginal bleeding during pregnancy.	Brazil	Pre-pregnancy: self-reported. Early pregnancy: (estimated from pre-pregnancy as per methods)	Exercise	Water aerobics three times per week (50 minutes each) in an indoor swimming pool.	Maternal cardiovascular capacity, heart rate, blood pressure, experience at delivery and fetal heart rate	71<br>68 (95.8%)
Bogaerts *et al*. (2013)	March 2008 and April 2011	Inclusion: BMI ≥29 kg/m^2. Exclusion criteria: >15 weeks' gestation, pre-existing type 1 diabetes, multiple pregnancy, primary need for nutritional advice and insufficient knowledge of the Dutch language	Belgium	Pre-pregnancy self-reported. Early pregnancy: (estimated from pre-pregnancy as per methods)	Mixed	Two arms. (1) Brochure group: brochure with advice on diet, physical activity and limiting excessive GWG, (2) Prenatal session group: brochure plus four prenatal motivational lifestyle intervention sessions led by a trained midwife.	GWG, Anxiety, feelings of depression	197<br>196 (99.5%)
Haakstad *et al*. (2011)	September 2007 to March 2008	Inclusion criteria: Nulliparous, low pre-pregnancy exercise, sufficient understanding of Norwegian, and ≤24 weeks' gestation. Exclusion criteria: history of miscarriages, severe heart disease, persistent bleeding after 12 weeks' gestation, multiple pregnancy, poorly controlled thyroid disease, pregnancy-induced hypertension or pre-eclampsia, diabetes or gestational diabetes, and other diseases that could interfere with participation.	Norway	Pre-pregnancy self-reported. Early pregnancy: measured at 12-24 weeks' gestation	Exercise	Aerobic dance/strength training class 2-3 times per week (one hour each) for a minimum of 12 weeks. Advised 30 minutes of moderate self-imposed physical activity on the remaining week-days (recorded in a personal training diary).	Infant birth weight, gestational age at delivery, Apgar score.	105<br>83 (79.0%)

For peer review only

Table S1. cont.

Author (Year)	Period	Original inclusion/exclusion criteria	Country	Method to measure GWG	Intervention	Intervention Detail	Outcome	Original i-WIP N	N (%) in this analysis
Harrison *et al*. (2013)	Not described	Inclusion criteria: 12-15 weeks' gestation, BMI ≥25 or ≥23 kg/m^2 if high-risk ethnicity (Polynesian, Asian, and African populations) or ≥30 kg/m^2, increased risk of GDM identified by risk prediction tool, agreement to complete an OGTT at 28 weeks' gestation. Exclusion criteria: multiple pregnancies, type 1 or 2 diabetes, a BMI ≥45 kg/m^2, pre-existing chronic medical conditions, and non-English-speaking	Australia	Pre-pregnancy: self-reported Early pregnancy: measured at 12-15 weeks' gestation.	Mixed	Behaviour change lifestyle intervention with four individual sessions based on the Social Cognitive Theory, at 14-16, 20, 24, and 28 weeks' gestation. Provided with simple healthy eating and physical activity messages. Behavioural change strategies were practised to identify short-term goals, and increase self-efficacy and self-monitoring. Goals were determined individually by the participants. Self-monitoring strategies included pedometers and the use of weight gain charts.	GWG, GDM screening	238	187 (78.6%)
Jeffries *et al*. (2009)	July and October 2007	Inclusion criteria: ≤14 weeks' gestation. Exclusion criteria were: age <18 years or >45 years, type 1 or type 2 diabetes mellitus, multiple pregnancy, or non-English speaking.	Australia	Pre-pregnancy self-reported. Early pregnancy: measured at 14 weeks' gestation	Mixed	Advised on optimal GWG based on IOM 1990 guidelines. Provided with a personalised weight measurement card and instructed to record their weight at 16, 20, 24, 28, 30, 32 and 34 weeks' gestation.	GWG, adherence to IOM guidelines (1990)	286	236 (82.5%)
Khaledan *et al*. (2010)	Not described	Inclusion criteria: Singleton, healthy pregnancy. Exclusion criteria: chronic disease, multiple pregnancy, complications, risk of preterm delivery, hypertension, severe anaemia, type 1 diabetes with poor control, morbid obesity, low maternal weight, history of completely sedentary lifestyle, heavy smoker.	Iran	Pre-pregnancy: na. Early pregnancy: Not described	Exercise	Aerobic (stepping) and flexibility exercise session three times per week (30-45 minutes each) for eight weeks.	Type of delivery, cause of caesarean section, maternal and fetal weight, birth weight and gestational age	39	37 (94.9%)
Khoury *et al*. (2005)	June 1999 to November 2001	Inclusion criteria: 17-20 weeks' gestation, singleton, healthy pregnancy, non-smoker (or stopped ≥5 years previously), 21-38 years, BMI 19-32 kg/m^2. Exclusion criteria: following a vegetarian or Mediterranean diet, immigrant to Norway from non-Western country, high-risk pregnancy, complications in a previous pregnancy, on-going hyperemesis gravidarum or bleeding after gestational week 12	Norway	Pre-pregnancy: na. Early pregnancy: measured at 17-20 week's gestation	Diet	Dietary advice from dietitian at inclusion, 24, 30 and 36 weeks' gestation. Cholesterol-lowering diet; promoted fish, low-fat meats and dairy products, oils, whole grains, fruits, vegetables, and legumes from gestational week 17-20 until delivery.	Maternal, cord, and neonatal plasma lipids and pregnancy-related complications, including hypertensive complications, preterm delivery, and intrauterine growth restriction, birth weight and maternal weight gain.	290	198 (68.3%)

For peer review only

Table S1. cont.

Author (Year)	Period	Original inclusion/exclusion criteria	Country	Method to measure GWG	Intervention	Intervention Detail	Outcome	Original i-WIP N / N (%) in this analysis
Nascimento *et al.* (2011)	August 2008 to October 2009	Inclusion criteria: overweight or obese, age >18 years, 14-24 weeks' gestation. Exclusion criteria: multiple gestations, exercising regularly, conditions that contraindicate exercise	Brazil	Pre-pregnancy self-reported. Early pregnancy: measured at 14-24 weeks' gestation	Exercise	One exercise class per week (40 minutes each), under the guidance of a trained physical therapist. Light-intensity to moderate-intensity exercises. Advised to exercise at home five times per week.	GWG, maternal arterial blood pressure and perinatal outcome, perception of quality of life.	82 / 77 (93.9%)
Oostdam *et al.* (2012)	November 2007 and April 2010	Inclusion criteria: obese or overweight and at least one of the following characteristics: history of macrosomia; history of GDM; or first-grade relative with type 2 diabetes. Exclusion criteria: >20 weeks' gestation; <18 years; inadequate knowledge of Dutch language; GDM; hypertension; alcohol abuse; drug abuse; use of any medication that affects insulin secretion or insulin sensitivity; chronic disease that could prevent the woman from implementing the study protocol.	Netherlands	Pre-pregnancy self-reported. Early pregnancy: measured at 15-20 weeks' gestation	Exercise	Aerobic and strength exercise classes (FitFor2 Study). Two days per week (60 minutes each), under supervision of physiotherapist, from 14-20 weeks' gestation to end of pregnancy.	Fasting blood glucose, insulin, HbA1c; body weight, BMI, daily physical activity, birth weight and fetal growth.	124 / 75 (60.5%)
Petrella *et al.* (2014)	April 2011 to October 2011	Inclusion criteria: Pre-pregnancy BMI ≥25 kg/m^2, >18 years and singleton pregnancy. Exclusion criteria: twin pregnancy, chronic diseases (i.e. diabetes mellitus, chronic hypertension, untreated thyroid diseases), previous GDM, smoker, previous bariatric surgery, regular physical activity, dietary supplements or herbal products known to affect body weight, other medical conditions that might affect body weight, and plans to deliver outside the centre	Italy	Pre-pregnancy self-reported. Early pregnancy: measured at 12 weeks' gestation	Mixed	Diet, physical activity and counselling about GWG. Dietary advice: low glycaemic index diet, three main meals and three snacks (1500 kcal/day). Physical activity advice: 3 days per week of moderate intensity activity (30 minutes each), monitored using pedometer. One-hour counselling session about appropriate GWG.	Excessive weight gain, gestational diabetes mellitus, gestational hypertension and preterm delivery.	61 / 58 (95.1%)

For peer review only

Table S1. cont.

Author (Year)	Period	Original inclusion/exclusion criteria	Country	Method to measure GWG	Intervention	Intervention Detail	Outcome	Original i-WIP N	N (%) in this analysis
Phelan *et al*. (2011)	2006 to 2008	Inclusion criteria: 10 - 16 week's gestation, BMI 19.8 - 40 kg/m², non-smoking, >18 years, fluency in English, access to a telephone, and a singleton pregnancy. Exclusion criteria: self-reported major health or psychiatric diseases, weight loss during pregnancy, or a history of >3 miscarriages	USA	Pre-pregnancy self-reported. Early pregnancy: measured at 10-16 weeks' gestation	Mixed	Behavioural intervention with diet and physical activity advice (Fit for Delivery Study). One face-to-face visit to discuss appropriate GWG, physical activity (30 min of walking most days of the week), and calorie goals (20 kcal/kg), with daily self-monitoring of eating, exercise, and weight. Provided with weight scales, food records, pedometers, weekly reminders, personalised graphs of weight gained with feedback, 3 supportive phone calls from the dietitian. If gained over or under weight gain guidelines received 2 additional supportive phone calls per month with meal plans and goals until weight gains returned to appropriate amounts.	GWG and the proportion who returned to pre-gravid weights by 6 months' postpartum.	401	395 (98.5%)
Poston *et al*. (2015)	March 2009 to June 2014	Inclusion criteria: >16 years, BMI ≥30 kg/m², singleton pregnancy, 15 - 18(+6) weeks' gestation. Exclusion criteria: unwilling or unable to give informed consent; underlying disorders, including a pre-pregnancy diagnosis of essential hypertension, diabetes, renal disease, systemic lupus erythematosus, antiphospholipid syndrome, sickle- cell disease, thalassaemia, coeliac disease, thyroid disease, and current psychosis; or currently being prescribed metformin.	UK	Pre-pregnancy: na. Early pregnancy: measured at 15-18 week's gestation	Mixed	Behavioural intervention with diet and physical activity advice (UPBEAT Study). One individual interview plus one group/individual session per week (60 minutes each) for 8 weeks. Advised to self-monitor, identify, and problem-solve of barriers to behaviour change and enlist social support. Dietary intervention aimed to promote a healthy pattern of eating and low glycaemic index diet. Exercise advice focused on incremental increases in walking from a pedometer- assessed baseline, tailored to pre-existing activities.	Gestational diabetes and large-for-gestational-age infants (≥90th customised birth weight centile)	1554	378 (24.3%)

For peer review only

Table S1. cont.

Author (Year)	Period	Original inclusion/exclusion criteria	Country	Method to measure GWG	Intervention	Intervention Detail	Outcome	Original i-WIP N / N (%) in this analysis
Rauh *et al*. (2013)	February 2010 and August 2011	Inclusion criteria: >18 years; singleton pregnancy; <18 weeks' gestation; BMI ≥18.5 kg/m², and sufficient German. Exclusion criteria: any condition preventing physical activity, GDM and uncontrolled chronic diseases that may affect weight development like thyroid dysfunction or psychiatric diseases	Germany	Pre-pregnancy self-reported. Early pregnancy: measured at 10-16 weeks' gestation	Mixed	Individual counselling sessions on nutrition, physical activity, and GWG monitoring (FeLIPO Study). Twice during pregnancy; 20th (60 minutes) and 30th (30 minutes) week of gestation. Provided with general healthy lifestyle information; prompted to self-monitor behaviour by recording diet, physical activity, and weight gain using weight gain charts; and to set behavioural goals based on the baseline situation (BMI, diet, physical activity) and individual preferences.	Weight gain in excess of IOM recommendations, and weight retention at 4 months' postpartum	250 / 231 (92.4%)
Renault *et al*. (2014)	March 2009 to March 2012	Inclusion criteria: >18 years, singleton pregnancy, normal scan in weeks 11-14, <16 weeks' gestation, sufficient Danish. Exclusion criteria: multiple pregnancy, pre-gestational diabetes, or other serious diseases limiting level of physical activity, previous bariatric surgery, or alcohol or drug abuse.	Denmark	Pre-pregnancy self-reported. Early pregnancy: measured at 11-16 weeks' gestation	Mixed	Physical activity intervention with or without dietary intervention (TOP Study). Physical activity advice: daily step count of 11,000, monitored with pedometer. Diet: hypocaloric (1200-1675 kcal), low-fat, Mediterranean-style diet and contact with an experienced dietitian every 2 weeks, alternating between outpatient visits and phone contacts (measurement of weight, encouragement, and correcting advice on the diet).	GWG, pregnancy complications and delivery complications.	425 / 370 (87.1%)
Sagedal *et al*. (2017)	September 2009 and February 2013	Inclusion criteria: Nulliparous, singleton pregnancy, ≤20 weeks' gestation, pre-pregnancy BMI of ≥19 kg/m^2, sufficient in Norwegian or English, and provided signed, informed consent. Exclusion criteria: pre-existing diabetes, disabilities precluding participation in a physical fitness programme, continued substance abuse, or planned relocation outside of the study area before delivery.	Norway	Pre-pregnancy self-reported. Early pregnancy: measured at ≤20 weeks' gestation	Mixed	Lifestyle intervention with diet and physical activity components (Norwegian Fit for Delivery Study). Dietary advice provided by telephone, with one initial consultation and follow-up 4–6 weeks later (20 minutes). Dietary advice aimed to increase awareness of food choices. GWG based on pre-pregnancy BMI provided. Physical activity: access to exercise classes at a local gym twice/week (60 minutes). Provided with booklets, access to study website, invitation to one cooking class and to an evening meeting.	GWG, birth weight of term infants, maternal fasting glucose levels at 30 weeks of gestation, maternal percentage of fat, incidence of operative deliveries, postpartum weight	606 / 576 (95.0%)

Table S1. cont.

Author (Year)	Period	Original inclusion/exclusion criteria	Country	Method to measure GWG	Intervention	Intervention Detail	Outcome	Original i-WIP N / N (%) in this analysis
Stafne *et al*. (2012)	April 2007 to June 2009 and October 2007 to January 2009	Inclusion criteria: Residing within 30 min drive from the hospital and available to attend weekly appointment, ≥18 years, singleton pregnancy. Exclusion criteria: High-risk pregnancies, diseases that could interfere with participation.	Norway	Pre-pregnancy: na. Early pregnancy: measured at 18-22 week's gestation	Exercise	Exercise sessions, once per week for 12 weeks (60 minutes each), in groups of 8–15 women, instructed by a physiotherapist. Offered between 20 and 36 weeks of pregnancy. Included aerobic activity, strength training and balance exercises. Women encouraged to follow a written home exercise program at least twice per week (45 minutes each).	Self-reported lumbopelvic pain and sick leave due to lumbopelvic pain.	855 / 699 (81.8%)
Vinter *et al*. (2011)	October 2007 to October 2010	Inclusion criteria: 18–40 years, 10–14 weeks' gestation, BMI 30–45 kg/m^2. Exclusion criteria: chronic medical conditions, prior serious obstetric complications, alcohol or drug abuse, non-Danish speaking, multiple pregnancy, positive OGTT.	Denmark	Pre-pregnancy: self-reported. Early pregnancy: measured at 10-14 week's gestation	Mixed	Dietary counselling and physical activity (The LiP Study - Lifestyle in Pregnancy). Four dietary counselling by dietitians at 15, 20, 28, and 35 weeks' gestation. Dietary advice was based on the official Danish recommendations, and aimed to limit GWG to 5 kg. Provided with free full-time membership in a fitness centre for 6 months, with exercise classes once per week (60 minutes each) with physiotherapists. Encouraged to be moderately physically active every day (30–60 minutes per day), provided with a pedometer to motivate and improve daily activity.	GWG, preeclampsia, pregnancy-induced hypertension, GDM, caesarean section, macrosomia, and admission to neonatal intensive care unit.	304 / 292 (96.1%)
Vitolo *et al*. (2011)	January 2007 to May 2008	Inclusion criteria: 10-29 weeks' gestation. Exclusion criteria: positive HIV test previous diagnosis of diabetes, hypertension, anaemia, any conditions preventing women from undertaking, exercise in pregnancy, and >35 years	Brazil	Pre-pregnancy: self-reported. Early pregnancy: collected in first trimester (method not described)	Diet	Dietary counselling provided according to nutritional needs. Underweight women advised to increase energy density of the diet, include two high-energy snacks and fruit daily. Well-nourished women advised to consume six portions fruit and vegetables and restrict energy dense foods. Overweight women were advised on meal timing, portion sizes, to restrict energy dense and processed foods.	GWG, gestational diabetes, pre-eclampsia, infant birth and premature delivery (<37wks), 1st and 5th min Apgar score, head circumference, type of delivery, birth weight <2500g	315 / 268 (85.1%)

For peer review only

Table S1. cont.

Author (Year)	Period	Original inclusion/exclusion criteria	Country	Method to measure GWG	Intervention	Intervention Detail	Outcome	Original i-WIP N / N (%) in this analysis
Walsh *et al*. (2012)	January 2007 to January 2011	Inclusion criteria: Secundigravid women, previous macrosomic infant (≥4 kg). Exclusion criteria: underlying medical disorders, including a previous history of gestational diabetes, use of any drugs, unable to provide full informed consent, <18 years, >18 weeks' gestation, and multiple pregnancy.	Ireland	Pre-pregnancy: na. Early pregnancy: measured at 12-15 week's gestation	Diet	One dietary education session (2 hours), with the research dietitian, at 15 weeks' gestation, groups of 2-6 women. Reinforcement of diet at 28 and 34 weeks' gestation. Advised on a low glycaemic index diet, and general healthy eating guidelines for pregnancy. Received written resources about low glycaemic index diet.	Birth weight, GWG, glucose intolerance.	800 / 517 (64.6%)
Wolff *et al*. (2008)	Not described	Inclusion criteria: non-diabetic, Caucasian, BMI >30 kg/m^2, 15 weeks' gestation. Exclusion criteria: smoker, <18 or >45 years, multiple pregnancy, medical complications known to affect fetal growth adversely or to contraindicate limitation of weight gain.	Denmark	Pre-pregnancy: self-reported. Early pregnancy: measured at 15 week's gestation	Diet	Dietary consultations with a dietitian, 10 times during pregnancy (60 minutes each). Advised to eat a healthy diet according to the official Danish dietary recommendations. Aim to restrict GWG to 6–7kg.	GWG, pregnancy-induced increases in insulin, leptin, and glucose. Weight retentions at 4 weeks' postpartum	59 / 55 (93.2%)

GWG: gestational weight gain; BMI: body mass index; GDM; gestational diabetes; OGTT: oral glucose tolerance test; IOM: Institute of Medicine

For peer review only

Table S2. Global classification of risk of bias on study level for trials included

Study Team	Item 1: Randomisation	Item 2: Allocation concealment	Item 3: Blinding of participants	Item 4: Blinding of outcome Assessment	Item 5: Incomplete outcome data	Item 6: Selective reporting	Global Risk of bias
Althuizen *et al*. (2013)	⊕	⊕	?	⊕	⊕	?	Low/Medium
Baciuk *et al*. (2008)	⊕	⊕	⊖	⊕	⊕	⊕	Low/Medium
Bogaerts *et al*. (2013)	⊕	?	⊖	⊖	⊕	⊕	High
Haakstad *et al*. (2011)	⊕	⊕	?	⊕	⊕	⊖	Low/Medium
Harrison *et al*. (2013)	⊕	⊕	?	⊕	⊕	⊕	Low/Medium
Jeffries *et al*. (2009)	⊕	⊕	?	⊕	⊕	⊕	Low/Medium
Khaledan *et al*. (2010)	⊕	?	⊖	⊖	⊕	⊕	High
Khoury *et al*. (2005)	⊕	⊕	?	⊕	⊕	⊕	Low/Medium
Nascimento *et al*. (2011)	⊕	⊕	⊖	⊖	⊕	⊕	High
Oostdam *et al*. (2012)	⊕	⊕	⊖	⊕	⊖	⊕	High
Petrella *et al*. (2014)	⊕	⊖	⊖	⊖	⊕	⊖	High
Phelan *et al*. (2011)	⊕	⊕	?	⊕	⊕	⊕	Low/Medium
Poston *et al*. (2015)	?	?	⊖	⊖	?	?	High
Rauh *et al*. (2013)	⊕	⊕	⊖	⊖	⊕	?	High
Renault *et al*. (2014)	⊕	⊕	⊖	⊖	⊕	⊖	High
Sagedal *et al*. (2016)	⊕	⊕	?	⊕	?	?	Low/Medium
Stafne *et al*. (2012)	⊕	⊕	⊖	⊖	⊕	⊕	High
Vinter *et al*. (2011)	⊕	⊕	⊖	⊖	⊕	⊖	High
Vitolo *et al*. (2011)	⊕	⊖	⊖	⊕	⊕	⊖	High
Walsh *et al*. (2012)	⊕	⊕	⊖	?	⊕	⊖	Low/Medium
Wolff *et al*. (2008)	⊕	⊕	?	⊖	⊖	⊕	High

Legend

⊕	⊖	?
Low	High	Unclear

Table S3. Individual Study Results – Gestational Weight Gain per Week

		Intervention			Control			Mean Difference (95% CI)	*P**
**Study name**	**Education**	**N**	**Mean**	**SD**	**N**	**Mean**	**SD**		
Althuizen *et al*. (2013)	Lower	49	0.39	0.34	49	0.47	0.26	-0.08 (-0.02, 0.04)	0.205
	Higher	40	0.47	0.20	49	0.43	0.27	0.04 (-0.06, 0.15)	0.391
Baciuk *et al*. (2008)	Lower	29	0.48	0.23	31	0.51	0.25	-0.04 (-0.16, 0.09)	0.561
	Higher	4	0.41	0.19	4	0.52	0.31	-0.11 (-0.55, 0.33)	0.557
Bogaerts *et al*. (2013)	Lower	73	0.31	0.26	40	0.41	0.25	-0.10 (-0.21, 0.01)	0.064
	Higher	60	0.29	0.24	23	0.42	0.28	-0.14 (-0.27, -0.01)	0.044
Haakstad *et al*. (2011)	Lower	7	0.47	0.15	6	0.39	0.18	0.08 (-0.13, 0.29)	0.411
	Higher	34	0.48	0.17	36	0.51	0.17	-0.03 (-0.11, 0.05)	0.432
Harrison *et al*. (2013)	Lower	56	0.41	0.18	56	0.46	0.25	-0.05 (-0.13, 0.03)	0.197
	Higher	40	0.44	0.19	35	0.47	0.26	-0.02 (-0.13, 0.08)	0.668
Jeffries *et al*. (2009)	Lower	42	0.45	0.18	32	0.47	0.14	-0.03 (-0.12, 0.05)	0.498
	Higher	83	0.43	0.17	79	0.46	0.16	-0.03 (-0.08, 0.03)	0.401
Khaledan *et al*. (2010)	Lower	11	0.61	0.26	14	0.73	0.33	-0.12 (-0.38, 0.13)	0.336
	Higher	6	0.55	0.23	6	0.51	0.17	0.04 (-0.22, 0.30)	0.736
Khoury *et al*. (2005)	Lower	21	0.56	0.20	14	0.54	0.17	0.01 (-0.12, 0.15)	0.822
	Higher	74	0.52	0.19	89	0.57	0.17	-0.05 (-0.10, 0.01)	0.108
Nascimento *et al*. (2011)	Lower	34	0.37	0.22	39	0.40	0.22	-0.03 (-0.13, 0.07)	0.562
	Higher	3	0.40	0.07	1	0.80	na	-0.40 (-0.73, -0.07)	0.035
Oostdam *et al*. (2012)	Lower	28	0.41	0.34	24	0.32	0.20	0.09 (-0.07, 0.25)	0.245
	Higher	10	0.40	0.21	13	0.44	0.21	-0.04 (-0.22, 0.14)	0.680
Petrella *et al*. (2014)	Lower	25	0.22	0.29	27	0.33	0.22	-0.11 (-0.26, 0.04)	0.137
	Higher	5	0.34	0.12	1	0.35	na	-0.01 (-0.39, 0.37)	0.950
Phelan *et al*. (2011)	Lower	27	0.50	0.25	36	0.46	0.19	0.04 (-0.07, 0.15)	0.469
	Higher	170	0.48	0.16	162	0.52	0.19	-0.03 (-0.07, 0.01)	0.094
Poston *et al*. (2015)	Lower	113	0.26	0.20	120	0.27	0.22	0.00 (-0.06, 0.06)	0.853
	Higher	66	0.20	0.21	79	0.26	0.20	-0.06 (-0.14, 0.01)	0.116
Rauh *et al*. (2013)	Lower	64	0.45	0.15	43	0.45	0.18	-0.03 (-0.11, 0.04)	0.904
	Higher	89	0.43	0.13	35	0.47	0.15	-0.07 (-0.13, -0.01)	0.120
Renault *et al*. (2014)	Lower	56	0.38	0.22	28	0.48	0.22	-0.09 (-0.20, 0.01)	0.082
	Higher	186	0.35	0.21	100	0.39	0.19	-0.04 (-0.09, 0.01)	0.169
Sagedal *et al*. (2017)	Lower	89	0.54	0.21	93	0.57	0.21	-0.03 (-0.09, 0.03)	0.339
	Higher	199	0.48	0.20	195	0.52	0.19	-0.04 (-0.08, 0.00)	0.037
Stafne *et al*. (2012)	Lower	31	0.53	0.15	38	0.59	0.23	-0.06 (-0.16, 0.03)	0.178
	Higher	339	0.51	0.17	291	0.52	0.17	-0.01 (-0.03, 0.02)	0.617
Vinter *et al*. (2011)	Lower	59	0.31	0.25	69	0.36	0.22	-0.06 (-0.15, 0.03)	0.212
	Higher	85	0.33	0.20	79	0.38	0.22	-0.05 (-0.12, 0.02)	0.158
Vitolo *et al*. (2011)	Lower	79	0.41	0.18	83	0.45	0.21	-0.04 (-0.10, 0.02)	0.199
	Higher	61	0.42	0.15	45	0.49	0.22	-0.07 (-0.15, 0.00)	0.053
Walsh *et al*. (2012)	Lower	60	0.46	0.16	62	0.52	0.21	-0.06 (-0.13, 0.01)	0.077
	Higher	188	0.49	0.16	207	0.53	0.18	-0.04 (-0.08, -0.01)	0.021
Wolff *et al*. (2008)	Lower	26	0.26	0.16	27	0.40	0.24	-0.14 (-0.25, -0.02)	0.021
	Higher	0†	na		2	0.38	0.24	na	na

Mean difference is calculated as intervention minus control
* P-value is independent-sample t-test between intervention and control
† T-test cannot be computed because at least one of the groups is empty.
Lower education: secondary education or less. Higher education: at least some tertiary education

For peer review only

Table S4. Comparison of maternal characteristics among women included and excluded in the IPD meta-analysis.

	Included		Excluded		*P**
	N		N		
**Early pregnancy weight** (kg) [a]	5183	78.60 (18.56)	4814	87.78 (51.06)	0.002
**Pre-pregnancy weight** (kg) [a]	5183	76.06 (18.74)	2398	85.82 (123.03)	0.030
**Height** (cm) [a]	5183	166.07 (7.03)	6697	169.30 (63.03)	<0.001
**Pre-pregnancy BMI** (kg/m^2) [a]	5183	27.57 (6.55)	2615	27.31 (12.56)	<0.001
**Gestational age** (weeks)					
**Baseline** [c]	5183	14.5 (4.0 - 32.0)	4478	14.0 (11.0 – 17.0)	<0.001
**Final follow-up** [c]	5183	36.6 (24.0 - 44.0)	6995	38.0 (35.0 – 41.0)	<0.001
**Delivery** [c]	5172	39.7 (26.0 - 44.0)	7007	40.0 (38.85 – 41.0)	0.022
**Infant birth weight** (g) [a]	5164	3531.73 (547.90)	7026	3701.22 (1479.82)	<0.001
**Maternal age** (years) [a]	5183	29.90 (4.97)	7041	30.80 (8.36)	<0.001
**Ethnicity**					
Caucasian [b]	3725	3291 (88.3)	6415	5588 (87.1)	<0.001
Asian [b]		84 (0.02)		231 (0.04)	
Black [b]		123 (0.03)		462 (0.08)	
Central/South American [b]		88 (0.02)		47 (0.01)	
Middle East (including Iran & Turkey) [b]		63 (0.01)		13 (0.00)	
Other [b]		76 (0.02)		74 (0.01)	

Data are presented as [a] mean (SD) [b] n (%) and [c] mean (range of minimum and maximum).

*P-value: T-tests and chi-square

For peer review only

Figure S1. Assessment of small study effects on trials in IPD meta-analysis of low educational attainment and gestational weight gain per week, controlling for BMI and intervention.

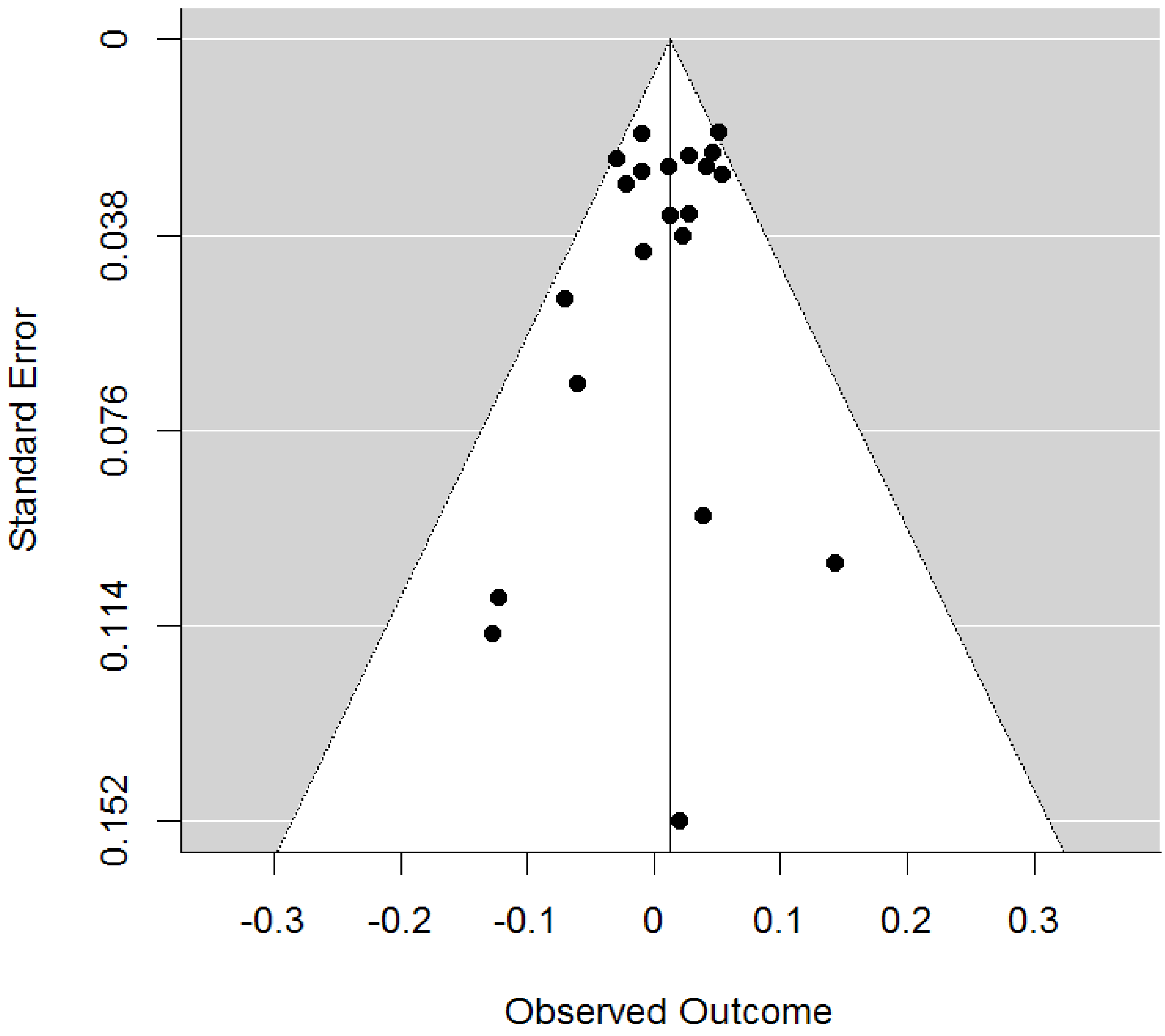

Figure S2. Forest plot of the IPD meta-analysis of low educational attainment (treatment effect [TE]) and gestational weight gain per week, controlling for BMI and intervention.

Study	TE	seTE	95%-CI	Weight
Althuizen, 2012	-0.01	0.0412	-0.01 [-0.09; 0.07]	3.3%
Baciuk, 2008	0.04	0.0930	0.04 [-0.14; 0.22]	0.8%
Bogaerts, 2012	0.02	0.0383	0.02 [-0.05; 0.10]	3.7%
Haakstad, 2011	-0.07	0.0506	-0.07 [-0.17; 0.03]	2.3%
Harrison, 2013	0.03	0.0339	0.03 [-0.04; 0.09]	4.5%
Jeffries, 2009	0.01	0.0247	0.01 [-0.04; 0.06]	7.1%
Khaledan, 2010	0.14	0.1020	0.14 [-0.06; 0.34]	0.6%
Khoury, 2005	0.01	0.0342	0.01 [-0.05; 0.08]	4.5%
Nascimento, 2011	-0.13	0.1157	-0.13 [-0.35; 0.10]	0.5%
Oostdam, 2012	-0.06	0.0670	-0.06 [-0.19; 0.07]	1.4%
Petrella, 2013	-0.12	0.1089	-0.12 [-0.34; 0.09]	0.6%
Phelan, 2011	-0.01	0.0256	-0.01 [-0.06; 0.04]	6.8%
Poston, (unpub)	0.04	0.0247	0.04 [-0.01; 0.09]	7.1%
Rauh, 2013	0.03	0.0227	0.03 [-0.02; 0.07]	7.9%
Renault, 2013	0.05	0.0262	0.05 [0.00; 0.11]	6.6%
Sagedal, (unpub)	0.05	0.0179	0.05 [0.02; 0.09]	10.2%
Stafne, 2012	0.05	0.0219	0.05 [0.00; 0.09]	8.2%
Vinter, 2011	-0.02	0.0282	-0.02 [-0.08; 0.03]	5.9%
Vitolo, 2011	-0.03	0.0230	-0.03 [-0.08; 0.02]	7.7%
Walsh, 2012	-0.01	0.0183	-0.01 [-0.05; 0.03]	10.0%
Wolff, 2008	0.02	0.1523	0.02 [-0.28; 0.32]	0.3%
**Random effects model**			**0.01 [0.00; 0.03]**	**100.0%**

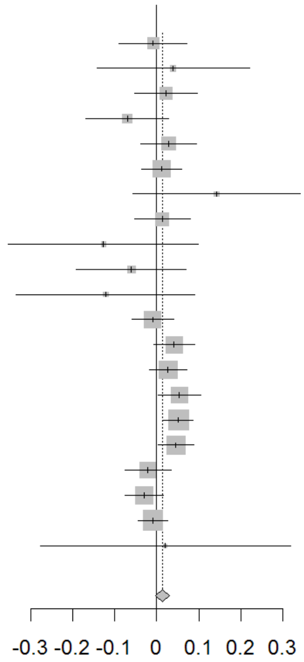

For peer review only

Appendix 1. Identification and selection of studies in individual participant data (IPD) meta-analysis for the i-WIP Database

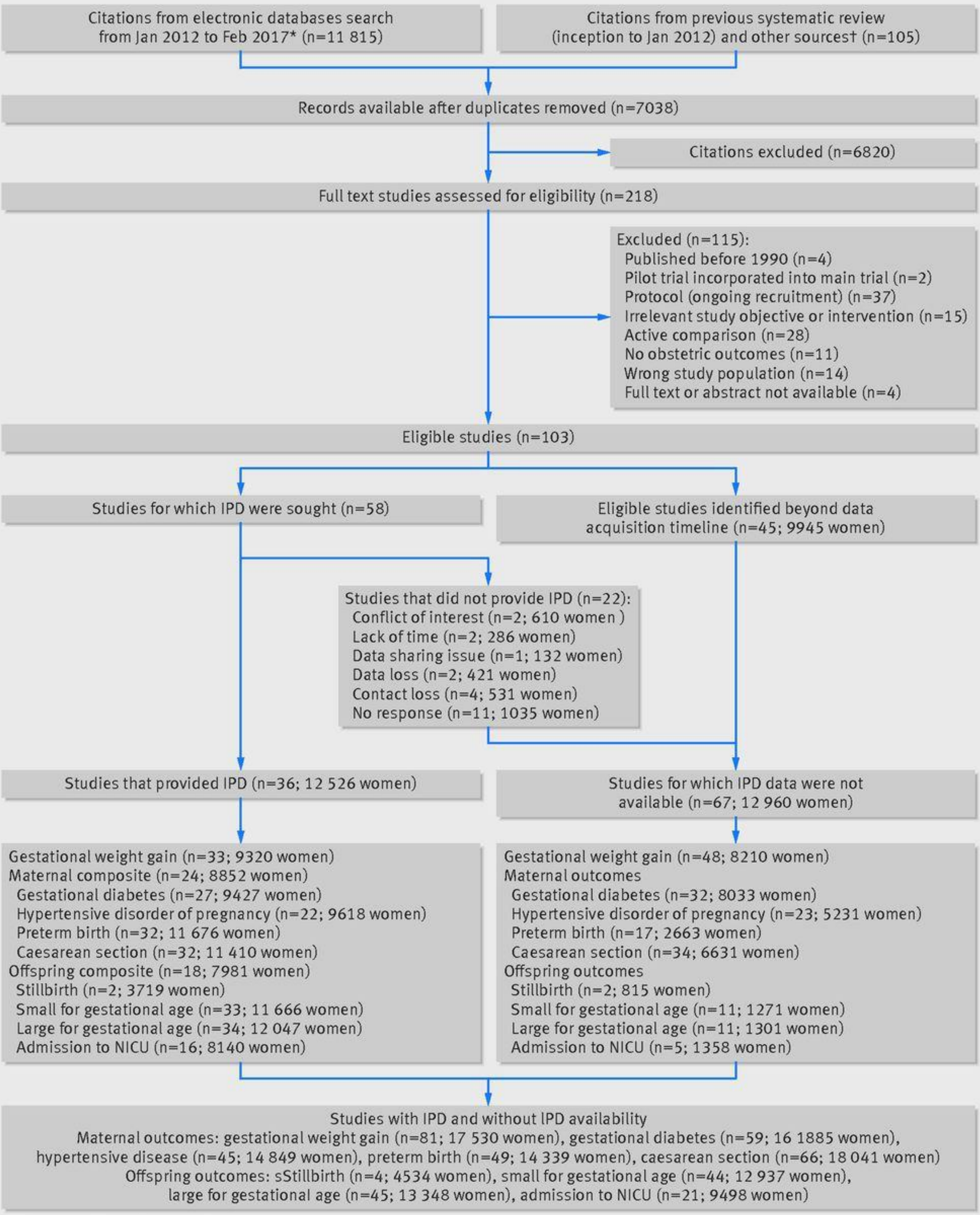

NICU=neonatal intensive care unit
*Database search was updated in October 2013 (9359 records), March 2015 (3551 records), January 2016 (1373 records), and February 2017 (1547 records)
†Other sources: reference search, personal communication, and Google search

Appendix 2. Search strategy for identification of randomised trials on lifestyle interventions in pregnancy and maternal and offspring outcomes (original search strategy from BMJ 2017;358:j3119)

Search strategy for Medline via Ovid

Item	Term
1	Pregnancy/
2	pregnan*.tw.
3	Gravidity/
4	gravid*.tw.
5	gestation*.tw.
6	Pregnant Women/
7	pregnant wom#n.tw.
8	(child adj3 bearing).tw.
9	childbearing.tw.
10	matern*.tw.
11	or/1-10
12	Weight Gain/ph [Physiology]
13	weight gain*.tw.
14	Weight Loss/ph [Physiology]
15	weight loss*.tw.
16	weight change*.tw.
17	Obesity/dh, me, ph, pc, px, th [Diet Therapy, Metabolism, Physiology, Prevention & Control, Psychology, Therapy]
18	obes*.tw.
19	Adiposity/ph [Physiology]
20	adipos*.tw.
21	Overweight/dh, me, ph, pc, px, th [Diet Therapy, Metabolism, Physiology, Prevention & Control, Psychology, Therapy]
22	overweight*.tw.
23	Body Mass Index/
24	bmi.tw.
25	or/12-24
26	exp Randomized Controlled Trial/
27	"randomized controlled trial".pt.
28	"controlled clinical trial".pt.
29	(random$ or placebo$).tw,sh.
30	((singl$ or double$ or triple$ or treble$) and (blind$ or mask$)).tw,sh.
31	single-blind method/
32	double-blind method/
33	or/26-32
34	11 and 25 and 33
35	exp Animals/
36	(rat$ or mouse or mice or hamster$ or animal$ or dog$ or cat$ or bovine or sheep or lamb$).af.
37	35 or 36
38	Humans/
39	human$.tw,ot,kf.
40	37 or 38
41	37 not (37 and 40)
42	34 not 41

Appendix 3. Institute of Medicine Guidelines for gestational weight gain per week in trimester 2 and 3, and extrapolated to trimester 1

Participant level data: A measured, early-pregnancy weight was available for 4,888 participants. Early-pregnancy weight was estimated using self-reported pre-pregnancy weight for the 295 participants missing this variable. Estimated weight gain per week from pre-pregnancy to early-pregnancy was calculated by the following formula, using only participants with both pre- and early-pregnancy weight, n = 3001:

$$\textit{Weight gain from pre- to early-pregnancy} = \frac{\textit{Early-pregnancy weight} - \textit{pre-pregnancy weight}}{\textit{Baseline GA}}$$

Mean weight gain from pre- to early-pregnancy was 0.175 kg/week. We used the following formula to estimate early-pregnancy weight for the 295 participants missing this variable:

Early-pregnancy weight = (Baseline GA*0.175 kg) + pre-pregnancy weight

We used pre-pregnancy weight to categorise participants according to BMI. Self-reported pre-pregnancy weight was available for 3,296 participants, and estimated using early-pregnancy weight for the remaining 1,887 participants. We used the following formula to estimate pre-pregnancy weight:

Pre-pregnancy weight = Early-pregnancy weight – (Baseline GA*0.175 kg)

Total weight gain was calculated by subtracting early-pregnancy weight from final follow-up weight. Weekly GWG was calculated using the following formula:

$$\textit{Weight Gain per week} = \frac{\textit{Total } GWG}{\textit{Final follow-up GA} - \textit{Baseline GA}}$$

Trimester 2 and 3 GWG/week was required for comparison with IOM 2009 guidelines. This was calculated using a four-step approach;

1. Trimester 1 GWG/week guidelines were extrapolated from the IOM guidelines(15) .

For peer review only

BMI Category	Total GWG (IOM Guidelines)			Rate of weight gain for Trimester 2 and 3 (IOM Guidelines)		
	Inadequate	Adequate	Excess	Mean	Range	
**Underweight**	<12.5	12.5 - 18.0	>18.0	0.51	0.44	0.58
**Normal**	<11.5	11.5 - 16.0	>16.0	0.42	0.35	0.50
**Overweight**	<7.0	7.0 - 11.5	>11.5	0.28	0.23	0.33
**Obese**	<5.0	5.0 - 9.0	>9.0	0.22	0.17	0.27

2. The number of weeks in trimester 1 that the participant was enrolled in the intervention was calculated by subtracting the baseline GA from 13. If the participant enrolled in the study at greater than 13 weeks, trimester 1 weeks were set as 0. The number of weeks in trimester 2 and 3 that the participant was enrolled in the intervention were calculated by subtracting the 13 from the final follow-up GA.
3. The rate of weight gain for Trimester 1 was calculated:

(*Rate of weight gain for Trimester 2 and 3*)**27= Total weight gain for Trimester 2 and 3* ***(A)***

Total GWG [ranges for adequate] - Total weight gain for Trimester 2 and 3 [ranges] = Total weight gain for Trimester 1 [ranges] ***(B)***

$$\frac{\text{Lower range Trimester 1 weight gain} + \text{Upper range Trimester 1 weight gain}}{2} = \text{Mean weight gain for Trimester 1 } \textbf{(C)}$$

$$\frac{\text{Total weight gain for Trimester 1 [mean and ranges]}}{13} = \text{Rate of weight gain for Trimester 1 } \textbf{(D)}$$

BMI Category	(A) Total weight gain for Trimester 2 and 3			Total weight gain for Trimester 1			(D) Rate of weight gain for Trimester 1		
	Mean	Range		(C) Mean	(B) Range		Mean	Range	
**Underweight**	13.77	11.88	15.66	1.48	0.62	2.34	0.11	0.05	0.18
**Normal**	11.34	9.45	13.50	2.28	2.05	2.50	0.18	0.16	0.19
**Overweight**	7.56	6.21	8.91	1.69	0.79	2.59	0.13	0.06	0.20
**Obese**	5.94	4.59	7.29	1.06	0.41	1.71	0.08	0.03	0.13

For peer review only

4. The contribution of trimester 1 weight gain to total GWG was estimated using the mean trimester 1 GWG/week (above) and the following equation:

$$\text{T1 weight gain} = (\text{T1 weight gain per week for BMI}) * (\text{T1 weeks in intervention})$$

5. Trimester 2 and 3 weight gain per week was calculated using the following equation:

$$\textit{T2 and T3 weight gain per week} = \frac{\textit{Total weight gain} - \textit{T1 weight gain}}{\textit{Weeks in T2 and T3}}$$

For peer review only

S2 Appendix. PRISMA-IPD Checklist of items to include when reporting a systematic review and meta-analysis of individual participant data (IPD)

PRISMA-IPD Section/topic	Item No	Checklist item	Reported on page
Title			
Title	1	Identify the report as a systematic review and meta-analysis of individual participant data.	1
Abstract			
Structured summary	2	Provide a structured summary including as applicable:	5-6
		**Background**: state research question and main objectives, with information on participants, interventions, comparators and outcomes.	
		**Methods**: report eligibility criteria; data sources including dates of last bibliographic search or elicitation, noting that IPD were sought; methods of assessing risk of bias.	
		**Results**: provide number and type of studies and participants identified and number (%) obtained; summary effect estimates for main outcomes (benefits and harms) with confidence intervals and measures of statistical heterogeneity. Describe the direction and size of summary effects in terms meaningful to those who would put findings into practice.	
		**Discussion:** state main strengths and limitations of the evidence, general interpretation of the results and any important implications.	
		**Other:** report primary funding source, registration number and registry name for the systematic review and IPD meta-analysis.	

For peer review only

Introduction			
Rationale	3	Describe the rationale for the review in the context of what is already known.	8-9
Objectives	4	Provide an explicit statement of the questions being addressed with reference, as applicable, to participants, interventions, comparisons, outcomes and study design (PICOS). Include any hypotheses that relate to particular types of participant-level subgroups.	9
Methods			
Protocol and registration	5	Indicate if a protocol exists and where it can be accessed. If available, provide registration information including registration number and registry name. Provide publication details, if applicable.	9
Eligibility criteria	6	Specify inclusion and exclusion criteria including those relating to participants, interventions, comparisons, outcomes, study design and characteristics (e.g. years when conducted, required minimum follow-up). Note whether these were applied at the study or individual level i.e. whether eligible participants were included (and ineligible participants excluded) from a study that included a wider population than specified by the review inclusion criteria. The rationale for criteria should be stated.	9
Identifying studies - information sources	7	Describe all methods of identifying published and unpublished studies including, as applicable: which bibliographic databases were searched with dates of coverage; details of any hand searching including of conference proceedings; use of study registers and agency or company databases; contact with the original research team and experts in the field; open adverts and surveys. Give the date of last search or elicitation.	9 and Protocol paper attached
Identifying studies - search	8	Present the full electronic search strategy for at least one database, including any limits used, such that it could be repeated.	9 and Protocol paper attached

For peer review only

Study selection processes	9	State the process for determining which studies were eligible for inclusion.	9 and Protocol paper attached
Data collection processes	10	Describe how IPD were requested, collected and managed, including any processes for querying and confirming data with investigators. If IPD were not sought from any eligible study, the reason for this should be stated (for each such study).<br><br>If applicable, describe how any studies for which IPD were not available were dealt with. This should include whether, how and what aggregate data were sought or extracted from study reports and publications (such as extracting data independently in duplicate) and any processes for obtaining and confirming these data with investigators.	9 and Protocol paper attached
Data items	11	Describe how the information and variables to be collected were chosen. List and define all study level and participant level data that were sought, including baseline and follow-up information. If applicable, describe methods of standardising or translating variables within the IPD datasets to ensure common scales or measurements across studies.	10
IPD integrity	A1	Describe what aspects of IPD were subject to data checking (such as sequence generation, data consistency and completeness, baseline imbalance) and how this was done.	10
Risk of bias assessment in individual studies.	12	Describe methods used to assess risk of bias in the individual studies and whether this was applied separately for each outcome. If applicable, describe how findings of IPD checking were used to inform the assessment. Report if and how risk of bias assessment was used in any data synthesis.	10
Specification of outcomes	13	State all treatment comparisons of interests. State all outcomes addressed and define them in detail. State whether they were pre-specified for the review and, if applicable, whether they were primary/main or secondary/additional	10-11

For peer review only

and effect measures		outcomes. Give the principal measures of effect (such as risk ratio, hazard ratio, difference in means) used for each outcome.	
Synthesis methods	14	Describe the meta-analysis methods used to synthesise IPD. Specify any statistical methods and models used. Issues should include (but are not restricted to):<br>• Use of a one-stage or two-stage approach.<br>• How effect estimates were generated separately within each study and combined across studies (where applicable).<br>• Specification of one-stage models (where applicable) including how clustering of patients within studies was accounted for.<br>• Use of fixed or random effects models and any other model assumptions, such as proportional hazards.<br>• How (summary) survival curves were generated (where applicable).<br>• Methods for quantifying statistical heterogeneity (such as I^2 and τ^2).<br>• How studies providing IPD and not providing IPD were analysed together (where applicable).<br>• How missing data within the IPD were dealt with (where applicable).	11-12
Exploration of variation in effects	A2	If applicable, describe any methods used to explore variation in effects by study or participant level characteristics (such as estimation of interactions between effect and covariates). State all participant-level characteristics that were analysed as potential effect modifiers, and whether these were pre-specified.	12
Risk of bias across studies	15	Specify any assessment of risk of bias relating to the accumulated body of evidence, including any pertaining to not obtaining IPD for particular studies, outcomes or other variables.	10
Additional analyses	16	Describe methods of any additional analyses, including sensitivity analyses. State which of these were pre-specified.	12
Results			
Study	17	Give numbers of studies screened, assessed for eligibility, and included in the systematic review with reasons for	13 and Table 1

For peer review only - http://bmjopen.bmj.com/site/about/guidelines.xhtml

selection and IPD obtained		exclusions at each stage. Indicate the number of studies and participants for which IPD were sought and for which IPD were obtained. For those studies where IPD were not available, give the numbers of studies and participants for which aggregate data were available. Report reasons for non-availability of IPD. Include a flow diagram.	
Study characteristics	18	For each study, present information on key study and participant characteristics (such as description of interventions, numbers of participants, demographic data, unavailability of outcomes, funding source, and if applicable duration of follow-up). Provide (main) citations for each study. Where applicable, also report similar study characteristics for any studies not providing IPD.	13 and Table S1
IPD integrity	A3	Report any important issues identified in checking IPD or state that there were none.	13 and Table S2
Risk of bias within studies	19	Present data on risk of bias assessments. If applicable, describe whether data checking led to the up-weighting or down-weighting of these assessments. Consider how any potential bias impacts on the robustness of meta-analysis conclusions.	13 and Table S2
Results of individual studies	20	For each comparison and for each main outcome (benefit or harm), for each individual study report the number of eligible participants for which data were obtained and show simple summary data for each intervention group (including, where applicable, the number of events), effect estimates and confidence intervals. These may be tabulated or included on a forest plot.	14 and Table S3
Results of syntheses	21	Present summary effects for each meta-analysis undertaken, including confidence intervals and measures of statistical heterogeneity. State whether the analysis was pre-specified, and report the numbers of studies and participants and, where applicable, the number of events on which it is based.	14-15. Tables 2-5
		When exploring variation in effects due to patient or study characteristics, present summary interaction estimates for each characteristic examined, including confidence intervals and measures of statistical heterogeneity. State whether	

For peer review only

		the analysis was pre-specified. State whether any interaction is consistent across trials.	
		Provide a description of the direction and size of effect in terms meaningful to those who would put findings into practice.	
Risk of bias across studies	22	Present results of any assessment of risk of bias relating to the accumulated body of evidence, including any pertaining to the availability and representativeness of available studies, outcomes or other variables.	13
Additional analyses	23	Give results of any additional analyses (e.g. sensitivity analyses). If applicable, this should also include any analyses that incorporate aggregate data for studies that do not have IPD. If applicable, summarise the main meta-analysis results following the inclusion or exclusion of studies for which IPD were not available.	15
Discussion			
Summary of evidence	24	Summarise the main findings, including the strength of evidence for each main outcome.	16
Strengths and limitations	25	Discuss any important strengths and limitations of the evidence including the benefits of access to IPD and any limitations arising from IPD that were not available.	18-19
Conclusions	26	Provide a general interpretation of the findings in the context of other evidence.	19
Implications	A4	Consider relevance to key groups (such as policy makers, service providers and service users). Consider implications for future research.	17-18
Funding			

For peer review only

Funding	27	Describe sources of funding and other support (such as supply of IPD), and the role in the systematic review of those providing such support.	6

For peer review only

For peer review only

BMJ Open

Impact of maternal education on response to lifestyle interventions to reduce gestational weight gain - Individual participant data meta-analysis

Journal:	*BMJ Open*
Manuscript ID	bmjopen-2018-025620.R2
Article Type:	Research
Date Submitted by the Author:	30-May-2019
Complete List of Authors:	O'Brien, Eileen; University College Dublin, UCD Perinatal Research Centre<br>Segurado, Ricardo; University College Dublin, CSTAR, School of Public Health, Physiotherapy and Population Science<br>Geraghty, Aisling; University College Dublin, UCD Perinatal Research Centre<br>Alberdi , Goiuri; University College Dublin, UCD Perinatal Research Centre<br>Rogozinska, Ewelina; Queen Mary's University London, Women's Health Research Unit<br>Astrup, Arne; Univesity of Copenhagen, Department of Nutrition, Exercise and Sports,<br>Barakat Carballo , Rubenomar; Universidad Politecnica de Madrid, Facultad de Ciencias de la Actividad Fısica y del Deporte<br>Bogaerts, Annick; University of Leuven, Department of Development and Regeneration KU Leuven; UC Leuven-Limburg, Faculty of Health and Social Work<br>Cecatti, Jose; University of Campinas, Obstetrics and Gynecology<br>Coomarasamy, Arri; University of Birmingham,<br>de Groot, Christianne; VU University Medical Center, Obstetrics and Gynaecology<br>Devlieger, R.; Universitaire Ziekenhuizen Leuven, Department of Obstetrics and Gynecology; KUL University of Leuven, Department of Development and Regeneration<br>Dodd, Jodie; The Unversity of Adelaide, Obstetrics and Gynaecology; Discipline of Obstetrics and Gynaecology<br>El Beltagy, Nermeen ; Alexandria University, Department of Obstetrics and Gynecology<br>Facchinetti , Fabio ; Azienda Ospedaliera Universitaria Policlinico di Modena, Obstetric Unit, Mother Infant Department<br>Geiker , Nina ; Rigshospitalet, Clinical Nutrition Research<br>Guelfi, Kym; The University of Western Australia, School of Human Sciences<br>Haakstad, Lene; Norges idrettshogskole, Department of Sports Medicine<br>Harrison, Cheryce; Monash University School of Public Health and Preventive Medicine, Monash Centre for Health Research and Implementation<br>Hauner, Hans; Technische Universitat Munchen Zentralinstitut fur Ernahrungs- und Lebensmittelforschung, Else Kroener-Fresenius-Center

for Nutritional Medicine
Jensen, DM; Syddansk Universitet Det Sundhedsvidenskabelige Fakultet, Department of Endocrinology
Khan, Khalid; Queen Mary, University of London, Multidisciplinary Evidence Synthesis Hub (mEsh); Queen Mary University of London, Women's Health Research Unit
Kinnunen, Tarja; University of Tampere, School of Health Sciences
Luoto, Riitta; Tampereen Yliopisto, Health Sciences
Willem Mol, Ben; University of Adelaide, Obstetrics/Gynaecology
Mørkved, Siv; NTNU Fakultet for ingeniorvitenskap og teknologi Trondheim, Department of Public Health and General Practice; Trondheim University Hospital, Clinical Services
Motahari-Tabari, Narges ; Mazandaran University of Medical Science, Faculty of nursing and midwifery, Midwifery Department
Owens, Julie; The University of Adelaide, Discipline of Obstetrics & Gynaecology
Perales, Maria; Universidad Politecnica de Madrid, Facultad de Ciencias de la Actividad Fısica y del Deporte
Petrella, Elisabetta; Universita degli Studi di Modena e Reggio Emilia Dipartimento di Educazione e Scienze Umane, Mother-Infant Department
Phelan, Suzanne; California Polytechnic State University College of Science and Mathematics, Kinesiology Department
Poston, Lucilla; Kings College, London, Division of Reproduction and Endocrinology
Rauh, Kathrin; Technische Universitat Munchen Zentralinstitut fur Ernahrungs- und Lebensmittelforschung, Else Kroener-Fresenius-Center for Nutritional Medicine; Competence Centre for Nutrition (KErn)
Rayanagoudar, Girish ; Queen Mary University of London, Women's Health Research Unit
Renault , KM ; University of Copenhagen, Departments of Obstetrics and Gynecology
Ruifrok, A; VU medisch centrum ICaR-VU Onderzoeksinstituut, Department of Obstetrics and Gynaecology; Academic Medical Centre, Department of Obstetrics and Gynecology
Sagedal, Linda; Sorlandet sykehus HF, Department of Obstetrics and Gynecology
Salvesen, Kjell; Trondheim University Hospital, Dept of Obstetrics and gynaecology; Norwegian University of Science and Technology, Department of Laboratory Medicine Children's and Women's Health
Scudeller, Tania; Universidade Federal de Sao Paulo, Department of Management and Health Care
Shen, Gary; University of Manitoba College of Medicine, Department of Internal Medicine
Shub, Alexis; University of Melbourne, Obstetrics and Gynaecology
Stafne, Signe; NTNU Fakultet for ingeniorvitenskap og teknologi Trondheim, Department of Public Health and General Practice; Sankt Olavs Hospital Universitetssykehuset i Trondheim, Clinical Services
Surita, Fernanda; University of Campinas, Obstetrics & Gynecology
Thangaratinam, Shakila; Queen Mary University of London, Women's Health Research Unit; Queen Mary University of London, Multidisciplinary Evidence Synthesis Hub (mEsh)
Tonstad, Serena ; Oslo universitetssykehus Ulleval, Department of Obstetrics and Gynecology
van Poppel, Mireille; VU University Medical Center, Department of Public and Occupational Health
Vinter, Christina ; Syddansk Universitet Det Sundhedsvidenskabelige Fakultet, Department of Obstetrics and Gynecology
Vistad, Ingvild ; Sorlandet Hospital Kristiansand , Obstetric and Gynegology
Yeo, SeonAe ; University of North Carolina at Chapel Hill, School of Nursing

	McAuliffe, Fionnuala; University College Dublin, UCD Perinatal Research Centre
**Primary Subject Heading**:	Nutrition and metabolism
Secondary Subject Heading:	Epidemiology, Nutrition and metabolism, Public health
Keywords:	Pregnancy, Lifestyle interventions, Socioeconomic status, Inequalities, Nutrition < TROPICAL MEDICINE

SCHOLARONE™
Manuscripts

Title: Impact of maternal education on response to lifestyle interventions to reduce gestational weight gain - Individual participant data meta-analysis

Author List:

E.C. O'Brien [1], R. Segurado [2], A.A. Geraghty [1], G. Alberdi [1], E. Rogozińska [3, 4], A. Astrup [5], R. Barakat Carballo [6], A. Bogaerts [7, 8, 9], J.G. Cecatti [10], A. Coomarasamy [11], C.J. de Groot [12], R. Devlieger [13, 14], J.Dodd [15], N.El Beltagy [16], F. Facchinetti [17], N.R. Geiker [18], K.J Guelfi [19], L. Haakstad [20], C.Harrison [21], H. Hauner [22], D.M. Jensen [23], K.S. Khan [4,26], T. I. Kinnunen [27], R. Luoto [28], B. W. Mol [29], S. Mørkved [30, 31], N. Motahari-Tabari [32], J. Owens [15], M. Perales, [6], E. Petrella [17], S. Phelan [33], L. Poston [34], K. Rauh [35,36], G. Rayanagoudar [26], K.M. Renault [37, 38], A.E. Ruifrok [12,39], L.R. Sagedal [40], K.Å Salvesen [41,42], T.T. Scudeller [43], G.X. Shen [44], A. Shub [45], S.N. Stafne [30, 31], F. Surita [10], S. Thangaratinam [4,26], S. Tonstad [46], M.N. van Poppel [47], C. Vinter [24], I. Vistad [40], S. Yeo [48], i-WIP (International Weight Management in Pregnancy) Collaborative Group, F.M. McAuliffe [1]*

Affiliations

1 UCD Perinatal Research Centre, Obstetrics & Gynaecology, UCD School of Medicine, University College Dublin, National Maternity Hospital, Dublin, Ireland

2 Centre for Support and Training in Analysis and Research (CSTAR), School of Public Health, Physiotherapy and Sports Science, University College Dublin, Dublin, Ireland

3 Women's Health Research Unit, Barts and The London School of Medicine and Dentistry, Queen Mary University of London, London, UK..

4 Multidisciplinary Evidence Synthesis Hub (mEsh), Barts and The London School of Medicine and Dentistry, Queen Mary University of London, London, UK.

5 Department of Nutrition, Exercise and Sports, Copenhagen University, Copenhagen, Copenhagen, Denmark.

6 Facultad de Ciencias de la Actividad Fısica y del Deporte-INEF, Universidad

For peer review only - http://bmjopen.bmj.com/site/about/guidelines.xhtml

Politecnica de Madrid, Madrid, Spain.

7 Department of Development and Regeneration KU Leuven, University of Leuven, Leuven, Belgium,

8 Faculty of Health and Social Work, research unit Healthy Living, UC Leuven-Limburg, Belgium

9 Faculty of Medicine and Health Sciences, Centre for Research and Innovation in Care (CRIC), University of Antwerp, Belgium.

10 Department of Obstetrics and Gynecology, School of Medical Sciences, University of Campinas (UNICAMP), Campinas, Brazil.

11 School of Clinical and Experimental Medicine, College of Medical and Dental Sciences, University of Birmingham, Birmingham, UK.

12 Department of Obstetrics and Gynaecology, Faculty of Medicine, VU University Medical Center, Amsterdam, The Netherlands.

13 Department of Obstetrics and Gynecology, University Hospitals Leuven, Belgium

14 Department of Development and Regeneration, KUL University of Leuven, Belgium

15 Discipline of Obstetrics and Gynaecology, School of Paediatrics and Reproductive Health, The University of Adelaide, Adelaide, Australia.

16 Department of Obstetrics and Gynecology, Alexandria University, Alexandria, Egypt.

17 Mother-Infant Department, University of Modena and Reggio Emilia, Modena, Italy.

18 Clinical Nutritional Research Unit, Copenhagen University Hospital Herlev-Gentofte, Gentofte, Denmark.

19 School of Human Sciences, The University of Western Australia, Perth, Australia.

20 Norwegian School of Sport Sciences, Department of Sports Medicine, Oslo, Norway.

21 Monash Centre for Health Research and Implementation -MCHRI, School of Public Health Monash University, Melbourne, Australia.

22 Else Kroener-Fresenius-Center for Nutritional Medicine, Klinikum rechts der Isar, Technische Universität München, Munich, Germany.

For peer review only

For peer review only - http://bmjopen.bmj.com/site/about/guidelines.xhtml

23 Department of Endocrinology, Odense University Hospital, University of Southern Denmark, Odense, Denmark.

24 Department of Gynecology and Obstetrics, Odense University Hospital, University of Southern Denmark, Odense, Denmark.

25 Department of Clinical Research, Faculty of Health Sciences, University of Southern Denmark

26 Women's Health Research Unit, Barts and The London School of Medicine and Dentistry, Queen Mary University of London, London, UK.

27 Faculty of Social Sciences/ Health Sciences, University of Tampere, Tampere, Finland.

28 School of Health Sciences, University of Tampere, Tampere, Finland.

29 Robinson Institute, School of Paediatrics and Reproductive Health, University of Adelaide, Adelaide, Australia.

30 Department of Public Health and General Practice, Faculty of Medicine, Norwegian University of Science and Technology, Trondheim, Norway.

31 Clinical Services, St. Olavs Hospital, Trondheim University Hospital, Trondheim, Norway.

32 Mazandaran University of Medical Science, Faculty of nursing and midwifery, Midwifery Department. Msc, consulting in Midwifery and Msc Sport of Science.

33 Kinesiology Department, California Polytechnic State University, San Luis Obispo, USA.

34 Division of Women's Health, Women's Health Academic Centre, King's College London, St. Thomas' Hospital, London, UK.

35 Else Kroener-Fresenius-Center for Nutritional Medicine, Chair of Nutritional Medicine, Technische Universität München, Munich, Germany.

36 Competence Centre for Nutrition (KErn), Freising, Germany.

37 Departments of Obstetrics and Gynecology, Hvidovre Hospital, University of

For peer review only

Copenhagen, Copenhagen, Denmark.
38 Obstetric Clinic, Juliane Marie Centret, Rigshospitalet, University of Copenhagen, Copenhagen, Denmark.
39 Department of Obstetrics and Gynecology, Academic Medical Centre, Amsterdam, The Netherlands.
40 Department of Obstetrics and Gynecology, Sorlandet Hospital, Kristiansand, Norway.
41 Department of Obstetrics and Gynaecology, St. Olavs Hospital, Trondheim University Hospital, Trondheim, Norway
42 Department of Laboratory Medicine Children's and Women's Health, Faculty of Medicine, Norwegian University of Science and Technology, Trondheim, Norway.
43 Department of Management and Health Care, Physical Therapy Course, Federal University of São Paulo (UNIFESP), Brazil
44 Department of Internal Medicine, University of Manitoba, Winnipeg, Canada.
45 Department of Obstetrics and Gynaecology, University of Melbourne, Melbourne, Australia.
46 Department of Obstetrics and Gynecology, Oslo University Hospital, Oslo, Norway.
47 Department of Public and Occupational Health, Amsterdam Public Health research institute, VU University Medical Center, Amsterdam, The Netherlands.
Institute of Sport Science, University of Graz, Austria.
48 University of North Carolina at Chapel Hill, School of Nursing, Chapel Hill, NC, USA.

Corresponding author:

Fionnuala M McAuliffe; Email: fionnuala.mcauliffe@ucd.ie

Key Words: Pregnancy; Lifestyle interventions; socioeconomic status; inequalities; nutrition

Word count: 3,737

For peer review only - http://bmjopen.bmj.com/site/about/guidelines.xhtml

Abstract

Objectives: To identify if maternal educational attainment is a prognostic factor for gestational weight gain (GWG), and to determine the differential effects of lifestyle interventions (diet-alone, exercise-alone or mixed approach) on gestational weight gain, stratified by educational attainment.

Design: Individual participant data meta-analysis using the previously established International Weight Management in Pregnancy (i-WIP) Collaborative Group database (https://iwipgroup.wixsite.com/collaboration). PRISMA-IPD Statement guidelines were followed.

Data sources: Major electronic databases, from inception to February 2017.

Eligibility criteria: Randomised controlled trials on diet and physical activity based interventions in pregnancy. Maternal educational attainment was required for inclusion and was categorised as higher education (≥ tertiary) or lower education (≤ secondary).

Risk of bias: Cochrane risk of bias tool was used.

Data synthesis: Principle measures of effect were odds ratio and regression coefficient.

Results: Of the 36 randomised controlled trials in the i-WIP database, 21 trials and 5,183 pregnant women were included. Women with lower educational attainment had an increased risk of excessive (OR:1.182; 95% CI:1.008-1.385; P:0.039) and inadequate weight gain (OR:1.284; 95% CI:1.045-1.577; *P*: 0.017). Among women with lower education, diet-alone interventions reduced risk of excessive weight gain (OR:0.515; 95% CI:0.339-0.785; *P*:0.002) and inadequate weight gain (OR:0.504; 95% CI:0.288-0.884; *P*:0.017), and reduced kg/week gain (*B*:-0.055; 95% CI:-0.098–-0.012; *P*:0.012). Mixed interventions reduced risk of excessive weight gain for women with lower education (OR:0.735; 95% CI:0.561-0.963; *P*:0.026). Among women with high education, diet-alone interventions reduced risk of excessive weight gain (OR:0.609; 95% CI:0.437-0.849; *P*:0.003), and mixed interventions reduced kg/week gain (*B*:-0.053; 95% CI:-0.069–-0.037; *P*:<0.001). Exercise-alone interventions did not impact GWG when stratified by education.

For peer review only - http://bmjopen.bmj.com/site/about/guidelines.xhtml

Conclusions: Pregnant women with lower education are at an increased risk of excessive and inadequate GWG. Diet-alone interventions seem the most appropriate choice for these women, and additional support through mixed interventions may also be beneficial.

Article Summary

Strengths and limitations of this study

- A major strength is the use of individual patient data to carry out meta-analysis from multiple nutrition and lifestyle interventions.
- The pooling of individual patient data allows for the estimation of the differential effects of education and lifestyle interventions on weight gain across the included cohorts with greater power than individual randomised controlled trials.
- We analysed and reported the findings using gestational weight gain per week, which enables comparison of studies that used different gestational ages when total weight gain was measured.
- A limitation is that most of the cohorts included are from wealthy countries; therefore, the results may not be representative of populations living in low and middle-income countries.
- Although the results were statistically significant, the effect sizes are small, thus the relevance of these findings in the clinical setting must be considered.

For peer review only

For peer review only - http://bmjopen.bmj.com/site/about/guidelines.xhtml

Introduction

According to the 2030 Sustainable Development Goals, the global health agenda is calling for improvements in maternal health and nutrition, equal opportunities for education, and reduced social, economic and political inequalities (1). The current situation is that maternal social, economic and educational backgrounds are key determinants of health and lifestyle behaviours among pregnant women across the world. Pregnancy and birth outcomes are negatively influenced by socioeconomic and deprivation factors (2–6). Prevalence of smoking, alcohol consumption and pre-pregnancy body mass index (BMI) are greater among women of lower socioeconomic status, while diet quality and physical activity participation are reduced within this group (7–9).

GWG is also influenced by socioeconomic status, with a recent systematic review finding that those of lower educational attainment are less likely to gain weight within the guidelines' recommended ranges for GWG, as described by the Institute of Medicine (IOM) (10). Excessive GWG increases the risk of adverse maternal and neonatal outcomes, including gestational diabetes, caesarean section, hypertension, large-for-gestational-age and stillbirth, while inadequate GWG is associated with increased risk of small-for-gestational-age infant (11,12). Moreover, greater GWG is associated with weight retention postpartum (13) and elevated BMI up to 15 years post-pregnancy (14), a potential factor exacerbating current global trends of overweight and obesity (15).

Since GWG outside of suggested guidelines is more prevalent among disadvantaged groups, it would seem wise that support and interventions that enable equitable opportunities to optimise health are prioritised. However, there is limited knowledge regarding GWG among women of various socioeconomic position, as recognised by the IOM (16). Pregnancy has been described as a stimulus for positive behaviour change (17) and is a time when women are in regular contact with healthcare professionals (18). Thus, a lifestyle intervention in pregnancy should, in theory, have a greater impact during this time than any other time in a woman's life. While there are many examples of lifestyle and behaviour

For peer review only - http://bmjopen.bmj.com/site/about/guidelines.xhtml

interventions in the literature that aim to manage weight gain during pregnancy (19); few interventions stratify analyses by socioeconomic status.

The International Weight Management in Pregnancy (i-WIP) Individual Patient Data Collaborative Network was established in 2013 to determine the differential effects of weight management interventions in pregnancy on maternal weight gain and pregnancy outcomes (20). The collaboration between researchers from 16 countries has resulted in a repository of data from 36 randomised trials with over 60 variables and individual data from more than 12,500 pregnant women. The i-WIP found that diet and physical activity based interventions reduce GWG and lower the odds of caesarean section (21). However, sub-analysis was not conducted among women of various educational attainments, thus compounding our lack of understanding for the type of pregnancy intervention that has the greatest effect among those of low educational attainment.

This research aims to use IPD meta-analysis using data from the previously established i-WIP Network to: (1) identify if educational attainment is a prognostic factor for GWG; (2) determine the differential effects of weight management interventions in pregnancy on maternal weight gain, stratified by educational attainment.

Methods

The checklist outlined in the Preferred Reporting Items for Systematic reviews and Meta-Analysis of Individual Participant Data (PRISMA-IPD) statement was followed (22). Full information about the search strategy is available in the i-WIP protocol paper and the i-WIP primary publication (20,21). In brief, major electronic databases from October 2013 to March 2015 were searched, with updated literature searches completed by the i-WIP team in February 2017 and more recently in June 2018. Given the logistics and resources involved in collating IPD, trials identified in the most recent literature search have not yet been added to the i-WIP dataset. Eligible studies were randomised controlled trials (RCT) that evaluated any dietary or lifestyle interventions with potential to influence maternal and fetal outcomes

For peer review only - http://bmjopen.bmj.com/site/about/guidelines.xhtml

related to maternal weight (20,23). The women in the study were required to be ≥18 years of age with a singleton pregnancy and they must have been enrolled to one of the following interventions; diet based, physical activity based, mixed approach (diet, physical activity, GWG monitoring and/or behaviour change components), or standard care (23). In total, 11,920 studies were identified, of which 36 studies were included (12,526 participants) (20,21,24). The flow-chart for the identification and selection of studies has been published (21) (Appendix 1). A full search strategy from the original i-WIP IPD for one database is presented in Appendix 2. All individual trials included in this IPD meta-analysis had ethical approval from relevant ethical committees.

IPD integrity and risk of bias assessment

The i-WIP Network performed a range of checks on the variables used during the analysis. The randomisation ratio, baseline characteristics and method of analysis provided by the original authors to the i-WIP Network were compared to the published information. Any discrepancies were queried and rectified as necessary with input from the original authors. As per the protocol, the i-WIP Network assessed the quality of each trial to evaluate the integrity of the randomisation and follow-up procedure. The Cochrane risk of bias tool was used and studies were classified as high risk of bias if they scored highly in at least one of following domains: randomisation, allocation concealment, blinding of outcome assessment, or incomplete outcome data. To be classified as low risk of bias, all items must have scored as low risk. In terms of publication bias, a funnel plot was created to assess the small study effects on trials in the IPD meta-analysis of educational attainment and gestational weight gain per week. (Figure S1).

Data items and specification of outcomes

The i-WIP protocol pre-specified that socioeconomic status would be evaluated as a potential prognostic variable for GWG, as secondary analysis to the i-WIP project (20). The exposure variable was maternal educational attainment, as this was the most commonly

For peer review only

used measure of socioeconomic status among studies in the i-WIP network. It has also been reported as a stable measure of individual socioeconomic status (25). Educational attainment was classified as less than or equal to secondary education (including no education, less than primary, completed primary, some secondary, or completed secondary education), or tertiary education (including vocational training, some university, bachelor degree, post-graduate degree or higher).

Intervention type was classified as diet based, physical activity based, or mixed approach (diet, physical activity, GWG monitoring and/or behaviour change components) (23), and participants were either exposed to the intervention or to standard care.

The primary outcome was maternal weight gain in pregnancy. The outcome was examined using two methods: according to the IOM 2009 guidelines (16); and GWG per week as a continuous variable. To calculate GWG, data were required for the following variables; baseline gestational age, final follow-up gestational age, pre-pregnancy or early-pregnancy weight, final follow-up weight and height. The method described in Appendix 3 was used to calculate GWG per week.

Specification of effect measures

The principle measures of effect were odds ratio (for analysis according to the IOM guidelines) and regression coefficient (for analysis of GWG as a continuous variable).

To assess the magnitude of study variability in the outcomes, we calculated an intra-class correlation coefficient (ICC) from the fitted models, interpretable as an index of heterogeneity such as the I^2. The intra-class correlation coefficient for the study (ICC_{study}) relates to the proportion of variability due to cohort-to-cohort variation, and the intra-class correlation coefficient for the intervention ($ICC_{intervention}$) relates to the proportion of variability in the intervention treatment effect. In the logistic models for categorical GWG the residual variance was replaced by $\pi^2/3$.

For peer review only - http://bmjopen.bmj.com/site/about/guidelines.xhtml

Synthesis methods

For comparisons of characteristics across education groups, we used independent sample *t* tests for continuous data and χ^2 tests for categorical data. For the categorical outcomes of inadequate, adequate, and excessive GWG, we used generalised linear mixed models: a multinomial logistic regression with a generalised logit link function. For the continuous outcomes of GWG (kg/week), we used a linear mixed model with an identity link function, assuming the outcome was approximately normally distributed. The models analysing the effect of education included random effects for each cohort. Random effects were specified for both the intercept and the slope of the intervention effect, allowing them to be correlated. The between-study variance in each effect was calculated by the ICC. Separate models were created for educational attainment strata to analyse the effect of each intervention type, as fixed effects. Linear mixed models were adjusted for factors known to influence GWG (BMI, maternal age, parity, ethnicity), in addition to the main effects and interaction of education and the interventions. In model 1 we adjusted for pre-pregnancy maternal BMI (continuous GWG model only). In model 2 we adjusted for pre-pregnancy maternal BMI (continuous GWG model only), maternal age, parity and ethnicity. As the IOM category definitions are linked to pre-pregnancy BMI category, this outcome intrinsically "adjusts" for maternal BMI; we can consider model 1 as "unadjusted", and model 2 as an "adjusted" model. All statistical analyses were performed using IBM SPSS Statistics for Windows, Version 24.0 (IBM, Armonk, NY).

Exploration of variation in effects

While controlling for between-study variation in outcomes and intervention effects as random effects, we explored the influence of educational attainment on the intervention effects as pre-specified fixed main effects and an interaction term. Tertiary education and the control (non-intervention) condition were set as the reference levels, and results for IOM categories used the adequate weight gain category as the reference outcome group.

For peer review only

Additional analyses

We proceeded with our model-building in a step-wise fashion, aiming at a pre-specified target of fixed effects as above, and both random intercept and intervention effects. A step-wise approach is often pragmatic in developing a mixed-effects model as small study size and uncertain variance/covariance structure can lead to models failing to converge. We therefore combined the addition of random and fixed effects to a model in the following stages: 1) empty model including no fixed predictors, only a random intercept; 2) education and intervention main effects and interaction added to the model, and controlling for pre-pregnancy maternal BMI (Model 1); 3) random effect of intervention was explored to account for potential population or intrinsic variability in the effect of the interventions on GWG; 4) additional potential confounders were added as individual-level fixed effects to either model 2 or 3: maternal age, parity, ethnicity (Model 2). Our strategy was to remove fixed interaction effects or random effects if not statistically significant, or if model fitting became problematic. Furthermore, within each study, independent sample *t*-tests were carried out to explore the differences in weight gain per week by education level.

Patient involvement

No patients were involved in the design of this study. However we plan to share and disseminate the findings with one of the parent participant involvement groups (the ROLO Families Advisory Committee).

Results

Protocol, IPD obtained and Study characteristics

The PRISMA-IPD checklist is included as a supplementary file. The original i-WIP IPD dataset contained 12,240 participants from 36 studies. Participants were excluded if they were missing data relating to the main outcomes or met the exclusion criteria (Table 1).

For peer review only

For peer review only - http://bmjopen.bmj.com/site/about/guidelines.xhtml

In total, 7,057 women were excluded from the analysis, while 5,183 remained. Participants that met the inclusion criteria were enrolled to 21 RCTs (26–46). The study characteristics, methods used to measure GWG, intervention details and outcome measures of the eligible trials are summarised in Table S1.

IPD integrity and Risk of bias assessment

No issues were identified in the integrity of the IPD. The global classification of risk of bias classified 12 studies as high risk and 9 as low/medium risk (Table S2). In terms of publication bias, the funnel plot (Figure S1) demonstrated an even distribution of effect estimates and standard error, indicating low bias in publications.

Results of individual studies

For the majority of studies, we did not observe a significant difference in GWG/week (kg) between participants in the intervention and control, split by educational attainment (Table S3). Within four of the studies (28,34,41,45), women with tertiary education enrolled to the intervention had significantly less weight gain/week. One study found that women with secondary education or less enrolled to the intervention had significantly less weight gain/week (46). The forest plot (Figure S2) demonstrates the results of individual studies for the IPD meta-analysis of low educational attainment and gestational weight gain per week, controlling for BMI and intervention. A comparison of maternal characteristics among women included and excluded in the IPD meta-analysis (Table S4) demonstrated some differences across the populations.

Results of syntheses

Of the 5,183 participants in this analysis, 3,273 (63.1%) had achieved at least some tertiary education, while 1,910 (36.9%) had, at most, completed secondary education. Within the secondary education group, 931 (48.8%) were enrolled to the control arm, and 653 (34.2%), 186 (9.7%) and 140 (7.3%) were enrolled to the intervention arm of mixed, diet, and exercise

For peer review only

trials, respectively. Within the tertiary education group, 1,531 (46.8%) were enrolled to the control arm of their respective RCT, while 1,023 (31.2%), 323 (9.9%) and 396 (12.1%) were enrolled to the intervention arm of mixed, diet and exercise trials, respectively.

Compared to women with tertiary education, women with lower education levels had significantly higher early-pregnancy weight and pre-pregnancy BMI, and were significantly shorter in height (Table 2). Fewer women with tertiary education had a BMI >30 kg/m^2, compared to women in the secondary education group (26.8% *vs.* 46.3%, *P* <0.001). Women within the tertiary education group were older, had lower parity and were more likely to be of Caucasian ethnicity compared to those with lower education.

Mean ± SD GWG per week and percentage of participants within each of the IOM categories, stratified by educational attainment and BMI category are shown in Table 3. Figure 1 displays GWG (kg/week), according to pre-pregnancy BMI, intervention and educational attainment.

The unadjusted linear mixed-models found that women with lower educational attainment gained significantly more weight per week (kg) compared to those with tertiary education (B = 0.014), however this association was not significant when the model was adjusted for maternal age, parity and ethnicity (Table 4a). The ICC_{study} was 0.1% and $ICC_{intervention}$ was 0.0%. Women of lower educational attainment had an increased risk of both excessive (OR: 1.182) and inadequate (OR: 1.284) GWG as per the IOM guidelines, controlling for intervention, maternal age, parity, ethnicity, with random effects (Table 4b). The ICC calculations for model 2 of Table 4 (b) were low; excessive weight gain: ICC_{study} 8.1% and $ICC_{intervention}$ 0.4%; inadequate weight gain: ICC_{study} 5.0% and $ICC_{intervention}$ 0.7%.

The intervention-by-education interaction effects were not statistically significant, and for ease of interpretation, these were removed, thus we present results for models stratified by education.

Among those of lower educational attainment, a significant reduction in weight gain per week (kg) was observed for both diet-alone interventions (B = -0.055) and mixed interventions (B =

For peer review only - http://bmjopen.bmj.com/site/about/guidelines.xhtml

-0.044), compared to those in the control arms, Table 5 (a). No significant effect was observed for exercise-alone interventions. Women with tertiary education enrolled to mixed interventions gained significantly less GWG per week (kg) (B = -0.053), than those in the control arms. Neither diet-alone nor exercise-alone interventions had significant effects on weight gain per week (kg) for women with tertiary education. The adjusted models (Model 2) included BMI, maternal age, parity and ethnicity as confounders, with fixed effects.

Women with lower education were significantly less likely to exceed the IOM guidelines for weight gain per week if they were enrolled to diet-alone interventions (OR: 0.515) or mixed intervention (OR: 0.735), compared to those in the control arms, Table 5 (b). These women were also less likely to gain inadequate weight per week if they were enrolled to a diet-alone intervention (OR: 0.504). Women with tertiary education enrolled to diet-alone interventions were less likely to exceed IOM guidelines (OR: 0.609), however, those enrolled to mixed interventions were more likely to gain inadequate weight gain per week (OR: 1.411), compared to those in the control arms. No effect was observed for exercise-alone interventions. The adjusted models (Model 2) included maternal age, parity and ethnicity as confounders, with fixed effects.

For peer review only

For peer review only - http://bmjopen.bmj.com/site/about/guidelines.xhtml

Discussion

Summary of evidence

This IPD meta-analysis of the i-WIP consortium of lifestyle interventions in pregnancy found that low maternal education is associated with increased risk of both excessive and inadequate GWG per week, although crude differences were not observed in the percentage of women who gained inadequately, adequately and excessively between women of high and low educational attainment. Diet-alone interventions reduced the risk of excessive GWG for all women, and reduced the risk of inadequate GWG for those with lower educational attainment. Mixed interventions were protective against excessive GWG for women with low education, but increased the risk of inadequate GWG among women with higher educational attainment. Exercise-alone interventions were not associated with GWG, regardless of maternal education status.

Compared to the literature

Maternal educational attainment less than or equal to secondary level was associated with an 18% increased risk of excessive GWG and a 28% increased risk of inadequate GWG per week, as per the IOM 2009 guidelines (16), compared to those with at least some tertiary education. A systematic review of observational studies also found that women with low maternal educational attainment were less likely to gain appropriate weight, as per the 2009 IOM recommendations (10). However, the review only provided a narrative of the evidence to date, not a meta-analysis, thus was restricted from drawing firm conclusions. Nonetheless, both the systematic review and our findings highlight the need for additional support for pregnant women of low educational attainment. Although the increased risk of inadequate and excessive GWG among women with lower education was statistically significant, this is not synonymous with clinical significance. Between women of low and high educational attainment, the actual percentage differences in those who gained inadequately (19.0% vs. 14.5%), adequately (21.0% vs. 25.1%) and excessively (60.0% vs. 60.4%) was relatively small.

For peer review only - http://bmjopen.bmj.com/site/about/guidelines.xhtml

The impact of interventions in pregnancy on weight gain has been previously examined (19,21,23,47), however, analyses were not stratified by socioeconomic status within these publications, thus our study provides new insights in this field. A 2015 Cochrane review compared the effect of interventions in pregnancy (diet or exercise, or both) on GWG. Unlike our findings, in which only diet and mixed interventions reduced excessive weight gain, all categories of interventions significantly reduced excessive weight gain compared to control; diet (RR: 0.77), diet and exercise (RR: 0.86), and diet counselling/other (RR: 0.46) (47). However, the review also highlighted an increased risk of low weight gain with all types of interventions; diet (RR: 1.24), diet and exercise (RR: 1.25), and diet counselling/other (RR: 1.21) (47). A systematic review by Thangaratinam *et al.* found that dietary interventions reduced total GWG by 3.84 kg, while mixed and physical activity interventions reduced total GWG to lesser extents (–1.06 kg and –0.72 kg, respectively) (23). Both mixed and physical activity interventions reduced risk of excessive weight gain, but insufficient data were available to assess the impact of dietary interventions on excessive weight gain (23). The i-WIP primary publication in 2017 found that diet and physical activity based interventions during pregnancy reduced GWG (−0.72 kg and −0.73 kg, respectively), but did not find differences in GWG across the subgroups examined (educational attainment was not assessed) (21).

In summary, based on our findings and previous studies, it seems that among women of high and low educational status, dietary interventions are most effective in reducing GWG. Mixed interventions could also be considered for those with lower education. Our study is novel in that it is the first IPD meta-analysis, using a one-step approach for statistical analyses, in which data from individual pregnant women were used to estimate the effect of varying types of lifestyle interventions on GWG of women from different educational backgrounds.

Clinical relevance of our findings

In practice, dietary interventions have the potential be used as a baseline intervention, offered to all pregnant women, to encourage appropriate weight gain. Women with secondary education or less could be offered mixed interventions (additional behaviour change counselling or physical activity

For peer review only - http://bmjopen.bmj.com/site/about/guidelines.xhtml

advice), to complement the dietary intervention. Exercise alone interventions were not effective in managing weight gain in this analysis. The reduction in GWG associated with dietary and mixed interventions among women of low educational attainment can be considered clinically significant, given that if a woman was to follow the prescribed intervention for the duration of trimester two and three (27 weeks) she would gain 1.49 and 1.19 kg less, respectively, than a woman following usual care.

The success of dietary interventions over other lifestyle interventions in reducing GWG may be attributed to many factors. The importance of a woman's diet in pregnancy has been documented for centuries (48), thus, dietary changes are perhaps more acceptable than changes in physical activity behaviours. Pregnancy itself is an incentive for positive behaviour changes in dietary choices, but less so for physical activity (17). Misconceptions regarding exercise during pregnancy remain, with many perceiving it to pose a safety risk for their baby (49), and believing pregnancy is a time for rest (50). In addition, women may experience feelings of tiredness, nausea and physical discomfort, as well as time commitments that prevent them from exercising (49–51).

Mixed approach interventions increased the risk of inadequate weight gain among highly educated women. A potential hypothesis is that these women may already be aware of healthy lifestyle strategies, have a higher locus of control (52) and future salience (53), and therefore may be more willingly to take actions for reducing weight gain. Consequently, providing multiple approaches to reduce GWG could have a paradoxical effect. However, since 60.4% of highly educated women in this meta-analysis exceeded weight gain guidelines, the focus of healthcare professionals should be on meeting adequate GWG, rather than exerting excess caution in avoiding mixed approach interventions. It is reasonable to suggest that the dietary approach would be sufficient among those with tertiary education in reducing excessive GWG.

Strengths and limitations

The pooling of individual patient data allows for the estimation of the differential effects of education and lifestyle interventions on weight gain across the included cohorts with greater power than individual RCTs (20). IPD meta-analysis does not rely on aggregate data extracted from

For peer review only - http://bmjopen.bmj.com/site/about/guidelines.xhtml

RCTs, and allows for models to be adjusted for confounders (22). Despite the congruence between our results and those in the wider literature, it must be highlighted that reporting of negative findings in lifestyle interventions is poor, thus the evidence base to which we compare our findings may not reflect the true nature of lifestyle interventions in pregnancy. The data were however strengthened by the relatively low variability between studies. In addition, we analysed and reported findings using GWG/week, which enabled the comparison of studies that used different gestational ages for measuring GWG.

We acknowledge that a limitation of our work is the lack of pre-pregnancy weight measurements available, a methodological issue that affects much GWG research (54). We classified BMI using self-reported pre-pregnancy weight, which may be subject to underreporting and hence under-classification of BMI (54). However, using the first trimester weight to determine pre-pregnancy BMI would also have been problematic due to weight gain in trimester 1 and potential for over-classification of BMI (54). Although it has been suggested that pre-pregnancy weight can be somewhat accurately estimated using the first trimester weight, by 12 weeks' gestation weight gain can result in up to 9% of women being misclassified for BMI (55). This was not a viable option since some the first weight measurements were taken as late as 18 weeks. Early-pregnancy weight was estimated using self-reported pre-pregnancy weight for a small number of participants (n=295, 5.7%).

While limitations of the IOM guidelines have been expressed (56), they are the most widely used recommendations, and are comparable to Intergrowth 21st Project weight gain centiles for women with a healthy BMI (57) and new weight gain charts for women with a healthy BMI proposed by the MOCO collaboration (58). We also used a novel method of extrapolating the IOM expected weight gain per week in trimester 1, and this enabled calculation of trimester 2 and 3 weight gain per week. The maternal characteristics of women who were included in the IPD meta-analysis were different to those excluded; a finding that is likely related to the eligibility criteria of this meta-analysis which excluded all data from some studies that did not collect data pertaining to key characteristics required.

For peer review only - http://bmjopen.bmj.com/site/about/guidelines.xhtml

Lastly, it must be stated that although the results were statistically significant, the effect sizes were small, thus the relevance of our findings in the clinical setting must be considered.

Conclusions

In summary, pregnant women with lower educational attainment are at an increased risk of both excessive and inadequate weight gain highlighting the need for intervention among this vulnerable population of women. Given that dietary interventions do not widen health inequalities, are successful in promoting appropriate weight gain, and are acceptable; healthcare professionals should consider implementing nutrition based interventions as part of baseline maternity care packages for all pregnant women.

For peer review only

For peer review only - http://bmjopen.bmj.com/site/about/guidelines.xhtml

Tables

Table 1. Participant Exclusion Criteria for Individual Participant Data

Reason for Exclusion	Excluded N	Remaining N
Full dataset		12240
Data missing: Baseline gestational age	2611	9629
Data missing: Final follow-up gestational age	2309	7320
Data missing: Maternal educational attainment	1878	5442
Multiple pregnancy	17	5425
Less than 18 years of age	75	5350
Data missing: Pre- and early-pregnancy weight	1	5349
Data missing: Final follow-up weight	137	5212
Data missing: Height (for pre-pregnancy BMI)	29	5183

For peer review only

For peer review only - http://bmjopen.bmj.com/site/about/guidelines.xhtml

Table 2. Participant characteristics in the total group, and split by education category

	Total N		Lower Education N		Higher Education N		*P**
**Early pregnancy weight (kg)** [a]	5183	78.60 (18.56)	1910	82.51 (20.26)	3273	76.31 (17.08)	<0.001
**Pre-pregnancy weight (kg)** [a]	5183	76.06 (18.74)	1712	79.61 (20.37)	3176	73.17 (16.96)	<0.001
**Height (cm)** [a]	5183	166.07 (7.03)	1910	164.76 (7.17)	3273	166.84 (6.84)	<0.001
**Pre-pregnancy BMI** (kg/m^2) [a]	5183	27.57 (6.55)	1910	29.45 (7.08)	3273	26.47 (5.95)	<0.001
Underweight [b]	5183	77 (1.5)	1910	32 (1.6))	3273	46 (1.4)	<0.001
Normal-weight [b]		2274 (43.9)		603 (31.5)		1672 (51.1)	
Overweight [b]		1073 (20.7)		393 (20.5)		681 (20.8)	
Obese [b]		1763 (34.0)		886 (46.3)		878 (26.8)	
**Gestational age** (weeks)							
**Baseline** [c]	5183	14.5 (4.0 - 32.0)	1910	13.9 (4.0 - 32.0)	3273	14.8 (4.0 - 32.0)	<0.001
**Final follow-up** [c]	5183	36.6 (24.0 - 44.0)	1910	36.6 (27.0 - 44.0)	3273	36.5 (24 - 43.0)	0.196
**Delivery** [c]	5172	39.7 (26.0 - 44.0)	1907	39.50 (27.0 - 44.0)	3265	39.75 (26.0 - 43.0)	<0.001
**Infant birth weight** (g) [a]	5164	3531.73 (547.90)	1908	3476.55 (559.43)	3256	3564.06 (538.49)	<0.001
**Maternal age** (years) [a]	5183	29.90 (4.97)	1910	28.50 (5.51)	3273	30.72 (4.43)	<0.001
**Parity** [a]	5135	0.73 (1.05)	1884	0.93 (1.20)	3251	0.62 (0.93)	<0.001
**Ethnicity**							
Caucasian [b]	3719	3291 (63.5)	1203	954 (49.9)	2516	2338 (71.4)	<0.001
Asian [b]		84 (1.6)		25 (1.3)		60 (1.8)	
Black [b]		123 (2.4)		71 (3.7)		53 (1.6)	
Central/South American [b]		88 (1.7)		76 (3.9)		13 (.4)	
Middle East (including Iran & Turkey) [b]		63 (1.2)		45 (2.3)		19 (.5)	
Other [b]		76 (1.4)		38 (1.9)		39 (1.2)	
Missing [b]		1465 (28.2)		708 (37.0)		758 (23.1)	

Data are presented as [a] mean (SD) [b] n (%) and [c] mean (range of minimum and maximum).
**P*-value: ANOVA and chi-square
BMI: body mass index. Underweight: <18.5 kg/m^2; Normal:18.5 - 24.9 kg/m^2; Overweight: 25.0 - 29.9 kg/m^2; Obese: ≥30 kg/m^2
Lower education: secondary education or less. Higher education: at least some tertiary education

For peer review only

For peer review only - http://bmjopen.bmj.com/site/about/guidelines.xhtml

Table 3. Gestational Weight Gain per Week (kg/week), and per IOM Guidelines, BMI Category and Education

			GWG/week (kg)	Trimester 2 and 3 GWG/week (kg), and per IOM Guidelines			
	**BMI Category**	**N**	**Total GWG/week Mean (SD)**	**T2 & 3 GWG/week Mean (SD)**	**Inadequate, n (%)**	**Adequate, n (%)**	**Excess, n (%)**
	**Total**	5183	0.45 (0.22)	0.46 (0.22)	836 (16.1%)	1224 (23.6%)	3123 (60.3%)
	**Underweight**	76	0.50 (0.16)	0.51 (0.16)	23 (30.3%)	34 (44.7%)	19 (25.0%)
**All Participants**	**Normal-weight**	2273	0.51 (0.28)	0.51 (0.18)	347 (15.3%)	778 (34.2%)	1148 (50.5%)
	**Overweight**	1072	0.48 (0.22)	0.49 (0.22)	113 (10.5%)	128 (11.9%)	831 (77.5%)
	**Obese**	1762	0.35 (0.23)	0.36 (0.24)	353 (20.0%)	284 (16.1%)	1125 (63.8%)
**Lower Education**	**Total**	1910	0.42 (0.24)	0.43 (0.25)	363 (19.0%)	401 (21.0%)	1146 (60.0%)
	**Underweight**	31	0.55 (0.20)	0.56 (0.20)	7 (22.6%)	11 (35.5%)	13 (41.9%)
	**Normal-weight**	602	0.51 (0.20)	0.52 (0.21)	108 (17.9%)	184 (30.6%)	310 (51.5%)
	**Overweight**	392	0.46 (0.24)	0.47 (0.24)	55 (14.0%)	59 (15.1%)	278 (70.9%)
	**Obese**	885	0.34 (0.24)	0.35 (0.25)	193 (21.8%)	147 (16.6%)	545 (61.6%)
**Higher Education**	**Total**	3273	0.46 (0.20)	0.47 (0.21)	473 (14.5%)	823 (25.1%)	1977 (60.4%)
	**Underweight**	45	0.46 (0.10)	0.47 (0.11)	16 (35.6%)	23 (51.1%)	6 (13.3%)
	**Normal-weight**	1671	0.50 (0.17)	0.51 (0.17)	239 (14.3%)	594 (35.5%)	838 (50.1%)
	**Overweight**	680	0.49 (0.20)	0.50 (0.20)	58 (8.5%)	69 (10.1%)	553 (81.3%)
	**Obese**	877	0.35 (0.23)	0.37 (0.24)	160 (18.2%)	137 (15.6%)	580 (66.1%)

GWG: gestational weight gain
BMI: body mass index. Underweight: <18.5 kg/m^2; Normal:18.5 - 24.9 kg/m^2; Overweight: 25.0 - 29.9 kg/m^2; Obese: ≥30 kg/m^2
Lower education: secondary education or less. Higher education: at least some tertiary education

For peer review only

Table 4. Generalised Linear Mixed-Models to show relationship between maternal educational attainment and (a) gestational weight gain per week (kg), and (b) gestational weight gain per week as per the IOM guidelines

		(a) Gestational Weight Gain per Week (kg/week)			(b) Gestational Weight Gain per Week as per the IOM Guidelines					
					Excessive Weight Gain			Inadequate Weight Gain		
	Education	*B*	95% CI	*P**	OR	95% CI	*P**	OR	95% CI	*P**
**MODEL 1 – (n = 5183) †**	Lower	0.014	(0.001 - 0.027)	0.032	1.224	(1.052 - 1.425)	0.009	1.259	(1.033 - 1.535)	0.022
	Higher	Ref			Ref			Ref		
**MODEL 2 – (n = 5135) ‡**	Lower	0.005	(-0.008 - 0.018)	0.465	1.182	(1.008 - 1.385)	0.039	1.284	(1.045 - 1.577)	0.017
	Higher	Ref			Ref			Ref		

**P*-value: Wald test.
† Adjusted for intervention with random intercept and intervention slope. Weight gain per week (kg/week) additionally adjusted for BMI (kg/m^2)
‡ Adjusted for intervention, maternal age, parity, ethnicity, with random intercept and intervention slope. Weight gain per week (kg/week) additionally adjusted for BMI (kg/m^2)
Lower education: secondary education or less. Higher education: at least some tertiary education

For peer review only - http://bmjopen.bmj.com/site/about/guidelines.xhtml

Table 5. Generalised Linear Mixed-Models to show relationship between maternal educational attainment, type of intervention and (a) gestational weight gain per week (kg), and (b) gestational weight gain per week as per the IOM guidelines

			(a) Gestational Weight Gain per Week (kg/week)			(b) Gestational Weight Gain per Week as per the IOM Guidelines					
						Excessive Weight Gain			Inadequate Weight Gain		
	Education	Intervention Type	*B*	95% CI	*P**	OR	95% CI	*P**	OR	95% CI	*P**
MODEL 1 †	Lower (n = 1910)	Mixed	-0.044	(-0.069, -0.019)	<0.001	0.745	(0.572, 0.971)	0.029	1.039	(0.745, 1.449)	0.821
		Diet	-0.053	(-0.097, -0.009)	0.018	0.524	(0.351, 0.782)	0.002	0.496	(0.285, 0.864)	0.013
		Exercise	-0.020	(-0.069, 0.029)	0.422	0.704	(0.446, 1.110)	0.131	0.634	(0.333, 1.208)	0.166
		Control	Ref			Ref			Ref		
	Higher (n = 3273)	Mixed	-0.056	(-0.071, -0.040)	<0.001	0.930	(0.753, 1.150)	0.504	1.407	(1.057, 1.874)	0.019
		Diet	-0.015	(-0.038, 0.009)	0.213	0.619	(0.446, 0.858)	0.004	0.943	(0.565, 1.574)	0.822
		Exercise	-0.014	(-0.036, 0.008)	0.206	1.027	(0.753, 1.402)	0.865	1.209	(0.770, 1.899)	0.410
		Control	Ref			Ref			Ref		
MODEL 2 ‡	Lower (n = 1884)	Mixed	-0.044	(-0.069, -0.020)	<0.001	0.735	(0.561, 0.963)	0.026	1.041	(0.744, 1.458)	0.813
		Diet	-0.055	(-0.098, -0.012)	0.012	0.515	(0.339, 0.785)	0.002	0.504	(0.288, 0.884)	0.017
		Exercise	-0.018	(-0.068, 0.032)	0.472	0.660	(0.405, 1.075)	0.095	0.612	(0.314, 1.193)	0.149
		Control	Ref			Ref			Ref		
	Higher (n = 3251)	Mixed	-0.053	(-0.069, -0.037)	<0.001	0.947	(0.765, 1.174)	0.620	1.411	(1.056, 1.884)	0.020
		Diet	-0.013	(-0.037, 0.010)	0.271	0.609	(0.437, 0.849)	0.003	0.962	(0.572, 1.616)	0.882
		Exercise	-0.018	(-0.040, 0.004)	0.111	1.030	(0.751, 1.413)	0.855	1.205	(0.761, 1.909)	0.427
		Control	Ref			Ref			Ref		

**P*-value: Wald test
† With fixed effects. Weight gain per week (kg/week) adjusted for BMI (kg/m^2)
‡ With fixed effects. Adjusted for maternal age, parity and ethnicity. Weight gain per week (kg/week) adjusted for BMI (kg/m^2)
Lower education: secondary education or less. Higher education: at least some tertiary education

For peer review only - http://bmjopen.bmj.com/site/about/guidelines.xhtml

Author contributions

E.C. O'Brien	Wrote a proposal to the i-WIP data access committee. Set the objectives, study design and data analysis plan. Analysis and interpretation of data; drafting of manuscript; critical revision; final approval of version to be published; agreeable to be accountable for all aspects of the work.
R. Segurado	Analysis and interpretation of data; drafting of manuscript; critical revision; final approval of version to be published; agreeable to be accountable for all aspects of the work.
A.A. Geraghty	Analysis and interpretation of data; drafting of manuscript; critical revision; final approval of version to be published; agreeable to be accountable for all aspects of the work.
G. Alberdi	Analysis and interpretation of data; drafting of manuscript; critical revision; final approval of version to be published; agreeable to be accountable for all aspects of the work.
E. Rogozińska	i-WIP IPD database manager; study conception and design; critical revision; final approval of version to be published; agreeable to be accountable for all aspects of the work.
A. Astrup	Study conception and design; critical revision; final approval of version to be published; agreeable to be accountable for all aspects of the work.
R. Barakat Carballo	Study conception and design; critical revision; final approval of version to be published; agreeable to be accountable for all aspects of the work.
A. Bogaerts	Study conception and design; critical revision; final approval of version to be published; agreeable to be accountable for all aspects of the work.
J.G. Cecatti	Study conception and design; critical revision; final approval of version to be published; agreeable to be accountable for all aspects of the work.
A. Coomarasamy	Study conception and design; critical revision; final approval of version to be published; agreeable to be accountable for all aspects of the work.
C.J. de Groot	Study conception and design; critical revision; final approval of version to be published; agreeable to be accountable for all aspects of the work.
R. Devlieger	Study conception and design; critical revision; final approval of version to be published; agreeable to be accountable for all aspects of the work.
J.Dodd	Study conception and design; critical revision; final approval of version to be published; agreeable to be accountable for all aspects of the work.
N.El Beltagy	Study conception and design; critical revision; final approval of version to be published; agreeable to be accountable for all aspects of the work.
F. Facchinetti	Study conception and design; critical revision; final approval of version to be published; agreeable to be accountable for all aspects of the work.
N.R. Geiker	Study conception and design; critical revision; final approval of

For peer review only

	version to be published; agreeable to be accountable for all aspects of the work.
K.J Guelfi	Study conception and design; critical revision; final approval of version to be published; agreeable to be accountable for all aspects of the work.
L. Haakstad	Study conception and design; critical revision; final approval of version to be published; agreeable to be accountable for all aspects of the work.
C.Harrison	Study conception and design; critical revision; final approval of version to be published; agreeable to be accountable for all aspects of the work.
H. Hauner	Study conception and design; critical revision; final approval of version to be published; agreeable to be accountable for all aspects of the work.
D.M. Jensen	Study conception and design; critical revision; final approval of version to be published; agreeable to be accountable for all aspects of the work.
K.S. Khan	Study conception and design; critical revision; final approval of version to be published; agreeable to be accountable for all aspects of the work.
T. I. Kinnunen	Study conception and design; critical revision; final approval of version to be published; agreeable to be accountable for all aspects of the work.
R. Luoto	Study conception and design; critical revision; final approval of version to be published; agreeable to be accountable for all aspects of the work.
B. W. Mol	Study conception and design; critical revision; final approval of version to be published; agreeable to be accountable for all aspects of the work.
S. Mørkved	Study conception and design; critical revision; final approval of version to be published; agreeable to be accountable for all aspects of the work.
N. Motahari	Study conception and design; critical revision; final approval of version to be published; agreeable to be accountable for all aspects of the work.
J. Owens	Study conception and design; critical revision; final approval of version to be published; agreeable to be accountable for all aspects of the work.
M. Perales	Study conception and design; critical revision; final approval of version to be published; agreeable to be accountable for all aspects of the work.
E. Petrella	Study conception and design; critical revision; final approval of version to be published; agreeable to be accountable for all aspects of the work.
S. Phelan	Study conception and design; critical revision; final approval of version to be published; agreeable to be accountable for all aspects of the work.
L. Poston	Study conception and design; critical revision; final approval of version to be published; agreeable to be accountable for all aspects of the work.
K. Rauh	Study conception and design; critical revision; final approval of

For peer review only

	version to be published; agreeable to be accountable for all aspects of the work.
G. Rayanagoudar	Study conception and design; critical revision; final approval of version to be published; agreeable to be accountable for all aspects of the work.
K.M. Renault	Study conception and design; critical revision; final approval of version to be published; agreeable to be accountable for all aspects of the work.
A.E. Ruifrok	Study conception and design; critical revision; final approval of version to be published; agreeable to be accountable for all aspects of the work.
L.R. Sagedal	Study conception and design; critical revision; final approval of version to be published; agreeable to be accountable for all aspects of the work.
K.Å Salvesen	Study conception and design; critical revision; final approval of version to be published; agreeable to be accountable for all aspects of the work.
T.T. Scudeller	Study conception and design; critical revision; final approval of version to be published; agreeable to be accountable for all aspects of the work.
G.X. Shen	Study conception and design; critical revision; final approval of version to be published; agreeable to be accountable for all aspects of the work.
A. Shub	Study conception and design; critical revision; final approval of version to be published; agreeable to be accountable for all aspects of the work.
S.N. Stafne	Study conception and design; critical revision; final approval of version to be published; agreeable to be accountable for all aspects of the work.
F. Surita	Study conception and design; critical revision; final approval of version to be published; agreeable to be accountable for all aspects of the work.
S. Thangaratinam	Study conception and design; critical revision; final approval of version to be published; agreeable to be accountable for all aspects of the work.
S. Tonstad	Study conception and design; critical revision; final approval of version to be published; agreeable to be accountable for all aspects of the work.
M.N. van Poppel	Study conception and design; critical revision; final approval of version to be published; agreeable to be accountable for all aspects of the work.
C. Vinter	Study conception and design; critical revision; final approval of version to be published; agreeable to be accountable for all aspects of the work.
I. Vistad	Study conception and design; critical revision; final approval of version to be published; agreeable to be accountable for all aspects of the work.
S. Yeo	Study conception and design; critical revision; final approval of version to be published; agreeable to be accountable for all aspects of the work.
i-WIP (International	NA – group name

For peer review only - http://bmjopen.bmj.com/site/about/guidelines.xhtml

Weight Management in Pregnancy) Collaborative Group	
F.M. McAuliffe	Wrote a proposal to the i-WIP data access committee. Set the objectives, study design and data analysis plan. Analysis and interpretation of data; drafting of manuscript; critical revision; final approval of version to be published; agreeable to be accountable for all aspects of the work.

Competing interests statement

The authors did not report any potential conflicts of interest

Funding Statement

The data come from a National Institute for Health Research (NIHR) funded project (HTA - 12/01/50) and the Queen Mary University of London is its legal Sponsor. The database was initially funded by the NIHR and is currently funded by World Health Organization (WHO). This work was also supported by the Health Research Board, Health Research Centre for Health and Diet Research, Ireland.

Data sharing statement

The Queen Mary University of London is the legal custodian of the i-WIP dataset. The access to the dataset is regulated by prespecified terms and conditions (available on request) and overseen by Data Access Committee. For further information visit: https://iwipgroup.wixsite.com/collaboration/related-projects

Acknowledgements

We are also grateful to the i-WIP Network for the individual participant data shared and to Helena Teede, Janette Khoury and Márcia Vitolo for the contribution of data.

For peer review only

References

1. United Nations. Transforming Our World: The 2030 Agenda for Sustainable Development. New York; 2015.
2. Agyemang C, Vrijkotte TGM, Droomers M, van der Wal MF, Bonsel GJ, Stronks K. The effect of neighbourhood income and deprivation on pregnancy outcomes in Amsterdam, The Netherlands. J Epidemiol Community Health. 2009;63(9):755-760.
3. Metcalfe A, Lail P, Ghali WA, Sauve RS. The association between neighbourhoods and adverse birth outcomes: a systematic review and meta-analysis of multi-level studies. Paediatr Perinat Epidemiol. 2011;25(3):236-245.
4. Morgen CS, Bjørk C, Andersen PK, Mortensen LH, Nybo Andersen A-M, Bjork C, et al. Socioeconomic position and the risk of preterm birth-a study within the Danish National Birth Cohort. Int J Epidemiol. 2008;37:1109-1120.
5. Silva LM, Jansen PW, Steegers E a P, Jaddoe VW V, Arends LR, Tiemeier H, et al. Mother's educational level and fetal growth: The genesis of health inequalities. Int J Epidemiol. 2010;39:1250-1261.
6. Blumenshine P, Egerter S, Barclay CJ, Cubbin C, Braveman PA. Socioeconomic Disparities in Adverse Birth Outcomes. Am J Prev Med. 2010;39(3):263-272.
7. Tofani AA, Lamarca G de A, Sheiham A, Vettore MV. The different effects of neighbourhood and individual social capital on health-compromising behaviours in women during pregnancy: a multi-level analysis. BMC Public Health. 2015;15:890.
8. Freisling H, Elmadfa I, Gall I. The effect of socioeconomic status on dietary intake, physical activity and Body Mass Index in Austrian pregnant women. J Hum Nutr Diet. 2006;19:437-445.
9. O'Brien EC, Alberdi G, Geraghty AA, McAuliffe FM. Lower education predicts poor response to dietary intervention in pregnancy, regardless of neighbourhood

For peer review only

affluence: secondary analysis from the ROLO randomised control trial. Public Health Nutr. 2017;20(16):2959–69.

10. O'Brien EC, Alberdi G, McAuliffe FM. The influence of socioeconomic status on gestational weight gain: a systematic review. J Public Heal. 2018;40(1):41-55

11. Truong YN, Yee LM, Caughey AB, Cheng YW. Weight gain in pregnancy: does the Institute of Medicine have it right? Am J Obstet Gynecol. 2015;212(3):362.e1-e8.

12. Goldstein RF, Abell SK, Ranasinha S, Misso M, Boyle J a., Black MH, et al. Association of gestational weight gain with maternal and infant outcomes: A systematic review and meta-analysis. JAMA. 2017;317(21):2207-2225.

13. O'Brien EC, Geraghty AA, O'Sullivan EJ, Riordan J, Horan MK, Larkin E, et al. Five-year follow up of a low glycaemic index dietary randomised controlled trial in pregnancy—no long-term maternal effects of a dietary intervention. BJOG.2019;126(4):514–24.

14. Amorim AR, Rössner S, Neovius M, Lourenço PM, Linné Y. Does excess pregnancy weight gain constitute a major risk for increasing long-term BMI? Obesity (Silver Spring). 2007;15(5):1278-1286.

15. NCD Risk Factor Collaboration (NCD-RisC). Trends in adult body-mass index in 200 countries from 1975 to 2014: a pooled analysis of 1698 population-based measurement studies with 19.2 million participants. Lancet. 2016;387(10026):1377-1396.

16. Institute of Medicine. National Research Council (US) Committee to Reexamine IOM Pregnancy Weight Guidelines. Weight Gain During Pregnancy. Washington (DC): National Academies Press (US); 2009.

17. O'Brien OA, Lindsay KL, McCarthy M, McGloin AF, Kennelly M, Scully HA, et al. Influences on the food choices and physical activity behaviours of overweight and obese pregnant women: A qualitative study. Midwifery. 2017;(47):28-35.

For peer review only

18. Jackson RA, Stotland NE, Caughey AB, Gerbert B. Improving diet and exercise in pregnancy with Video Doctor counseling: A randomized trial. Patient Educ Couns. 2011;83(2):203-209.

19. Thangaratinam S, Rogozinska E, Jolly K, Glinkowski S, Duda W, Borowiack E, et al. Interventions to reduce or prevent obesity in pregnant women: a systematic review. Health Technol Assess. 2012;16(31).

20. Ruifrok AE, Rogozinska E, van Poppel MNM, Rayanagoudar G, Kerry S, de Groot CJM, et al. Study protocol: differential effects of diet and physical activity based interventions in pregnancy on maternal and fetal outcomes--individual patient data (IPD) meta-analysis and health economic evaluation. Syst Rev. 2014;3:131.

21. The International Weight Management in Pregnancy (i-WIP) Collaborative Group. Effect of diet and physical activity based interventions in pregnancy on gestational weight gain and pregnancy outcomes: meta-analysis of individual participant data from randomised trials. BMJ. 2017;358:j3119

22. Stewart LA, Clarke M, Rovers M, Riley RD, Simmonds M, Stewart G, et al. Preferred Reporting Items for a Systematic Review and Meta-analysis of Individual Participant Data. JAMA. 2015;313(16):1657.

23. Thangaratinam S, Rogozinska E, Jolly K, Glinkowski S, Roseboom T, Tomlinson JW, et al. Effects of interventions in pregnancy on maternal weight and obstetric outcomes: meta-analysis of randomised evidence. BMJ. 2012;344:e2088.

24. Ruifrok AE, Rogozinska E, van Poppel MNM, Rayanagoudar G, Kerry S, de Groot CJM, et al. Erratum to: Study protocol: differential effects of diet and physical activity based interventions in pregnancy on maternal and fetal outcomes: individual patient data (IPD) meta-analysis and health economic evaluation. Syst Rev. 2015;4:101.

25. Shavers VL. Measurement of socioeconomic status in health disparities research. J Natl Med Assoc. 2007;99(9):1013-1023.

For peer review only

26. Althuizen E, van der Wijden C, van Mechelen W, Seidell J, van Poppel M. The effect of a counselling intervention on weight changes during and after pregnancy: a randomised trial. BJOG. 2013;120:92-99.

27. Baciuk EP, Pereira RI, Cecatti JG, Braga AF, Cavalcante SR. Water aerobics in pregnancy: Cardiovascular response, labor and neonatal outcomes. Reprod Health. 2008;5:10.

28. Bogaerts AFL, Devlieger R, Nuyts E, Witters I, Gyselaers W, van den Bergh BRH. Effects of lifestyle intervention in obese pregnant women on gestational weight gain and mental health: a randomized controlled trial. Int J Obes. 2013;37(6):814-821.

29. Haakstad LAH, Bø K. Exercise in pregnant women and birth weight: a randomized controlled trial. BMC Pregnancy Childbirth. 2011;11:66.

30. Harrison CL, Lombard CB, Strauss BJ, Teede HJ. Optimizing healthy gestational weight gain in women at high risk of gestational diabetes: A randomized controlled trial. Obesity. 2013;21(5):904-909.

31. Jeffries K, Shub A, Walker SP, Hiscock R, Permezel M. Reducing excessive weight gain in pregnancy: a randomised controlled trial. Med J Aust. 2009;191(8):429-433.

32. Khaledan A, Mirdar S, Motahari Tabari N, Ahmad Shirvani M. Effect of an Aerobic Exercise Program on Fetal Growth in Pregnant Women. J Hayat. 2010;16(1):55-64.

33. Khoury J, Henriksen T, Christophersen B, Tonstad S. Effect of a cholesterol-lowering diet on maternal, cord, and neonatal lipids, and pregnancy outcome: A randomized clinical trial. Am J Obstet Gynecol. 2005;193(4):1292-1301.

34. Nascimento S, Surita F, Parpinelli M, Siani S, Pinto e Silva J. The effect of an antenatal physical exercise programme on maternal/perinatal outcomes and quality of life in overweight and obese pregnant women: a randomised clinical trial. BJOG. 2011;118:1455-1463.

For peer review only

35. Oostdam N, van Poppel M, Wouters M, Eekhoff E, Bekedam D, Kuchenbecker W, et al. No effect of the FitFor2 exercise programme on blood glucose, insulin sensitivity, and birthweight in pregnant women who were overweight and at risk for gestational diabetes: results of a randomised controlled trial. BJOG. 2012;119:1098-1107.

36. Petrella E, Malavolti M, Bertarini V, Pignatti L, Neri I, Battistini NC, et al. Gestational weight gain in overweight and obese women enrolled in a healthy lifestyle and eating habits program. J Matern Neonatal Med. 2014;27(13):1348-1352.

37. Phelan S, Phipps MG, Abrams B, Darroch F, Schaffner A, Wing RR. Randomized trial of a behavioral intervention to prevent excessive gestational weight gain: the Fit for Delivery Study. Am J Clin Nutr. 2011;93(4):772-779.

38. Poston L, Bell R, Croker H, Flynn AC, Godfrey KM, Goff L, et al. Effect of a behavioural intervention in obese pregnant women (the UPBEAT study): a multicentre, randomised controlled trial. Lancet Diabetes Endocrinol. 2015;3(10):767-777.

39. Rauh K, Gabriel E, Kerschbaum E, Schuster T, von Kries R, Amann-Gassner U, et al. Safety and efficacy of a lifestyle intervention for pregnant women to prevent excessive maternal weight gain: a cluster-randomized controlled trial. BMC Pregnancy Childbirth. 2013;13(1):151.

40. Renault KM, N Nørgaard K, Nilas L, Carlsen EM, Cortes D, Pryds O, et al. The Treatment of Obese Pregnant Women (TOP) study: A randomized controlled trial of the effect of physical activity intervention assessed by pedometer with or without dietary intervention in obese pregnant women. Am J Obstet Gynecol. 2014;210(2):134.e1-e134.e9.

41. Sagedal L, Øverby N, Bere E, Torstveit M, Lohne-Seiler H, Småstuen M, et al. Lifestyle intervention to limit gestational weight gain: the Norwegian Fit for Delivery randomised controlled trial. BJOG. 2016. doi: 10.1111/1471-0528.13862

For peer review only

42. Stafne SN, Salvesen KÅ, Romundstad PR, Stuge B, Mørkved S. Does regular exercise during pregnancy influence lumbopelvic pain? A randomized controlled trial. Acta Obstet Gynecol Scand. 2012;91:552-559.

43. Vinter CA, Jensen DM, Ovesen P, Beck-Nielsen H, Jørgensen JS. The LiP (Lifestyle in Pregnancy) study: a randomized controlled trial of lifestyle intervention in 360 obese pregnant women. Diabetes Care. 2011;34(12):2502-2507.

44. Vítolo MR, Bueno MSF, Gama CM. [Impact of a dietary counseling program on the gain weight speed of pregnant women attended in a primary care service]. Rev Bras Ginecol Obs. 2011;33(1):13-9.

45. Walsh JM, McGowan CA, Mahony R, Foley ME, McAuliffe FM. Low glycaemic index diet in pregnancy to prevent macrosomia (ROLO study): randomised control trial. BMJ. 2012;345:e5605.

46. Wolff S, Legarth J, Vangsgaard K, Toubro S, Astrup A. A randomized trial of the effects of dietary counseling on gestational weight gain and glucose metabolism in obese pregnant women. Int J Obes. 2008;32:495-501.

47. Muktabhant B, Lawrie TA, Lumbiganon P, Laopaiboon M. Diet or exercise, or both, for preventing excessive weight gain in pregnancy. Cochrane Database Syst Rev. 2015;6:CD007145.

48. Institute of Medicine (US) and Food and Nutritional Board. Historical Trends in Clinical Practice, Maternal Nutritional Status, and the Course and Outcome of Pregnancy. In: Nutrition During Pregnancy. Washington (DC); 1990:37-38.

49. Duncombe D, Wertheim EH, Skouteris H, Paxton SJ, Kelly L. Factors related to exercise over the course of pregnancy including women's beliefs about the safety of exercise during pregnancy. Midwifery. 2009;25(4):430-438.

50. Heery E, McConnon A, Kelleher CC, Wall PG, McAuliffe FM. Perspectives on weight gain and lifestyle practices during pregnancy among women with a history of

For peer review only

macrosomia: a qualitative study in the Republic of Ireland. BMC Pregnancy Childbirth. 2013;13:202.

51. Cioffi J, Schmied V, Dahlen H, Mills A, Thornton C, Duff M, et al. Physical Activity in Pregnancy: Women's Perceptions, Practices, and Influencing Factors. J Midwifery Womens Health. 2010;55(5):455-461

52. Weisman CS, Hillemeier MM, Chase GA, Misra DP, Chuang CH, Parrott R, et al. Women's perceived control of their birth outcomes in the Central Pennsylvania Women's Health Study: implications for the use of preconception care. Womens Health Issues. 2008;18(1):17-25.

53. Wardle J, Steptoe A. Socioeconomic differences in attitudes and beliefs about healthy lifestyles. J Epidemiol Community Health. 2003;57(6):440-443.

54. Gilmore LA, Redman LM. Weight gain in pregnancy and application of the 2009 IOM guidelines: toward a uniform approach. Obesity (Silver Spring). 2015;23(3):507-511.

55. Krukowski RA, West DS, DiCarlo M, Shankar K, Cleves MA, Saylors ME, et al. Are early first trimester weights valid proxies for preconception weight? BMC Pregnancy Childbirth. 2016;16(1):357.

56. Kapadia MZ, Park CK, Beyene J, Giglia L, Maxwell C, McDonald SD. Can we safely recommend gestational weight gain below the 2009 guidelines in obese women? A systematic review and meta-analysis. Obes Rev. 2015;16(3):189-206.

57. Cheikh Ismail L, Bishop DC, Pang R, Ohuma EO, Kac G, Abrams B, et al. Gestational weight gain standards based on women enrolled in the Fetal Growth Longitudinal Study of the INTERGROWTH-21st Project: a prospective longitudinal cohort study. BMJ. 2016;352:i555.

58. Santos S, Eekhout I, Voerman E, Gaillard R, Barros H, Charles M-A, et al. Gestational weight gain charts for different body mass index groups for women in Europe, North America, and Oceania. BMC Med. 2018;16(1):201.

For peer review only

Figure Captions

Figure 1. Gestational weight gain (kg/week), stratified by to pre-pregnancy BMI, intervention and educational attainment

For peer review only

For peer review only - http://bmjopen.bmj.com/site/about/guidelines.xhtml

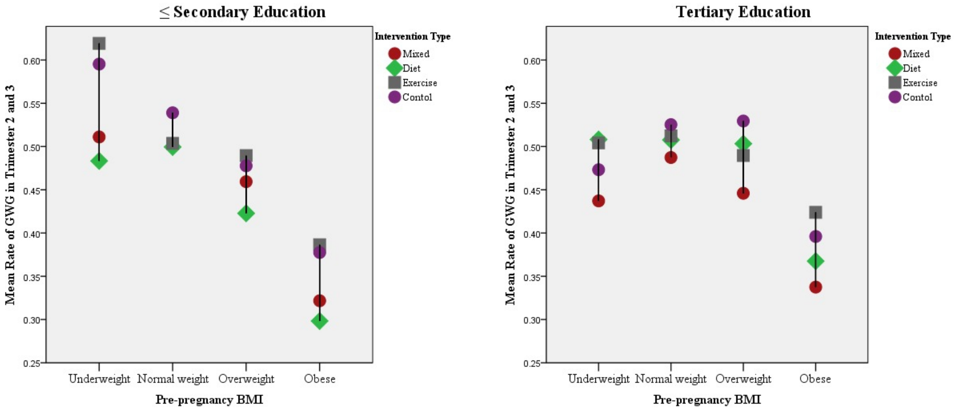

Figure 1. Gestational weight gain (kg/week), according to pre-pregnancy BMI, intervention and educational attainment

440x190mm (72 x 72 DPI)

Supplementary Files

For peer review only

Table S1. Study Characteristics of Randomised Control Trials Included in the IPD Meta-Analysis (n = 21)

Author (Year)	Period	Original inclusion/exclusion criteria	Country	Method to measure gestational weight gain (GWG)	Intervention	Intervention Detail	Outcome	Original i-WIP N<br>N (%) in this analysis
Althuizen *et al*. (2013)	February 2005 to May 2006	Inclusion criteria: primigravida, ability to read and speak Dutch, ≤14 weeks' gestation.	Netherlands	Pre-pregnancy: self-reported. Early pregnancy: measured at 15 weeks' gestation	Mixed	Counselling sessions (The New Life[style] Study) five times during pregnancy (15 minutes each) on weight, physical activity and diet.	Excessive weight gain, BMI, postpartum weight retention, birth weight, macrosomia, preterm birth, gestational diabetes	258<br>187 (72.5%)
Baciuk *et al*. (2008)	Not described	Inclusion criteria: <20 weeks' gestation, singleton pregnancy, receiving prenatal care at the institution and intended to give birth there. Exclusion criteria: engaging in regular physical activity, ≥2 caesarean sections, neurological, cardio-vascular, pulmonary, muscular-skeletal or endocrine disorders, morbid obesity, severe anaemia or vaginal bleeding during pregnancy.	Brazil	Pre-pregnancy: self-reported. Early pregnancy: (estimated from pre-pregnancy as per methods)	Exercise	Water aerobics three times per week (50 minutes each) in an indoor swimming pool.	Maternal cardiovascular capacity, heart rate, blood pressure, experience at delivery and fetal heart rate	71<br>68 (95.8%)
Bogaerts *et al*. (2013)	March 2008 and April 2011	Inclusion: BMI ≥29 kg/m^2. Exclusion criteria: >15 weeks' gestation, pre-existing type 1 diabetes, multiple pregnancy, primary need for nutritional advice and insufficient knowledge of the Dutch language	Belgium	Pre-pregnancy self-reported. Early pregnancy: (estimated from pre-pregnancy as per methods)	Mixed	Two arms. (1) Brochure group: brochure with advice on diet, physical activity and limiting excessive GWG, (2) Prenatal session group: brochure plus four prenatal motivational lifestyle intervention sessions led by a trained midwife.	GWG, Anxiety, feelings of depression	197<br>196 (99.5%)
Haakstad *et al*. (2011)	September 2007 to March 2008	Inclusion criteria: Nulliparous, low pre-pregnancy exercise, sufficient understanding of Norwegian, and ≤24 weeks' gestation. Exclusion criteria: history of miscarriages, severe heart disease, persistent bleeding after 12 weeks' gestation, multiple pregnancy, poorly controlled thyroid disease, pregnancy-induced hypertension or pre-eclampsia, diabetes or gestational diabetes, and other diseases that could interfere with participation.	Norway	Pre-pregnancy self-reported. Early pregnancy: measured at 12-24 weeks' gestation	Exercise	Aerobic dance/strength training class 2-3 times per week (one hour each) for a minimum of 12 weeks. Advised 30 minutes of moderate self-imposed physical activity on the remaining week-days (recorded in a personal training diary).	Infant birth weight, gestational age at delivery, Apgar score.	105<br>83 (79.0%)

For peer review only

Table S1. cont.

Author (Year)	Period	Original inclusion/exclusion criteria	Country	Method to measure GWG	Intervention	Intervention Detail	Outcome	Original i-WIP N / N (%) in this analysis
Harrison *et al*. (2013)	Not described	Inclusion criteria: 12-15 weeks' gestation, BMI ≥25 or ≥23 kg/m^2 if high-risk ethnicity (Polynesian, Asian, and African populations) or ≥30 kg/m^2, increased risk of GDM identified by risk prediction tool, agreement to complete an OGTT at 28 weeks' gestation. Exclusion criteria: multiple pregnancies, type 1 or 2 diabetes, a BMI ≥45 kg/m^2, pre-existing chronic medical conditions, and non-English-speaking	Australia	Pre-pregnancy: self-reported Early pregnancy: measured at 12-15 weeks' gestation.	Mixed	Behaviour change lifestyle intervention with four individual sessions based on the Social Cognitive Theory, at 14-16, 20, 24, and 28 weeks' gestation. Provided with simple healthy eating and physical activity messages. Behavioural change strategies were practised to identify short-term goals, and increase self-efficacy and self-monitoring. Goals were determined individually by the participants. Self-monitoring strategies included pedometers and the use of weight gain charts.	GWG, GDM screening	238 / 187 (78.6%)
Jeffries *et al*. (2009)	July and October 2007	Inclusion criteria: ≤14 weeks' gestation. Exclusion criteria were: age <18 years or >45 years, type 1 or type 2 diabetes mellitus, multiple pregnancy, or non-English speaking.	Australia	Pre-pregnancy self-reported. Early pregnancy: measured at 14 weeks' gestation	Mixed	Advised on optimal GWG based on IOM 1990 guidelines. Provided with a personalised weight measurement card and instructed to record their weight at 16, 20, 24, 28, 30, 32 and 34 weeks' gestation.	GWG, adherence to IOM guidelines (1990)	286 / 236 (82.5%)
Khaledan *et al*. (2010)	Not described	Inclusion criteria: Singleton, healthy pregnancy. Exclusion criteria: chronic disease, multiple pregnancy, complications, risk of preterm delivery, hypertension, severe anaemia, type 1 diabetes with poor control, morbid obesity, low maternal weight, history of completely sedentary lifestyle, heavy smoker.	Iran	Pre-pregnancy: na. Early pregnancy: Not described	Exercise	Aerobic (stepping) and flexibility exercise session three times per week (30-45 minutes each) for eight weeks.	Type of delivery, cause of caesarean section, maternal and fetal weight, birth weight and gestational age	39 / 37 (94.9%)
Khoury *et al*. (2005)	June 1999 to November 2001	Inclusion criteria: 17-20 weeks' gestation, singleton, healthy pregnancy, non-smoker (or stopped ≥5 years previously), 21-38 years, BMI 19-32 kg/m^2. Exclusion criteria: following a vegetarian or Mediterranean diet, immigrant to Norway from non-Western country, high-risk pregnancy, complications in a previous pregnancy, on-going hyperemesis gravidarum or bleeding after gestational week 12	Norway	Pre-pregnancy: na. Early pregnancy: measured at 17-20 week's gestation	Diet	Dietary advice from dietitian at inclusion, 24, 30 and 36 weeks' gestation. Cholesterol-lowering diet; promoted fish, low-fat meats and dairy products, oils, whole grains, fruits, vegetables, and legumes from gestational week 17-20 until delivery.	Maternal, cord, and neonatal plasma lipids and pregnancy-related complications, including hypertensive complications, preterm delivery, and intrauterine growth restriction, birth weight and maternal weight gain.	290 / 198 (68.3%)

For peer review only

Table S1. cont.

Author (Year)	Period	Original inclusion/exclusion criteria	Country	Method to measure GWG	Intervention	Intervention Detail	Outcome	Original i-WIP N / N (%) in this analysis
Nascimento *et al.* (2011)	August 2008 to October 2009	Inclusion criteria: overweight or obese, age >18 years, 14-24 weeks' gestation. Exclusion criteria: multiple gestations, exercising regularly, conditions that contraindicate exercise	Brazil	Pre-pregnancy self-reported. Early pregnancy: measured at 14-24 weeks' gestation	Exercise	One exercise class per week (40 minutes each), under the guidance of a trained physical therapist. Light-intensity to moderate-intensity exercises. Advised to exercise at home five times per week.	GWG, maternal arterial blood pressure and perinatal outcome, perception of quality of life.	82 / 77 (93.9%)
Oostdam *et al.* (2012)	November 2007 and April 2010	Inclusion criteria: obese or overweight and at least one of the following characteristics: history of macrosomia; history of GDM; or first-grade relative with type 2 diabetes. Exclusion criteria: >20 weeks' gestation; <18 years; inadequate knowledge of Dutch language; GDM; hypertension; alcohol abuse; drug abuse; use of any medication that affects insulin secretion or insulin sensitivity; chronic disease that could prevent the woman from implementing the study protocol.	Netherlands	Pre-pregnancy self-reported. Early pregnancy: measured at 15-20 weeks' gestation	Exercise	Aerobic and strength exercise classes (FitFor2 Study). Two days per week (60 minutes each), under supervision of physiotherapist, from 14-20 weeks' gestation to end of pregnancy.	Fasting blood glucose, insulin, HbA1c; body weight, BMI, daily physical activity, birth weight and fetal growth.	124 / 75 (60.5%)
Petrella *et al.* (2014)	April 2011 to October 2011	Inclusion criteria: Pre-pregnancy BMI ≥25 kg/m^2, >18 years and singleton pregnancy. Exclusion criteria: twin pregnancy, chronic diseases (i.e. diabetes mellitus, chronic hypertension, untreated thyroid diseases), previous GDM, smoker, previous bariatric surgery, regular physical activity, dietary supplements or herbal products known to affect body weight, other medical conditions that might affect body weight, and plans to deliver outside the centre	Italy	Pre-pregnancy self-reported. Early pregnancy: measured at 12 weeks' gestation	Mixed	Diet, physical activity and counselling about GWG. Dietary advice: low glycaemic index diet, three main meals and three snacks (1500 kcal/day). Physical activity advice: 3 days per week of moderate intensity activity (30 minutes each), monitored using pedometer. One-hour counselling session about appropriate GWG.	Excessive weight gain, gestational diabetes mellitus, gestational hypertension and preterm delivery.	61 / 58 (95.1%)

For peer review only

Table S1. cont.

Author (Year)	Period	Original inclusion/exclusion criteria	Country	Method to measure GWG	Intervention	Intervention Detail	Outcome	Original i-WIP N / N (%) in this analysis
Phelan *et al*. (2011)	2006 to 2008	Inclusion criteria: 10 - 16 week's gestation, BMI 19.8 - 40 kg/m², non-smoking, >18 years, fluency in English, access to a telephone, and a singleton pregnancy. Exclusion criteria: self-reported major health or psychiatric diseases, weight loss during pregnancy, or a history of >3 miscarriages	USA	Pre-pregnancy self-reported. Early pregnancy: measured at 10-16 weeks' gestation	Mixed	Behavioural intervention with diet and physical activity advice (Fit for Delivery Study). One face-to-face visit to discuss appropriate GWG, physical activity (30 min of walking most days of the week), and calorie goals (20 kcal/kg), with daily self-monitoring of eating, exercise, and weight. Provided with weight scales, food records, pedometers, weekly reminders, personalised graphs of weight gained with feedback, 3 supportive phone calls from the dietitian. If gained over or under weight gain guidelines received 2 additional supportive phone calls per month with meal plans and goals until weight gains returned to appropriate amounts.	GWG and the proportion who returned to pre-gravid weights by 6 months' postpartum.	401 / 395 (98.5%)
Poston *et al*. (2015)	March 2009 to June 2014	Inclusion criteria: >16 years, BMI ≥30 kg/m², singleton pregnancy, 15 - 18(+6) weeks' gestation. Exclusion criteria: unwilling or unable to give informed consent; underlying disorders, including a pre-pregnancy diagnosis of essential hypertension, diabetes, renal disease, systemic lupus erythematosus, antiphospholipid syndrome, sickle- cell disease, thalassaemia, coeliac disease, thyroid disease, and current psychosis; or currently being prescribed metformin.	UK	Pre-pregnancy: na. Early pregnancy: measured at 15-18 week's gestation	Mixed	Behavioural intervention with diet and physical activity advice (UPBEAT Study). One individual interview plus one group/individual session per week (60 minutes each) for 8 weeks. Advised to self-monitor, identify, and problem-solve of barriers to behaviour change and enlist social support. Dietary intervention aimed to promote a healthy pattern of eating and low glycaemic index diet. Exercise advice focused on incremental increases in walking from a pedometer- assessed baseline, tailored to pre-existing activities.	Gestational diabetes and large-for-gestational-age infants (≥90th customised birth weight centile)	1554 / 378 (24.3%)

For peer review only

Table S1. cont.

Author (Year)	Period	Original inclusion/exclusion criteria	Country	Method to measure GWG	Intervention	Intervention Detail	Outcome	Original i-WIP N / N (%) in this analysis
Rauh *et al*. (2013)	February 2010 and August 2011	Inclusion criteria: >18 years; singleton pregnancy; <18 weeks' gestation; BMI ≥18.5 kg/m², and sufficient German. Exclusion criteria: any condition preventing physical activity, GDM and uncontrolled chronic diseases that may affect weight development like thyroid dysfunction or psychiatric diseases	Germany	Pre-pregnancy self-reported. Early pregnancy: measured at 10-16 weeks' gestation	Mixed	Individual counselling sessions on nutrition, physical activity, and GWG monitoring (FeLIPO Study). Twice during pregnancy; 20th (60 minutes) and 30th (30 minutes) week of gestation. Provided with general healthy lifestyle information; prompted to self-monitor behaviour by recording diet, physical activity, and weight gain using weight gain charts; and to set behavioural goals based on the baseline situation (BMI, diet, physical activity) and individual preferences.	Weight gain in excess of IOM recommendations, and weight retention at 4 months' postpartum	250 / 231 (92.4%)
Renault *et al*. (2014)	March 2009 to March 2012	Inclusion criteria: >18 years, singleton pregnancy, normal scan in weeks 11-14, <16 weeks' gestation, sufficient Danish. Exclusion criteria: multiple pregnancy, pre-gestational diabetes, or other serious diseases limiting level of physical activity, previous bariatric surgery, or alcohol or drug abuse.	Denmark	Pre-pregnancy self-reported. Early pregnancy: measured at 11-16 weeks' gestation	Mixed	Physical activity intervention with or without dietary intervention (TOP Study). Physical activity advice: daily step count of 11,000, monitored with pedometer. Diet: hypocaloric (1200-1675 kcal), low-fat, Mediterranean-style diet and contact with an experienced dietitian every 2 weeks, alternating between outpatient visits and phone contacts (measurement of weight, encouragement, and correcting advice on the diet).	GWG, pregnancy complications and delivery complications.	425 / 370 (87.1%)
Sagedal *et al*. (2017)	September 2009 and February 2013	Inclusion criteria: Nulliparous, singleton pregnancy, ≤20 weeks' gestation, pre-pregnancy BMI of ≥19 kg/m^2, sufficient in Norwegian or English, and provided signed, informed consent. Exclusion criteria: pre-existing diabetes, disabilities precluding participation in a physical fitness programme, continued substance abuse, or planned relocation outside of the study area before delivery.	Norway	Pre-pregnancy self-reported. Early pregnancy: measured at ≤20 weeks' gestation	Mixed	Lifestyle intervention with diet and physical activity components (Norwegian Fit for Delivery Study). Dietary advice provided by telephone, with one initial consultation and follow-up 4–6 weeks later (20 minutes). Dietary advice aimed to increase awareness of food choices. GWG based on pre-pregnancy BMI provided. Physical activity: access to exercise classes at a local gym twice/week (60 minutes). Provided with booklets, access to study website, invitation to one cooking class and to an evening meeting.	GWG, birth weight of term infants, maternal fasting glucose levels at 30 weeks of gestation, maternal percentage of fat, incidence of operative deliveries, postpartum weight	606 / 576 (95.0%)

Table S1. cont.

Author (Year)	Period	Original inclusion/exclusion criteria	Country	Method to measure GWG	Intervention	Intervention Detail	Outcome	Original i-WIP N<br>N (%) in this analysis
Stafne *et al*. (2012)	April 2007 to June 2009 and October 2007 to January 2009	Inclusion criteria: Residing within 30 min drive from the hospital and available to attend weekly appointment, ≥18 years, singleton pregnancy. Exclusion criteria: High-risk pregnancies, diseases that could interfere with participation.	Norway	Pre-pregnancy: na. Early pregnancy: measured at 18-22 week's gestation	Exercise	Exercise sessions, once per week for 12 weeks (60 minutes each), in groups of 8–15 women, instructed by a physiotherapist. Offered between 20 and 36 weeks of pregnancy. Included aerobic activity, strength training and balance exercises. Women encouraged to follow a written home exercise program at least twice per week (45 minutes each).	Self-reported lumbopelvic pain and sick leave due to lumbopelvic pain.	855<br>699 (81.8%)
Vinter *et al*. (2011)	October 2007 to October 2010	Inclusion criteria: 18–40 years, 10–14 weeks' gestation, BMI 30–45 kg/m^2. Exclusion criteria: chronic medical conditions, prior serious obstetric complications, alcohol or drug abuse, non-Danish speaking, multiple pregnancy, positive OGTT.	Denmark	Pre-pregnancy: self-reported. Early pregnancy: measured at 10-14 week's gestation	Mixed	Dietary counselling and physical activity (The LiP Study - Lifestyle in Pregnancy). Four dietary counselling by dietitians at 15, 20, 28, and 35 weeks' gestation. Dietary advice was based on the official Danish recommendations, and aimed to limit GWG to 5 kg. Provided with free full-time membership in a fitness centre for 6 months, with exercise classes once per week (60 minutes each) with physiotherapists. Encouraged to be moderately physically active every day (30–60 minutes per day), provided with a pedometer to motivate and improve daily activity.	GWG, preeclampsia, pregnancy-induced hypertension, GDM, caesarean section, macrosomia, and admission to neonatal intensive care unit.	304<br>292 (96.1%)
Vitolo *et al*. (2011)	January 2007 to May 2008	Inclusion criteria: 10-29 weeks' gestation. Exclusion criteria: positive HIV test previous diagnosis of diabetes, hypertension, anaemia, any conditions preventing women from undertaking, exercise in pregnancy, and >35 years	Brazil	Pre-pregnancy: self-reported. Early pregnancy: collected in first trimester (method not described)	Diet	Dietary counselling provided according to nutritional needs. Underweight women advised to increase energy density of the diet, include two high-energy snacks and fruit daily. Well-nourished women advised to consume six portions fruit and vegetables and restrict energy dense foods. Overweight women were advised on meal timing, portion sizes, to restrict energy dense and processed foods.	GWG, gestational diabetes, pre-eclampsia, infant birth and premature delivery (<37wks), 1st and 5th min Apgar score, head circumference, type of delivery, birth weight <2500g	315<br>268 (85.1%)

For peer review only

Table S1. cont.

Author (Year)	Period	Original inclusion/exclusion criteria	Country	Method to measure GWG	Intervention	Intervention Detail	Outcome	Original i-WIP N / N (%) in this analysis
Walsh *et al*. (2012)	January 2007 to January 2011	Inclusion criteria: Secundigravid women, previous macrosomic infant (≥4 kg). Exclusion criteria: underlying medical disorders, including a previous history of gestational diabetes, use of any drugs, unable to provide full informed consent, <18 years, >18 weeks' gestation, and multiple pregnancy.	Ireland	Pre-pregnancy: na. Early pregnancy: measured at 12-15 week's gestation	Diet	One dietary education session (2 hours), with the research dietitian, at 15 weeks' gestation, groups of 2-6 women. Reinforcement of diet at 28 and 34 weeks' gestation. Advised on a low glycaemic index diet, and general healthy eating guidelines for pregnancy. Received written resources about low glycaemic index diet.	Birth weight, GWG, glucose intolerance.	800 / 517 (64.6%)
Wolff *et al*. (2008)	Not described	Inclusion criteria: non-diabetic, Caucasian, BMI >30 kg/m^2, 15 weeks' gestation. Exclusion criteria: smoker, <18 or >45 years, multiple pregnancy, medical complications known to affect fetal growth adversely or to contraindicate limitation of weight gain.	Denmark	Pre-pregnancy: self-reported. Early pregnancy: measured at 15 week's gestation	Diet	Dietary consultations with a dietitian, 10 times during pregnancy (60 minutes each). Advised to eat a healthy diet according to the official Danish dietary recommendations. Aim to restrict GWG to 6–7kg.	GWG, pregnancy-induced increases in insulin, leptin, and glucose. Weight retentions at 4 weeks' postpartum	59 / 55 (93.2%)

GWG: gestational weight gain; BMI: body mass index; GDM; gestational diabetes; OGTT: oral glucose tolerance test; IOM: Institute of Medicine

For peer review only

Table S2. Global classification of risk of bias on study level for trials included

Study Team	Item 1: Randomisation	Item 2: Allocation concealment	Item 3: Blinding of participants	Item 4: Blinding of outcome Assessment	Item 5: Incomplete outcome data	Item 6: Selective reporting	Global Risk of bias
Althuizen *et al*. (2013)	⊕	⊕	?	⊕	⊕	?	Low/Medium
Baciuk *et al*. (2008)	⊕	⊕	⊖	⊕	⊕	⊕	Low/Medium
Bogaerts *et al*. (2013)	⊕	?	⊖	⊖	⊕	⊕	High
Haakstad *et al*. (2011)	⊕	⊕	?	⊕	⊕	⊖	Low/Medium
Harrison *et al*. (2013)	⊕	⊕	?	⊕	⊕	⊕	Low/Medium
Jeffries *et al*. (2009)	⊕	⊕	?	⊕	⊕	⊕	Low/Medium
Khaledan *et al*. (2010)	⊕	?	⊖	⊖	⊕	⊕	High
Khoury *et al*. (2005)	⊕	⊕	?	⊕	⊕	⊕	Low/Medium
Nascimento *et al*. (2011)	⊕	⊕	⊖	⊖	⊕	⊕	High
Oostdam *et al*. (2012)	⊕	⊕	⊖	⊕	⊖	⊕	High
Petrella *et al*. (2014)	⊕	⊖	⊖	⊖	⊕	⊖	High
Phelan *et al*. (2011)	⊕	⊕	?	⊕	⊕	⊕	Low/Medium
Poston *et al*. (2015)	?	?	⊖	⊖	?	?	High
Rauh *et al*. (2013)	⊕	⊕	⊖	⊖	⊕	?	High
Renault *et al*. (2014)	⊕	⊕	⊖	⊖	⊕	⊖	High
Sagedal *et al*. (2016)	⊕	⊕	?	⊕	?	?	Low/Medium
Stafne *et al*. (2012)	⊕	⊕	⊖	⊖	⊕	⊕	High
Vinter *et al*. (2011)	⊕	⊕	⊖	⊖	⊕	⊖	High
Vitolo *et al*. (2011)	⊕	⊖	⊖	⊕	⊕	⊖	High
Walsh *et al*. (2012)	⊕	⊕	⊖	?	⊕	⊖	Low/Medium
Wolff *et al*. (2008)	⊕	⊕	?	⊖	⊖	⊕	High

Legend		
⊕ Low	⊖ High	? Unclear

Table S3. Individual Study Results – Gestational Weight Gain per Week

		Intervention			Control			Mean Difference (95% CI)	*P**
**Study name**	**Education**	**N**	**Mean**	**SD**	**N**	**Mean**	**SD**		
Althuizen *et al*. (2013)	Lower	49	0.39	0.34	49	0.47	0.26	-0.08 (-0.02, 0.04)	0.205
	Higher	40	0.47	0.20	49	0.43	0.27	0.04 (-0.06, 0.15)	0.391
Baciuk *et al*. (2008)	Lower	29	0.48	0.23	31	0.51	0.25	-0.04 (-0.16, 0.09)	0.561
	Higher	4	0.41	0.19	4	0.52	0.31	-0.11 (-0.55, 0.33)	0.557
Bogaerts *et al*. (2013)	Lower	73	0.31	0.26	40	0.41	0.25	-0.10 (-0.21, 0.01)	0.064
	Higher	60	0.29	0.24	23	0.42	0.28	-0.14 (-0.27, -0.01)	0.044
Haakstad *et al*. (2011)	Lower	7	0.47	0.15	6	0.39	0.18	0.08 (-0.13, 0.29)	0.411
	Higher	34	0.48	0.17	36	0.51	0.17	-0.03 (-0.11, 0.05)	0.432
Harrison *et al*. (2013)	Lower	56	0.41	0.18	56	0.46	0.25	-0.05 (-0.13, 0.03)	0.197
	Higher	40	0.44	0.19	35	0.47	0.26	-0.02 (-0.13, 0.08)	0.668
Jeffries *et al*. (2009)	Lower	42	0.45	0.18	32	0.47	0.14	-0.03 (-0.12, 0.05)	0.498
	Higher	83	0.43	0.17	79	0.46	0.16	-0.03 (-0.08, 0.03)	0.401
Khaledan *et al*. (2010)	Lower	11	0.61	0.26	14	0.73	0.33	-0.12 (-0.38, 0.13)	0.336
	Higher	6	0.55	0.23	6	0.51	0.17	0.04 (-0.22, 0.30)	0.736
Khoury *et al*. (2005)	Lower	21	0.56	0.20	14	0.54	0.17	0.01 (-0.12, 0.15)	0.822
	Higher	74	0.52	0.19	89	0.57	0.17	-0.05 (-0.10, 0.01)	0.108
Nascimento *et al*. (2011)	Lower	34	0.37	0.22	39	0.40	0.22	-0.03 (-0.13, 0.07)	0.562
	Higher	3	0.40	0.07	1	0.80	na	-0.40 (-0.73, -0.07)	0.035
Oostdam *et al*. (2012)	Lower	28	0.41	0.34	24	0.32	0.20	0.09 (-0.07, 0.25)	0.245
	Higher	10	0.40	0.21	13	0.44	0.21	-0.04 (-0.22, 0.14)	0.680
Petrella *et al*. (2014)	Lower	25	0.22	0.29	27	0.33	0.22	-0.11 (-0.26, 0.04)	0.137
	Higher	5	0.34	0.12	1	0.35	na	-0.01 (-0.39, 0.37)	0.950
Phelan *et al*. (2011)	Lower	27	0.50	0.25	36	0.46	0.19	0.04 (-0.07, 0.15)	0.469
	Higher	170	0.48	0.16	162	0.52	0.19	-0.03 (-0.07, 0.01)	0.094
Poston *et al*. (2015)	Lower	113	0.26	0.20	120	0.27	0.22	0.00 (-0.06, 0.06)	0.853
	Higher	66	0.20	0.21	79	0.26	0.20	-0.06 (-0.14, 0.01)	0.116
Rauh *et al*. (2013)	Lower	64	0.45	0.15	43	0.45	0.18	-0.03 (-0.11, 0.04)	0.904
	Higher	89	0.43	0.13	35	0.47	0.15	-0.07 (-0.13, -0.01)	0.120
Renault *et al*. (2014)	Lower	56	0.38	0.22	28	0.48	0.22	-0.09 (-0.20, 0.01)	0.082
	Higher	186	0.35	0.21	100	0.39	0.19	-0.04 (-0.09, 0.01)	0.169
Sagedal *et al*. (2017)	Lower	89	0.54	0.21	93	0.57	0.21	-0.03 (-0.09, 0.03)	0.339
	Higher	199	0.48	0.20	195	0.52	0.19	-0.04 (-0.08, 0.00)	0.037
Stafne *et al*. (2012)	Lower	31	0.53	0.15	38	0.59	0.23	-0.06 (-0.16, 0.03)	0.178
	Higher	339	0.51	0.17	291	0.52	0.17	-0.01 (-0.03, 0.02)	0.617
Vinter *et al*. (2011)	Lower	59	0.31	0.25	69	0.36	0.22	-0.06 (-0.15, 0.03)	0.212
	Higher	85	0.33	0.20	79	0.38	0.22	-0.05 (-0.12, 0.02)	0.158
Vitolo *et al*. (2011)	Lower	79	0.41	0.18	83	0.45	0.21	-0.04 (-0.10, 0.02)	0.199
	Higher	61	0.42	0.15	45	0.49	0.22	-0.07 (-0.15, 0.00)	0.053
Walsh *et al*. (2012)	Lower	60	0.46	0.16	62	0.52	0.21	-0.06 (-0.13, 0.01)	0.077
	Higher	188	0.49	0.16	207	0.53	0.18	-0.04 (-0.08, -0.01)	0.021
Wolff *et al*. (2008)	Lower	26	0.26	0.16	27	0.40	0.24	-0.14 (-0.25, -0.02)	0.021
	Higher	0†	na		2	0.38	0.24	na	na

Mean difference is calculated as intervention minus control
* P-value is independent-sample t-test between intervention and control
† T-test cannot be computed because at least one of the groups is empty.
Lower education: secondary education or less. Higher education: at least some tertiary education

For peer review only

Table S4. Comparison of maternal characteristics among women included and excluded in the IPD meta-analysis.

	Included		Excluded		*P**
	N		N		
**Early pregnancy weight** (kg) [a]	5183	78.60 (18.56)	4814	87.78 (51.06)	0.002
**Pre-pregnancy weight** (kg) [a]	5183	76.06 (18.74)	2398	85.82 (123.03)	0.030
**Height** (cm) [a]	5183	166.07 (7.03)	6697	169.30 (63.03)	<0.001
**Pre-pregnancy BMI** (kg/m^2) [a]	5183	27.57 (6.55)	2615	27.31 (12.56)	<0.001
**Gestational age** (weeks)					
**Baseline** [c]	5183	14.5 (4.0 - 32.0)	4478	14.0 (11.0 – 17.0)	<0.001
**Final follow-up** [c]	5183	36.6 (24.0 - 44.0)	6995	38.0 (35.0 – 41.0)	<0.001
**Delivery** [c]	5172	39.7 (26.0 - 44.0)	7007	40.0 (38.85 – 41.0)	0.022
**Infant birth weight** (g) [a]	5164	3531.73 (547.90)	7026	3701.22 (1479.82)	<0.001
**Maternal age** (years) [a]	5183	29.90 (4.97)	7041	30.80 (8.36)	<0.001
**Ethnicity**					
Caucasian [b]	3725	3291 (88.3)	6415	5588 (87.1)	<0.001
Asian [b]		84 (0.02)		231 (0.04)	
Black [b]		123 (0.03)		462 (0.08)	
Central/South American [b]		88 (0.02)		47 (0.01)	
Middle East (including Iran & Turkey) [b]		63 (0.01)		13 (0.00)	
Other [b]		76 (0.02)		74 (0.01)	

Data are presented as [a] mean (SD) [b] n (%) and [c] mean (range of minimum and maximum).

*P-value: T-tests and chi-square

For peer review only

Figure S1. Assessment of small study effects on trials in IPD meta-analysis of low educational attainment and gestational weight gain per week, controlling for BMI and intervention.

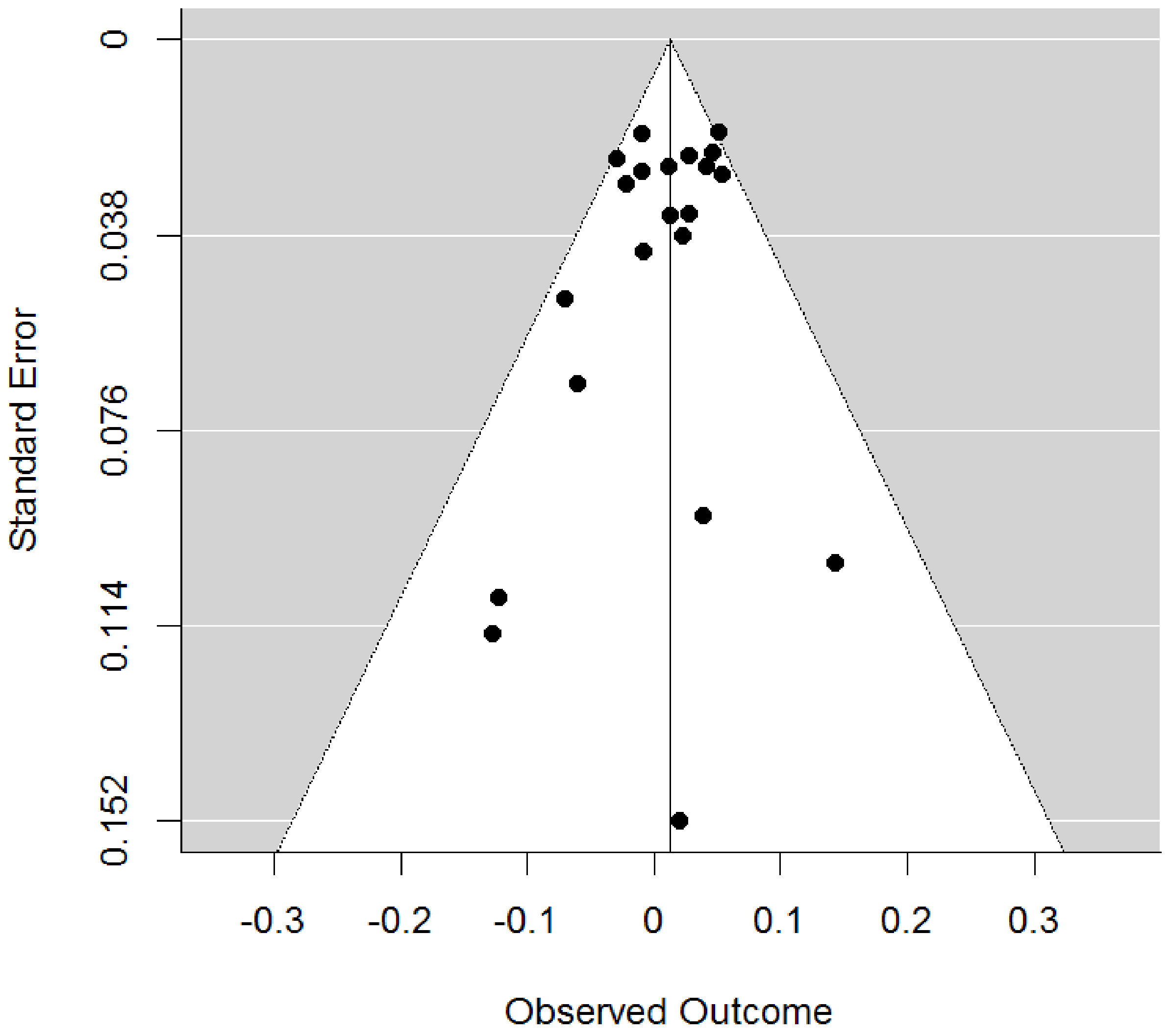

Figure S2. Forest plot of the IPD meta-analysis of low educational attainment (treatment effect [TE]) and gestational weight gain per week, controlling for BMI and intervention.

Study	TE	seTE	95%-CI	Weight
Althuizen, 2012	-0.01	0.0412	-0.01 [-0.09; 0.07]	3.3%
Baciuk, 2008	0.04	0.0930	0.04 [-0.14; 0.22]	0.8%
Bogaerts, 2012	0.02	0.0383	0.02 [-0.05; 0.10]	3.7%
Haakstad, 2011	-0.07	0.0506	-0.07 [-0.17; 0.03]	2.3%
Harrison, 2013	0.03	0.0339	0.03 [-0.04; 0.09]	4.5%
Jeffries, 2009	0.01	0.0247	0.01 [-0.04; 0.06]	7.1%
Khaledan, 2010	0.14	0.1020	0.14 [-0.06; 0.34]	0.6%
Khoury, 2005	0.01	0.0342	0.01 [-0.05; 0.08]	4.5%
Nascimento, 2011	-0.13	0.1157	-0.13 [-0.35; 0.10]	0.5%
Oostdam, 2012	-0.06	0.0670	-0.06 [-0.19; 0.07]	1.4%
Petrella, 2013	-0.12	0.1089	-0.12 [-0.34; 0.09]	0.6%
Phelan, 2011	-0.01	0.0256	-0.01 [-0.06; 0.04]	6.8%
Poston, (unpub)	0.04	0.0247	0.04 [-0.01; 0.09]	7.1%
Rauh, 2013	0.03	0.0227	0.03 [-0.02; 0.07]	7.9%
Renault, 2013	0.05	0.0262	0.05 [0.00; 0.11]	6.6%
Sagedal, (unpub)	0.05	0.0179	0.05 [0.02; 0.09]	10.2%
Stafne, 2012	0.05	0.0219	0.05 [0.00; 0.09]	8.2%
Vinter, 2011	-0.02	0.0282	-0.02 [-0.08; 0.03]	5.9%
Vitolo, 2011	-0.03	0.0230	-0.03 [-0.08; 0.02]	7.7%
Walsh, 2012	-0.01	0.0183	-0.01 [-0.05; 0.03]	10.0%
Wolff, 2008	0.02	0.1523	0.02 [-0.28; 0.32]	0.3%
**Random effects model**			**0.01 [0.00; 0.03]**	**100.0%**

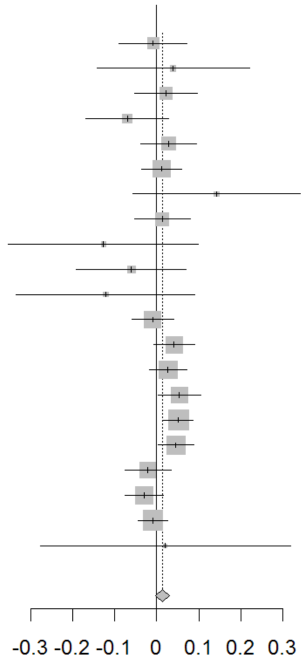

For peer review only

Appendix 1. Identification and selection of studies in individual participant data (IPD) meta-analysis for the i-WIP Database

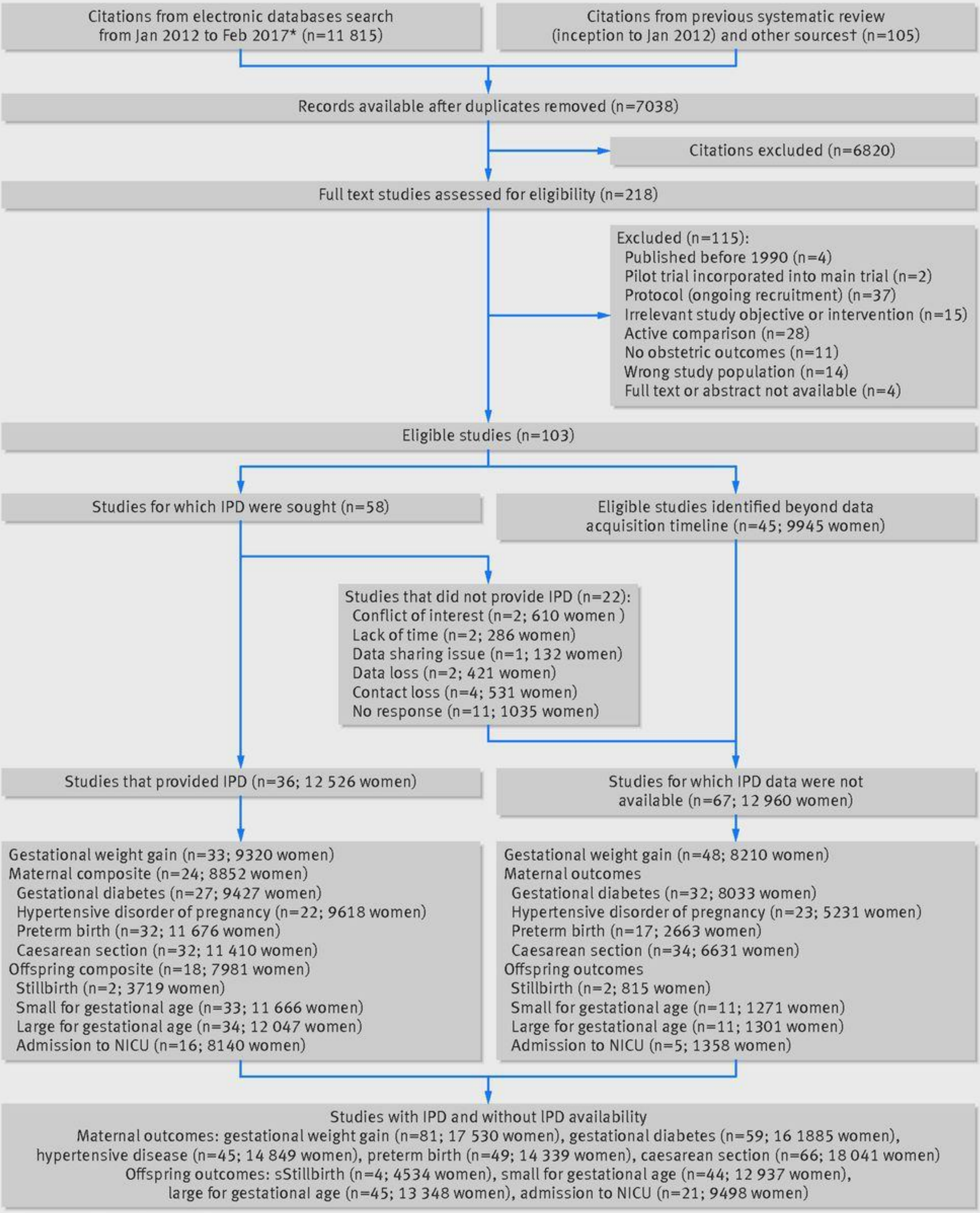

NICU=neonatal intensive care unit
*Database search was updated in October 2013 (9359 records), March 2015 (3551 records), January 2016 (1373 records), and February 2017 (1547 records)
†Other sources: reference search, personal communication, and Google search

Appendix 2. Search strategy for identification of randomised trials on lifestyle interventions in pregnancy and maternal and offspring outcomes (original search strategy from BMJ 2017;358:j3119)

Search strategy for Medline via Ovid

Item	Term
1	Pregnancy/
2	pregnan*.tw.
3	Gravidity/
4	gravid*.tw.
5	gestation*.tw.
6	Pregnant Women/
7	pregnant wom#n.tw.
8	(child adj3 bearing).tw.
9	childbearing.tw.
10	matern*.tw.
11	or/1-10
12	Weight Gain/ph [Physiology]
13	weight gain*.tw.
14	Weight Loss/ph [Physiology]
15	weight loss*.tw.
16	weight change*.tw.
17	Obesity/dh, me, ph, pc, px, th [Diet Therapy, Metabolism, Physiology, Prevention & Control, Psychology, Therapy]
18	obes*.tw.
19	Adiposity/ph [Physiology]
20	adipos*.tw.
21	Overweight/dh, me, ph, pc, px, th [Diet Therapy, Metabolism, Physiology, Prevention & Control, Psychology, Therapy]
22	overweight*.tw.
23	Body Mass Index/
24	bmi.tw.
25	or/12-24
26	exp Randomized Controlled Trial/
27	"randomized controlled trial".pt.
28	"controlled clinical trial".pt.
29	(random$ or placebo$).tw,sh.
30	((singl$ or double$ or triple$ or treble$) and (blind$ or mask$)).tw,sh.
31	single-blind method/
32	double-blind method/
33	or/26-32
34	11 and 25 and 33
35	exp Animals/
36	(rat$ or mouse or mice or hamster$ or animal$ or dog$ or cat$ or bovine or sheep or lamb$).af.
37	35 or 36
38	Humans/
39	human$.tw,ot,kf.
40	37 or 38
41	37 not (37 and 40)
42	34 not 41

Appendix 3. Institute of Medicine Guidelines for gestational weight gain per week in trimester 2 and 3, and extrapolated to trimester 1

Participant level data: A measured, early-pregnancy weight was available for 4,888 participants. Early-pregnancy weight was estimated using self-reported pre-pregnancy weight for the 295 participants missing this variable. Estimated weight gain per week from pre-pregnancy to early-pregnancy was calculated by the following formula, using only participants with both pre- and early-pregnancy weight, n = 3001:

$$\textit{Weight gain from pre- to early-pregnancy} = \frac{\textit{Early-pregnancy weight} - \textit{pre-pregnancy weight}}{\textit{Baseline GA}}$$

Mean weight gain from pre- to early-pregnancy was 0.175 kg/week. We used the following formula to estimate early-pregnancy weight for the 295 participants missing this variable:

$$\text{Early-pregnancy weight} = (\text{Baseline GA}*0.175\text{ kg}) + \text{pre-pregnancy weight}$$

We used pre-pregnancy weight to categorise participants according to BMI. Self-reported pre-pregnancy weight was available for 3,296 participants, and estimated using early-pregnancy weight for the remaining 1,887 participants. We used the following formula to estimate pre-pregnancy weight:

$$\text{Pre-pregnancy weight} = \text{Early-pregnancy weight} - (\text{Baseline GA}*0.175\text{ kg})$$

Total weight gain was calculated by subtracting early-pregnancy weight from final follow-up weight. Weekly GWG was calculated using the following formula:

$$\textit{Weight Gain per week} = \frac{\textit{Total } GWG}{\textit{Final follow-up GA} - \textit{Baseline GA}}$$

Trimester 2 and 3 GWG/week was required for comparison with IOM 2009 guidelines. This was calculated using a four-step approach;

1. Trimester 1 GWG/week guidelines were extrapolated from the IOM guidelines(15) .

BMI Category	Total GWG (IOM Guidelines)			Rate of weight gain for Trimester 2 and 3 (IOM Guidelines)		
	Inadequate	Adequate	Excess	Mean	Range	
**Underweight**	<12.5	12.5 - 18.0	>18.0	0.51	0.44	0.58
**Normal**	<11.5	11.5 - 16.0	>16.0	0.42	0.35	0.50
**Overweight**	<7.0	7.0 - 11.5	>11.5	0.28	0.23	0.33
**Obese**	<5.0	5.0 - 9.0	>9.0	0.22	0.17	0.27

2. The number of weeks in trimester 1 that the participant was enrolled in the intervention was calculated by subtracting the baseline GA from 13. If the participant enrolled in the study at greater than 13 weeks, trimester 1 weeks were set as 0. The number of weeks in trimester 2 and 3 that the participant was enrolled in the intervention were calculated by subtracting the 13 from the final follow-up GA.
3. The rate of weight gain for Trimester 1 was calculated:

(*Rate of weight gain for Trimester 2 and 3*)**27= Total weight gain for Trimester 2 and 3* ***(A)***

Total GWG [ranges for adequate] - Total weight gain for Trimester 2 and 3 [ranges] = Total weight gain for Trimester 1 [ranges] ***(B)***

$$\frac{\text{Lower range Trimester 1 weight gain} + \text{Upper range Trimester 1 weight gain}}{2} = \text{Mean weight gain for Trimester 1 } \textbf{(C)}$$

$$\frac{\text{Total weight gain for Trimester 1 [mean and ranges]}}{13} = \text{Rate of weight gain for Trimester 1 } \textbf{(D)}$$

BMI Category	(A) Total weight gain for Trimester 2 and 3			Total weight gain for Trimester 1			(D) Rate of weight gain for Trimester 1		
	Mean	Range		(C) Mean	(B) Range		Mean	Range	
**Underweight**	13.77	11.88	15.66	1.48	0.62	2.34	0.11	0.05	0.18
**Normal**	11.34	9.45	13.50	2.28	2.05	2.50	0.18	0.16	0.19
**Overweight**	7.56	6.21	8.91	1.69	0.79	2.59	0.13	0.06	0.20
**Obese**	5.94	4.59	7.29	1.06	0.41	1.71	0.08	0.03	0.13

For peer review only

4. The contribution of trimester 1 weight gain to total GWG was estimated using the mean trimester 1 GWG/week (above) and the following equation:

$$\text{T1 weight gain} = (\text{T1 weight gain per week for BMI}) * (\text{T1 weeks in intervention})$$

5. Trimester 2 and 3 weight gain per week was calculated using the following equation:

$$\textit{T2 and T3 weight gain per week} = \frac{\textit{Total weight gain} - \textit{T1 weight gain}}{\textit{Weeks in T2 and T3}}$$

For peer review only

PRISMA-IPD Checklist of items to include when reporting a systematic review and meta-analysis of individual participant data (IPD)

PRISMA-IPD Section/topic	Item No	Checklist item	Reported on page
Title			
Title	1	Identify the report as a systematic review and meta-analysis of individual participant data.	1
Abstract			
Structured summary	2	Provide a structured summary including as applicable:	5-6
		**Background**: state research question and main objectives, with information on participants, interventions, comparators and outcomes.	
		**Methods**: report eligibility criteria; data sources including dates of last bibliographic search or elicitation, noting that IPD were sought; methods of assessing risk of bias.	
		**Results**: provide number and type of studies and participants identified and number (%) obtained; summary effect estimates for main outcomes (benefits and harms) with confidence intervals and measures of statistical heterogeneity. Describe the direction and size of summary effects in terms meaningful to those who would put findings into practice.	
		**Discussion:** state main strengths and limitations of the evidence, general interpretation of the results and any important implications.	
		**Other:** report primary funding source, registration number and registry name for the systematic review and IPD metaanalysis.	

For peer review only

Introduction			
Rationale	3	Describe the rationale for the review in the context of what is already known.	8-9
Objectives	4	Provide an explicit statement of the questions being addressed with reference, as applicable, to participants, interventions, comparisons, outcomes and study design (PICOS). Include any hypotheses that relate to particular types of participant-level subgroups.	9
Methods			
Protocol and registration	5	Indicate if a protocol exists and where it can be accessed. If available, provide registration information including registration number and registry name. Provide publication details, if applicable.	9
Eligibility criteria	6	Specify inclusion and exclusion criteria including those relating to participants, interventions, comparisons, outcomes, study design and characteristics (e.g. years when conducted, required minimum follow-up). Note whether these were applied at the study or individual level i.e. whether eligible participants were included (and ineligible participants excluded) from a study that included a wider population than specified by the review inclusion criteria. The rationale for criteria should be stated.	9
Identifying studies - information sources	7	Describe all methods of identifying published and unpublished studies including, as applicable: which bibliographic databases were searched with dates of coverage; details of any hand searching including of conference proceedings; use of study registers and agency or company databases; contact with the original research team and experts in the field; open adverts and surveys. Give the date of last search or elicitation.	9 and Protocol paper attached

For peer review only

Identifying studies - search	8	Present the full electronic search strategy for at least one database, including any limits used, such that it could be repeated.	9 and Protocol paper attached

Study selection processes	9	State the process for determining which studies were eligible for inclusion.	9 and Protocol paper attached
Data collection processes	10	Describe how IPD were requested, collected and managed, including any processes for querying and confirming data with investigators. If IPD were not sought from any eligible study, the reason for this should be stated (for each such study).	9 and Protocol paper attached
		If applicable, describe how any studies for which IPD were not available were dealt with. This should include whether, how and what aggregate data were sought or extracted from study reports and publications (such as extracting data independently in duplicate) and any processes for obtaining and confirming these data with investigators.	
Data items	11	Describe how the information and variables to be collected were chosen. List and define all study level and participant level data that were sought, including baseline and follow-up information. If applicable, describe methods of standardising or translating variables within the IPD datasets to ensure common scales or measurements across studies.	10
IPD integrity	A1	Describe what aspects of IPD were subject to data checking (such as sequence generation, data consistency and completeness, baseline imbalance) and how this was done.	10

For peer review only

Risk of bias assessment in individual studies.	12	Describe methods used to assess risk of bias in the individual studies and whether this was applied separately for each outcome. If applicable, describe how findings of IPD checking were used to inform the assessment. Report if and how risk of bias assessment was used in any data synthesis.	10
Specification of outcomes and effect measures	13	State all treatment comparisons of interests. State all outcomes addressed and define them in detail. State whether they were pre-specified for the review and, if applicable, whether they were primary/main or secondary/additional outcomes. Give the principal measures of effect (such as risk ratio, hazard ratio, difference in means) used for each outcome.	10-11
Synthesis methods	14	Describe the meta-analysis methods used to synthesise IPD. Specify any statistical methods and models used.<br>Issues should include (but are not restricted to):<br>• Use of a one-stage or two-stage approach.<br>• How effect estimates were generated separately within each study and combined across studies (where applicable).<br>• Specification of one-stage models (where applicable) including how clustering of patients within studies was accounted for.<br>• Use of fixed or random effects models and any other model assumptions, such as proportional hazards.<br>• How (summary) survival curves were generated (where applicable).<br>• Methods for quantifying statistical heterogeneity (such as I^2 and $\square^2$).<br>• How studies providing IPD and not providing IPD were analysed together (where applicable).<br>• How missing data within the IPD were dealt with (where applicable).	11-12

For peer review only

Exploration of variation in effects	A2	If applicable, describe any methods used to explore variation in effects by study or participant level characteristics (such as estimation of interactions between effect and covariates). State all participant-level characteristics that were analysed as potential effect modifiers, and whether these were pre-specified.	12
Risk of bias across studies	15	Specify any assessment of risk of bias relating to the accumulated body of evidence, including any pertaining to not obtaining IPD for particular studies, outcomes or other variables.	10
Additional analyses	16	Describe methods of any additional analyses, including sensitivity analyses. State which of these were pre-specified.	12

Results			
Study selection and IPD obtained	17	Give numbers of studies screened, assessed for eligibility, and included in the systematic review with reasons for exclusions at each stage. Indicate the number of studies and participants for which IPD were sought and for which IPD were obtained. For those studies where IPD were not available, give the numbers of studies and participants for which aggregate data were available. Report reasons for non-availability of IPD. Include a flow diagram.	13 and Table 1
Study characteristics	18	For each study, present information on key study and participant characteristics (such as description of interventions, numbers of participants, demographic data, unavailability of outcomes, funding source, and if applicable duration of follow-up). Provide (main) citations for each study. Where applicable, also report similar study characteristics for any studies not providing IPD.	13 and Table S1
IPD integrity	A3	Report any important issues identified in checking IPD or state that there were none.	13 and Table S2

For peer review only

Risk of bias within studies	19	Present data on risk of bias assessments. If applicable, describe whether data checking led to the up-weighting or down-weighting of these assessments. Consider how any potential bias impacts on the robustness of meta-analysis conclusions.	13 and Table S2
Results of individual studies	20	For each comparison and for each main outcome (benefit or harm), for each individual study report the number of eligible participants for which data were obtained and show simple summary data for each intervention group (including, where applicable, the number of events), effect estimates and confidence intervals. These may be tabulated or included on a forest plot.	14 and Table S3
Results of syntheses	21	Present summary effects for each meta-analysis undertaken, including confidence intervals and measures of statistical heterogeneity. State whether the analysis was pre-specified, and report the numbers of studies and participants and, where applicable, the number of events on which it is based.	14-15. Tables 2-5
		When exploring variation in effects due to patient or study characteristics, present summary interaction estimates for each characteristic examined, including confidence intervals and measures of statistical heterogeneity. State whether the analysis was pre-specified. State whether any interaction is consistent across trials.	
		Provide a description of the direction and size of effect in terms meaningful to those who would put findings into practice.	
Risk of bias across studies	22	Present results of any assessment of risk of bias relating to the accumulated body of evidence, including any pertaining to the availability and representativeness of available studies, outcomes or other variables.	13

For peer review only

Additional analyses	23	Give results of any additional analyses (e.g. sensitivity analyses). If applicable, this should also include any analyses that incorporate aggregate data for studies that do not have IPD. If applicable, summarise the main meta-analysis results following the inclusion or exclusion of studies for which IPD were not available.	15
Discussion			
Summary of evidence	24	Summarise the main findings, including the strength of evidence for each main outcome.	16
Strengths and limitations	25	Discuss any important strengths and limitations of the evidence including the benefits of access to IPD and any limitations arising from IPD that were not available.	18-19
Conclusions	26	Provide a general interpretation of the findings in the context of other evidence.	19
Implications	A4	Consider relevance to key groups (such as policy makers, service providers and service users). Consider implications for future research.	17-18

Funding			
Funding	27	Describe sources of funding and other support (such as supply of IPD), and the role in the systematic review of those providing such support.	6

For peer review only

For peer review only
